# New and known species of the genus *Campylaimus* Cobb, 1920 (Nematoda: Araeolaimida: Diplopeltidae) from North European marine habitats

**DOI:** 10.3897/BDJ.7.e46545

**Published:** 2019-10-28

**Authors:** Oleksandr Holovachov

**Affiliations:** 1 Swedish Museum of Natural History, Stockholm, Sweden Swedish Museum of Natural History Stockholm Sweden

**Keywords:** Bratten, Diplopeltidae, Gullmarn fjord, identification key, new species, revision, Skagerrak, Sweden, taxonomy

## Abstract

**Background:**

The genus *Campylaimus* is a broadly distributed but relatively uncommon genus of marine and brackish nematodes with 20 nominal species and one *nomen nudum.* Many species descriptions and redescriptions are based on very few (single) individuals, which limits our understanding of inter- and intra-specific variability and morphology-based species boundaries.

**New information:**

Two new species were found in the Skagerrak off the west coast of Sweden. *Campylaimus triclados* sp. n. is characterised by body 0.59–0.69 mm long; cuticle without longitudinal striation; anteriormost edge of the amphid anterior to the oral opening; dorsal limb of the amphid equal to 1.6–2.5 labial region diameters in male, 1.9 labial region diameters in female; ventral limb of the amphid extends towards anterior part of the intestine in male and midpharynx in female, 4.3–5.5 times the length of the dorsal limb in male and 2.2–2.8 times the length of the dorsal limb in female; ventral limb of the amphid is as wide as the dorsal limb; interamphideal space extends further than the posterior end of the dorsal limb but not reaching the posterior end of the ventral limb; secretory-excretory pore opens posterior to the cardia; spicules 19–24 µm long; two precloacal supplements; tail equal to 4.3–5.6 anal body diameters in length, with conoid terminal part. *Campylaimus longispiculus* sp. n. is characterised by body 0.56–0.65 mm long; cuticle without longitudinal striation; anteriormost edge of the amphid anterior to the oral opening; dorsal limb of the amphid equal to 1.8–2.1 labial region diameters in male; ventral limb of the amphid extends towards anterior part of the intestine in male, 2.8–3.5 times the length of the dorsal limb in male; ventral limb of the amphid is as wide as the dorsal limb; interamphideal space absent; secretory-excretory pore opens posterior to the cardia; spicules 28–35 µm long; two precloacal supplements; tail equal to 4.9–5.3 anal body diameters in length, with clavate terminal part. In addition, following nine species are new records for the fauna of Sweden: *Campylaimus amphidialis, C. inaequalis*, *C. lefeverei*, *C. minutus*, *C. orientalis*, *C. rimatus*, *C. siwaschensis*, *C. striatus* and *C. tkatchevi*.

## Introduction

The genus *Campylaimus* Cobb, 1920 is one of the few nematode genera with its oral opening positioned subterminally, shifted to the dorsal side of the body. This unusual feature, subdorsal position of the mouth in nematodes, was even discussed by Fitch and Sudhaus (2002) in a context of evolution of body plans. However, precise morphology of the foregut in this genus was not always clearly understood by the researchers. The original description of the type species of the genus *Campylaimus, C. inaequalis* Cobb, 1920 stated that "*the mouth is ... on the ventral side of the head a little behind the anterior extremity*" (Cobb 1920). Even though this error was quickly corrected by Gerlach (1950), who clearly described the mouth to be placed on the dorsal side of the anterior end, Timm (1961) and Huang and Zhang (2006) followed the mistake made by Cobb (1920). Fortunately, all other publications, including most recent revisions by Villares et al. (2013) and Fadeeva et al. (2016), correctly describe the subdorsal to dorsal position of the mouth opening in all 20 known species of the genus *Campylaimus* (*Campylaimus abditus* Bussau, 1993 was described in an unpublished doctoral thesis (Bussau 1993) and must be considered a *nomen nudum*).

Twelve species of the genus *Campylaimus* were found during ongoing studies of marine nematodes along Swedish coasts and one species was collected in Germany. Two species are described as new to science and nine species are new to the fauna of Sweden. One species, *Campylaimus gerlachi* Timm, 1961 has already been recorded from the Baltic (Ólafsson and Elmgren 1997), but is re-described here in order to clarify its taxonomic status and its affinities to closely-related species.

## Materials and methods

Bottom sediment samples were collected in multiple locations along the coast of Sweden: in the Baltic, Bothnia Sea and Bothnia Gulf, southern part of the Skagerrak and in Gullmarn Fjord. All samples were collected with a bottom dredge or box corer and further sieved in the laboratory before fixation. Nematodes were extracted from samples using a decanting and sieving method (smallest mesh sizes: 45 µm or 70 µm). Freshwater was used during sieving to induce an osmotic shock in nematodes, inducing their detachment from the substrate. Material retained on the sieves was immediately fixed in a 4% formaldehyde solution in the sea water.

For light microscopy, formaldehyde-preserved specimens were transferred to pure glycerine using the Seinhorst (1959) rapid method as modified by De Grisse (1969). Permanent nematode mounts on the glass slides were prepared using the paraffin wax ring method. All curved structures were measured along the curved median line. Measurements in all tables are presented in µm as mean ±SD and (range) where appropriate. Species descriptions and illustrations include only diagnostic, species-specific characters and are written in a "telegraphic style", while features characteristic to all species of the genus *Campylaimus* are given in the extended genus diagnosis only. Terminology follows Maggenti (2005). Abbreviations follow Decraemer and Hunt (2006). Specimens are deposited in the invertebrate collection of the Department of Zoology, Swedish Museum of Natural History, Stockholm, Sweden (SMNH).

## Taxon treatments

### Campylaimus

Cobb, 1920

6EE5C5BB-86A9-537C-A160-CD946E1E8CDA

http://nemys.ugent.be/aphia.php?p=taxdetails&id=2437

Campylaimus Campylaimus inaequalis Cobb, 1920

#### Description

**Adult.** Body cylindrical or fusiform, tapering anteriorly in the pharyngeal region and posteriorly in the tail region, straight or curved, either ventrally or dorsally, upon fixation. Cuticle annulated along the entire body, annules equal in width; annulation starts at a level with the cephalic setae bases and extends towards the tail terminus; annules visually smooth or with fine longitudinal striation as seen under the light microscope. When present, longitudinal striation covers annules over the entire body length. Lateral alae present; anterior end of lateral alae may connect with the amphideal fovea in some species; posterior end extends towards tail terminus, ending at a certain distance from the tail tip. Body pores and epidermal glands absent. Somatic sensilla present. Labial region bluntly rounded, lips fused, tip of the anterior end often with much thicker cuticle. There are no cuticularised plates or other refractive structures around the anterior end. Inner labial sensilla invisible if present. Outer labial sensilla on the apical surface of the labial region. Of the outer labial sensilla, only lateral ones are often clearly visible under the light microscipe, located half way between the tip of the lip region and the anterior margin of the amphid. Cephalic sensilla setiform; their bases located at the base of the labial region, at various levels relative to the anterior edge of the amphid. Subcephalic and cervical sensilla, deirid and ocelli absent. Amphids usually similar in shape and size between sexes, sexual dimorphism, if present, is reflected in the relative length of the amphid or its sections: amphidial fovea an inverted U-shape with its ventral limb as long as or longer than the dorsal limb. Space between the amphidial limbs is variable in size (narrow or wide). Two species with the amphid in a shape of a longitudinal slit, assumed to be formed due to the reduction of the dorsal limb (Thanh and Gagarin 2011, Thanh et al. 2012). Amphidial glands often large, located at the level with the posterior part of the pharynx. Secretory-excretory system usually present; renette cell located opposite to the anterior part of intestine. Secretory-excretory ampulla present, located at the level of the intestine in the majority of species (if known). Cuticularised secretory-excretory duct very short, opens to the exterior via a pore, located at the level of the anterior part of intestine, except for *C. amphidialis*, in which it opens to the outside on the labial region. Nerve ring surrounding pharyngo-intestinal junction, just posterior to the muscular pharynx. Buccal cavity small and undifferentiated: cheilostom broad cylndrical, without rhabdia or sclerotisations; gymnostom and stegostom undifferentiated, closed or funnel-shaped, its slender lining continuous with that of the pharynx. Pharyngeal tubes absent. Pharynx uniformly muscular, cylindrical or fusiform, without visible subdivisions, with evenly distributed myofilaments; pharyngeal lumen uniform in thickness along the entire pharynx length; valves absent. Number and arrangement of pharyngeal glands and their orifices unclear. Cardia elongate, glandular. Tail conoid or subcylindrical, in some species with clavate terminal part. Three caudal glands present, their bodies and nuclei are incaudal; they open via a common spinneret.

**Female** reproductive system didelphic-amphidelphic with equally developed branches, ovaries outstretched. Spermatheca present in some species. Vulva equatorial, transverse (when described). Vagina straight; *pars proximalis vaginae* encircled by single sphincter muscle; *pars refringens vaginae* and epiptygmata absent. Tail most commonly with one pair of ventrosublateral setae located along the middle of the tail and one pair of sublateral setae located subterminally. Rectum short.

**Male** reproductive system diorchic, testes opposed: anterior testis outstretched and posterior testis reflexed. Spicules paired and symmetrical, arcuate, with weakly defined manubrium and subcylindrical shaft. Gubernaculum plate-like, without strongly developed apophysis. Some species with 2-5 precloacal supplements arranged midventrally; ventrosublateral precloacal and postcloacal sensilla absent. Tail most commonly with two pairs of ventrosublateral setae located along the posterior half of the tail and one pair of sublateral setae located subterminally.

#### Valid species

*Campylaimus abnormis* Nguyen Vu Thanh & Gagarin, 2011 (Thanh and Gagarin 2011)

*Campylaimus amphidialis* Fadeeva, Mordukhovich & Zograf, 2016 (Fadeeva et al. 2016)

*Campylaimus arquatus* Villares, Martelli, Lo Russo & Pastor, 2013 (Villares et al. 2013)

*Campylaimus bonariensis* Villares, Martelli, Lo Russo & Pastor, 2013 (Villares et al. 2013)

*Campylaimus cylindricus* Gerlach, 1956 (Gerlach 1956)

*Campylaimus gerlachi* Timm, 1961 (Gerlach 1950, Timm 1961)

*Campylaimus gracilis* Nguyen Vu Thanh, Nguyen Thanh Hien & Gagarin, 2012 (Thanh et al. 2012)

*Campylaimus inaequalis* Cobb, 1920 (Cobb 1920)

*Campylaimus lefeverei* Gerlach, 1956 (Gerlach 1956)

*Campylaimus longispiculus* sp. n.

*Campylaimus minor* Timm, 1961 (Timm 1961)

*Campylaimus minutus* Fadeeva, Mordukhovich & Zograf, 2016 (Fadeeva et al. 2016)

*Campylaimus mirus* Gerlach, 1950 (Gerlach 1956)

*Campylaimus orientalis* Fadeeva, Mordukhovich & Zograf, 2016 (Fadeeva et al. 2016)

*Campylaimus patagonicus* Villares, Martelli, Lo Russo & Pastor, 2013 (Villares et al. 2013)

*Campylaimus ponticus* Sergeeva, 1981 (Sergeeva 1981)

*Campylaimus pulcher* Fadeeva, Mordukhovich & Zograf, 2016 (Fadeeva et al. 2016)

*Campylaimus rimatus* Vitiello, 1974 (Vitiello 1974)

*Campylaimus siwaschensis* Sergeeva, 1981 (Sergeeva 1981)

*Campylaimus striatus* Boucher & Helléouët, 1977 (Boucher and Helléouët 1977)

*Campylaimus tkatchevi* Tchesunov, 1978 (Tchesunov 1978)

*Campylaimus triclados* sp. n.

#### Nomen nudum

*Campylaimus abditus* Bussau, 1993 (Bussau 1993)

### Campylaimus gerlachi

Timm, 1961

11916A1E-316B-523E-ACD7-A474B3EDDF6F

http://nemys.ugent.be/aphia.php?p=taxdetails&id=121400

#### Materials

- **Type status:** Other material. **Occurrence:** catalogNumber: SMNH-177084; individualCount: 3; sex: 2 females, 1 male; **Location:** waterBody: Baltic; country: Sweden; maximumDepthInMeters: 0.02; verbatimLatitude: 58°46,00'N; verbatimLongitude: 17°49,52'E; **Identification:** identifiedBy: O. Holovachov; dateIdentified: 2018; **Event:** year: 2011; month: 5; day: 26; habitat: Soft sediment
- **Type status:** Other material. **Occurrence:** catalogNumber: SMNH-177085; individualCount: 1; sex: female; **Location:** waterBody: Skagerrak; country: Sweden; locality: Gullmarsfjord, near Fiskebäckskil; minimumDepthInMeters: 8; maximumDepthInMeters: 15; verbatimLatitude: 58°15,09'N; verbatimLongitude: 11°27,54'E; **Identification:** identifiedBy: O. Holovachov; dateIdentified: 2018; **Event:** year: 2011; month: 8; day: 11; habitat: Muddy sand
- **Type status:** Other material. **Occurrence:** catalogNumber: SMNH-177086; individualCount: 2; sex: 1 female, 1 juvenile; **Location:** waterBody: Baltic; country: Sweden; locality: Öregrund; minimumDepthInMeters: 38; maximumDepthInMeters: 38; verbatimLatitude: 60°24,21'N; verbatimLongitude: 18°29,10'E; **Identification:** identifiedBy: O. Holovachov; dateIdentified: 2018; **Event:** year: 2011; month: 5; day: 19; habitat: Soft mud with clay
- **Type status:** Other material. **Occurrence:** catalogNumber: SMNH-177087; individualCount: 2; sex: female; **Location:** waterBody: Skagerrak; country: Sweden; locality: Bratten; minimumDepthInMeters: 45; maximumDepthInMeters: 55; verbatimLatitude: 58°17,32'N; verbatimLongitude: 11°11,24'E; **Identification:** identifiedBy: O. Holovachov; dateIdentified: 2018; **Event:** year: 2011; month: 8; day: 9; habitat: Coarse sediment with algae
- **Type status:** Other material. **Occurrence:** catalogNumber: SMNH-177088; individualCount: 2; sex: male; **Location:** waterBody: Skagerrak; country: Sweden; locality: Bratten; minimumDepthInMeters: 30; maximumDepthInMeters: 70; verbatimLatitude: 58°22,14'N; verbatimLongitude: 11°05,00'E; **Identification:** identifiedBy: O. Holovachov; dateIdentified: 2018; **Event:** year: 2011; month: 8; day: 9; habitat: Gravel, mud, algae
- **Type status:** Other material. **Occurrence:** catalogNumber: SMNH-177089; individualCount: 1; sex: female; **Location:** waterBody: Baltic; country: Sweden; maximumDepthInMeters: 0.02; verbatimLatitude: 58°46,00'N; verbatimLongitude: 17°49,52'E; **Identification:** identifiedBy: O. Holovachov; dateIdentified: 2018; **Event:** year: 2011; month: 5; day: 26; habitat: Soft sediment
- **Type status:** Other material. **Occurrence:** catalogNumber: SMNH-177090; individualCount: 1; sex: female; **Location:** waterBody: Skagerrak; country: Sweden; locality: Bratten; minimumDepthInMeters: 139; maximumDepthInMeters: 153; verbatimLatitude: 58°34,19'N; verbatimLongitude: 10°38,20'E; **Identification:** identifiedBy: O. Holovachov; dateIdentified: 2018; **Event:** year: 2012; month: 10; day: 12; habitat: Soft bottom
- **Type status:** Other material. **Occurrence:** catalogNumber: SMNH-177092; individualCount: 1; sex: female; **Location:** waterBody: Skagerrak; country: Sweden; locality: Gullmarsfjord, Kristineberg-Lysekil; minimumDepthInMeters: 53; maximumDepthInMeters: 53; verbatimLatitude: 58°15,73'N; verbatimLongitude: 11°26,10'E; **Identification:** identifiedBy: O. Holovachov; dateIdentified: 2018; **Event:** year: 2014; month: 8; day: 14; habitat: Mud
- **Type status:** Other material. **Occurrence:** catalogNumber: SMNH-177098; individualCount: 1; sex: female; **Location:** waterBody: Skagerrak; country: Sweden; locality: Bratten; minimumDepthInMeters: 139; maximumDepthInMeters: 153; verbatimLatitude: 58°34,19'N; verbatimLongitude: 10°38,20'E; **Identification:** identifiedBy: O. Holovachov; dateIdentified: 2018; **Event:** year: 2012; month: 10; day: 12; habitat: Soft bottom
- **Type status:** Other material. **Occurrence:** catalogNumber: SMNH-177109; individualCount: 1; sex: male; **Location:** waterBody: Skagerrak; country: Sweden; locality: Gullmarsfjord, near Fiskebäckskil; minimumDepthInMeters: 44; maximumDepthInMeters: 44; verbatimLatitude: 58°15,63'N; verbatimLongitude: 11°27,72'E; **Identification:** identifiedBy: O. Holovachov; dateIdentified: 2018; **Event:** year: 2011; month: 8; day: 11; habitat: Soft mud
- **Type status:** Other material. **Occurrence:** catalogNumber: SMNH-177112; individualCount: 1; sex: female; **Location:** waterBody: Skagerrak; country: Sweden; locality: Gullmarsfjord, near Fiskebäckskil; minimumDepthInMeters: 30; maximumDepthInMeters: 39; verbatimLatitude: 58°15,13'N; verbatimLongitude: 11°27,31'E; **Identification:** identifiedBy: O. Holovachov; dateIdentified: 2018; **Event:** year: 2010; month: 8; day: 21; habitat: Mud
- **Type status:** Other material. **Occurrence:** catalogNumber: SMNH-177121; individualCount: 1; sex: female; **Location:** waterBody: Baltic; country: Sweden; locality: Höga Kusten; minimumDepthInMeters: 86; maximumDepthInMeters: 86; verbatimLatitude: 62°49,10'N; verbatimLongitude: 18°23,44'E; **Identification:** identifiedBy: O. Holovachov; dateIdentified: 2018; **Event:** year: 2011; month: 5; day: 23; habitat: Silty mud on rather stiff clay
- **Type status:** Other material. **Occurrence:** catalogNumber: SMNH-177125; individualCount: 1; sex: female; **Location:** waterBody: Skagerrak; country: Sweden; locality: Bratten; minimumDepthInMeters: 139; maximumDepthInMeters: 153; verbatimLatitude: 58°34,19'N; verbatimLongitude: 10°38,20'E; **Identification:** identifiedBy: O. Holovachov; dateIdentified: 2018; **Event:** year: 2012; month: 10; day: 12; habitat: Soft bottom
- **Type status:** Other material. **Occurrence:** catalogNumber: SMNH TYPE-9205 (same slide as *C. triclados*); individualCount: 1; sex: female; **Location:** waterBody: Skagerrak; country: Sweden; locality: Gullmarsfjord, near Fiskebäckskil; minimumDepthInMeters: 30; maximumDepthInMeters: 30; verbatimLatitude: 58°15,25'N; verbatimLongitude: 11°27,30'E; **Identification:** identifiedBy: Oleksandr Holovachov; dateIdentified: 2018; **Event:** year: 2011; month: 8; day: 11; habitat: Soft mud
- **Type status:** Other material. **Occurrence:** catalogNumber: SMNH TYPE-9207 (same slide as *C. triclados*); individualCount: 1; sex: female; **Location:** waterBody: Skagerrak; country: Sweden; locality: Gullmarsfjord, near Fiskebäckskil; minimumDepthInMeters: 30; maximumDepthInMeters: 30; verbatimLatitude: 58°15,25'N; verbatimLongitude: 11°27,30'E; **Identification:** identifiedBy: Oleksandr Holovachov; dateIdentified: 2018; **Event:** year: 2011; month: 8; day: 11; habitat: Soft mud

#### Description

**Figure 1.**
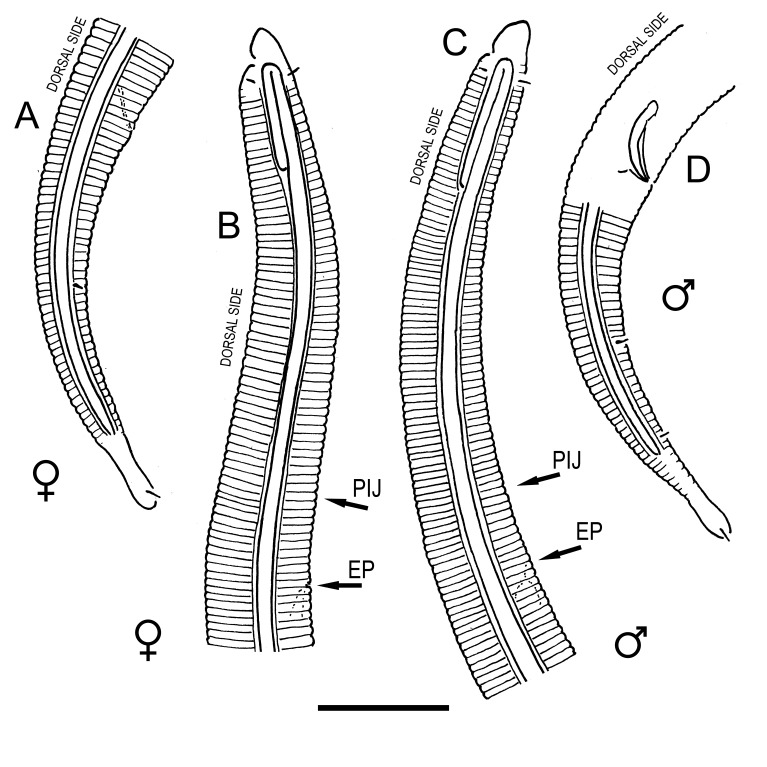
*Campylaimus gerlachi* Timm, 1961 (scale bars = 20 µm, PIJ = pharyngo-intestinal junction/cardia, EP = secretory-excretory pore): a: female tail; b: female pharyngeal region; c: male pharyngeal region; d: male caudal region.

**Measurements.** Table 1. **Adult. Figs 1, 2, 3.** Cuticle without longitudinal striation. Space between dorsal and ventral limbs of amphid not developed (Fig. 2a). Lateral alae narrow, appearing externally as two straight lines encompassing entire length of amphid. It originates at level with anterior edge of amphid, extends posteriorly as two lines parallel and very close to ventral limb of the amphid (Fig. 2b) and ends at level of posterior fifth of tail by merging with posterior end of ventral limb of amphid (Fig. 3c, d). Secretory-excretory pore opens posterior to cardia, at level with anterior part of intestine (Fig. 2d). Tail with clavate terminal part. **Male.** Anteriormost edge of amphid positioned just posterior to oral opening. Dorsal limb of amphid extends for a short distance posteriorly, equal to 2.2–2.7 labial region diameters in length. Ventral limb of amphid extends along entire body to posterior fifth of tail. Ventral limb of amphid is 1.0–1.5 wider than dorsal limb. Spicules with rounded manubrium and subcylindrical, arcuate shaft (Fig. 3a). Gubernaculum with dorsal apophysis (Fig. 3b). Precloacal supplements/sensilla invisible in current specimens. Two pairs of ventrosublateral setae located along posterior half of tail and one pair of sublateral setae located subterminally. **Female.** Anteriormost edge of amphid positioned just posterior to oral opening. Dorsal limb of amphid extends for a short distance posteriorly, equal to 2.1–2.3 labial region diameters in length. Ventral limb of amphid extends along entire body to posterior fifth of tail. Ventral limb of amphid is 1.5 wider than dorsal limb. Vagina straight (Fig. 2e, f). One pair of ventrosublateral setae located along middle of tail and one pair of sublateral setae located subterminally.

#### Diagnosis

Body 0.34–0.52 mm long; cuticle without longitudinal striation; anteriormost edge of amphid just posterior to oral opening; dorsal limb of amphid equal to 2.2–2.7 labial region diameters in male, 2.1–2.3 labial region diameters in female; ventral limb of amphid extends towards posterior fifth of tail in both female and male; ventral limb of amphid is 1.0–1.5 wider than dorsal limb; interamphideal space absent; secretory-excretory pore opens posterior to cardia; spicules 14–22 µm long; precloacal supplements indistinct; tail equal to 4.7–8.9 anal body diameters in length, with clavate terminal part.

#### Taxon discussion

Originally described as *C. inaequalis* by Gerlach (1950) and subsequently renamed into *C. gerlachi* by Timm (1961), the species was described from the Kiel Bay (Gerlach 1950), the Bay of Bengal (Timm 1961) and from the Yellow Sea (Huang and Zhang 2006), not accounting for published records from other parts of the world which were not accompanied by the proper species descriptions. Recent specimens and populations described by Gerlach (1950), Huang and Zhang (2006), Timm (1961) are united by a combination of the following characters: body less than 0.7 mm long, dorsal limb of the amphid equal to 2–3 labial region diameters in length, ventral limb of the amphid extends towards the posterior part of the tail, but does not reach the tail tip, both limbs of roughly equal width and the tail terminus clavate in shape. Villares et al. (2013) found a single male from Argentina with two precloacal supplements, which they also described under the name *C. gerlachi.* Unfortunately, since the description of this specimen does not cover all diagnostic features, it is impossible to verify the taxonomic position of the specimen and compare it to the population of *C. gerlachi* found in Sweden.

### Campylaimus minutus

Fadeeva, Mordukhovich & Zograf, 2016

817A171C-C006-5E70-95C8-180B9966A8E0

http://nemys.ugent.be/aphia.php?p=taxdetails&id=883820

#### Materials

- **Type status:** Other material. **Occurrence:** catalogNumber: SMNH-177090; individualCount: 4; sex: female; **Location:** waterBody: Skagerrak; country: Sweden; locality: Bratten; minimumDepthInMeters: 139; maximumDepthInMeters: 153; verbatimLatitude: 58°34,19'N; verbatimLongitude: 10°38,20'E; **Identification:** identifiedBy: O. Holovachov; dateIdentified: 2018; **Event:** year: 2012; month: 10; day: 12; habitat: Soft bottom
- **Type status:** Other material. **Occurrence:** catalogNumber: SMNH-177091; individualCount: 1; sex: female; **Location:** waterBody: Skagerrak; country: Sweden; locality: Bratten; minimumDepthInMeters: 139; maximumDepthInMeters: 153; verbatimLatitude: 58°34,19'N; verbatimLongitude: 10°38,20'E; **Identification:** identifiedBy: O. Holovachov; dateIdentified: 2018; **Event:** year: 2012; month: 10; day: 12; habitat: Soft bottom
- **Type status:** Other material. **Occurrence:** catalogNumber: SMNH-177092; individualCount: 1; sex: male; **Location:** waterBody: Skagerrak; country: Sweden; locality: Gullmarsfjord, Kristineberg-Lysekil; minimumDepthInMeters: 53; maximumDepthInMeters: 53; verbatimLatitude: 58°15,73'N; verbatimLongitude: 11°26,10'E; **Identification:** identifiedBy: O. Holovachov; dateIdentified: 2018; **Event:** year: 2014; month: 8; day: 14; habitat: Mud
- **Type status:** Other material. **Occurrence:** catalogNumber: SMNH-177093; individualCount: 1; sex: female; **Location:** waterBody: Skagerrak; country: Sweden; locality: Gullmarsfjord, Kristineberg-Lysekil; minimumDepthInMeters: 53; maximumDepthInMeters: 53; verbatimLatitude: 58°15,73'N; verbatimLongitude: 11°26,10'E; **Identification:** identifiedBy: O. Holovachov; dateIdentified: 2018; **Event:** year: 2014; month: 8; day: 14; habitat: Mud
- **Type status:** Other material. **Occurrence:** catalogNumber: SMNH-177124; individualCount: 3; sex: 2 females, 1 male; **Location:** waterBody: Skagerrak; country: Sweden; locality: Bratten; minimumDepthInMeters: 139; maximumDepthInMeters: 153; verbatimLatitude: 58°34,19'N; verbatimLongitude: 10°38,20'E; **Identification:** identifiedBy: O. Holovachov; dateIdentified: 2018; **Event:** year: 2012; month: 10; day: 12; habitat: Soft bottom
- **Type status:** Other material. **Occurrence:** catalogNumber: SMNH-177125; individualCount: 1; sex: female; **Location:** waterBody: Skagerrak; country: Sweden; locality: Bratten; minimumDepthInMeters: 139; maximumDepthInMeters: 153; verbatimLatitude: 58°34,19'N; verbatimLongitude: 10°38,20'E; **Identification:** identifiedBy: O. Holovachov; dateIdentified: 2018; **Event:** year: 2012; month: 10; day: 12; habitat: Soft bottom

#### Description

**Figure 4.**
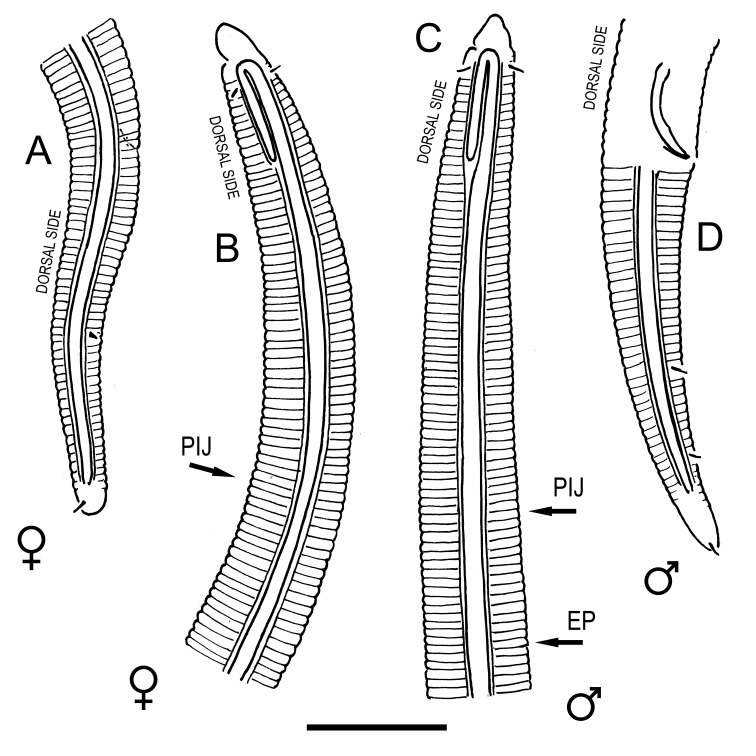
*Campylaimus minutus* Fadeeva, Mordukhovich & Zograf, 2016 (scale bars = 20 µm, PIJ = pharyngo-intestinal junction/cardia, EP = secretory-excretory pore): a: female tail; b: female pharyngeal region; c: male pharyngeal region; d: male caudal region.

**Measurements.** Table 1. **Adult. Figs 4, 5.** Cuticle without longitudinal striation. Space between dorsal and ventral limbs of amphid not developed. Lateral alae narrow, appearing externally as two straight lines encompassing entire length of amphid. It originates at level with anterior edge of amphid, extends posteriorly as two lines parallel and very close to ventral limb of amphid and ends close to tail tip by merging with posterior end of ventral limb of amphid (Fig. 5d). Secretory-excretory pore opens posterior to cardia, at level with anterior part of intestine. Tail with conoid terminal part. **Male.** Anteriormost edge of amphid positioned at level with oral opening. Dorsal limb of amphid extends for a short distance posteriorly, equal to 2.2 labial region diameters in length. Ventral limb of amphid extends along entire body to terminal part of tail. Ventral limb of the amphid is 1.0-1.5 wider than dorsal limb. Spicules with rounded manubrium and subcylindrical, arcuate shaft. Gubernaculum platelike, without apophysis. Precloacal supplements indistinct/absent. **Female.** Anteriormost edge of amphid positioned just posterior to or at level with oral opening. Dorsal limb of amphid extends for a short distance posteriorly, equal to 1.6-2.6 labial region diameters in length. Ventral limb of amphid extends along entire body to terminal part of tail. Ventral limb of amphid is 1.5-2.0 wider than dorsal limb. Vagina straight (Fig. 5c). One pair of ventrosublateral setae located along the middle of tail and one pair of sublateral setae located subterminally.

#### Diagnosis

Body 0.36–0.56 mm long; cuticle without longitudinal striation; anteriormost edge of amphid at level with oral opening; dorsal limb of amphid equal to 2.2 labial region diameters in male, 1.6–2.6 labial region diameters in female; ventral limb of amphid extends towards terminal part of tail in both female and male; ventral limb of amphid is 1.0–2.0 times wider than dorsal limb; interamphideal space absent; secretory-excretory pore opens posterior to cardia; spicules 16 µm long; precloacal supplements indistinct; tail equal to 4.1–6.0 anal body diameters in length, with conoid terminal part.

#### Taxon discussion

Recent specimens are nearly identical with the type populaiton of *C. minutus* from the Sea of Japan (Fadeeva et al. 2016) in qualitative and quantitative characters. *C. minutus* was originally distinguished from *C. gerlachi* in having relatively shorter cephalic setae (equal to 20% vs. 50% of the labial region width in length) and relatively shorter tail (<5 vs. >5 anal body diameters in length). As pointed below in the discussion, length of cephalic setae is often impossible to measure correctly, while the relative length of the tail in the *C. gerlachi* from Kiel Bay is exactly 5 (Gerlach 1950). Two species can however be distinguished from each other by the shape of the tail (clavate in *C. gerlachi* vs. conoid in *C. minutus*, see also Figure 5B in Fadeeva et al. (2016)) and in the position of the posterior end of the amphid (at a distance from the tail terminus in *C. gerlachi* vs. close to the tail terminus in *C. minutus*). Female specimen depicted on the Figures 5F-G in Fadeeva et al. (2016) could belong to *C. gerlachi* instead.

### Campylaimus tkatchevi

Tchesunov, 1978

D7256612-3FCE-54DD-8080-F6F085C012D0

http://nemys.ugent.be/aphia.php?p=taxdetails&id=878597

#### Materials

- **Type status:** Other material. **Occurrence:** catalogNumber: SMNH-177075; individualCount: 1; sex: female; **Location:** waterBody: Skagerrak; country: Sweden; locality: Gullmarsfjord, near Fiskebäckskil; minimumDepthInMeters: 40; maximumDepthInMeters: 50; verbatimLatitude: 58°15,40'N; verbatimLongitude: 11°27,46'E; **Identification:** identifiedBy: O. Holovachov; dateIdentified: 2018; **Event:** year: 2010; month: 8; day: 21; habitat: Mud
- **Type status:** Other material. **Occurrence:** catalogNumber: SMNH-177094; individualCount: 1; **Location:** waterBody: Skagerrak; country: Sweden; locality: Gullmarsfjord, Kristineberg-Lysekil; minimumDepthInMeters: 53; maximumDepthInMeters: 53; verbatimLatitude: 58°15,73'N; verbatimLongitude: 11°26,10'E; **Identification:** identifiedBy: O. Holovachov; dateIdentified: 2018; **Event:** year: 2014; month: 8; day: 14; habitat: Mud
- **Type status:** Other material. **Occurrence:** catalogNumber: SMNH-177096; individualCount: 1; sex: male; **Location:** waterBody: Skagerrak; country: Sweden; locality: Gullmarsfjord, near Fiskebäckskil; minimumDepthInMeters: 8; maximumDepthInMeters: 15; verbatimLatitude: 58°15,09'N; verbatimLongitude: 11°27,54'E; **Identification:** identifiedBy: O. Holovachov; dateIdentified: 2018; **Event:** year: 2011; month: 8; day: 11; habitat: Muddy sand
- **Type status:** Other material. **Occurrence:** catalogNumber: SMNH-177103; individualCount: 1; sex: female; **Location:** waterBody: Skagerrak; country: Sweden; locality: Bratten; minimumDepthInMeters: 352; maximumDepthInMeters: 374; verbatimLatitude: 58°19,18'N; verbatimLongitude: 10°29,34'E; **Identification:** identifiedBy: O. Holovachov; dateIdentified: 2018; **Event:** year: 2012; month: 10; day: 10; habitat: Soft bottom
- **Type status:** Other material. **Occurrence:** catalogNumber: SMNH-177113; individualCount: 2; sex: 1 female, 1 male; **Location:** waterBody: Skagerrak; country: Sweden; locality: Bratten; minimumDepthInMeters: 55; maximumDepthInMeters: 70; verbatimLatitude: 58°22,19'N; verbatimLongitude: 11°04,55'E; **Identification:** identifiedBy: O. Holovachov; dateIdentified: 2018; **Event:** year: 2011; month: 8; day: 9; habitat: Mud and clay
- **Type status:** Other material. **Occurrence:** catalogNumber: SMNH-177114; individualCount: 2; sex: female; **Location:** waterBody: Skagerrak; country: Sweden; locality: Gullmarsfjord, Kristineberg-Lysekil; minimumDepthInMeters: 53; maximumDepthInMeters: 53; verbatimLatitude: 58°15,73'N; verbatimLongitude: 11°26,10'E; **Identification:** identifiedBy: O. Holovachov; dateIdentified: 2018; **Event:** year: 2014; month: 8; day: 14; habitat: Mud
- **Type status:** Other material. **Occurrence:** catalogNumber: SMNH-177117; individualCount: 1; sex: male; **Location:** waterBody: Skagerrak; country: Sweden; locality: Gullmarsfjord, Klubban; verbatimLatitude: 58°15,03'N; verbatimLongitude: 11°27,54'E; **Identification:** identifiedBy: O. Holovachov; dateIdentified: 2018; **Event:** year: 2014; month: 8; day: 14; habitat: Shells, gravel, sand, mud
- **Type status:** Other material. **Occurrence:** catalogNumber: SMNH-177118; individualCount: 3; sex: 1 female, 2 juveniles; **Location:** waterBody: Skagerrak; country: Sweden; locality: Gullmarsfjord, near Fiskebäckskil; minimumDepthInMeters: 44; maximumDepthInMeters: 44; verbatimLatitude: 58°15,63'N; verbatimLongitude: 11°27,72'E; **Identification:** identifiedBy: O. Holovachov; dateIdentified: 2018; **Event:** year: 2011; month: 8; day: 11; habitat: Soft mud
- **Type status:** Other material. **Occurrence:** catalogNumber: SMNH-177119; individualCount: 3; sex: 1 female, 2 males; **Location:** waterBody: Skagerrak; country: Sweden; locality: Gullmarsfjord, Kristineberg-Lysekil; minimumDepthInMeters: 53; maximumDepthInMeters: 53; verbatimLatitude: 58°15,73'N; verbatimLongitude: 11°26,10'E; **Identification:** identifiedBy: O. Holovachov; dateIdentified: 2018; **Event:** year: 2014; month: 8; day: 14; habitat: Mud
- **Type status:** Other material. **Occurrence:** catalogNumber: SMNH-177120; individualCount: 1; sex: male; **Location:** waterBody: Skagerrak; country: Sweden; locality: Gullmarsfjord; minimumDepthInMeters: 54; maximumDepthInMeters: 54; verbatimLatitude: 58°16,00'N; verbatimLongitude: 11°28,00'E; **Identification:** identifiedBy: O. Holovachov; dateIdentified: 2018; **Event:** year: 1976; month: 4; day: 12; habitat: Very fine sediment

#### Description

**Figure 6.**
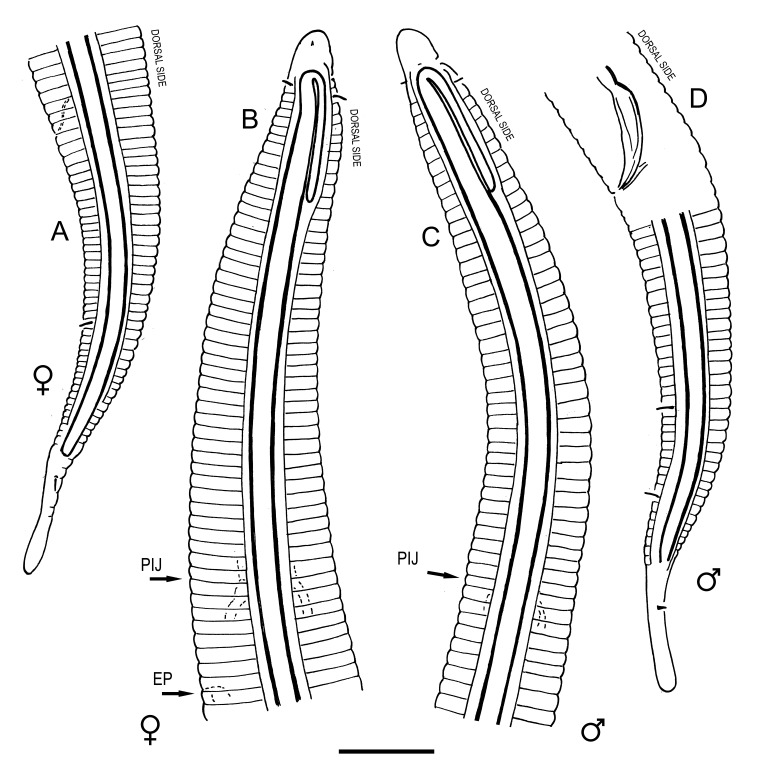
*Campylaimus tkatchevi* Tchesunov, 1978 (scale bars = 20 µm, PIJ = pharyngo-intestinal junction/cardia, EP = secretory-excretory pore): a: female tail; b: female pharyngeal region; c: male pharyngeal region; d: male caudal region.

**Measurements.** Table 2. **Adult. Figs 6, 7.** Cuticle without longitudinal striation. Space between dorsal and ventral limbs of amphid not developed. Lateral alae broad, appearing externally as two crenate lines encompassing entire length of amphid. It originates at level with anterior edge of amphid, extends posteriorly as two lines parallel to and widely spaced from ventral limb of the amphid (Fig. 7c, d) and ends at level of posterior fourth of tail by merging with posterior end of ventral limb of amphid. Secretory-excretory pore opens posterior to cardia, at level with anterior part of intestine (Fig. 7f). Tail with clavate terminal part. **Male.** Anteriormost edge of amphid positioned at level with oral opening. Dorsal limb of amphid extends for a short distance posteriorly, equal to 2.1–2.6 labial region diameters in length. Ventral limb of amphid extends along entire body to terminal part of tail. Ventral limb of amphid is 1.7–2.0 times wider than dorsal limb. Spicules with rounded manubrium and conoid, arcuate shaft. Gubernaculum platelike, without apophysis. Precloacal supplements absent/indistinct. Two pairs of ventrosublateral setae located along posterior half of tail and one pair of sublateral setae located between posterior end of lateral alae and tail terminus. **Female.** Anteriormost edge of amphid positioned at level with oral opening. Dorsal limb of amphid extends for a short distance posteriorly, equal to 1.8–2.4 labial region diameters in length. Ventral limb of amphid extends along entire body to terminal part of tail. Ventral limb of amphid is 1.7–2.5 times wider than dorsal limb. Vagina straight (Fig. 7e). One pair of ventrosublateral setae located along middle of tail and one pair of sublateral setae located close to posterior end of lateral alae.

#### Diagnosis

Body 0.56–0.73 mm long; cuticle without longitudinal striation; anteriormost edge of amphid at level with oral opening; dorsal limb of amphid equal to 2.1–2.6 labial region diameters in male, 1.8–2.4 labial region diameters in female; ventral limb of amphid extends towards posterior fourth of tail in both female and male; ventral limb of amphid is 1.7–2.5 wider than dorsal limb; interamphideal space absent; secretory-excretory pore opens posterior to cardia; spicules 27–30 µm long; precloacal supplements indistinct; tail equal to 4.8–5.9 anal body diameters in length, with clavate terminal part.

#### Taxon discussion

In having relatively long dorsal branch of the amphid, broad ventral branch of the amphid and clavate tail, recent specimens are most similar to *C. tkatchevi* described from the Caspian Sea (Tchesunov 1978), differing only in somewhat longer body (0.56–0.73 mm vs. 0.45–0.61 mm in the type population) and spicules (27–30 µm vs. 23 µm in the type population).

### Campylaimus siwaschensis

Sergeeva, 1981

2E5C169B-EED0-53AB-A7B5-F31100A813A2

http://nemys.ugent.be/aphia.php?p=taxdetails&id=230033

#### Materials

- **Type status:** Holotype. **Occurrence:** catalogNumber: SMNH-177089; individualCount: 1; sex: female; **Location:** waterBody: Baltic; country: Sweden; maximumDepthInMeters: 0.02; verbatimLatitude: 58°46,00'N; verbatimLongitude: 17°49,52'E; **Identification:** identifiedBy: O. Holovachov; dateIdentified: 2018; **Event:** year: 2011; month: 5; day: 26; habitat: Soft sediment

#### Description

**Figure 8.**
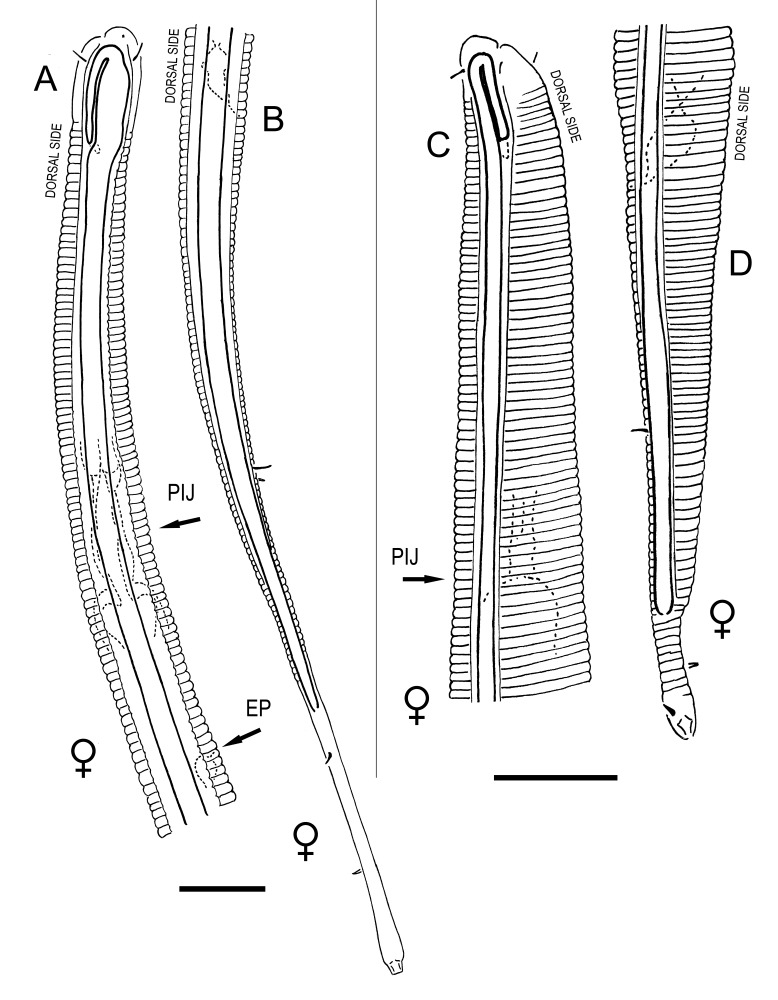
*Campylaimus siwaschensis* Sergeeva, 1981 (c-d) and *C. lefeverei* Gerlach, 1956 (a-b) (scale bars = 20 µm, PIJ = pharyngo-intestinal junction/cardia, EP = secretory-excretory pore): a, c: female pharyngeal region; b, d; female tail.

**Measurements.** Table 2. **Adult. Figs 8, 9.** Cuticle without longitudinal striation. Space between dorsal and ventral limbs of amphid not developed. Lateral alae narrow, appearing externally as two straight lines encompassing entire length of amphid. It originates at level with anterior edge of amphid, extends posteriorly as two lines parallel and very close to ventral limb of amphid and ends at level of posterior fifth of tail (Fig. 9c) by merging with posterior end of ventral limb of amphid. Secretory-excretory pore opens posterior to cardia, at level with anterior part of intestine. Tail with clavate terminal part. **Male.** Not found. **Female.** Anteriormost edge of amphid positioned just posterior to oral opening. Dorsal limb of amphid extends for a short distance posteriorly, equal to 1.2 labial region diameters in length. Ventral limb of amphid extends along entire body to terminal part of tail. Ventral limb of amphid is 1.3 times wider than dorsal limb. Vagina straight. One pair of ventrosublateral setae located along middle of tail and one pair of dorsosublateral setae located half way between posterior end of lateral alae and tal terminus.

#### Diagnosis

Body 0.6 mm long; cuticle without longitudinal striation; anteriormost edge of amphid posterior to oral opening; dorsal limb of amphid equal to 1.2 labial region diameters in female; ventral limb of amphid extends towards posterior fifth of tail; ventral limb of amphid is 1.3 wider than dorsal limb; interamphideal space absent; secretory-excretory pore opens posterior to cardia; tail equal to 5.8 anal body diameters in length, with clavate terminal part.

#### Taxon discussion

This species was originally described from the Lake Syvash, based on a single female (Sergeeva 1981). The original description has crucial disagreements between the text and the illustration of the species regarding the size of the dorsal amphideal limb: the text states that the dorsal limb of the amphid is 23.3 µm long, but the illustration shows it to be only around 12–13 µm long. Recent specimen collected in the Baltic is similar to the holotype female in most body measurements and specifically in relatively short dorsal amphideal limb (12–13 µm vs. 14 µm in recent specimen), if the illustration of the holotype is to be trusted.

### Campylaimus lefeverei

Gerlach, 1956

23F8C2EC-59B0-5DB7-BD8C-B2827C28E14C

http://nemys.ugent.be/aphia.php?p=taxdetails&id=121402

#### Materials

- **Type status:** Other material. **Occurrence:** catalogNumber: SMNH-177077; individualCount: 1; sex: female; **Location:** waterBody: Skagerrak; country: Sweden; locality: Gullmarsfjord, Kvarnbukten; minimumDepthInMeters: 5; maximumDepthInMeters: 5; **Identification:** identifiedBy: O. Holovachov; dateIdentified: 2018; **Event:** year: 2010; month: 8; day: 27

#### Description

**Measurements.** Table 2. **Adult. Figs 8, 10.** Cuticle without longitudinal striation. Space between dorsal and ventral limbs of amphid not developed. Lateral alae broad, appearing externally as two crenate lines encompassing entire length of the amphid. It originates at level with anterior edge of amphid, extends posteriorly as two lines parallel to and widely spaced from ventral limb of amphid (Fig. 10f) and ends at level of posterior fourth of tail by merging with posterior end of ventral limb of amphid. Interamphideal space absent. Cardia extremely long, more than corresponding body diameter in length. Secretory-excretory pore opens posterior to cardia, at level with anterior part of intestine (Fig. 10d). Tail with clavate terminal part. **Male.** Not found. **Female.** Anteriormost edge of amphid posterior to oral opening. Dorsal limb of amphid extends for a short distance posteriorly, equal to 1.6 labial region diameters in length. Ventral limb of amphid extends along entire body to posterior fourth of tail. Ventral limb of amphid is 3 times wider than dorsal limb. Vagina straight (Fig. 10e). Spermatheca absent, spermathozoa in uterus. One pair of ventrosublateral setae located along middle of tail and one pair of dorsosublateral setae located just behind posterior end of the lateral alae.

#### Diagnosis

Body 0.96 mm long; cuticle without longitudinal striation; anteriormost edge of amphid posterior to oral opening; dorsal limb of amphid equal to 1.6 labial region diameters in female; ventral limb of amphid extends towards posterior fourth of tail; ventral limb of amphid is 3 times wider than dorsal limb; interamphideal space absent; secretory-excretory pore opens posterior to cardia; tail equal to 14.5 anal body diameters in length, with clavate terminal part.

#### Taxon discussion

Original description of this species is based on two specimens, a male and a female (Gerlach 1956) while the recent specimen is a female. Both populations are similar in the morphology of the anterior end: position of the oral opening anterior to the anteriormost edge of the amphid and in the shape of the amphid without interamphideal space, with ventral limb of the amphid extending towards tail tip and the dorsal limb of the amphid 1.6–1.7 times the labial region diameter in length. Observed differences between the two populations include much smaller body of the recently collected female (959 µm vs. 1553–1555 µm in the original description) and thus smaller measurements of a number of characters (length of cephalic setae, length of dorsal limb of the amphid etc.). Since inter- and intraspecific variability in species of the genus *Campylaimus* are currently poorly understood and since no qualitative differences can be found between these two populations, they are considered here to belong to the same species.

### Campylaimus rimatus

Vitiello, 1974

285BBBF4-FDD7-5569-B6C6-CD8A2644B6F4

http://nemys.ugent.be/aphia.php?p=taxdetails&id=161218

#### Materials

- **Type status:** Other material. **Occurrence:** catalogNumber: SMNH-177072; individualCount: 1; sex: female; **Location:** waterBody: Skagerrak; country: Sweden; locality: Bratten; minimumDepthInMeters: 352; maximumDepthInMeters: 374; verbatimLatitude: 58°19,18'N; verbatimLongitude: 10°29,34'E; **Identification:** identifiedBy: O. Holovachov; dateIdentified: 2018; **Event:** year: 2012; month: 10; day: 10; habitat: Soft bottom
- **Type status:** Other material. **Occurrence:** catalogNumber: SMNH-177073; individualCount: 1; sex: male; **Location:** waterBody: Skagerrak; country: Sweden; locality: Bratten; minimumDepthInMeters: 232; maximumDepthInMeters: 240; verbatimLatitude: 58°27,40'N; verbatimLongitude: 10°33,56'E; **Identification:** identifiedBy: O. Holovachov; dateIdentified: 2018; **Event:** year: 2012; month: 10; day: 12; habitat: Soft bottom
- **Type status:** Other material. **Occurrence:** catalogNumber: SMNH-177074; individualCount: 2; sex: male; **Location:** waterBody: Skagerrak; country: Sweden; locality: Bratten; minimumDepthInMeters: 139; maximumDepthInMeters: 153; verbatimLatitude: 58°34,19'N; verbatimLongitude: 10°38,20'E; **Identification:** identifiedBy: O. Holovachov; dateIdentified: 2018; **Event:** year: 2012; month: 10; day: 12; habitat: Soft bottom
- **Type status:** Other material. **Occurrence:** catalogNumber: SMNH-177095; individualCount: 1; sex: male; **Location:** waterBody: Skagerrak; country: Sweden; locality: Bratten; minimumDepthInMeters: 390; maximumDepthInMeters: 428; verbatimLatitude: 58°22,30'N; verbatimLongitude: 10°20,33'E; **Identification:** identifiedBy: O. Holovachov; dateIdentified: 2018; **Event:** year: 2012; month: 10; day: 10; habitat: Soft bottom
- **Type status:** Other material. **Occurrence:** catalogNumber: SMNH-177097; individualCount: 1; sex: male; **Location:** waterBody: Skagerrak; country: Sweden; locality: Gullmarsfjord, near Fiskebäckskil; minimumDepthInMeters: 30; maximumDepthInMeters: 39; verbatimLatitude: 58°15,13'N; verbatimLongitude: 11°27,31'E; **Identification:** identifiedBy: O. Holovachov; dateIdentified: 2018; **Event:** year: 2010; month: 8; day: 21; habitat: Mud
- **Type status:** Other material. **Occurrence:** catalogNumber: SMNH-177102; individualCount: 2; sex: 1 female, 1 male; **Location:** waterBody: Skagerrak; country: Sweden; locality: Bratten; minimumDepthInMeters: 180; maximumDepthInMeters: 216; verbatimLatitude: 58°28,16'N; verbatimLongitude: 10°37,04'E; **Identification:** identifiedBy: O. Holovachov; dateIdentified: 2018; **Event:** year: 2012; month: 10; day: 11; habitat: Hard bottom
- **Type status:** Other material. **Occurrence:** catalogNumber: SMNH-177104; individualCount: 3; sex: female; **Location:** waterBody: Skagerrak; country: Sweden; locality: Bratten; minimumDepthInMeters: 232; maximumDepthInMeters: 240; verbatimLatitude: 58°27,40'N; verbatimLongitude: 10°33,56'E; **Identification:** identifiedBy: O. Holovachov; dateIdentified: 2018; **Event:** year: 2012; month: 10; day: 12; habitat: Soft bottom
- **Type status:** Other material. **Occurrence:** catalogNumber: SMNH-177105; individualCount: 1; sex: female; **Location:** waterBody: Skagerrak; country: Sweden; locality: Bratten; minimumDepthInMeters: 248; maximumDepthInMeters: 316; verbatimLatitude: 58°28,22'N; verbatimLongitude: 10°29,39'E; **Identification:** identifiedBy: O. Holovachov; dateIdentified: 2018; **Event:** year: 2012; month: 10; day: 11; habitat: Mixed bottom
- **Type status:** Other material. **Occurrence:** catalogNumber: SMNH-177106; individualCount: 2; sex: 1 female, 1 male; **Location:** waterBody: Skagerrak; country: Sweden; locality: Bratten; minimumDepthInMeters: 390; maximumDepthInMeters: 428; verbatimLatitude: 58°22,30'N; verbatimLongitude: 10°20,33'E; **Identification:** identifiedBy: O. Holovachov; dateIdentified: 2018; **Event:** year: 2012; month: 10; day: 10; habitat: Soft bottom
- **Type status:** Other material. **Occurrence:** catalogNumber: SMNH-177107; individualCount: 3; sex: 2 females, 1 male; **Location:** waterBody: Skagerrak; country: Sweden; locality: Bratten; minimumDepthInMeters: 351; maximumDepthInMeters: 387; verbatimLatitude: 58°22,19'N; verbatimLongitude: 10°23,50'E; **Identification:** identifiedBy: O. Holovachov; dateIdentified: 2018; **Event:** year: 2012; month: 10; day: 10; habitat: Soft bottom
- **Type status:** Other material. **Occurrence:** catalogNumber: SMNH-177123; individualCount: 2; sex: male; **Location:** waterBody: Skagerrak; country: Sweden; locality: Bratten; minimumDepthInMeters: 221; maximumDepthInMeters: 260; verbatimLatitude: 58°28,30'N; verbatimLongitude: 10°33,22'E; **Identification:** identifiedBy: O. Holovachov; dateIdentified: 2018; **Event:** year: 2012; month: 10; day: 11; habitat: Soft bottom
- **Type status:** Other material. **Occurrence:** catalogNumber: SMNH TYPE-9206 (same slide as *C. triclados*); individualCount: 1; sex: male; **Location:** waterBody: Skagerrak; country: Sweden; locality: Gullmarsfjord, near Fiskebäckskil; minimumDepthInMeters: 30; maximumDepthInMeters: 30; verbatimLatitude: 58°15,25'N; verbatimLongitude: 11°27,30'E; **Identification:** identifiedBy: Oleksandr Holovachov; dateIdentified: 2018; **Event:** year: 2011; month: 8; day: 11; habitat: Soft mud

#### Description

**Figure 11.**
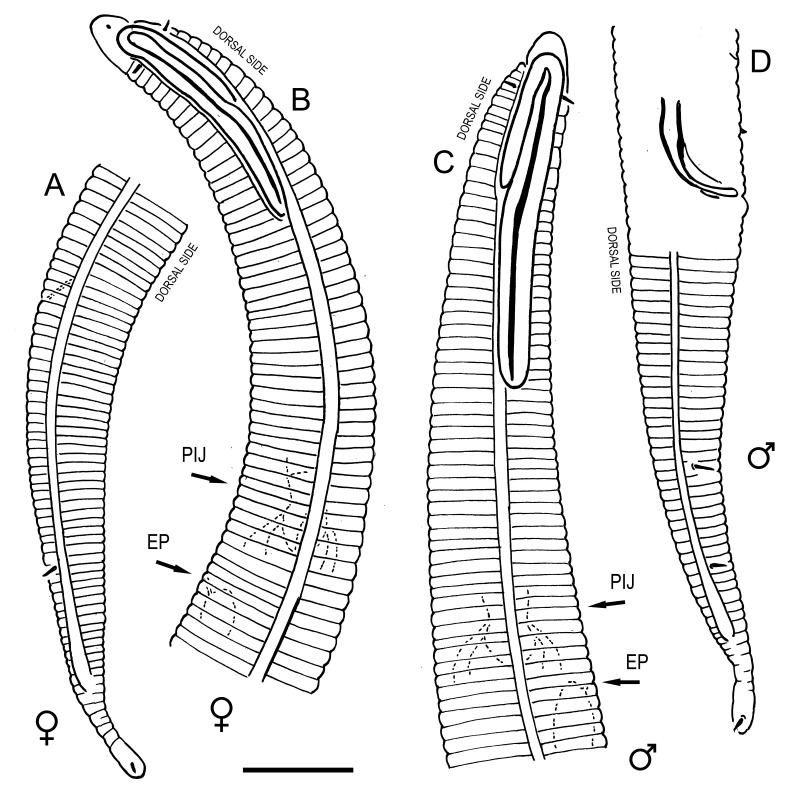
*Campylaimus rimatus* Vitiello, 1974 (scale bars = 20 µm, PIJ = pharyngo-intestinal junction/cardia, EP = secretory-excretory pore): a: female tail; b: female pharyngeal region; c: male pharyngeal region; d: male caudal region.

**Measurements.** Table 3. **Adult. Figs 11, 12, 13, 14**. Cuticle without longitudinal striation. Lateral pair of outer labial sensilla distinct in some specimens, located half way between tip of lip region and anterior margin of amphid (Fig. 12a). Space between dorsal and ventral limbs of amphid developed. Lateral alae narrow, appearing externally as smooth uniform band with straight margins (Fig. 13e), but with distinct subcuticular channel and crenate margins when focused midway (Fig. 13f). It originates at posterior end of dorsal limb of amphid and ends in a small expansion at basis of clavate part of tail. Secretory-excretory pore opens posterior to cardia, at level with anterior part of intestine (Fig. 13c, d). Tail with clavate terminal part. **Male.** Anteriormost edge of amphid positioned anterior to oral opening. Dorsal limb of amphid extends for a short distance posteriorly, equal to 2.2–2.6 labial region diameters in length. Ventral limb of amphid extends along pharyngeal region to level of midpharynx; 1.9–2.4 times the length of dorsal limb. Ventral limb of amphid is as wide as dorsal limb. Interamphideal space extends along pharyngeal region to same level as ventral limb. Spicules with rounded manubrium and conoid, arcuate shaft; distinct velum. Gubernaculum with weak caudal apophysis, often hard to discern. One small precloacal papilliform sensillum located on 5th–6th annule anterior to cloacal opening (Fig. 14a, b). Second precloacal pore-like sensillum located on 11th annule anterior to cloacal opening (Fig. 14a, b). Two pairs of ventrosublateral setae located along posterior half of tail and one pair of dorsosublateral setae located subterminally. **Female.** Anteriormost edge of amphid positioned anterior to oral opening. Dorsal limb of amphid extends for a short distance posteriorly, equal to 1.9–2.2 labial region diameters in length. Ventral limb of amphid extends along pharyngeal region to level of midpharynx; 1.6–1.9 times the length of dorsal limb. Ventral limb of amphid is as wide as dorsal limb. Interamphideal space extends along pharyngeal region to same level as ventral limb. Vagina straight. One pair of ventrosublateral setae located along posterior third of tail and one pair of dorsosublateral setae located subterminally.

#### Diagnosis

Body 0.63–0.73 mm long; cuticle without longitudinal striation; anteriormost edge of amphid anterior to oral opening; dorsal limb of amphid equal to 2.2–2.6 labial region diameters in male, 1.9–2.6 labial region diameters in female; ventral limb of amphid extends towards midpharynx in both sexes, 1.9-2.4 times the length of dorsal limb in male and 1.6-1.9 times the length of dorsal limb in female; ventral limb of amphid is as wide as dorsal limb; interamphideal space extends to same level as ventral limb; secretory-excretory pore opens posterior to cardia; spicules 23–26 µm long; two precloacal supplements; tail equal to 4.1–5.3 anal body diameters in length, with clavate terminal part.

#### Taxon discussion

Original description of this species is based on one male and two females (Vitiello 1974), while recent material includes 11 specimens. Both populations are similar in most body measurements and in the morphology of the anterior end: position of the oral opening posterior to the anteriormost edge of the amphid and in the shape of the amphid with developed interamphideal space, ventral limb of the amphid two times the length of the dorsal limb and extending to the level of the middle of the pharynx. Observed differences between two populations are limited to spicule length (31 µm in the holotype male and 23-26 µm in recent specimens).

### Campylaimus orientalis

Fadeeva, Mordukhovich & Zograf, 2016

8F7CF42F-F965-5755-8A19-006A8A91635F

http://nemys.ugent.be/aphia.php?p=taxdetails&id=883821

#### Materials

- **Type status:** Other material. **Occurrence:** catalogNumber: SMNH-177075; individualCount: 1; sex: female; **Location:** waterBody: Skagerrak; country: Sweden; locality: Gullmarsfjord, near Fiskebäckskil; minimumDepthInMeters: 40; maximumDepthInMeters: 50; verbatimLatitude: 58°15,40'N; verbatimLongitude: 11°27,46'E; **Identification:** identifiedBy: O. Holovachov; dateIdentified: 2018; **Event:** year: 2010; month: 8; day: 21; habitat: Mud
- **Type status:** Other material. **Occurrence:** catalogNumber: SMNH-177078; individualCount: 2; sex: female; **Location:** waterBody: Skagerrak; country: Sweden; locality: Bratten; minimumDepthInMeters: 25; maximumDepthInMeters: 50; verbatimLatitude: 58°22,20'N; verbatimLongitude: 11°09,26'E; **Identification:** identifiedBy: O. Holovachov; dateIdentified: 2018; **Event:** year: 2011; month: 8; day: 9; habitat: Muddy sand
- **Type status:** Other material. **Occurrence:** catalogNumber: SMNH-177079; individualCount: 1; sex: female; **Location:** waterBody: Skagerrak; country: Sweden; locality: Gullmarsfjord, Kristineberg-Lysekil; minimumDepthInMeters: 53; maximumDepthInMeters: 53; verbatimLatitude: 58°15,73'N; verbatimLongitude: 11°26,10'E; **Identification:** identifiedBy: O. Holovachov; dateIdentified: 2018; **Event:** year: 2014; month: 8; day: 14; habitat: Mud
- **Type status:** Other material. **Occurrence:** catalogNumber: SMNH-177080; individualCount: 3; sex: 1 female, 2 males; **Location:** waterBody: Skagerrak; country: Sweden; locality: Gullmarsfjord, near Fiskebäckskil; minimumDepthInMeters: 20; maximumDepthInMeters: 30; verbatimLatitude: 58°16,04'N; verbatimLongitude: 11°27,34'E; **Identification:** identifiedBy: O. Holovachov; dateIdentified: 2018; **Event:** year: 2010; month: 8; day: 21; habitat: Mud
- **Type status:** Other material. **Occurrence:** catalogNumber: SMNH-177081; individualCount: 2; sex: 1 female, 1 male; **Location:** waterBody: Skagerrak; country: Sweden; locality: Gullmarsfjord, Kristineberg-Lysekil; minimumDepthInMeters: 53; maximumDepthInMeters: 53; verbatimLatitude: 58°15,73'N; verbatimLongitude: 11°26,10'E; **Identification:** identifiedBy: O. Holovachov; dateIdentified: 2018; **Event:** year: 2014; month: 8; day: 14; habitat: Mud
- **Type status:** Other material. **Occurrence:** catalogNumber: SMNH-177082; individualCount: 1; sex: female; **Location:** waterBody: Skagerrak; country: Sweden; locality: Gullmarsfjord, near Fiskebäckskil; minimumDepthInMeters: 40; maximumDepthInMeters: 50; verbatimLatitude: 58°15,40'N; verbatimLongitude: 11°27,46'E; **Identification:** identifiedBy: O. Holovachov; dateIdentified: 2018; **Event:** year: 2010; month: 8; day: 21; habitat: Mud
- **Type status:** Other material. **Occurrence:** catalogNumber: SMNH-177083; individualCount: 1; sex: female; **Location:** waterBody: Skagerrak; country: Sweden; locality: Bratten; minimumDepthInMeters: 139; maximumDepthInMeters: 153; verbatimLatitude: 58°34,19'N; verbatimLongitude: 10°38,20'E; **Identification:** identifiedBy: O. Holovachov; dateIdentified: 2018; **Event:** year: 2012; month: 10; day: 12; habitat: Soft bottom
- **Type status:** Other material. **Occurrence:** catalogNumber: SMNH-177093; individualCount: 4; sex: 3 females, 1 male; **Location:** waterBody: Skagerrak; country: Sweden; locality: Gullmarsfjord, Kristineberg-Lysekil; minimumDepthInMeters: 53; maximumDepthInMeters: 53; verbatimLatitude: 58°15,73'N; verbatimLongitude: 11°26,10'E; **Identification:** identifiedBy: Oleksandr Holovachov; dateIdentified: 2018; **Event:** year: 2014; month: 8; day: 14; habitat: Mud
- **Type status:** Other material. **Occurrence:** catalogNumber: SMNH-177097; individualCount: 7; sex: 2 females, 5 males; **Location:** waterBody: Skagerrak; country: Sweden; locality: Gullmarsfjord, near Fiskebäckskil; minimumDepthInMeters: 30; maximumDepthInMeters: 39; verbatimLatitude: 58°15,13'N; verbatimLongitude: 11°27,31'E; **Identification:** identifiedBy: O. Holovachov; dateIdentified: 2018; **Event:** year: 2010; month: 8; day: 21; habitat: Mud
- **Type status:** Other material. **Occurrence:** catalogNumber: SMNH-177099; individualCount: 1; sex: female; **Location:** waterBody: Skagerrak; country: Sweden; locality: Gullmarsfjord, near Fiskebäckskil; minimumDepthInMeters: 8; maximumDepthInMeters: 15; verbatimLatitude: 58°15,09'N; verbatimLongitude: 11°27,54'E; **Identification:** identifiedBy: O. Holovachov; dateIdentified: 2018; **Event:** year: 2011; month: 8; day: 11; habitat: Muddy sand
- **Type status:** Other material. **Occurrence:** catalogNumber: SMNH-177100; individualCount: 2; sex: 1 female, 1 male; **Location:** waterBody: Skagerrak; country: Sweden; locality: Bratten; minimumDepthInMeters: 53; maximumDepthInMeters: 53; verbatimLatitude: 58°20,06'N; verbatimLongitude: 11°09,24'E; **Identification:** identifiedBy: O. Holovachov; dateIdentified: 2018; **Event:** year: 2011; month: 8; day: 9
- **Type status:** Other material. **Occurrence:** catalogNumber: SMNH-177101; individualCount: 1; sex: female; **Location:** waterBody: Skagerrak; country: Sweden; locality: Bratten; minimumDepthInMeters: 25; maximumDepthInMeters: 50; verbatimLatitude: 58°22,20'N; verbatimLongitude: 11°09,26'E; **Identification:** identifiedBy: O. Holovachov; dateIdentified: 2018; **Event:** year: 2011; month: 8; day: 9; habitat: Muddy sand
- **Type status:** Other material. **Occurrence:** catalogNumber: SMNH-177114; individualCount: 3; sex: 1 female, 2 males; **Location:** waterBody: Skagerrak; country: Sweden; locality: Gullmarsfjord, Kristineberg-Lysekil; minimumDepthInMeters: 53; maximumDepthInMeters: 53; verbatimLatitude: 58°15,73'N; verbatimLongitude: 11°26,10'E; **Identification:** identifiedBy: O. Holovachov; dateIdentified: 2018; **Event:** year: 2014; month: 8; day: 14; habitat: Mud
- **Type status:** Other material. **Occurrence:** catalogNumber: SMNH-177117; individualCount: 1; sex: female; **Location:** waterBody: Skagerrak; country: Sweden; locality: Gullmarsfjord, Klubban; verbatimLatitude: 58°15,03'N; verbatimLongitude: 11°27,54'E; **Identification:** identifiedBy: O. Holovachov; dateIdentified: 2018; **Event:** year: 2014; month: 8; day: 14; habitat: Shells, gravel, sand, mud
- **Type status:** Other material. **Occurrence:** catalogNumber: SMNH-177118; individualCount: 2; sex: 1 female, 1 male; **Location:** waterBody: Skagerrak; country: Sweden; locality: Gullmarsfjord, near Fiskebäckskil; minimumDepthInMeters: 44; maximumDepthInMeters: 44; verbatimLatitude: 58°15,63'N; verbatimLongitude: 11°27,72'E; **Identification:** identifiedBy: O. Holovachov; dateIdentified: 2018; **Event:** year: 2011; month: 8; day: 11; habitat: Soft mud
- **Type status:** Other material. **Occurrence:** catalogNumber: SMNH-177119; individualCount: 2; sex: 1 female, 1 male; **Location:** waterBody: Skagerrak; country: Sweden; locality: Gullmarsfjord, Kristineberg-Lysekil; minimumDepthInMeters: 53; maximumDepthInMeters: 53; verbatimLatitude: 58°15,73'N; verbatimLongitude: 11°26,10'E; **Identification:** identifiedBy: O. Holovachov; dateIdentified: 2018; **Event:** year: 2014; month: 8; day: 14; habitat: Mud
- **Type status:** Other material. **Occurrence:** catalogNumber: SMNH-177120; individualCount: 1; sex: female; **Location:** waterBody: Skagerrak; country: Sweden; locality: Gullmarsfjord; minimumDepthInMeters: 54; maximumDepthInMeters: 54; verbatimLatitude: 58°16,00'N; verbatimLongitude: 11°28,00'E; **Identification:** identifiedBy: O. Holovachov; dateIdentified: 2018; **Event:** year: 1976; month: 4; day: 12; habitat: Very fine sediment
- **Type status:** Other material. **Occurrence:** catalogNumber: SMNH-177122; individualCount: 1; sex: male; **Location:** waterBody: Skagerrak; country: Sweden; locality: Gullmarsfjord, near Fiskebäckskil; minimumDepthInMeters: 30; maximumDepthInMeters: 30; verbatimLatitude: 58°15,25'N; verbatimLongitude: 11°27,30'E; **Identification:** identifiedBy: O. Holovachov; dateIdentified: 2018; **Event:** year: 2011; month: 8; day: 11; habitat: Soft mud
- **Type status:** Other material. **Occurrence:** catalogNumber: SMNH TYPE-9205 (same slide as *C. triclados*); individualCount: 2; sex: female; **Location:** waterBody: Skagerrak; country: Sweden; locality: Gullmarsfjord, near Fiskebäckskil; minimumDepthInMeters: 30; maximumDepthInMeters: 30; verbatimLatitude: 58°15,25'N; verbatimLongitude: 11°27,30'E; **Identification:** identifiedBy: Oleksandr Holovachov; dateIdentified: 2018; **Event:** year: 2011; month: 8; day: 11; habitat: Soft mud
- **Type status:** Other material. **Occurrence:** catalogNumber: SMNH TYPE-9206 (same slide as *C. triclados*); individualCount: 1; sex: male; **Location:** waterBody: Skagerrak; country: Sweden; locality: Gullmarsfjord, near Fiskebäckskil; minimumDepthInMeters: 30; maximumDepthInMeters: 30; verbatimLatitude: 58°15,25'N; verbatimLongitude: 11°27,30'E; **Identification:** identifiedBy: Oleksandr Holovachov; dateIdentified: 2018; **Event:** year: 2011; month: 8; day: 11; habitat: Soft mud

#### Description

**Figure 15.**
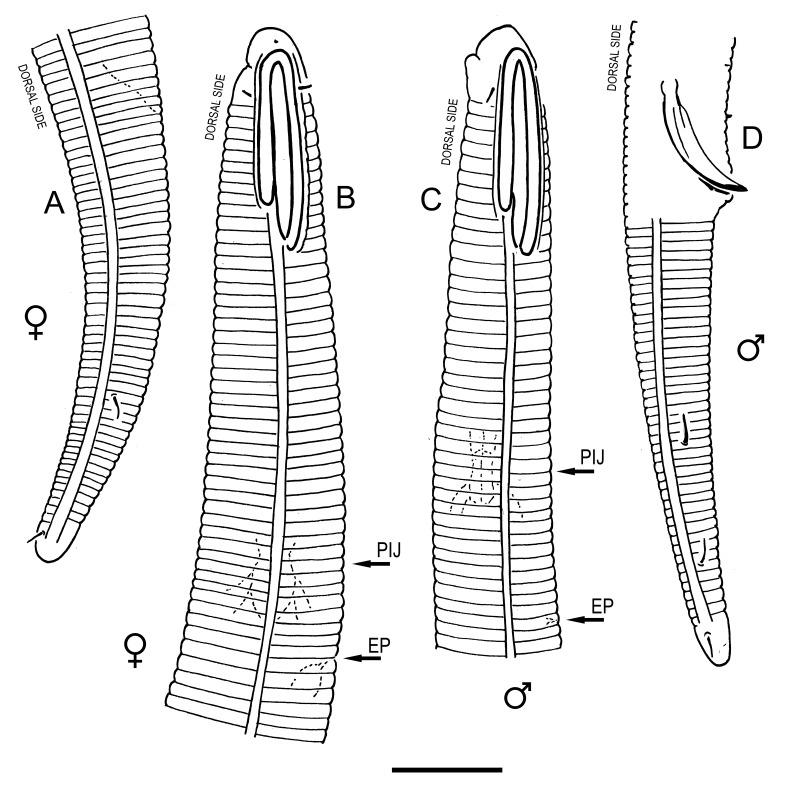
*Campylaimus orientalis* Fadeeva, Mordukhovich & Zograf, 2016 (scale bars = 20 µm, PIJ = pharyngo-intestinal junction/cardia, EP = secretory-excretory pore): a: female tail; b: female pharyngeal region; c: male pharyngeal region; d: male caudal region.

**Measurements.** Table 3. **Adult. Figs 15, 16, 17**. Cuticle without longitudinal striation. Lateral pair of outer labial sensilla distinct in some specimens, located half way between tip of lip region and anterior margin of amphid. Space between dorsal and ventral limbs of amphid developed. Lateral alae narrow, appearing externally as smooth uniform band with straight margins. It originates at posterior end of dorsal limb of amphid and ends in a small expansion at basis of tail tip (Fig. 17f). Secretory-excretory pore opens posterior to cardia, at level with anterior part of intestine. Tail with conoid, gradually narrowing terminal part. **Male.** Anteriormost edge of amphid positioned anterior to oral opening. Dorsal limb of amphid extends for a short distance posteriorly, equal to 1.9–2.3 labial region diameters in length. Ventral limb of amphid extends along pharyngeal region to level of anterior third of pharynx; 1.2–1.4 times the length of dorsal limb. Ventral limb of amphid is as wide as dorsal limb. Interamphideal space extends along pharyngeal region to same level as ventral limb. Spicules with rounded manubrium and conoid, arcuate shaft. Gubernaculum plate-like, without apophysis. One small precloacal papilliform sensillum located on 6th annule anterior to cloacal opening (Fig. 17c, d). Second precloacal pore-like sensillum located on 10th–11th annule anterior to cloacal opening (Fig. 17c, d). Two pairs of ventrosublateral setae located along posterior half of tail and one pair of dorsosublateral setae located subterminally. **Female.** Anteriormost edge of amphid positioned anterior to oral opening. Dorsal limb of amphid extends for a short distance posteriorly, equal to 1.6–2.3 labial region diameters in length. Ventral limb of amphid extends along pharyngeal region to level of anterior third of pharynx; 1.2–1.4 times the length of dorsal limb. Ventral limb of amphid is as wide as dorsal limb. Interamphideal space extends along pharyngeal region to same level as ventral limb. Vagina straight. Vulva a transverse slit. One pair of ventrosublateral setae located along posterior third of tail and one pair of dorsosublateral setae located subterminally.

#### Diagnosis

Body 0.58–0.65 mm long; cuticle without longitudinal striation; anteriormost edge of amphid anterior to oral opening; dorsal limb of amphid equal to 1.9–2.3 labial region diameters in male, 1.6–2.3 labial region diameters in female; ventral limb of amphid extends towards anterior third of pharynx in both sexes, 1.2–1.4 times the length of dorsal limb in both sexes; ventral limb of amphid is as wide as dorsal limb; interamphideal space extends to same level as ventral limb; secretory-excretory pore opens posterior to cardia; spicules 23–27 µm long; two precloacal supplements; tail equal to 3.9–5.3 anal body diameters in length, with conoid terminal part.

#### Taxon discussion

The main distinguishing feature of *C. orientalis* is the shape of the amphid, where the ventral limb is only slightly longer than the dorsal limb and the inter-amphideal space is equal in width to either of the limbs of the amphid (Fadeeva et al. 2016). This feature unites both type population from the Sea of Japan and recently collected specimens from the Skagerrak, with recent specimens only being somewhat smaller in size (0.58–0.65 mm vs. 0.61–0.85 mm in type specimens), which also proportionally affects a number of other characters.

### Campylaimus triclados

Holovachov, 2019 sp. n.

49E268BD-CC27-5160-8A78-63C74B5EDE40

urn:lsid:zoobank.org:act:E9BF6AF6-E7CF-4EB5-AA10-0F308AF8616E

#### Materials

- **Type status:** Holotype. **Occurrence:** catalogNumber: SMNH TYPE-9205; individualCount: 1; sex: male; **Location:** waterBody: Skagerrak; country: Sweden; locality: Gullmarsfjord, near Fiskebäckskil; minimumDepthInMeters: 30; maximumDepthInMeters: 30; verbatimLatitude: 58°15,25'N; verbatimLongitude: 11°27,30'E; **Identification:** identifiedBy: Oleksandr Holovachov; dateIdentified: 2018; **Event:** year: 2011; month: 8; day: 11; habitat: Soft mud
- **Type status:** Paratype. **Occurrence:** catalogNumber: SMNH TYPE-9207; individualCount: 1; sex: female; **Location:** waterBody: Skagerrak; country: Sweden; locality: Gullmarsfjord, near Fiskebäckskil; minimumDepthInMeters: 30; maximumDepthInMeters: 30; verbatimLatitude: 58°15,25'N; verbatimLongitude: 11°27,30'E; **Identification:** identifiedBy: Oleksandr Holovachov; dateIdentified: 2018; **Event:** year: 2011; month: 8; day: 11; habitat: Soft mud
- **Type status:** Paratype. **Occurrence:** catalogNumber: SMNH TYPE-9206; individualCount: 2; sex: 1 female, 1 male; **Location:** waterBody: Skagerrak; country: Sweden; locality: Gullmarsfjord, near Fiskebäckskil; minimumDepthInMeters: 30; maximumDepthInMeters: 30; verbatimLatitude: 58°15,25'N; verbatimLongitude: 11°27,30'E; **Identification:** identifiedBy: Oleksandr Holovachov; dateIdentified: 2018; **Event:** year: 2011; month: 8; day: 11; habitat: Soft mud
- **Type status:** Other material. **Occurrence:** catalogNumber: SMNH-177105; individualCount: 1; sex: male; **Location:** waterBody: Skagerrak; country: Sweden; locality: Bratten; minimumDepthInMeters: 248; maximumDepthInMeters: 316; verbatimLatitude: 58°28,22'N; verbatimLongitude: 10°29,39'E; **Identification:** identifiedBy: O. Holovachov; dateIdentified: 2018; **Event:** year: 2012; month: 10; day: 11; habitat: Mixed bottom
- **Type status:** Other material. **Occurrence:** catalogNumber: SMNH-177106; individualCount: 2; sex: 1 female, 1 juvenile; **Location:** waterBody: Skagerrak; country: Sweden; locality: Bratten; minimumDepthInMeters: 390; maximumDepthInMeters: 428; verbatimLatitude: 58°22,30'N; verbatimLongitude: 10°20,33'E; **Identification:** identifiedBy: O. Holovachov; dateIdentified: 2018; **Event:** year: 2012; month: 10; day: 10; habitat: Soft bottom
- **Type status:** Other material. **Occurrence:** catalogNumber: SMNH-177107; individualCount: 1; sex: male; **Location:** waterBody: Skagerrak; country: Sweden; locality: Bratten; minimumDepthInMeters: 351; maximumDepthInMeters: 387; verbatimLatitude: 58°22,19'N; verbatimLongitude: 10°23,50'E; **Identification:** identifiedBy: O. Holovachov; dateIdentified: 2018; **Event:** year: 2012; month: 10; day: 10; habitat: Soft bottom
- **Type status:** Other material. **Occurrence:** catalogNumber: SMNH-177118; individualCount: 2; sex: 1 female, 1 male; **Location:** waterBody: Skagerrak; country: Sweden; locality: Gullmarsfjord, near Fiskebäckskil; minimumDepthInMeters: 44; maximumDepthInMeters: 44; verbatimLatitude: 58°15,63'N; verbatimLongitude: 11°27,72'E; **Identification:** identifiedBy: O. Holovachov; dateIdentified: 2018; **Event:** year: 2011; month: 8; day: 11; habitat: Soft mud
- **Type status:** Other material. **Occurrence:** catalogNumber: SMNH-177122; individualCount: 1; sex: male; **Location:** waterBody: Skagerrak; country: Sweden; locality: Gullmarsfjord, near Fiskebäckskil; minimumDepthInMeters: 30; maximumDepthInMeters: 30; verbatimLatitude: 58°15,25'N; verbatimLongitude: 11°27,30'E; **Identification:** identifiedBy: O. Holovachov; dateIdentified: 2018; **Event:** year: 2011; month: 8; day: 11; habitat: Soft mud

#### Description

**Figure 18.**
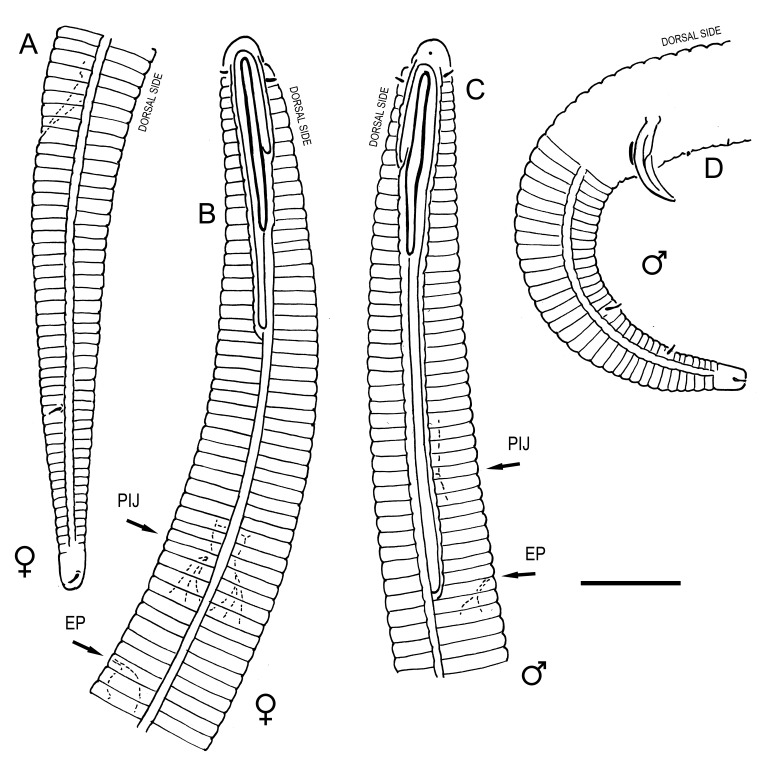
*Campylaimus triclados* sp. n. (scale bars = 20 µm, PIJ = pharyngo-intestinal junction/cardia, EP = secretory-excretory pore): a: female tail; b: female pharyngeal region; c: male pharyngeal region; d: male caudal region.

**Measurements.** Table 4. **Adult. Figs 18, 19, 20, 21.** Cuticle without longitudinal striation. Space between dorsal and ventral limbs of amphid developed. Lateral alae narrow, appearing externally as smooth uniform band with straight margins, but with crenate margins when focused midway. It originates at posterior end of dorsal limb of amphid and gradually disappears at a short distance from the tail tip. Secretory-excretory pore opens posterior to cardia, at level with anterior part of intestine (Fig. 20c, d). Tail with conoid terminal part. **Male.** Anteriormost edge of amphid positioned anterior to oral opening. Dorsal limb of amphid extends for a short distance posteriorly, equal to 1.6–2.5 labial region diameters in length. Ventral limb of amphid extends along pharyngeal region to level of anterior part of intestine; 4.3–5.5 times the length of dorsal limb. Ventral limb of amphid is as wide as dorsal limb. Interamphideal space extends along pharyngeal region further than posterior end of dorsal limb but not reaching posterior end of ventral limb. Spicules with rounded manubrium and conoid, arcuate shaft; distinct velum. Gubernaculum platelike, without apophysis. One small precloacal papilliform sensillum located on 5th-6th annule anterior to cloacal opening (Fig. 21a). Second precloacal pore-like sensillum located on 9th annule anterior to cloacal opening (Fig. 21a). Two pairs of ventrosublateral setae located along posterior half of tail and one pair of dorsosublateral setae located subterminally. **Female.** Anteriormost edge of amphid positioned anterior to oral opening. Dorsal limb of amphid extends for a short distance posteriorly, equal to 1.9 labial region diameters in length. Ventral limb of amphid extends along pharyngeal region to level of midpharynx; 2.2–2.8 times the length of dorsal limb. Ventral limb of amphid is as wide as dorsal limb. Interamphideal space extends along pharyngeal region further than posterior end of the dorsal limb but not reaching posterior end of ventral limb. Vagina straight. One pair of ventrosublateral setae located along posterior third of tail and one pair of dorsosublateral setae located subterminally.

#### Diagnosis

Body 0.59–0.69 mm long; cuticle without longitudinal striation; anteriormost edge of amphid anterior to oral opening; dorsal limb of amphid equal to 1.6–2.5 labial region diameters in male, 1.9 labial region diameters in female; ventral limb of amphid extends towards anterior part of intestine in male and midpharynx in female, 4.3–5.5 times the length of dorsal limb in male and 2.2–2.8 times the length of dorsal limb in female; ventral limb of amphid is as wide as the dorsal limb; interamphideal space extends further than posterior end of dorsal limb but not reaching posterior end of ventral limb; secretory-excretory pore opens posterior to cardia; spicules 19–24 µm long; two precloacal supplements; tail equal to 4.3–5.6 anal body diameters in length, with conoid terminal part.

#### Etymology

The specific epithet refers to the three-partite shape of the amphid, with three "branches": strongly developed dorsal and ventral limbs and prominent interamphideal space.

#### Taxon discussion

This new species can be easily differentiated from all currently known species of the genus *Campylaimus* by the shape of the amphid, with interamphideal space being longer than the dorsal limb of the amphid, but shorter than the ventral limb of the amphid. In all other known species with developed interamphideal space (*C. rimatus, C. mirus* and *C. orientalis*), it is usually as long as the ventral limb of the amphid.

### Campylaimus mirus

Gerlach, 1950

96257CB6-2C43-55D4-AF50-A7215B01746F

http://nemys.ugent.be/aphia.php?p=taxdetails&id=121403

#### Materials

- **Type status:** Other material. **Occurrence:** catalogNumber: SMNH-177076; individualCount: 1; sex: female; **Location:** country: Germany; locality: Helgoland; georeferenceProtocol: label; **Identification:** identifiedBy: Oleksandr Holovachov; dateIdentified: 2018; **Event:** year: 2011; month: 5; day: 31; habitat: Sediment

#### Description

**Figure 22.**
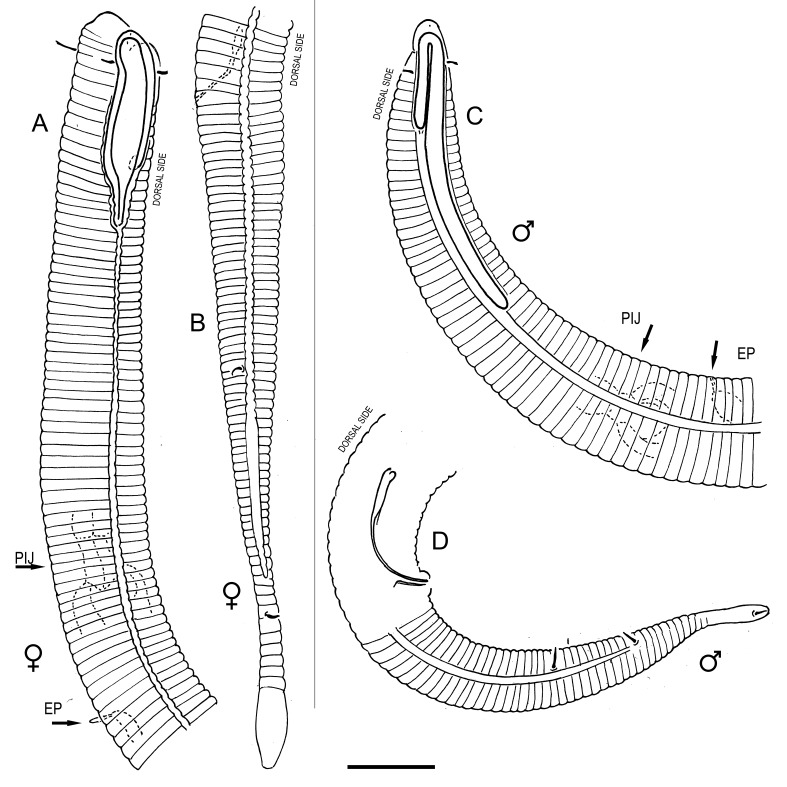
*Campylaimus mirus* Gerlach, 1950 (a-b) and *Campylaimus longispiculus* sp. n. (c-d) (scale bars = 20 µm, PIJ = pharyngo-intestinal junction/cardia, EP = secretory-excretory pore): a: female pharyngeal region; b: female tail; c: male pharyngeal region; d: male caudal region.

**Measurements.** Table 4. **Adult. Figs 22, 23.** Cuticle without longitudinal striation. Space between dorsal and ventral limbs of amphid developed. Lateral alae narrow, with crenate margins. It originates at posterior end of ventral limb of amphid and ends in a long expansion along posterior third of tail. Secretory-excretory pore opens posterior to cardia, at level with anterior part of intestine. Tail with clavate terminal part. **Male.** Not found. **Female.** Anteriormost edge of amphid positioned anterior to oral opening. Dorsal limb of amphid extends for a short distance posteriorly, equal to 1.7 labial region diameters in length. Ventral limb of amphid extends along pharyngeal region to level of anterior third of the pharynx; 1.4 times the length of dorsal limb. Ventral limb of amphid 1.3 wider than dorsal limb. Interamphideal space very broad, extends along pharyngeal region to same level as ventral limb. Vagina straight. Spermatheca absent, spermathozoa in uterus. One pair of ventrosublateral setae located along middle of tail and one pair of dorsosublateral setae located half way between posterior end of lateral alae and tal terminus.

#### Diagnosis

Body 0.93 mm long; cuticle without longitudinal striation; anteriormost edge of amphid anterior to oral opening; dorsal limb of amphid equal to 1.7 labial region diameters in female; ventral limb of amphid extends towards anterior part of pharynx, 1.4 times the length of dorsal limb in female; ventral limb of amphid is 1.3 wider than dorsal limb; interamphideal space extends to same level as ventral limb; secretory-excretory pore opens posterior to cardia; tail equal to 7.3 anal body diameters in length, with clavate terminal part.

#### Taxon discussion

Original description of this species is based on a single male specimen (Gerlach 1950), while the recent specimen is a female. Both specimens are similar in most body measurements and in the morphology of the anterior end: position of the oral opening posterior to the anteriormost edge of the amphid and in the shape of the amphid with developed interamphideal space, ventral limb of the amphid 1.4 times the length of the dorsal limb and extending to the level of the anterior third of the pharynx. Observed differences between the two specimens include slender body (a = 42) shorter amphid (36 µm) and broader annules (5 µm) in the male (vs. a = 28, 46 µm and 2 µm, respectively, in the female).

### Campylaimus inaequalis

Cobb, 1920

25B6A589-DC56-5811-BEA6-3A27C603DA4D

http://nemys.ugent.be/aphia.php?p=taxdetails&id=121401

#### Materials

- **Type status:** Other material. **Occurrence:** catalogNumber: SMNH-177115; individualCount: 1; sex: female; **Location:** waterBody: Skagerrak; country: Sweden; locality: Bratten; minimumDepthInMeters: 25; maximumDepthInMeters: 50; verbatimLatitude: 58°22,20'N; verbatimLongitude: 11°09,26'E; **Identification:** identifiedBy: Oleksandr Holovachov; dateIdentified: 2018; **Event:** year: 2011; month: 8; day: 9; habitat: Muddy sand
- **Type status:** Other material. **Occurrence:** catalogNumber: SMNH-177116; individualCount: 1; sex: female; **Location:** waterBody: Skagerrak; country: Sweden; locality: Gullmarsfjord, Klubban; verbatimLatitude: 58°15,03'N; verbatimLongitude: 11°27,54'E; **Identification:** identifiedBy: Oleksandr Holovachov; dateIdentified: 2018; **Event:** year: 2014; month: 8; day: 14; habitat: Shells, gravel, sand, mud
- **Type status:** Other material. **Occurrence:** catalogNumber: SMNH-177117; individualCount: 1; sex: female; **Location:** waterBody: Skagerrak; country: Sweden; locality: Gullmarsfjord, Klubban; verbatimLatitude: 58°15,03'N; verbatimLongitude: 11°27,54'E; **Identification:** identifiedBy: Oleksandr Holovachov; dateIdentified: 2018; **Event:** year: 2014; month: 8; day: 14; habitat: Shells, gravel, sand, mud
- **Type status:** Other material. **Occurrence:** catalogNumber: SMNH TYPE-9205 (same slide as *C. triclados*); individualCount: 1; sex: male; **Location:** waterBody: Skagerrak; country: Sweden; locality: Gullmarsfjord, near Fiskebäckskil; minimumDepthInMeters: 30; maximumDepthInMeters: 30; verbatimLatitude: 58°15,25'N; verbatimLongitude: 11°27,30'E; **Identification:** identifiedBy: Oleksandr Holovachov; dateIdentified: 2018; **Event:** year: 2011; month: 8; day: 11; habitat: Soft mud

#### Description

**Figure 24.**
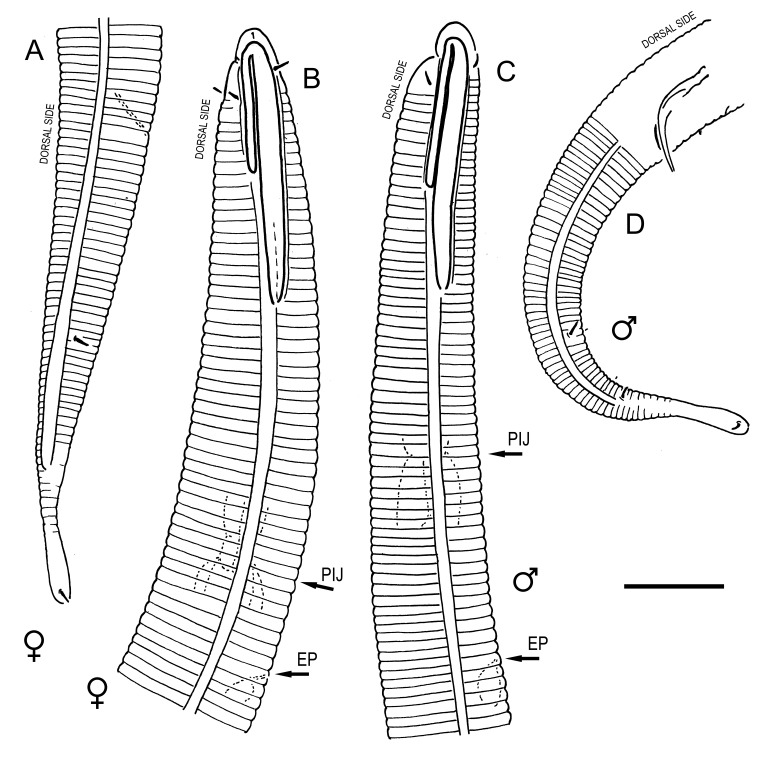
*Campylaimus inaequalis* Cobb, 1920 (scale bars = 20 µm, PIJ = pharyngo-intestinal junction/cardia, EP = secretory-excretory pore): a: female tail; b: female pharyngeal region; c: male pharyngeal region; d: male caudal region.

**Measurements.** Table 5. **Adult. Figs 24, 25.** Cuticle without longitudinal striation. Space between dorsal and ventral limbs of amphid not developed. Lateral alae narrow, with straight margins. It originates at posterior end of ventral limb of amphid and ends along posterior third of the tail. Secretory-excretory pore opens posterior to cardia, at level with anterior part of intestine. Tail with clavate terminal part. **Male.** Anteriormost edge of amphid positioned anterior to oral opening. Dorsal limb of amphid extends for a short distance posteriorly, equal to 2.3 labial region diameters in length. Ventral limb of amphid extends along pharyngeal region to level of mid-pharynx; 1.7 times the length of dorsal limb. Ventral limb of amphid two times wider than dorsal limb. Spicules with rounded manubrium and conoid, arcuate shaft. Gubernaculum platelike, without apophysis. One small precloacal papilliform sensillum located on 5th annule anterior to cloacal opening. Second precloacal pore-like sensillum located on 8th annule anterior to cloacal opening. Two pairs of ventrosublateral setae located along posterior half of tail and one pair of sublateral setae located subterminally. **Female.** Anteriormost edge of amphid positioned anterior to oral opening. Dorsal limb of amphid extends for a short distance posteriorly, equal to 1.9–2.0 labial region diameters in length. Ventral limb of amphid extends along pharyngeal region to level of midpharynx; 2.0–2.3 times the length of dorsal limb. Ventral limb of amphid two times wider than dorsal limb. Vagina straight. One pair of ventrosublateral setae located along middle of tail and one pair of dorsosublateral setae located subterminally.

#### Diagnosis

Body 0.53–0.64 mm long; cuticle without longitudinal striation; anteriormost edge of amphid anterior to oral opening; dorsal limb of amphid equal to 2.3 labial region diameters in male, 1.9–2.0 labial region diameters in female; ventral limb of amphid extends towards mid-pharynx in both sexes, 1.7 times the length of dorsal limb in male and 2.0–2.3 times the length of dorsal limb in female; ventral limb of amphid is twice as wide as dorsal limb; interamphideal space absent; secretory-excretory pore opens posterior to cardia; spicules 24 µm long; precloacal supplements indistinct; tail equal to 4.5–5.8 anal body diameters in length, with clavate terminal part.

#### Taxon discussion

The original description of *C. inaequalis* is detailed and well illustrated (Cobb 1920), although without most of the morphometric characters which are currently considered important. The identification of recent specimens is based on the combination of the following two morphological features: ventral limb of the amphid is roughtly twice (1.7–2.3) as long as the dorsal limb and clavate terminal part of the tail. Specimens described by Vitiello (1970) under the name Campylaimus cf. inaequalis cannot be identified to any known species because of the unclear description of the amphid given in the text of an article, in particular the length of the ventral amphideal limb cannot be deduced from either the drawings or the text.

### Campylaimus striatus

Boucher & Helléouët, 1977

5C98D104-2E3C-503D-B065-A2392EFEAD17

http://nemys.ugent.be/aphia.php?p=taxdetails&id=230034

#### Materials

- **Type status:** Other material. **Occurrence:** catalogNumber: SMNH-177109; individualCount: 1; sex: male; **Location:** waterBody: Skagerrak; country: Sweden; locality: Gullmarsfjord, near Fiskebäckskil; minimumDepthInMeters: 44; maximumDepthInMeters: 44; verbatimLatitude: 58°15,63'N; verbatimLongitude: 11°27,72'E; **Identification:** identifiedBy: O. Holovachov; dateIdentified: 2018; **Event:** year: 2011; month: 8; day: 11; habitat: Soft mud
- **Type status:** Other material. **Occurrence:** catalogNumber: SMNH-177112; individualCount: 1; sex: female; **Location:** waterBody: Skagerrak; country: Sweden; locality: Gullmarsfjord, near Fiskebäckskil; minimumDepthInMeters: 30; maximumDepthInMeters: 39; verbatimLatitude: 58°15,13'N; verbatimLongitude: 11°27,31'E; **Identification:** identifiedBy: O. Holovachov; dateIdentified: 2018; **Event:** year: 2010; month: 8; day: 21; habitat: Mud
- **Type status:** Other material. **Occurrence:** catalogNumber: SMNH-177114; individualCount: 1; sex: male; **Location:** waterBody: Skagerrak; country: Sweden; locality: Gullmarsfjord, Kristineberg-Lysekil; minimumDepthInMeters: 53; maximumDepthInMeters: 53; verbatimLatitude: 58°15,73'N; verbatimLongitude: 11°26,10'E; **Identification:** identifiedBy: O. Holovachov; dateIdentified: 2018; **Event:** year: 2014; month: 8; day: 14; habitat: Mud
- **Type status:** Other material. **Occurrence:** catalogNumber: SMNH TYPE-9206 (same slide as *C. triclados*); individualCount: 1; sex: male; **Location:** waterBody: Skagerrak; country: Sweden; locality: Gullmarsfjord, near Fiskebäckskil; minimumDepthInMeters: 30; maximumDepthInMeters: 30; verbatimLatitude: 58°15,25'N; verbatimLongitude: 11°27,30'E; **Identification:** identifiedBy: Oleksandr Holovachov; dateIdentified: 2018; **Event:** year: 2011; month: 8; day: 11; habitat: Soft mud
- **Type status:** Other material. **Occurrence:** catalogNumber: SMNH TYPE-9208 (same slide as *C. longispiculus*); individualCount: 3; sex: female; **Location:** waterBody: Skagerrak; country: Sweden; locality: Bratten; minimumDepthInMeters: 139; maximumDepthInMeters: 153; verbatimLatitude: 58°34,19'N; verbatimLongitude: 10°38,20'E; **Identification:** identifiedBy: Oleksandr Holovachov; dateIdentified: 2018; **Event:** year: 2012; month: 10; day: 12; habitat: Soft bottom

#### Description

**Figure 26.**
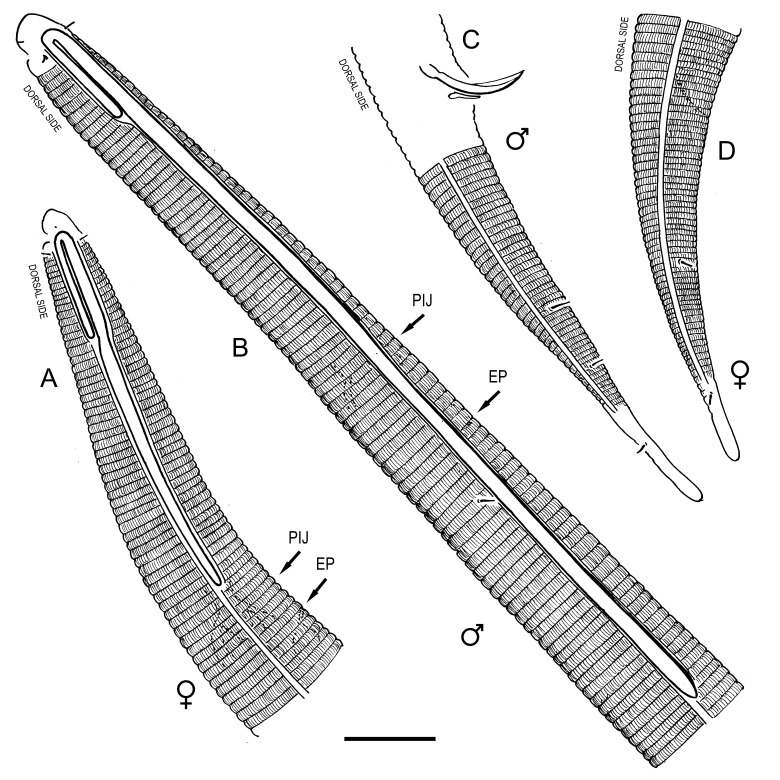
*Campylaimus striatus* Boucher & Helléouët, 1977 (scale bars = 20 µm, PIJ = pharyngo-intestinal junction/cardia, EP = secretory-excretory pore): a: female pharyngeal region; b: male pharyngeal region; c: male caudal region; d: female tail.

**Measurements.** Table 5. **Adult. Figs 26, 27, 28.** Cuticle with distinct longitudinal striation. Space between dorsal and ventral limbs of amphid not developed. Lateral alae narrow, appearing externally as smooth uniform band with straight margins. It originates at posterior end of ventral limb of amphid and ends along posterior fifth of tail. Secretory-excretory pore opens posterior to cardia, at level with anterior part of intestine. Tail with digitate terminal part. **Male.** Anteriormost edge of amphid positioned at level with oral opening. Dorsal limb of amphid extends for a short distance posteriorly, equal to 1.9-2.0 labial region diameters in length. Ventral limb of amphid extends along pharyngeal region to level of anterior part of intestine; 8.3-9.5 times the length of dorsal limb. Ventral limb of amphid 1.7-2.5 wider than dorsal limb. Spicules with rounded manubrium and conoid, arcuate shaft. Gubernaculum platelike, without developed apophysis, but with proximal part bent caudally. Precloacal supplements indistinct/absent. Two pairs of ventrosublateral setae located along posterior half of tail and one pair of dorsosublateral setae located just posterior end of the lateral alae. **Female.** Anteriormost edge of amphid positioned at level with oral opening. Dorsal limb of amphid extends for a short distance posteriorly, equal to 1.8-2.6 labial region diameters in length. Ventral limb of amphid extends along pharyngeal region just anterior or just posterior to pharyngo-intestinal junction; 3.4-5.7 times the length of dorsal limb. Ventral limb of amphid 1–2 times wider than dorsal limb. Vagina straight. One pair of ventrosublateral setae located along middle of tail and one pair of sublateral setae located just posterior to end of lateral alae.

#### Diagnosis

Body 0.52–0.73 mm long; cuticle with longitudinal striation; anteriormost edge of amphid at level with oral opening; dorsal limb of amphid equal to 1.9–2.0 labial region diameters in male, 1.8–2.6 labial region diameters in female; ventral limb of amphid extends towards anterior part of intestine in male and towards pharyngo-intestinal junction in female, 8.3–9.5 times the length of dorsal limb in male and 3.4–5.7 times the length of dorsal limb in female; ventral limb of amphid is 1–2 wider than dorsal limb; interamphideal space absent; secretory-excretory pore opens posterior to cardia; spicules 26–27 µm long; precloacal supplements indistinct; tail equal to 4.6–5.7 anal body diameters in length, with digitate terminal part.

#### Taxon discussion

*C. striatus* is so far the only known species of the genus with distinctly striated cuticular annulation. Original description of this species is based on three males and two females (although only two males and one female were measured, Boucher and Helléouët (1977)) while recent population includes two males and four females. Both populations are similar in the morphology of the cuticle (presence of longitudinal striations), in the size and proportions of the amphid and presence of sexual dimorphism in amphid size (ventral limb of the amphid extends towards anterior part of the intestine in male and towards the pharyngo-intestinal junction in female). Type specimens are relatively larger (L = 0.83-0.89 mm vs. 0.52–0.73 mm), with longer cephalic setae (6–7 µm vs. 1–3 µm) and longer tail (144–154 µm, c' = 6.3–9.6 vs. 82–112 µm, c' = 4.6–5.7). Since no qualitative differences between the two populations can be found and since differences in morphometrics can be explained by georgaphical variability or difficulty in measuring (cephalic sensilla), both populations are herefore considered to be conspecific.

### Campylaimus amphidialis

Fadeeva, Mordukhovich & Zograf, 2016

800AD0FD-B51D-55F1-9EE3-55739A8054E6

http://nemys.ugent.be/aphia.php?p=taxdetails&id=883817

#### Materials

- **Type status:** Other material. **Occurrence:** catalogNumber: SMNH-177093; individualCount: 2; sex: 1 female, 1 male; **Location:** waterBody: Skagerrak; country: Sweden; locality: Gullmarsfjord, Kristineberg-Lysekil; minimumDepthInMeters: 53; maximumDepthInMeters: 53; verbatimLatitude: 58°15,73'N; verbatimLongitude: 11°26,10'E; **Identification:** identifiedBy: O. Holovachov; dateIdentified: 2018; **Event:** year: 2014; month: 8; day: 14; habitat: Mud
- **Type status:** Other material. **Occurrence:** catalogNumber: SMNH-177094; individualCount: 2; **Location:** waterBody: Skagerrak; country: Sweden; locality: Gullmarsfjord, Kristineberg-Lysekil; minimumDepthInMeters: 53; maximumDepthInMeters: 53; verbatimLatitude: 58°15,73'N; verbatimLongitude: 11°26,10'E; **Identification:** identifiedBy: O. Holovachov; dateIdentified: 2018; **Event:** year: 2014; month: 8; day: 14; habitat: Mud
- **Type status:** Other material. **Occurrence:** catalogNumber: SMNH-177095; individualCount: 3; sex: male; **Location:** waterBody: Skagerrak; country: Sweden; locality: Bratten; minimumDepthInMeters: 390; maximumDepthInMeters: 428; verbatimLatitude: 58°22,30'N; verbatimLongitude: 10°20,33'E; **Identification:** identifiedBy: O. Holovachov; dateIdentified: 2018; **Event:** year: 2012; month: 10; day: 10; habitat: Soft bottom
- **Type status:** Other material. **Occurrence:** catalogNumber: SMNH-177098; individualCount: 5; sex: 3 females, 2 males; **Location:** waterBody: Skagerrak; country: Sweden; locality: Bratten; minimumDepthInMeters: 139; maximumDepthInMeters: 153; verbatimLatitude: 58°34,19'N; verbatimLongitude: 10°38,20'E; **Identification:** identifiedBy: O. Holovachov; dateIdentified: 2018; **Event:** year: 2012; month: 10; day: 12; habitat: Soft bottom
- **Type status:** Other material. **Occurrence:** catalogNumber: SMNH-177099; individualCount: 1; sex: male; **Location:** waterBody: Skagerrak; country: Sweden; locality: Gullmarsfjord, near Fiskebäckskil; minimumDepthInMeters: 8; maximumDepthInMeters: 15; verbatimLatitude: 58°15,09'N; verbatimLongitude: 11°27,54'E; **Identification:** identifiedBy: O. Holovachov; dateIdentified: 2018; **Event:** year: 2011; month: 8; day: 11; habitat: Muddy sand
- **Type status:** Other material. **Occurrence:** catalogNumber: SMNH-177102; individualCount: 1; sex: male; **Location:** waterBody: Skagerrak; country: Sweden; locality: Bratten; minimumDepthInMeters: 180; maximumDepthInMeters: 216; verbatimLatitude: 58°28,16'N; verbatimLongitude: 10°37,04'E; **Identification:** identifiedBy: O. Holovachov; dateIdentified: 2018; **Event:** year: 2012; month: 10; day: 11; habitat: Hard bottom
- **Type status:** Other material. **Occurrence:** catalogNumber: SMNH-177103; individualCount: 1; sex: male; **Location:** waterBody: Skagerrak; country: Sweden; locality: Bratten; minimumDepthInMeters: 352; maximumDepthInMeters: 374; verbatimLatitude: 58°19,18'N; verbatimLongitude: 10°29,34'E; **Identification:** identifiedBy: O. Holovachov; dateIdentified: 2018; **Event:** year: 2012; month: 10; day: 10; habitat: Soft bottom
- **Type status:** Other material. **Occurrence:** catalogNumber: SMNH-177104; individualCount: 2; sex: 1 female, 1 male; **Location:** waterBody: Skagerrak; country: Sweden; locality: Bratten; minimumDepthInMeters: 232; maximumDepthInMeters: 240; verbatimLatitude: 58°27,40'N; verbatimLongitude: 10°33,56'E; **Identification:** identifiedBy: Oleksandr Holovachov; dateIdentified: 2018; **Event:** year: 2012; month: 10; day: 12; habitat: Soft bottom
- **Type status:** Other material. **Occurrence:** catalogNumber: SMNH-177105; individualCount: 3; sex: 2 males, 1 female; **Location:** waterBody: Skagerrak; country: Sweden; locality: Bratten; minimumDepthInMeters: 248; maximumDepthInMeters: 316; verbatimLatitude: 58°28,22'N; verbatimLongitude: 10°29,39'E; **Identification:** identifiedBy: O. Holovachov; dateIdentified: 2018; **Event:** year: 2012; month: 10; day: 11; habitat: Mixed bottom
- **Type status:** Other material. **Occurrence:** catalogNumber: SMNH-177106; individualCount: 1; sex: female; **Location:** waterBody: Skagerrak; country: Sweden; locality: Bratten; minimumDepthInMeters: 390; maximumDepthInMeters: 428; verbatimLatitude: 58°22,30'N; verbatimLongitude: 10°20,33'E; **Identification:** identifiedBy: O. Holovachov; dateIdentified: 2018; **Event:** year: 2012; month: 10; day: 10; habitat: Soft bottom
- **Type status:** Other material. **Occurrence:** catalogNumber: SMNH-177107; individualCount: 1; sex: male; **Location:** waterBody: Skagerrak; country: Sweden; locality: Bratten; minimumDepthInMeters: 351; maximumDepthInMeters: 387; verbatimLatitude: 58°22,19'N; verbatimLongitude: 10°23,50'E; **Identification:** identifiedBy: O. Holovachov; dateIdentified: 2018; **Event:** year: 2012; month: 10; day: 10; habitat: Soft bottom
- **Type status:** Other material. **Occurrence:** catalogNumber: SMNH-177108; individualCount: 1; sex: male; **Location:** waterBody: Skagerrak; country: Sweden; locality: Bratten; minimumDepthInMeters: 180; maximumDepthInMeters: 216; verbatimLatitude: 58°28,16'N; verbatimLongitude: 10°37,04'E; **Identification:** identifiedBy: O. Holovachov; dateIdentified: 2018; **Event:** year: 2012; month: 10; day: 11; habitat: Hard bottom
- **Type status:** Other material. **Occurrence:** catalogNumber: SMNH-177110; individualCount: 1; sex: male; **Location:** waterBody: Skagerrak; country: Sweden; locality: Gullmarsfjord, Kristineberg-Lysekil; minimumDepthInMeters: 53; maximumDepthInMeters: 53; verbatimLatitude: 58°15,73'N; verbatimLongitude: 11°26,10'E; **Identification:** identifiedBy: O. Holovachov; dateIdentified: 2018; **Event:** year: 2014; month: 8; day: 14; habitat: Mud
- **Type status:** Other material. **Occurrence:** catalogNumber: SMNH-177112; individualCount: 1; sex: male; **Location:** waterBody: Skagerrak; country: Sweden; locality: Gullmarsfjord, near Fiskeb√§ckskil; minimumDepthInMeters: 30; maximumDepthInMeters: 39; verbatimLatitude: 58°15,13'N; verbatimLongitude: 11°27,31'E; **Identification:** identifiedBy: O. Holovachov; dateIdentified: 2018; **Event:** year: 2010; month: 8; day: 21; habitat: Mud
- **Type status:** Other material. **Occurrence:** catalogNumber: SMNH-177113; individualCount: 1; sex: male; **Location:** waterBody: Skagerrak; country: Sweden; locality: Bratten; minimumDepthInMeters: 55; maximumDepthInMeters: 70; verbatimLatitude: 58°22,19'N; verbatimLongitude: 11°04,55'E; **Identification:** identifiedBy: O. Holovachov; dateIdentified: 2018; **Event:** year: 2011; month: 8; day: 9; habitat: Mud and clay
- **Type status:** Other material. **Occurrence:** catalogNumber: SMNH-177114; individualCount: 1; sex: female; **Location:** waterBody: Skagerrak; country: Sweden; locality: Gullmarsfjord, Kristineberg-Lysekil; minimumDepthInMeters: 53; maximumDepthInMeters: 53; verbatimLatitude: 58°15,73'N; verbatimLongitude: 11°26,10'E; **Identification:** identifiedBy: O. Holovachov; dateIdentified: 2018; **Event:** year: 2014; month: 8; day: 14; habitat: Mud
- **Type status:** Other material. **Occurrence:** catalogNumber: SMNH-177117; individualCount: 2; sex: 1 female, 1 male; **Location:** waterBody: Skagerrak; country: Sweden; locality: Gullmarsfjord, Klubban; verbatimLatitude: 58°15,03'N; verbatimLongitude: 11°27,54'E; **Identification:** identifiedBy: O. Holovachov; dateIdentified: 2018; **Event:** year: 2014; month: 8; day: 14; habitat: Shells, gravel, sand, mud
- **Type status:** Other material. **Occurrence:** catalogNumber: SMNH-177118; individualCount: 2; sex: male; **Location:** waterBody: Skagerrak; country: Sweden; locality: Gullmarsfjord, near Fiskebäckskil; minimumDepthInMeters: 44; maximumDepthInMeters: 44; verbatimLatitude: 58°15,63'N; verbatimLongitude: 11°27,72'E; **Identification:** identifiedBy: O. Holovachov; dateIdentified: 2018; **Event:** year: 2011; month: 8; day: 11; habitat: Soft mud
- **Type status:** Other material. **Occurrence:** catalogNumber: SMNH-177119; individualCount: 2; sex: female; **Location:** waterBody: Skagerrak; country: Sweden; locality: Gullmarsfjord, Kristineberg-Lysekil; minimumDepthInMeters: 53; maximumDepthInMeters: 53; verbatimLatitude: 58°15,73'N; verbatimLongitude: 11°26,10'E; **Identification:** identifiedBy: O. Holovachov; dateIdentified: 2018; **Event:** year: 2014; month: 8; day: 14; habitat: Mud
- **Type status:** Other material. **Occurrence:** catalogNumber: SMNH-177122; individualCount: 1; sex: male; **Location:** waterBody: Skagerrak; country: Sweden; locality: Gullmarsfjord, near Fiskebäckskil; minimumDepthInMeters: 30; maximumDepthInMeters: 30; verbatimLatitude: 58°15,25'N; verbatimLongitude: 11°27,30'E; **Identification:** identifiedBy: O. Holovachov; dateIdentified: 2018; **Event:** year: 2011; month: 8; day: 11; habitat: Soft mud
- **Type status:** Other material. **Occurrence:** catalogNumber: SMNH-177123; individualCount: 2; sex: female; **Location:** waterBody: Skagerrak; country: Sweden; locality: Bratten; minimumDepthInMeters: 221; maximumDepthInMeters: 260; verbatimLatitude: 58°28,30'N; verbatimLongitude: 10°33,22'E; **Identification:** identifiedBy: O. Holovachov; dateIdentified: 2018; **Event:** year: 2012; month: 10; day: 11; habitat: Soft bottom
- **Type status:** Other material. **Occurrence:** catalogNumber: SMNH-177096; individualCount: 3; sex: female; **Location:** waterBody: Skagerrak; country: Sweden; locality: Gullmarsfjord, near Fiskebäckskil; minimumDepthInMeters: 8; maximumDepthInMeters: 15; verbatimLatitude: 58°15,09'N; verbatimLongitude: 11°27,54'E; **Identification:** identifiedBy: O. Holovachov; dateIdentified: 2018; **Event:** year: 2011; month: 8; day: 11; habitat: Muddy sand
- **Type status:** Other material. **Occurrence:** catalogNumber: SMNH TYPE-9207 (same slide as *C. triclados*); individualCount: 3; sex: 1 female, 2 males; **Location:** waterBody: Skagerrak; country: Sweden; locality: Gullmarsfjord, near Fiskebäckskil; minimumDepthInMeters: 30; maximumDepthInMeters: 30; verbatimLatitude: 58°15,25'N; verbatimLongitude: 11°27,30'E; **Identification:** identifiedBy: Oleksandr Holovachov; dateIdentified: 2018; **Event:** year: 2011; month: 8; day: 11; habitat: Soft mud
- **Type status:** Other material. **Occurrence:** catalogNumber: SMNH TYPE-9206 (same slide as *C. triclados*); individualCount: 3; sex: 1 female, 2 males; **Location:** waterBody: Skagerrak; country: Sweden; locality: Gullmarsfjord, near Fiskebäckskil; minimumDepthInMeters: 30; maximumDepthInMeters: 30; verbatimLatitude: 58°15,25'N; verbatimLongitude: 11°27,30'E; **Identification:** identifiedBy: Oleksandr Holovachov; dateIdentified: 2018; **Event:** year: 2011; month: 8; day: 11; habitat: Soft mud
- **Type status:** Other material. **Occurrence:** catalogNumber: SMNH TYPE-9208; individualCount: 2; sex: 1 female, 1 male; **Location:** waterBody: Skagerrak; country: Sweden; locality: Bratten; minimumDepthInMeters: 139; maximumDepthInMeters: 153; verbatimLatitude: 58°34,19'N; verbatimLongitude: 10°38,20'E; **Identification:** identifiedBy: Oleksandr Holovachov; dateIdentified: 2018; **Event:** year: 2012; month: 10; day: 12; habitat: Soft bottom

#### Description

**Figure 29.**
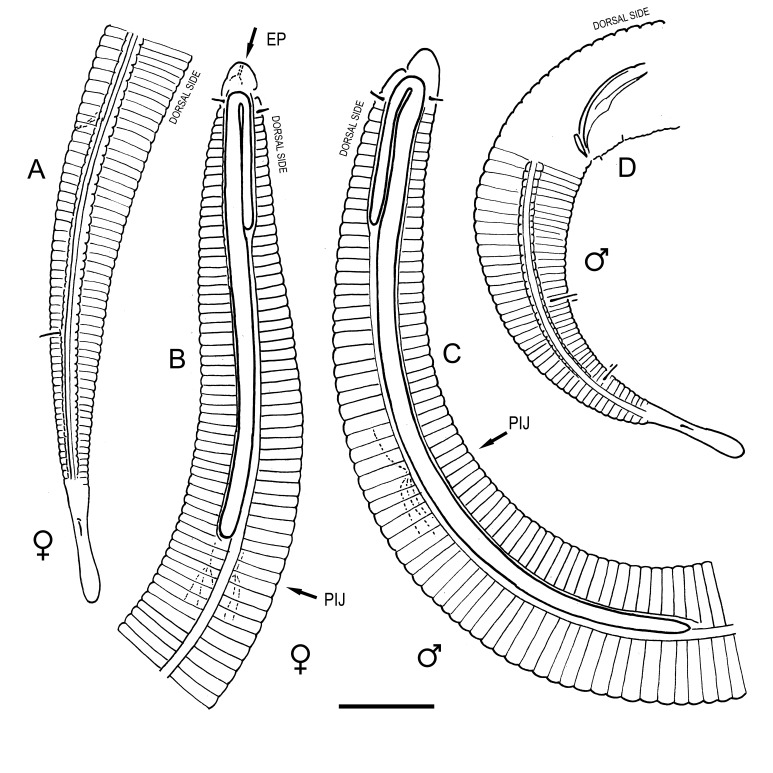
*Campylaimus amphidialis* Fadeeva, Mordukhovich & Zograf, 2016 (scale bars = 20 µm, PIJ = pharyngo-intestinal junction/cardia, EP = secretory-excretory pore): a: female tail; b: female pharyngeal region; c: male pharyngeal region; d: male caudal region.

**Measurements.** Table 6. **Adult. Figs 29, 30, 31, 32.** Cuticle without longitudinal striation. Space between dorsal and ventral limbs of amphid not developed. Lateral alae narrow, appearing externally as smooth uniform band with straight margins along most of body, but with distinct subcuticular channel and crenate margins when focused midway (Fig. 32a, b) along cloacal/anal region and anterior part of tail. It originates at posterior end of ventral limb of amphid and ends along posterior third of tail. Secretory-excretory pore opens apically on anterior end (Fig. 31b, c, d, e). Tail with clavate terminal part. **Male.** Anteriormost edge of amphid positioned at level with oral opening. Dorsal limb of amphid extends for a short distance posteriorly, equal to 2.7–3.5 labial region diameters in length. Ventral limb of amphid extends along pharyngeal region to level of anterior part of intestine; 4.6–5.3 times the length of dorsal limb. Ventral limb of amphid 1.3–1.7 wider than dorsal limb. Spicules with rounded manubrium and conoid, arcuate shaft. Gubernaculum platelike, without apophysis. One small precloacal papilliform sensillum located on 2nd annule anterior to cloacal opening (Fig. 32c, d). Second precloacal pore-like sensillum located on 7th-8th annule anterior to cloacal opening (Fig. 32c, d). Two pairs of ventrosublateral setae located along posterior half of tail and one pair of dorsosublateral setae located half way between posterior end of lateral alae and tail terminus. **Female.** Anteriormost edge of amphid positioned at level with oral opening. Dorsal limb of amphid extends for a short distance posteriorly, equal to 2.8–3.2 labial region diameters in length. Ventral limb of amphid extends along pharyngeal region just anterior to pharyngo-intestinal junction; 3.2–3.3 times the length of dorsal limb. Ventral limb of amphid 1.3–2.0 times wider than dorsal limb. Vagina straight (Fig. 31f). One pair of ventrosublateral setae located along middle of tail and one pair of sublateral setae located 1/3 way between posterior end of lateral alae and tail terminus.

#### Diagnosis

Body 0.53–0.63 mm long; cuticle without longitudinal striation; anteriormost edge of amphid at level with oral opening; dorsal limb of amphid equal to 2.7–3.5 labial region diameters in male, 2.8–3.2 labial region diameters in female; ventral limb of amphid extends towards anterior part of intestine in male and pharynx base in female, 4.6–5.3 times the length of dorsal limb in male and 3.2–3.3 times the length of dorsal limb in female; ventral limb of amphid is 1.3–2.0 times wider than dorsal limb; interamphideal space absent; secretory-excretory pore opens apically on anterior end; spicules 24–29 µm long; two precloacal supplements; tail equal to 4.5–6.4 anal body diameters in length, with clavate terminal part.

#### Taxon discussion

The original description of *C. amphidialis* is based only on three females, males not being known to the authors (Fadeeva et al. 2016). Recent specimens are nearly identical to the type population in measurements and general morphology, including size and shape of the amphid. Furthermore, images of the type specimens kindly provided by Dr. N. Fadeeva, clearly show modified lateral alae along the caudal region in one of the specimens, confirming the conspecificity of both populations.

### Campylaimus longispiculus

Holovachov, 2019 sp. n.

E24C09DC-8750-5A33-998B-0CDC38F058E1

urn:lsid:zoobank.org:act:1A98B343-0730-4851-9B82-2B555771C689

#### Materials

- **Type status:** Holotype. **Occurrence:** catalogNumber: SMNH TYPE-9208; individualCount: 1; sex: male; **Location:** waterBody: Skagerrak; country: Sweden; locality: Bratten; minimumDepthInMeters: 139; maximumDepthInMeters: 153; verbatimLatitude: 58°34,19'N; verbatimLongitude: 10°38,20'E; **Identification:** identifiedBy: Oleksandr Holovachov; dateIdentified: 2018; **Event:** year: 2012; month: 10; day: 12; habitat: Soft bottom
- **Type status:** Other material. **Occurrence:** catalogNumber: SMNH-177107; individualCount: 1; sex: male; **Location:** waterBody: Skagerrak; country: Sweden; locality: Bratten; minimumDepthInMeters: 351; maximumDepthInMeters: 387; verbatimLatitude: 58°22,19'N; verbatimLongitude: 10°23,50'E; **Identification:** identifiedBy: O. Holovachov; dateIdentified: 2018; **Event:** year: 2012; month: 10; day: 10; habitat: Soft bottom
- **Type status:** Other material. **Occurrence:** catalogNumber: SMNH-177111; individualCount: 1; sex: male; **Location:** waterBody: Skagerrak; country: Sweden; locality: Bratten; minimumDepthInMeters: 53; maximumDepthInMeters: 53; verbatimLatitude: 58°20,06'N; verbatimLongitude: 11°09,24'E; **Identification:** identifiedBy: O. Holovachov; dateIdentified: 2018; **Event:** year: 2011; month: 8; day: 9; habitat: Muddy sediment

#### Description

**Measurements.** Table 6. **Adult. Figs 22, 33.** Cuticle without longitudinal striation. Space between dorsal and ventral limbs of amphid not developed. Lateral alae narrow, appearing externally as smooth uniform band with straight margins. It originates at posterior end of ventral limb of amphid and fades away along posterior third of tail. Secretory-excretory pore opens posterior to cardia, at level with anterior part of intestine. Tail with clavate terminal part. **Male.** Anteriormost edge of amphid positioned anterior to oral opening. Dorsal limb of amphid extends for a short distance posteriorly, equal to 1.8–2.1 labial region diameters in length. Ventral limb of amphid extends along pharyngeal region to level of anterior part of intestine; 2.8–3.5 times the length of dorsal limb. Ventral limb of amphid equal to or slightly wider than dorsal limb. Spicules with rounded manubrium and elongate, arcuate shaft. Gubernaculum without apophysis. One small precloacal papilliform sensillum located on 2nd annule anterior to cloacal opening (Fig. 33d). Second precloacal pore-like sensillum located on 7th–8th annule anterior to cloacal opening (Fig. 33d). These sensilla are less obvious in the holotype. Two pairs of ventrosublateral setae located along posterior half of tail and one pair of dorsosublateral setae located subterminally. **Female.** Not found.

#### Diagnosis

Body 0.56–0.65 mm long; cuticle without longitudinal striation; anteriormost edge of amphid anterior to oral opening; dorsal limb of amphid equal to 1.8–2.1 labial region diameters in male; ventral limb of amphid extends towards anterior part of intestine in male, 2.8–3.5 times the length of dorsal limb in male; ventral limb of amphid is as wide as dorsal limb; interamphideal space absent; secretory-excretory pore opens posterior to cardia; spicules 28–35 µm long; two precloacal supplements; tail equal to 4.9–5.3 anal body diameters in length, with clavate terminal part.

#### Etymology

Specific epithet refers to relatively long spicules in this species.

#### Taxon discussion

The new species is most similar to *C. amphidialis* and *C. striatus* in the size and shape of the amphid, with ventral limb being more than three times as long as the dorsal limb, especially in males. It differs from both *C. amphidialis* and *C. striatus* in having relatively shorter ventral limb of the amphid, equal to 2.8–3.5 times the dorsal limb (in male), vs. 8.3–9.5 in *C. striatus* and 4.6–5.3 in *C. amphidialis.* It further differs from *C. striatus* in having smooth annulation of cuticle (vs. with longitudinal striation in *C. striatus*) and from *C. amphidialis* in the position of the excretory pore at the level with the anterior part of intestine (vs. apically on the anterior end in *C. amphidialis*) and simple lateral alae along the posterior part of the body (vs. with crenate margins in *C. amphidialis*).

## Identification Keys

### Identification key to species of the genus *Campylaimus* Cobb, 1920

**Table:** 

1	Amphid in the shape of a long longitudinal slit	2
–	Amphid loop-shaped	3
2	Amphid extends along the entire body towards the tail tip	*C. abnormis*
–	Amphid extends along the anterior part of the pharyngeal region only	*C. gracilis*
3	Ventral limb of the amphid extends along the anterior part of the body and does not reach the tail	4
–	Ventral limb of the amphid extends along the entire body towards the tail tip	15
4	Amphid with widely spaced limbs, appearing as three limbs instead of two	5
–	Amphid without space between the limbs, appearing as two limbs only	8
5	All three limbs of different length	*C. triclados* sp.n.
–	Middle and ventral limb equal in length	6
6	Ventral limb of the amphid 2-3 times as long as the dorsal limb	*C. rimatus*
–	Ventral limb of the amphid less than 1.5 times as long as the dorsal limb	7
7	Inter-amphideal space wider than either one of the limbs of the amphid; tail clavate	*C. mirus*
–	Inter-amphideal space is as wide as either one of the limbs of the amphid; tail conoid	*C. orientalis*
8	Ventral and dorsal limbs of the amphid equal in length	9
–	Ventral and dorsal limbs of the amphid unequal in length	10
9	Body longer than 1 mm; amphid longer than 45 µm	*C. cylindricus*
–	Body shorter than 0.8 mm; amphid shorter than 40 µm	*C. pulcher*
10	Dorsal limb of the amphid equal to or shorter than 1/2 of the labial region diameter in length	11
–	Dorsal limb of the amphid longer than the labial region diameter in length	12
11	Body shorter than 0.4 mm	*C. minor*
–	Body longer than 0.8 mm	*C. ponticus*
12	Ventral limb of the amphid ≈ 2 times as long as the dorsal limb	*C. inaequalis*
–	Ventral limb of the amphid > 3 times as long as the dorsal limb	13
13	Cuticle with longitudinal striations	*C. striatus*
–	Cuticle without longitudinal striations	14
14	Excretory pore opens posterior to the pharyngo-intestinal junction	*C. longispiculus* sp.n.
–	Excretory pore opens apically on the lip region	*C. amphidialis*
15	Precloacal papilliform sensilla present	16
–	Precloacal papilliform sensilla absent	18
16	Anterior end of the body cylindrical; cephalic setae equal to one labial region diameter in length	*C. patagonicus*
–	Anterior end of the body conoid; cephalic setae less than 0.5 labial region diameters in length	17
17	Three precloacal papilliform sensilla	*C. bonariensis*
–	Five precloacal papilliform sensilla	*C. arcuatus*
18	Ventral limb of the amphid is two times broader/wider than the dorsal limb	19
–	Both limbs of the amphid are equally broad/wide	20
19	Body 0.9–1.5 mm long; c' = 10–15	*C. lefeverei*
–	Body 0.4–0.8 mm long; c' = 4.8–6.0	*C. tkatchevi*
20	Dorsal limb of the amphid equal to 1.5 labial region diameters in length	*C. siwaschensis*
–	Dorsal limb of the amphid equal to 2-3 labial region diameters in length	21
21	Tail tip clavate	*C. gerlachi*
–	Tail tip conoid	*C. minutus*

## Discussion

In the past, identification and differentiation of various species of *Campylaimus* was based not only on the shape and size of the amphid, but also on the position of the oral opening, various body measurements and, lately, on the number of pre-cloacal supplements (Fadeeva et al. 2016, Huang and Zhang 2006, Villares et al. 2013). Regarding the position of the oral opening, all recent studies showed that in all species of this genus, it is located subdorsally and the subventral position of the oral opening in *C. gerlachi* (Huang and Zhang 2006, Timm 1961), *C. minor* (Timm 1961) and *C. inaequalis* (Cobb 1920) was a misinterpretation that should not be repeated in future publications.

Amongst a large number of morphometric characters used for species differentiation, one feature should be discussed in detail here. The length of the cephalic setae was used to separate *C. minutus* (Fadeeva et al. 2016) and *C. siwaschensis* (Sergeeva 1981) from *C. gerlachi*. As experienced during preparation of this publication, the length of cephalic setae can only be measured precisely when such setae are located perpendicular to the optical axis of the microscope, which is most commonly not the case. Most of the species descriptions thus underestimate the actual length of the cephalic setae, making this morphometric value of limited use for species identification and differentiation.

Another interesting and potentially useful morphological character is the presence and number of precloacal supplements in males. Besides current work, precloacal supplements in males of *Campylaimus* were described only once (Villares et al. 2013), where they were used to separate new species from each other and from other species of the genus. It is clear that these structures are easy to overlook and that they were not included in species descriptions of those species which are now known to possess them, such as *C. amphidialis, C. inaequalis, C. orientalis* and *C. rimatus* (Cobb 1920, Fadeeva et al. 2016, Vitiello 1970).

The position of the secretory-excretory pore is another feature mentioned only in few species descriptions (Tchesunov 1978). Although it is most frequently located at the level with the anterior part of the intestine, one species was found to be different – secretory-excretory pore in *C. amphidialis* is located apically, on the top of the anterior end and is one of the unique features separating this species from the rest of the congeners.

However, the most striking, variable and commonly used character to identify and differentiate *Campylaimus* species is the shape of the amphid, whose origin and evolution was discussed in detail by Tchesunov (2006). Despite a certain level of intraspecific variability and sexual dimorphism, the overall structure of the amphid and relative size of its components (ventral and dorsal limb and, if present, interamphideal space) are so far the most reliable and, in fact, easiest to observe and interpret diagnostic characters. However, it is crucially important to correctly describe the posterior end of the ventral limb of the amphid and to distinguish it from the lateral alae. The description of *C. ponticus* in this sense is a good example where the text of the description contradicts the illustrations (Sergeeva 1981).

In conclusion, the most stable taxonomic characters to be used in the taxonomy and identification of the genus *Campylaimus* are the size and shape of the amphid and its elements (absolute and relative length and width of limbs, presence, length and width of inter-amphideal space), morphology of the cuticle and lateral alae, position of the secretory-excretory pore, length and shape of the spicules and shape of the tail terminus.

## Supplementary Material

XML Treatment for Campylaimus

XML Treatment for Campylaimus gerlachi

XML Treatment for Campylaimus minutus

XML Treatment for Campylaimus tkatchevi

XML Treatment for Campylaimus siwaschensis

XML Treatment for Campylaimus lefeverei

XML Treatment for Campylaimus rimatus

XML Treatment for Campylaimus orientalis

XML Treatment for Campylaimus triclados

XML Treatment for Campylaimus mirus

XML Treatment for Campylaimus inaequalis

XML Treatment for Campylaimus striatus

XML Treatment for Campylaimus amphidialis

XML Treatment for Campylaimus longispiculus

## Figures and Tables

**Figure 2a.**
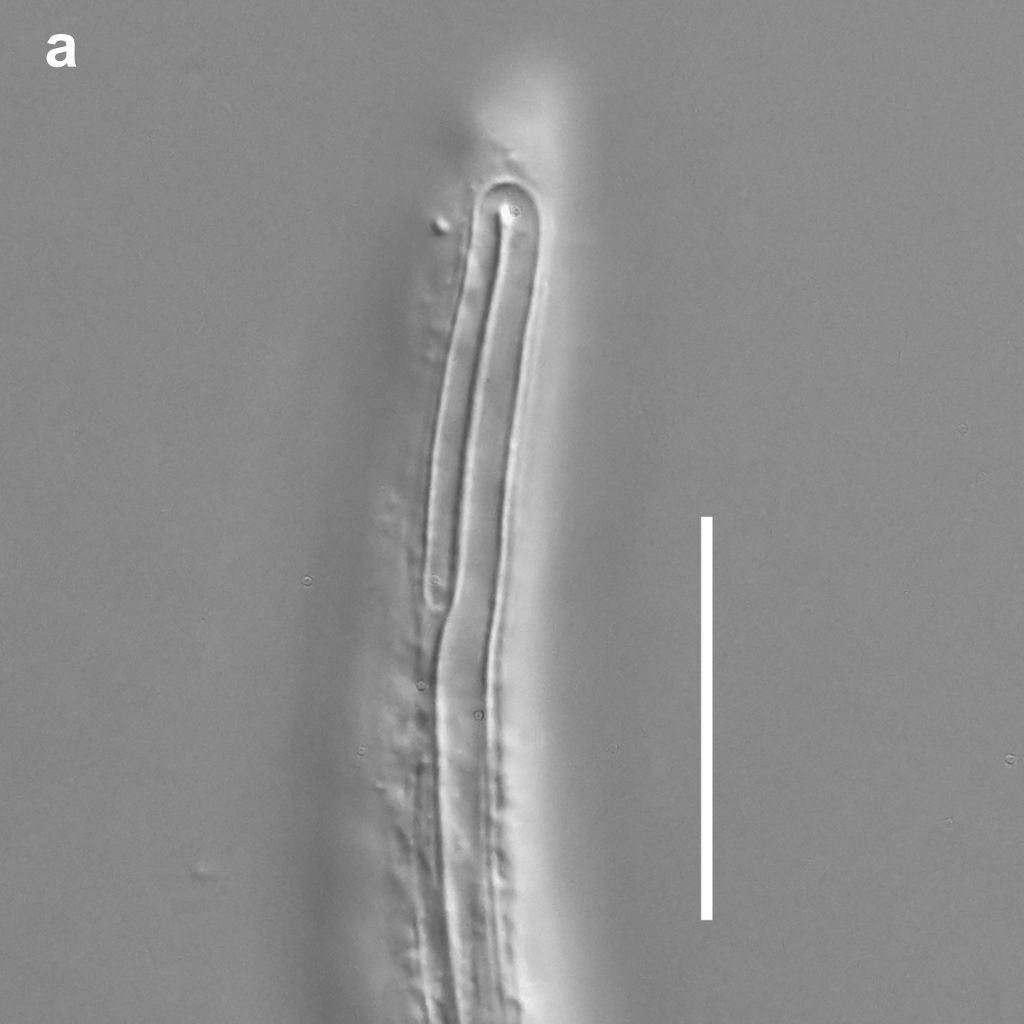
anterior end, surface view showing amphid (ventral side to the left)

**Figure 2b.**
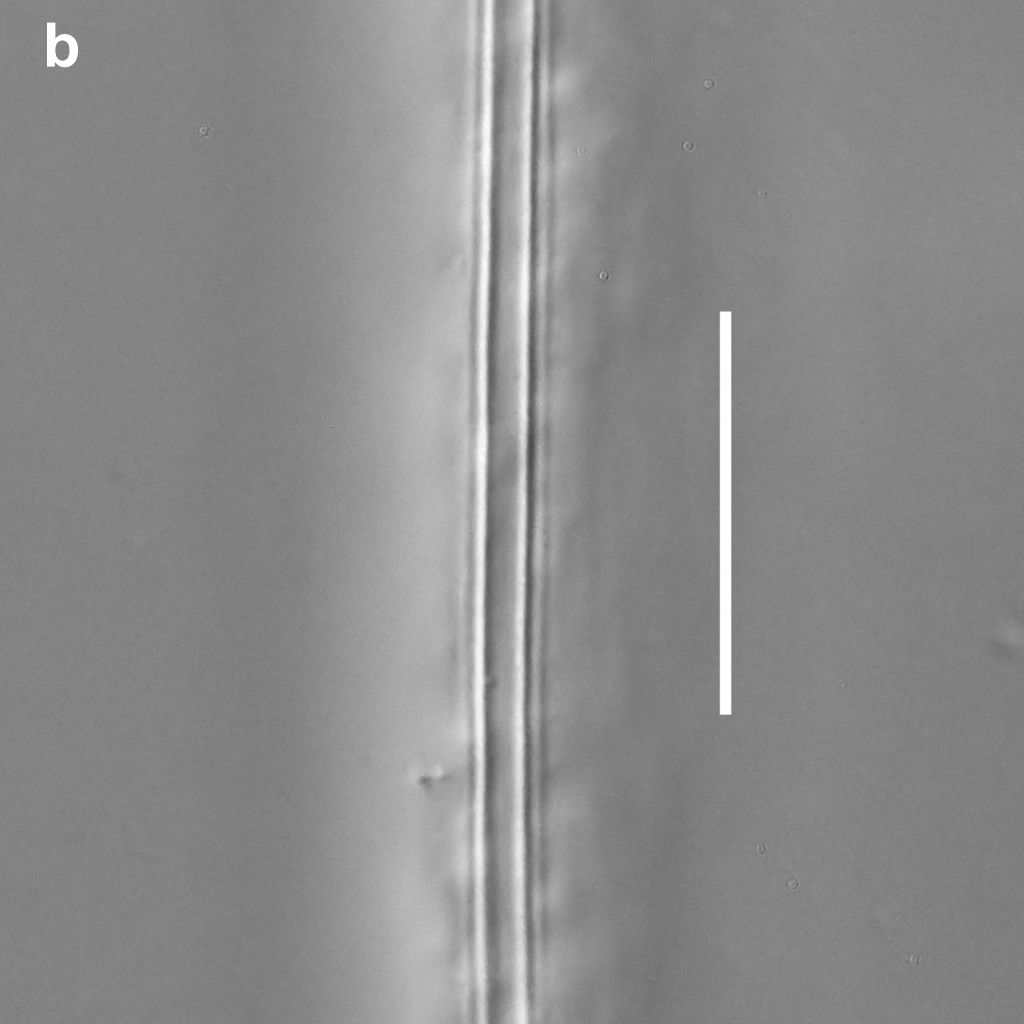
lateral alae at mid-body, surface view

**Figure 2c.**
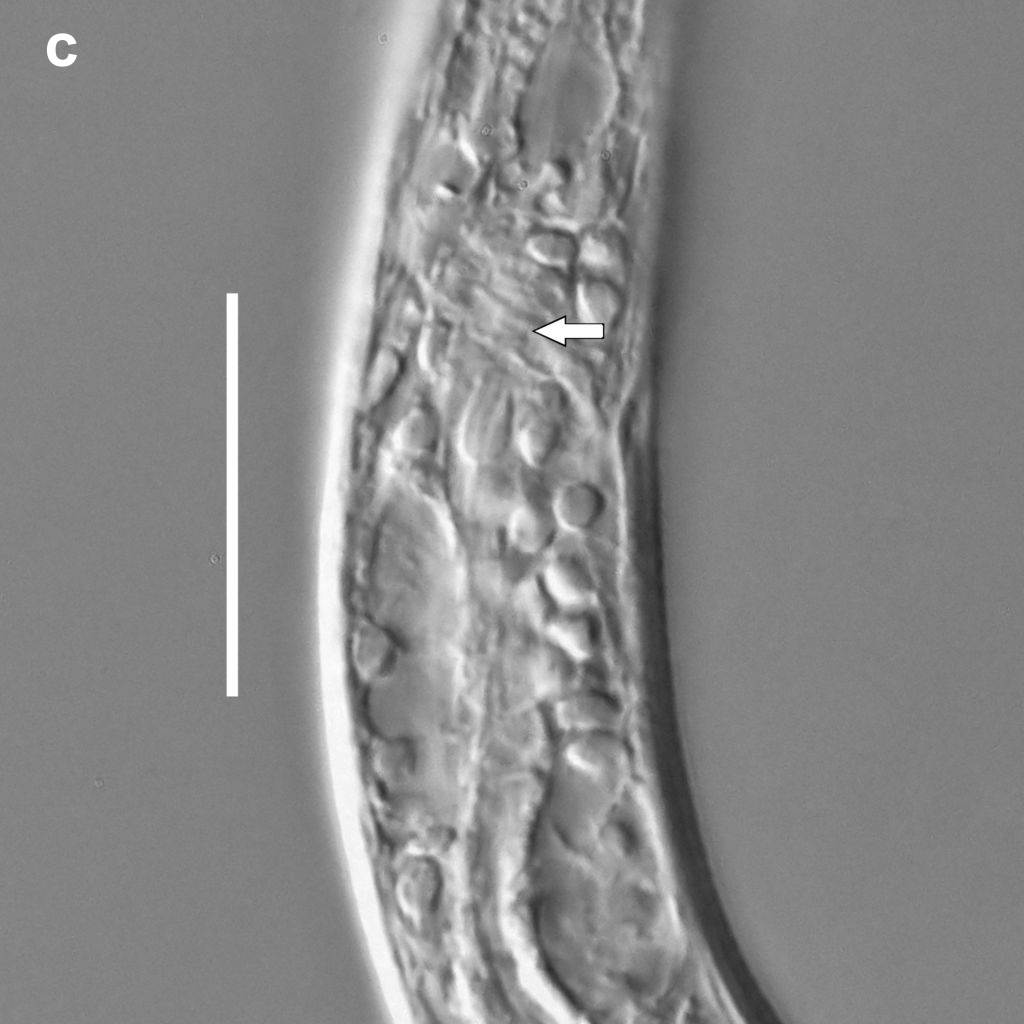
nerve ring surrounding pharyngo-intestinal junction (arrow)

**Figure 2d.**
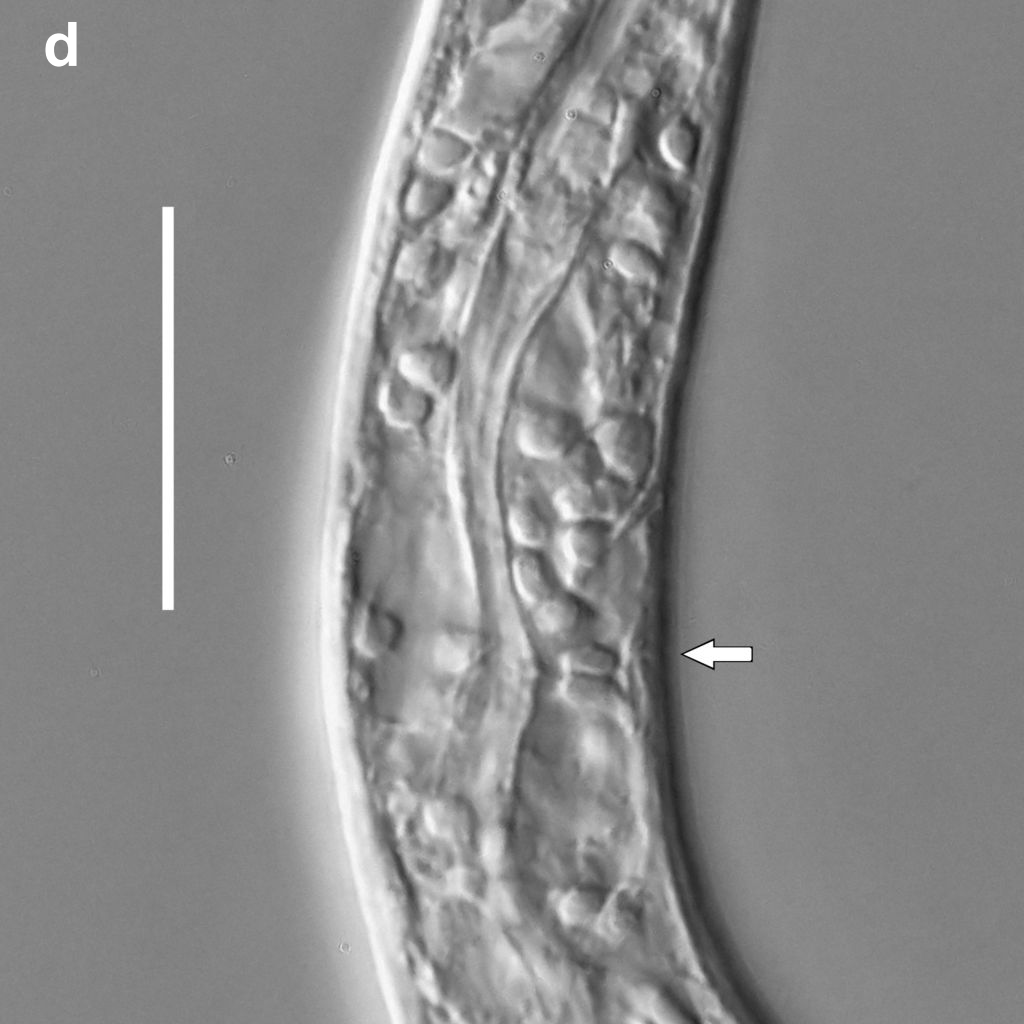
secretory-excretory pore at level with anterior part of intestine (arrow)

**Figure 2e.**
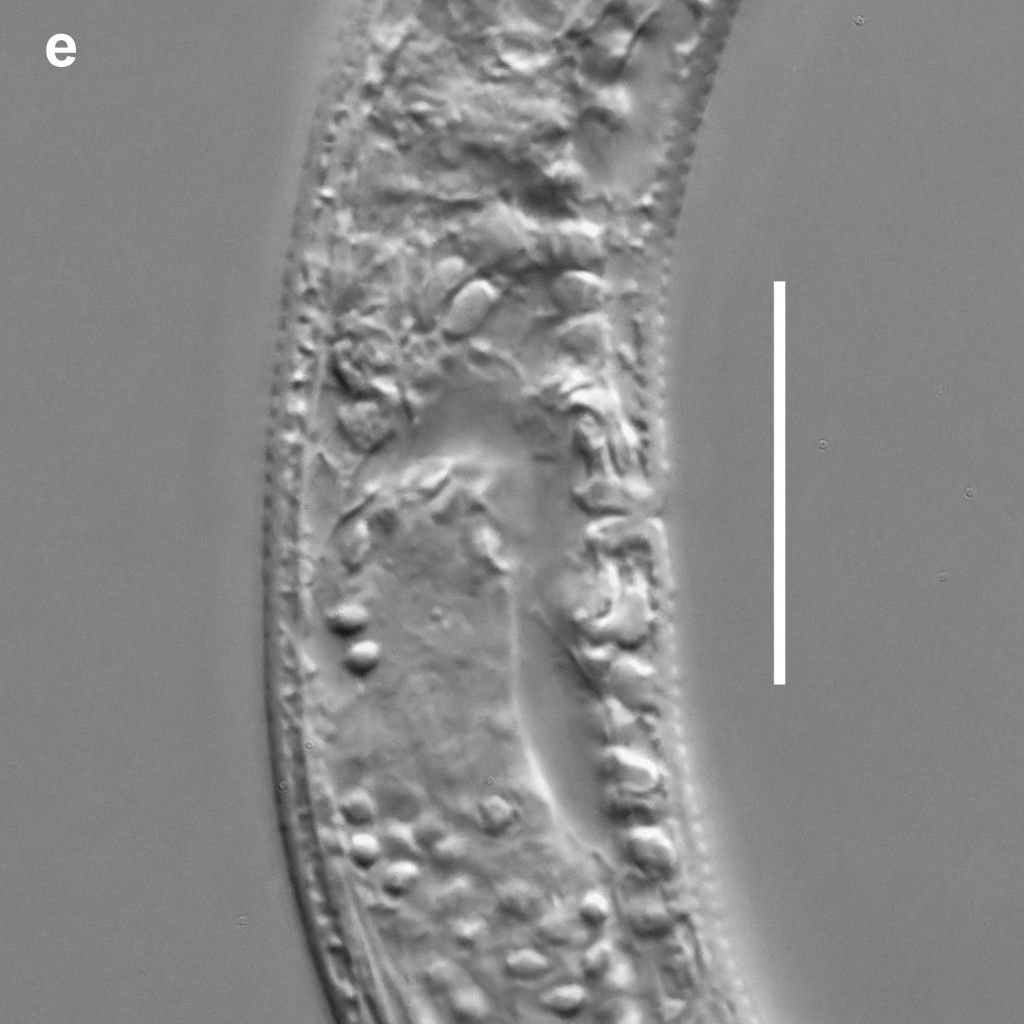
vulval region

**Figure 2f.**
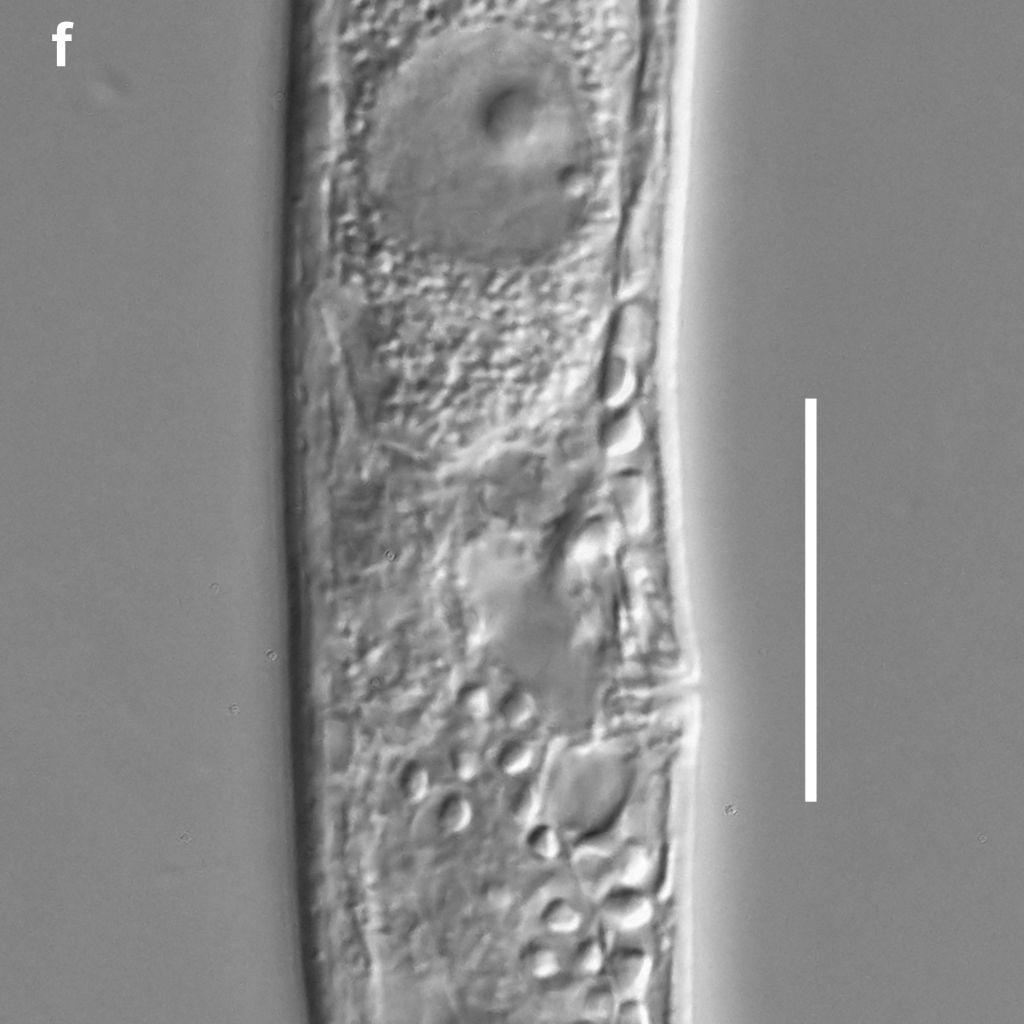
vulval region

**Figure 3a.**
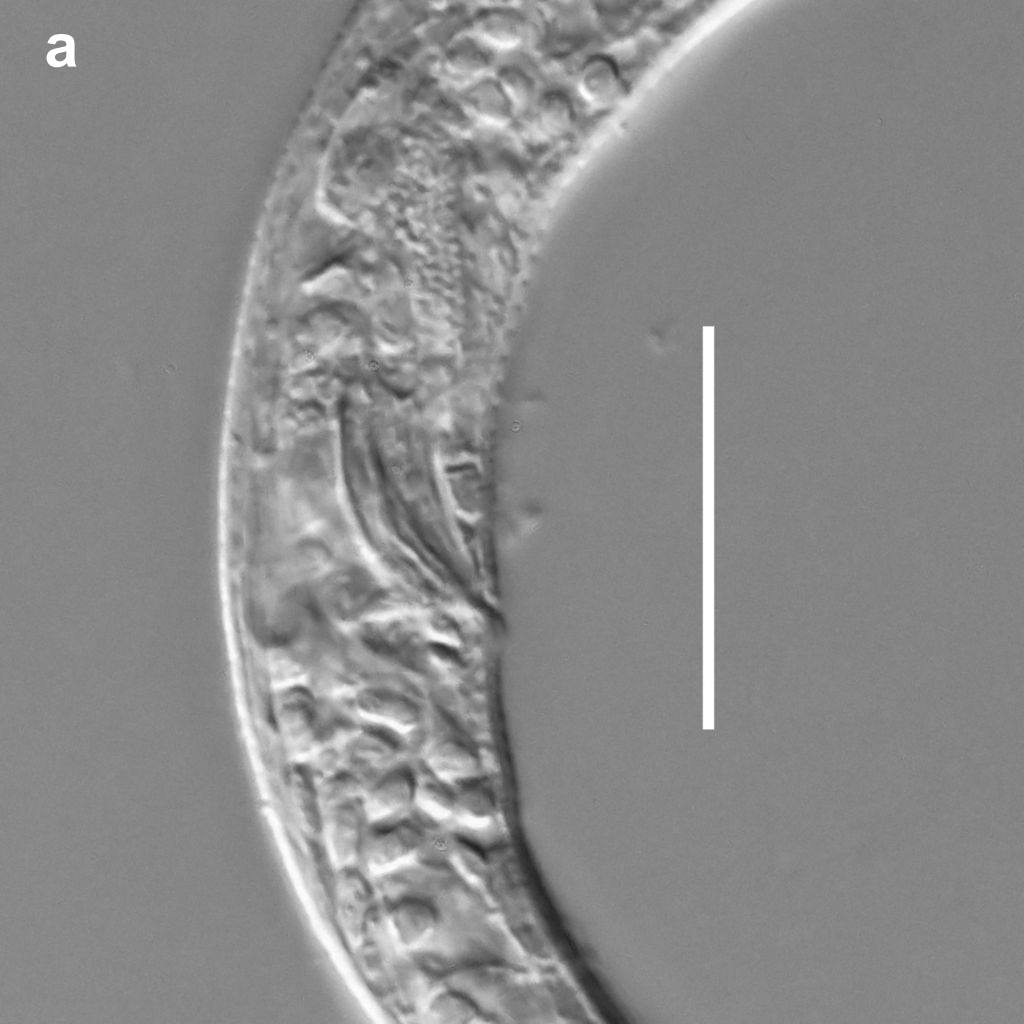
male cloacal region showing spicules

**Figure 3b.**
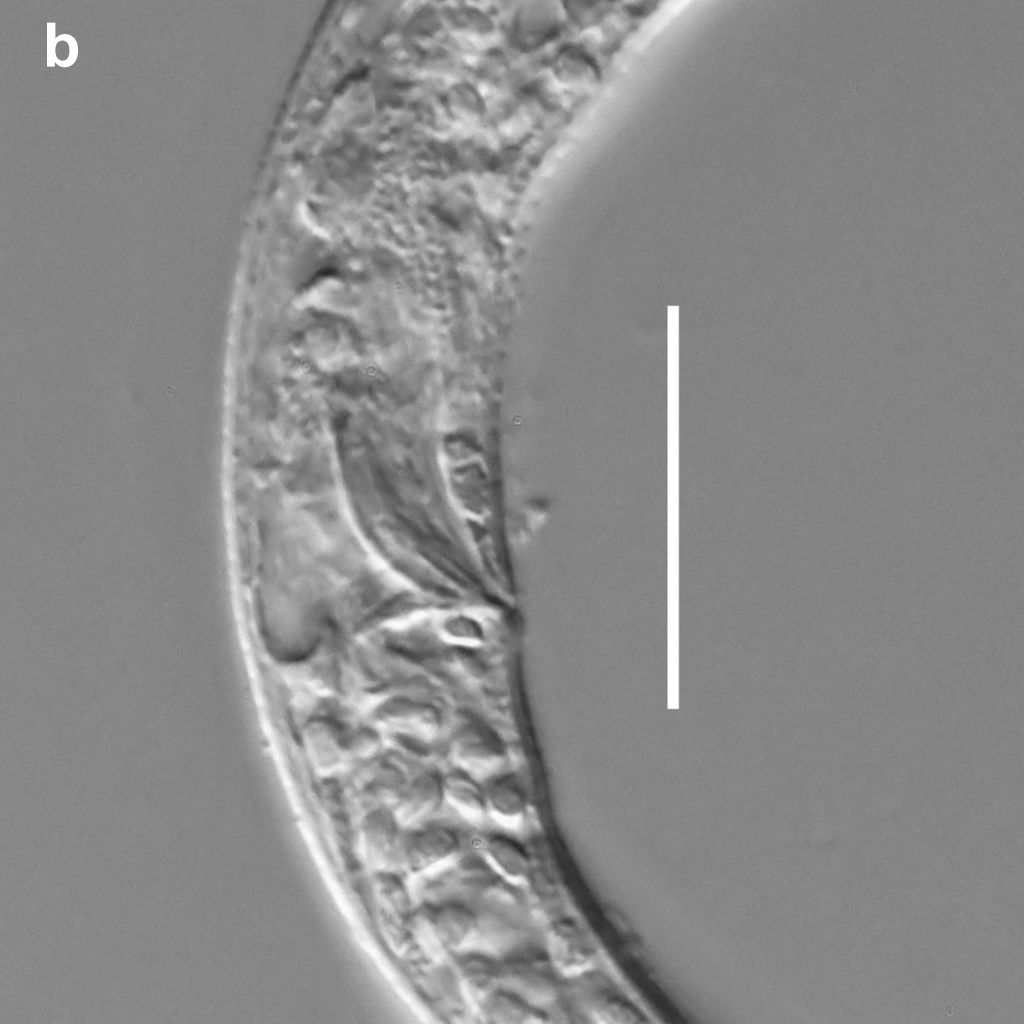
male cloacal region showing gubernaculum

**Figure 3c.**
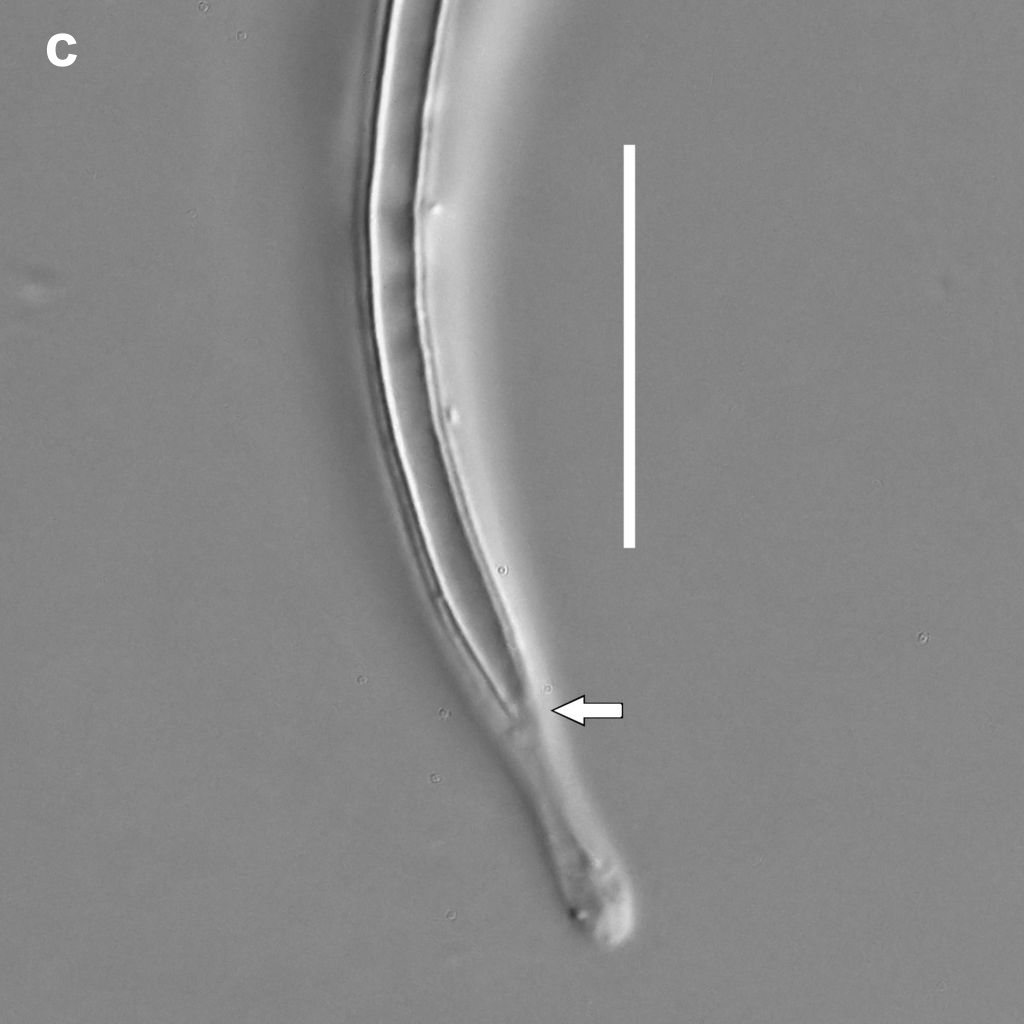
caudal region, surface view showing posterior end of amphid and lateral alae (arrow)

**Figure 3d.**
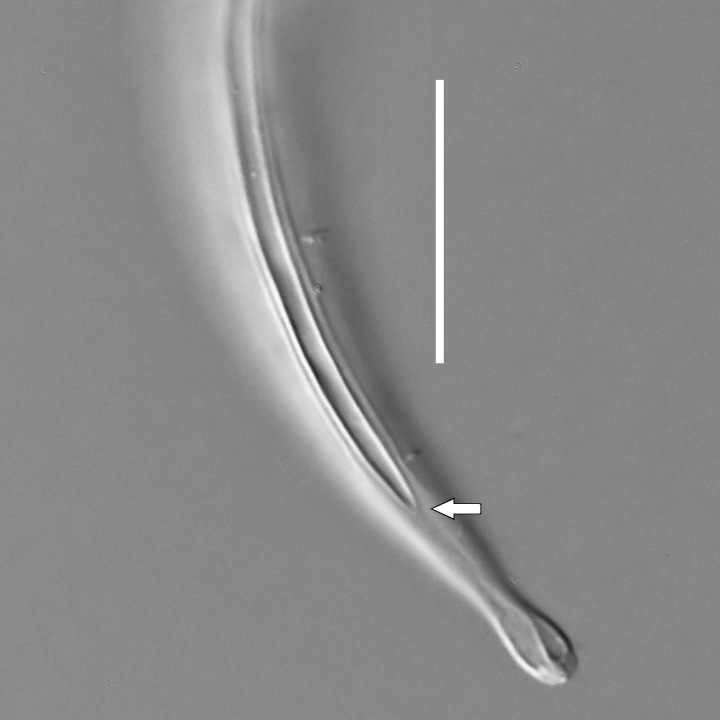
caudal region, surface view showing posterior end of amphid and lateral alae (arrow)

**Figure 5a.**
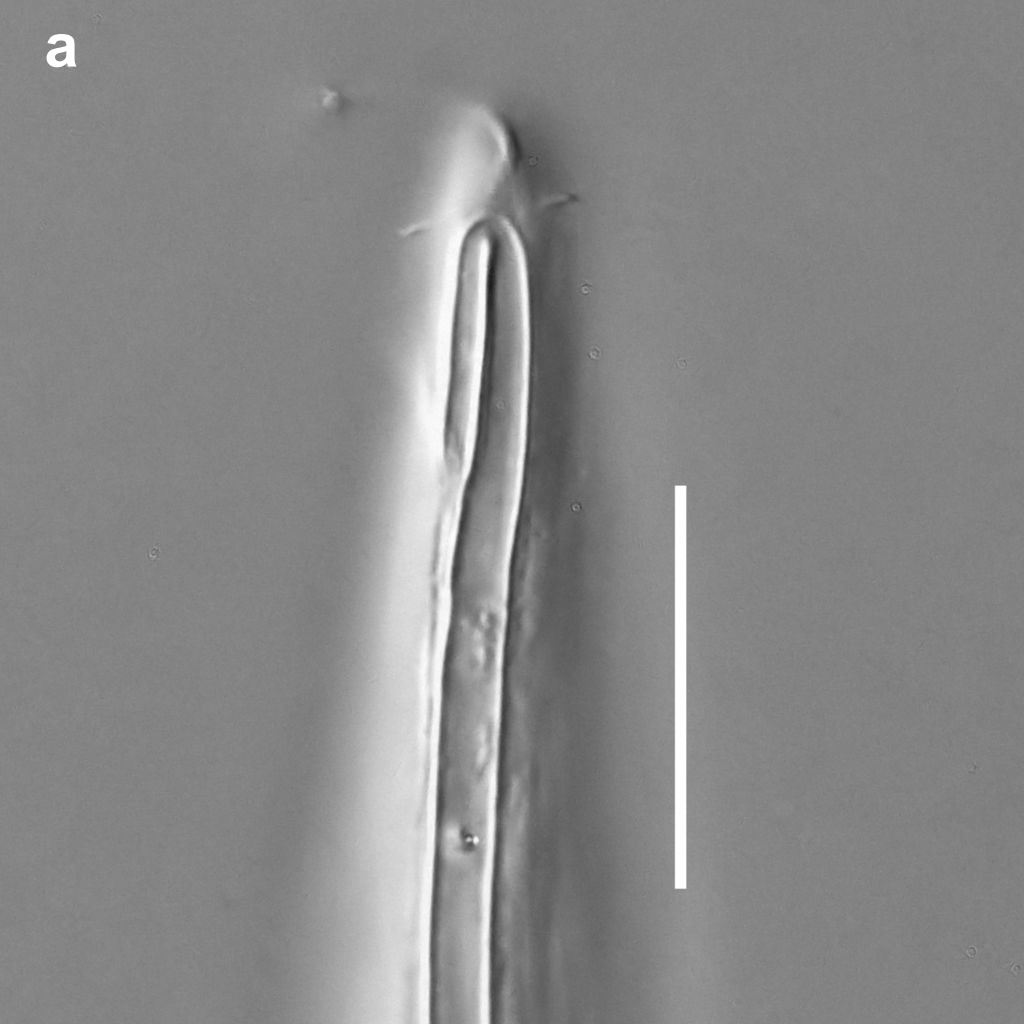
anterior end, surface view showing amphid (ventral side to the right)

**Figure 5b.**
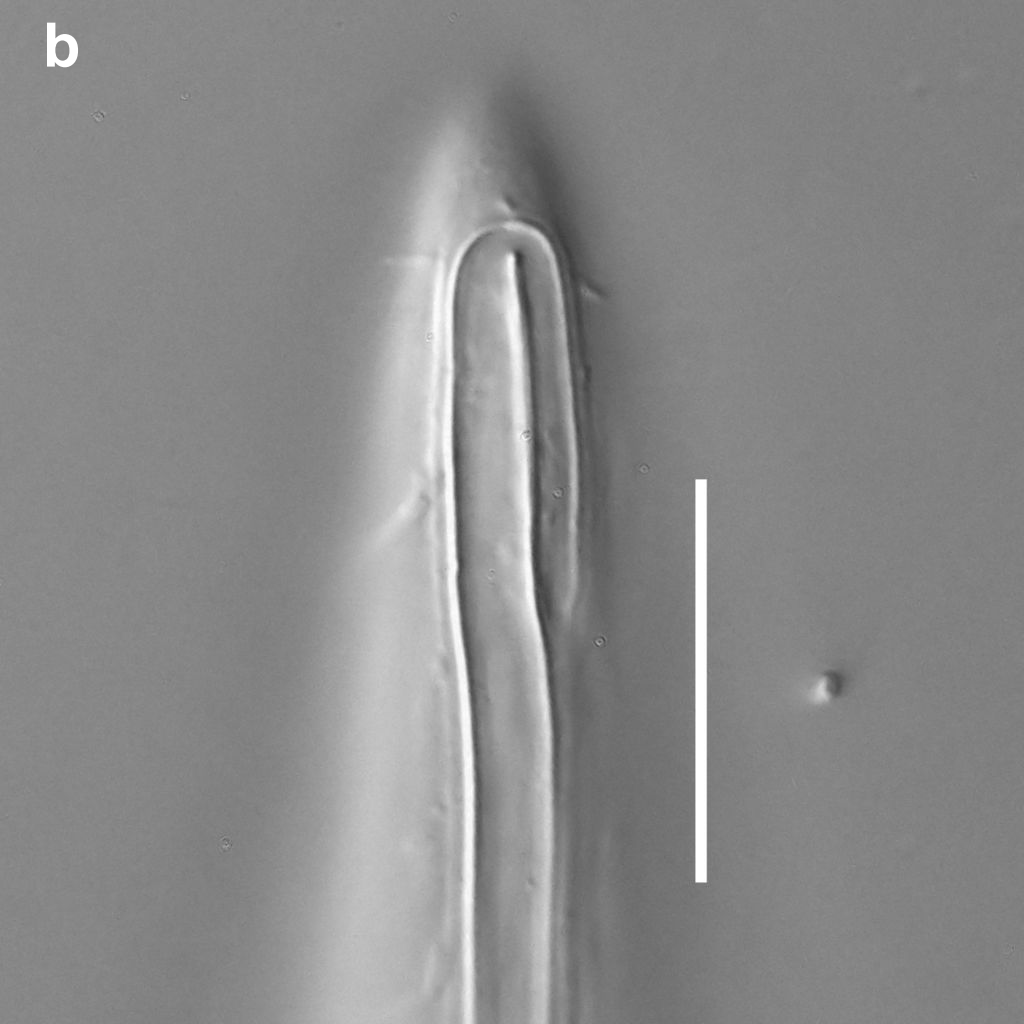
anterior end, surface view showing amphid (ventral side to the left)

**Figure 5c.**
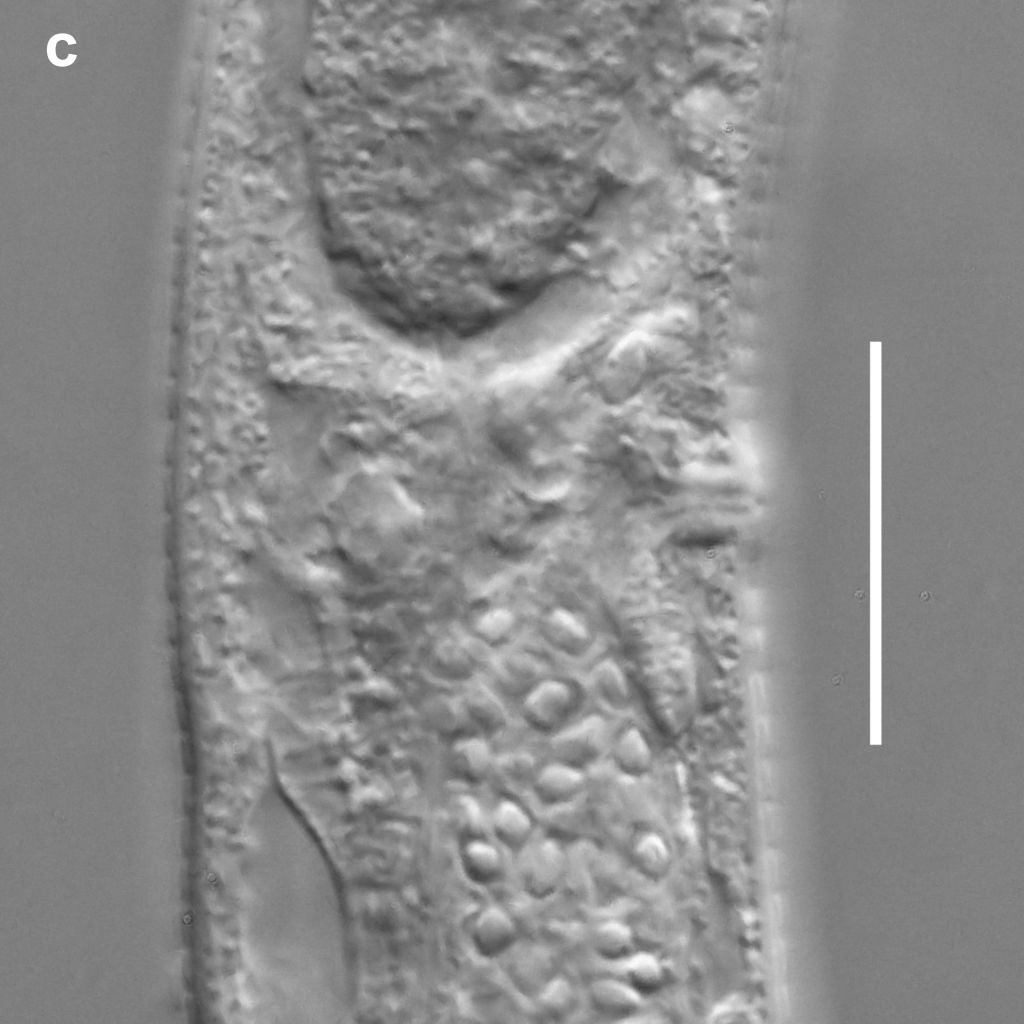
vulval region

**Figure 5d.**
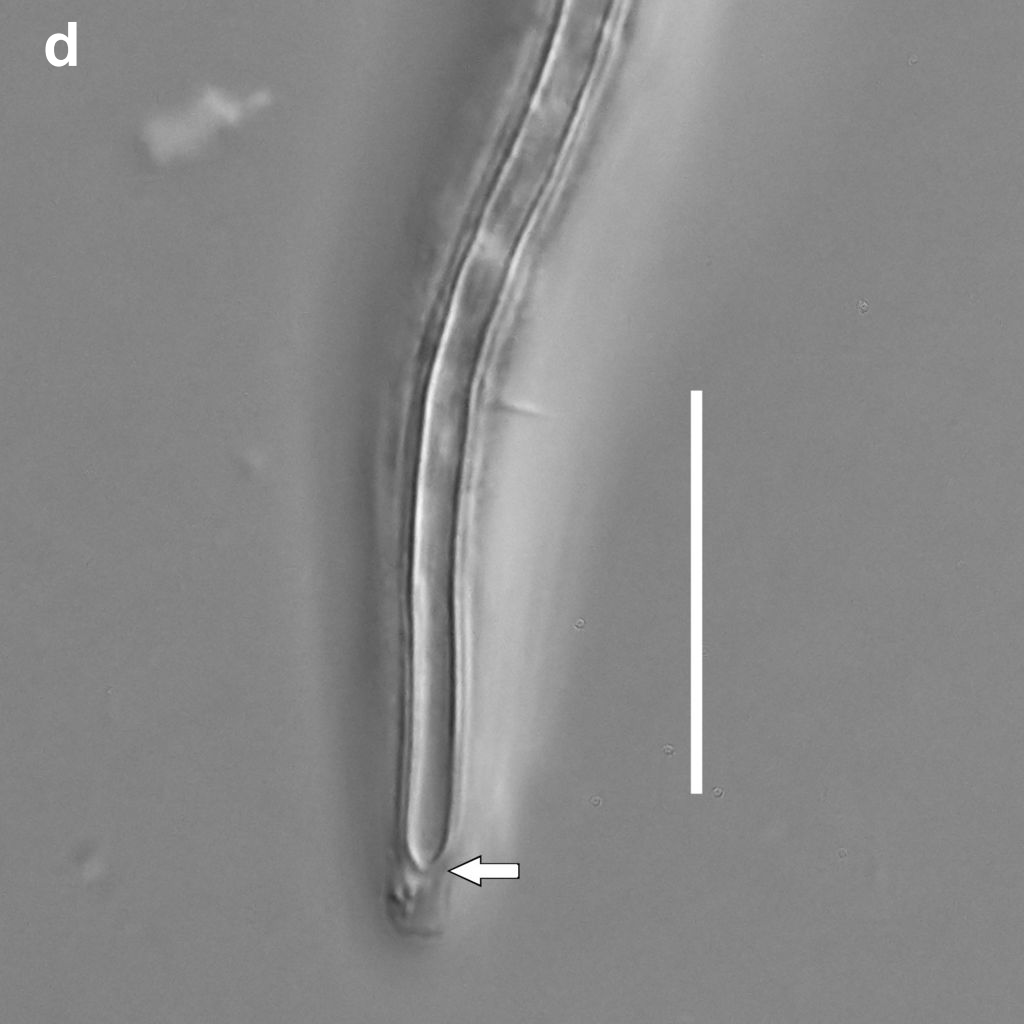
caudal region, surface view showing posterior end of amphid and lateral alae (arrow)

**Figure 7a.**
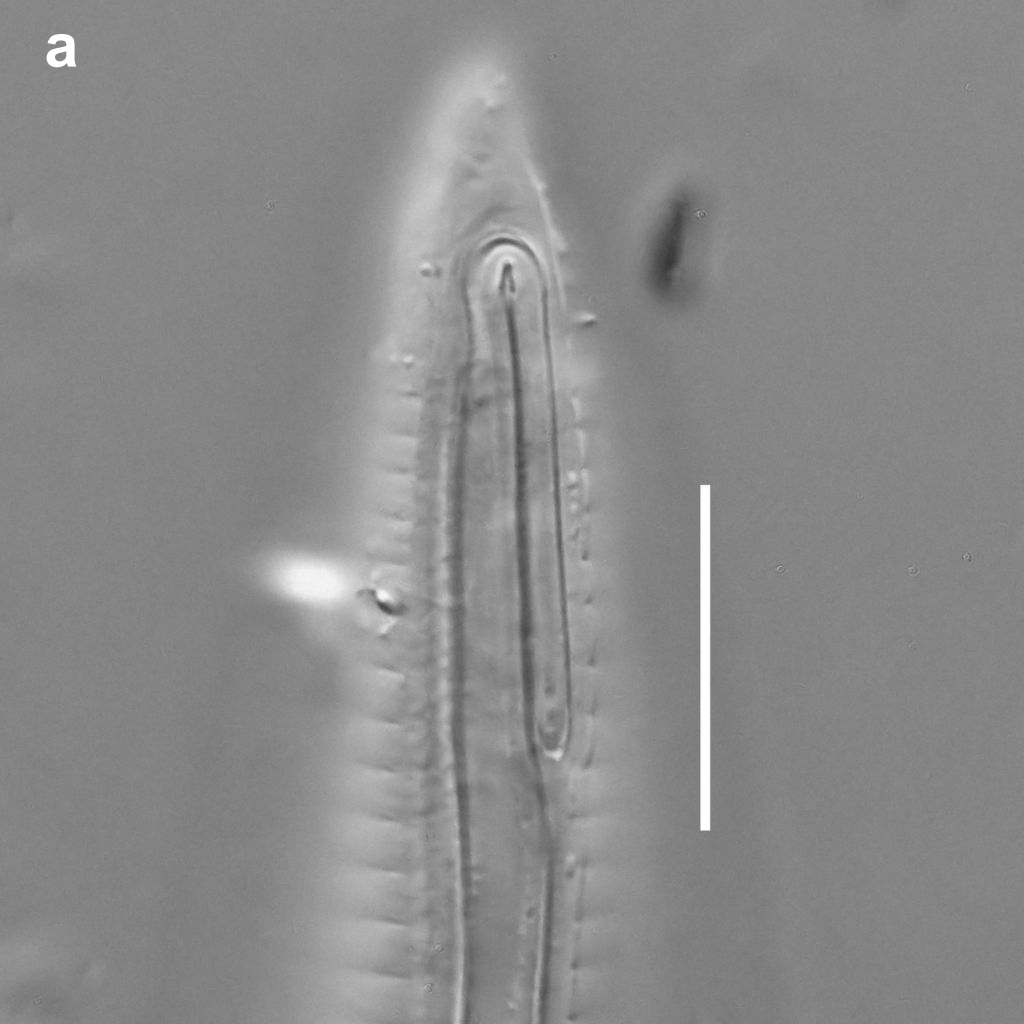
anterior end, surface view showing anterior part of amphid (ventral side to the left)

**Figure 7b.**
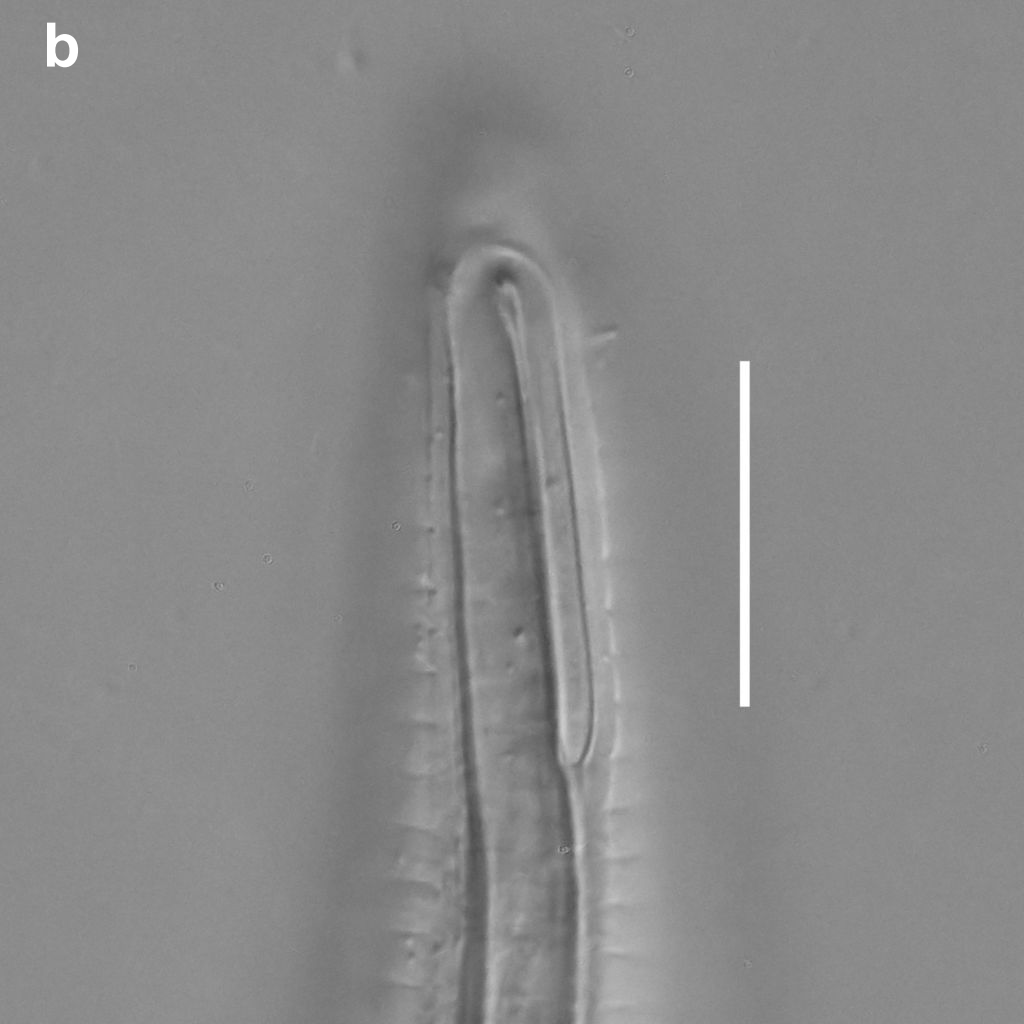
anterior end, surface view showing anterior part of amphid (ventral side to the left)

**Figure 7c.**
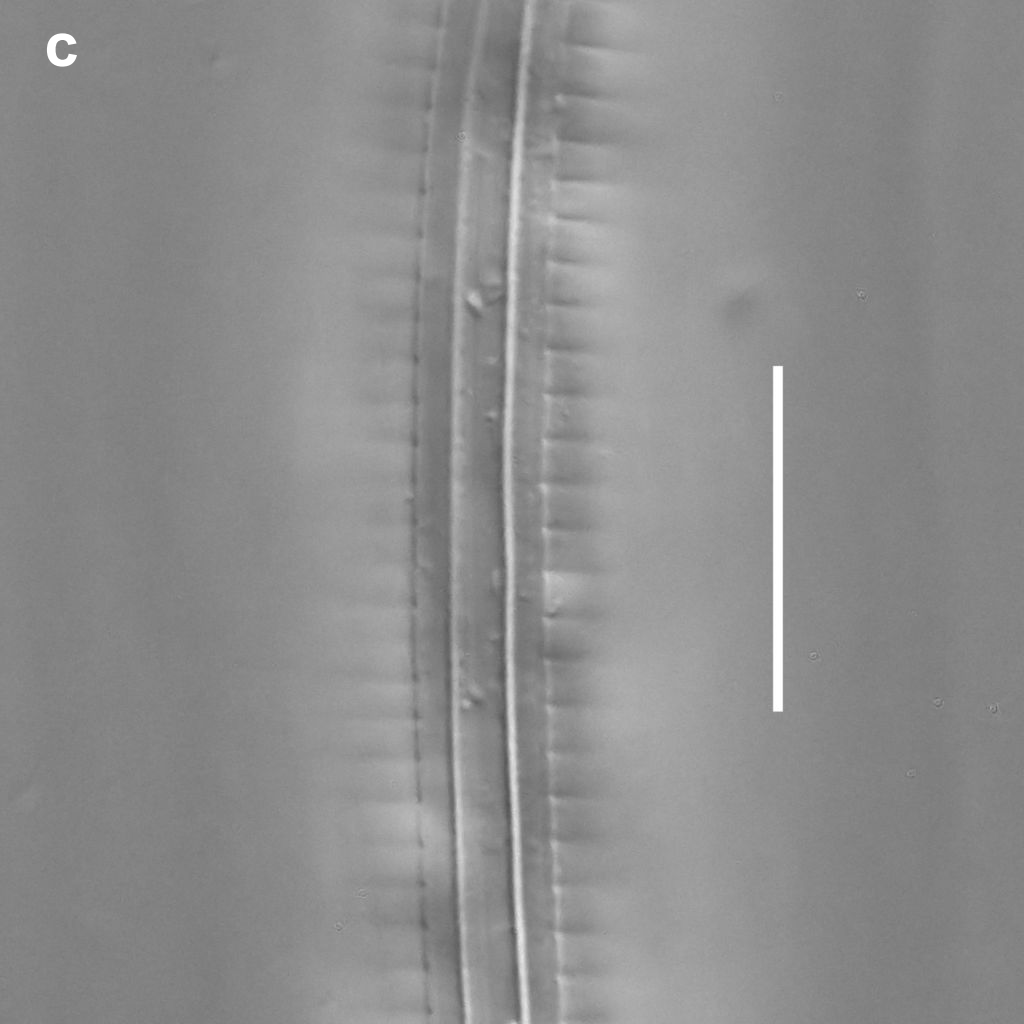
lateral alae at mid-body, surface view

**Figure 7d.**
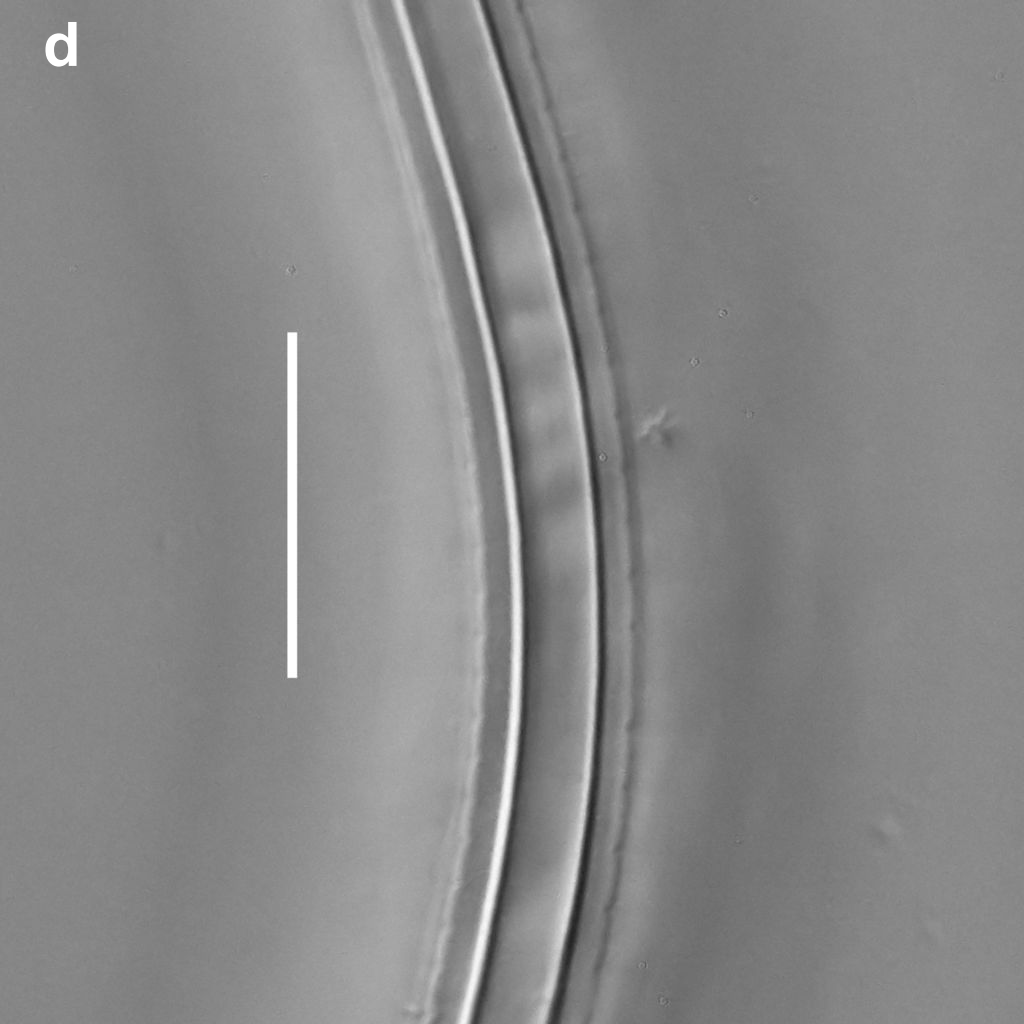
lateral alae at mid-body, surface view

**Figure 7e.**
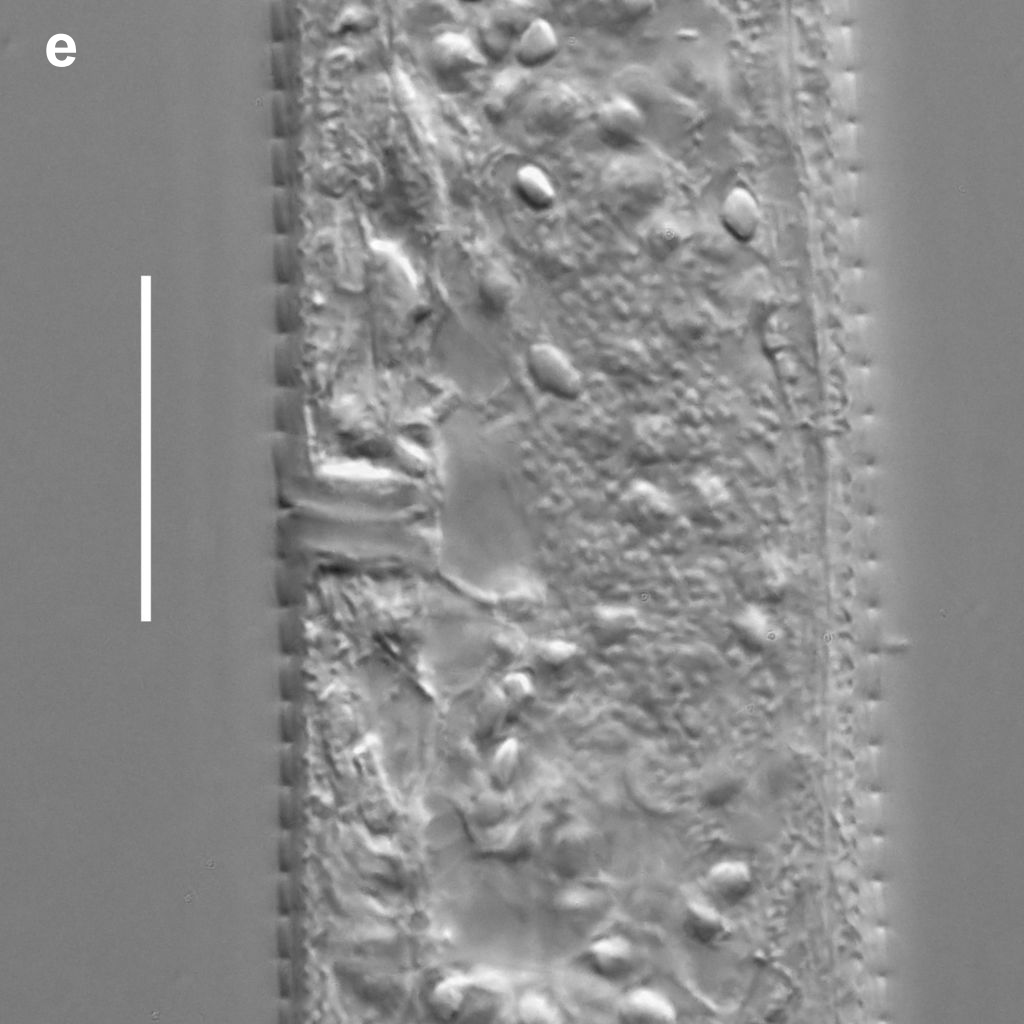
vulval region

**Figure 7f.**
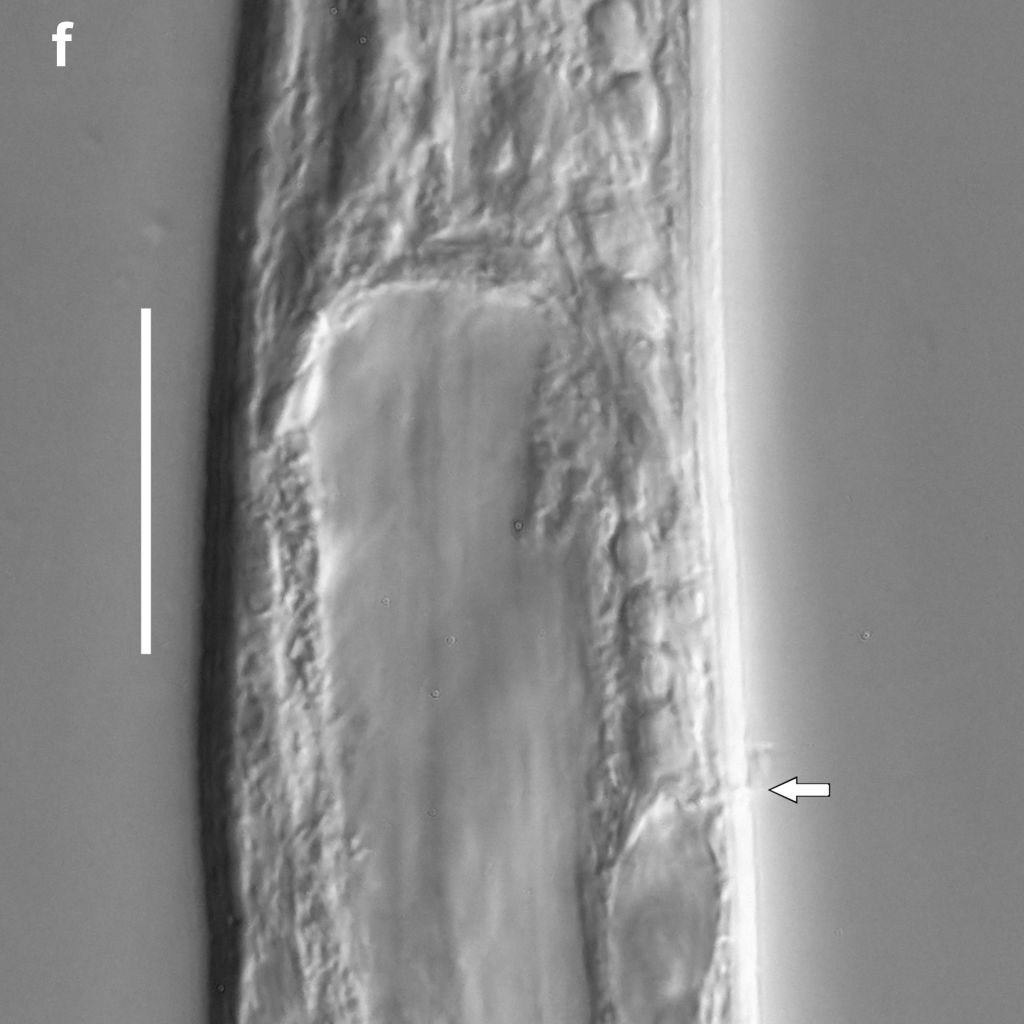
secretory-excretory pore at level with anterior part of intestine (arrow)

**Figure 9a.**
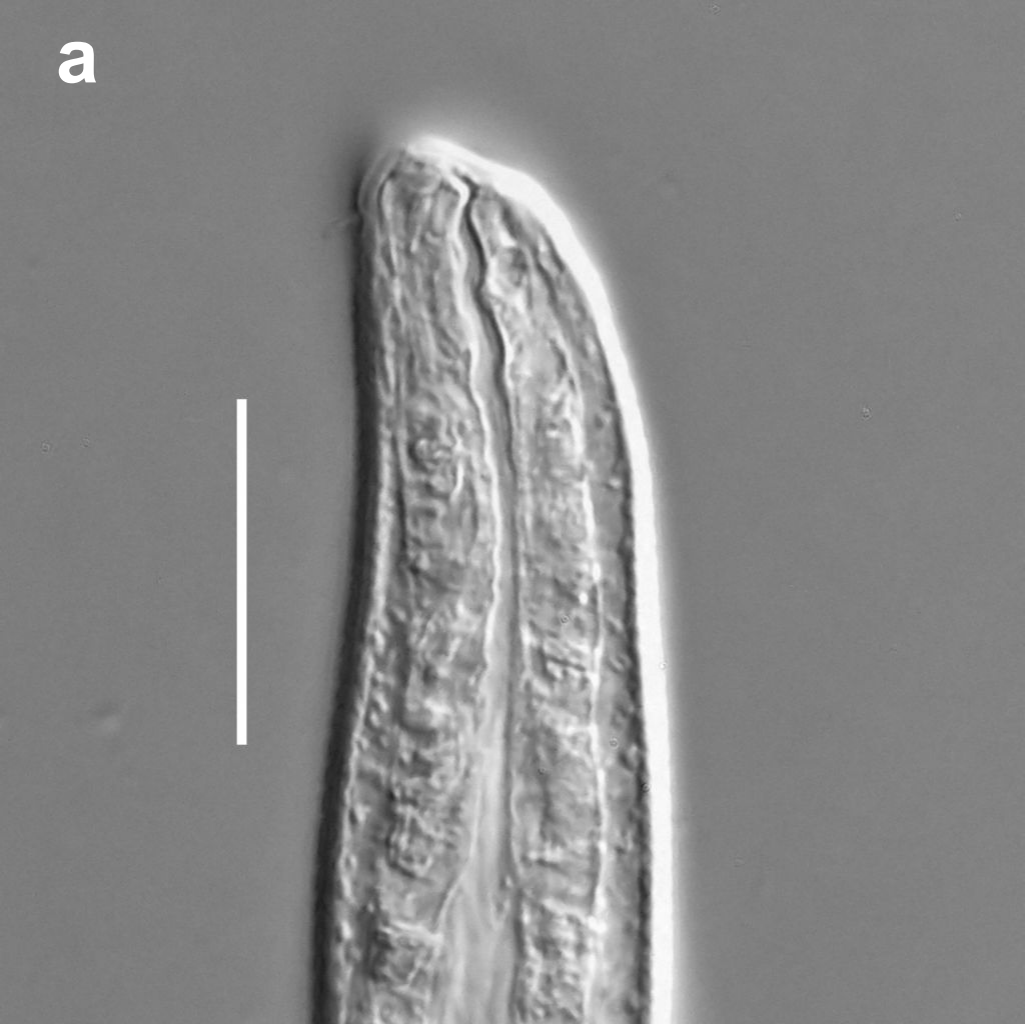
anterior end, median section (ventral side to the left)

**Figure 9b.**
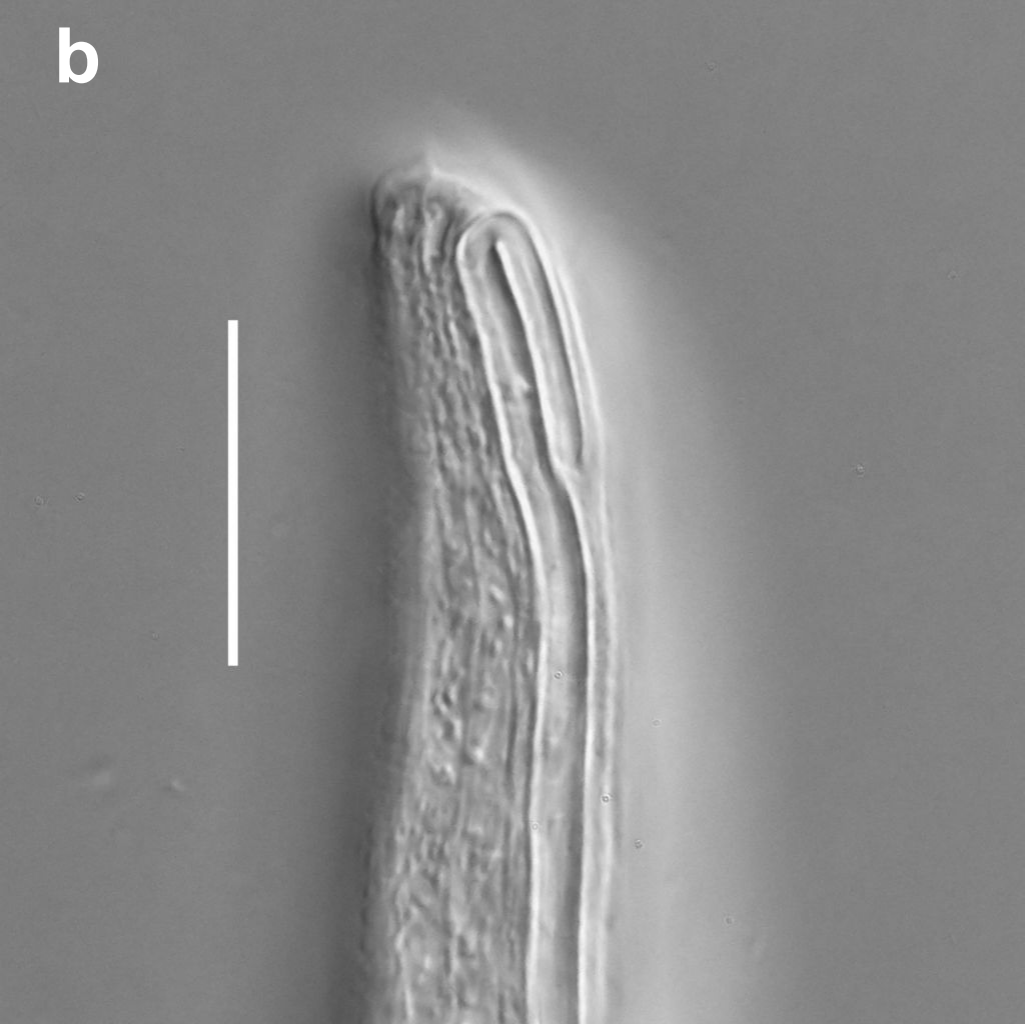
anterior end, surface view showing anterior part of amphid (ventral side to the left)

**Figure 9c.**
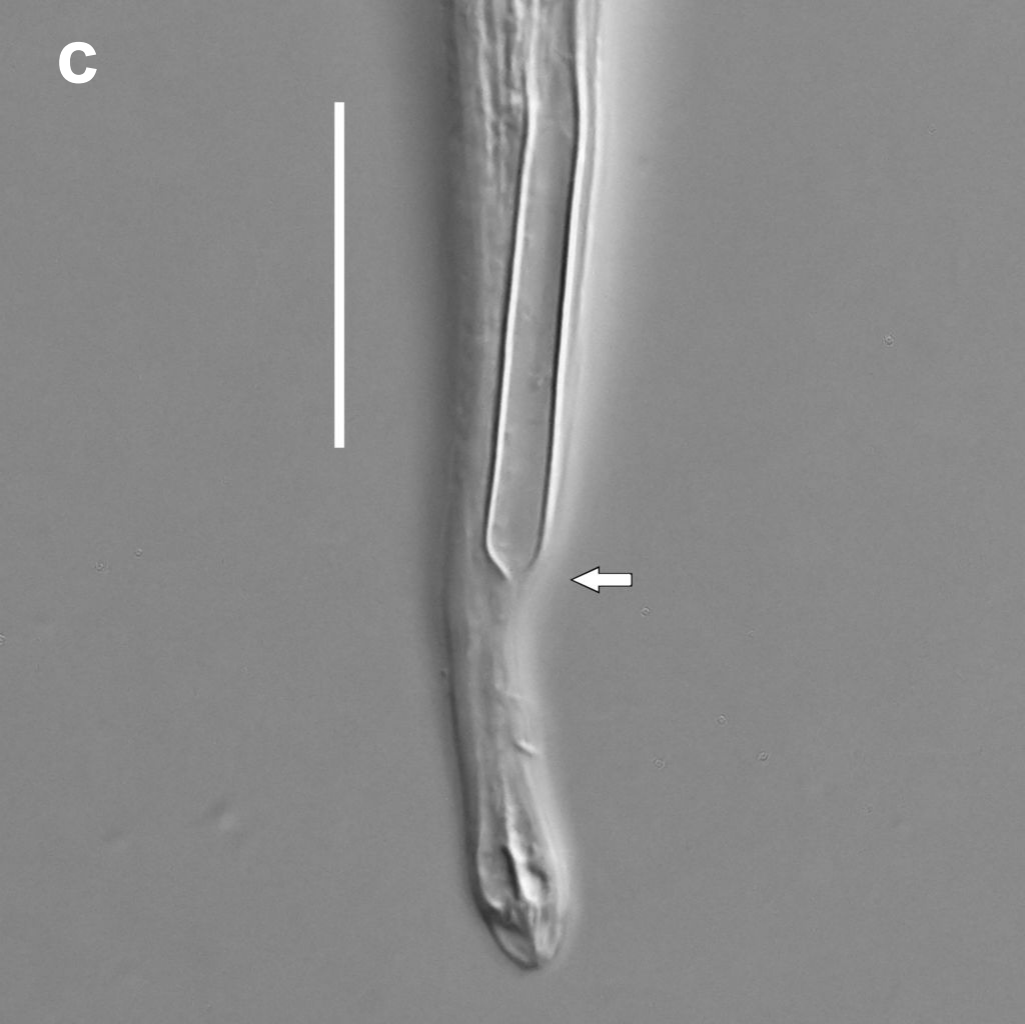
caudal region, surface view showing posterior end of amphid and lateral alae (arrow)

**Figure 10a.**
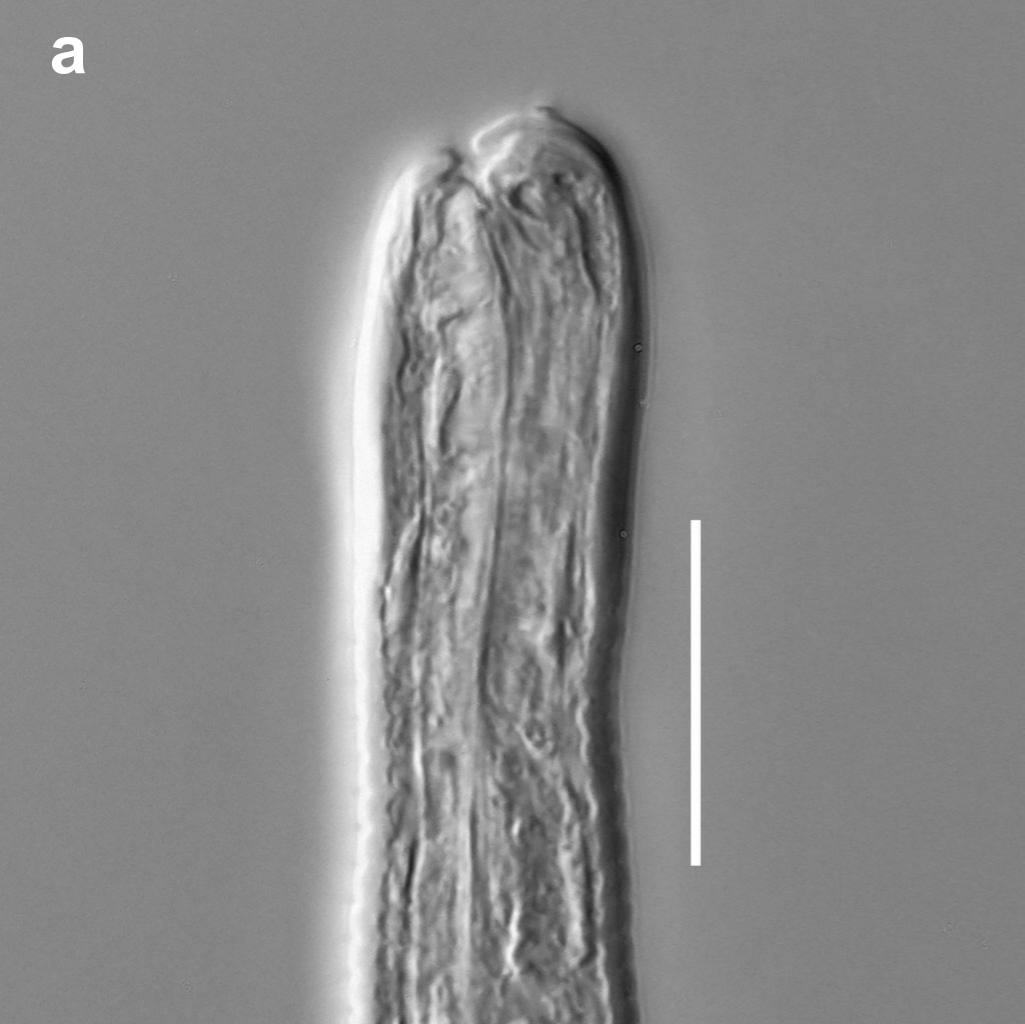
anterior end, median section (ventral side to the right)

**Figure 10b.**
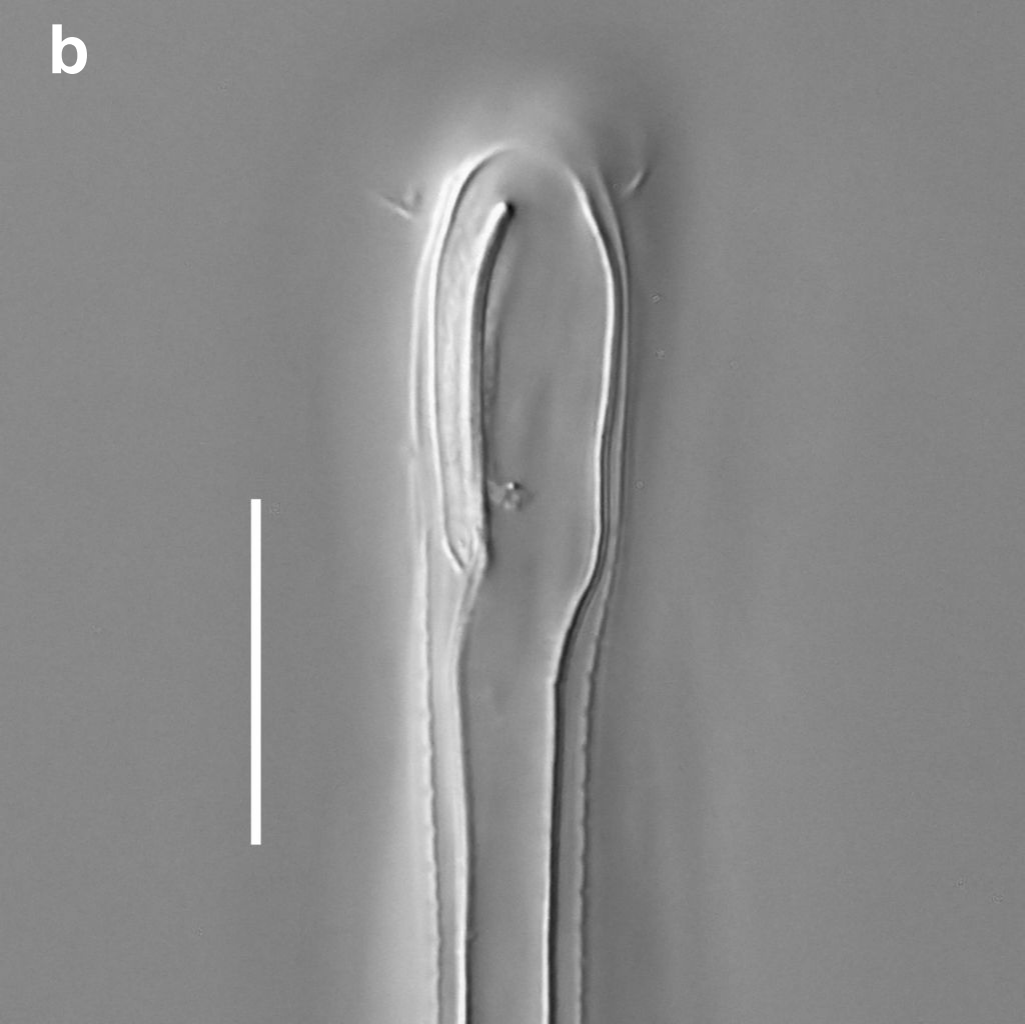
anterior end, surface view showing anterior part of amphid (ventral side to the right)

**Figure 10c.**
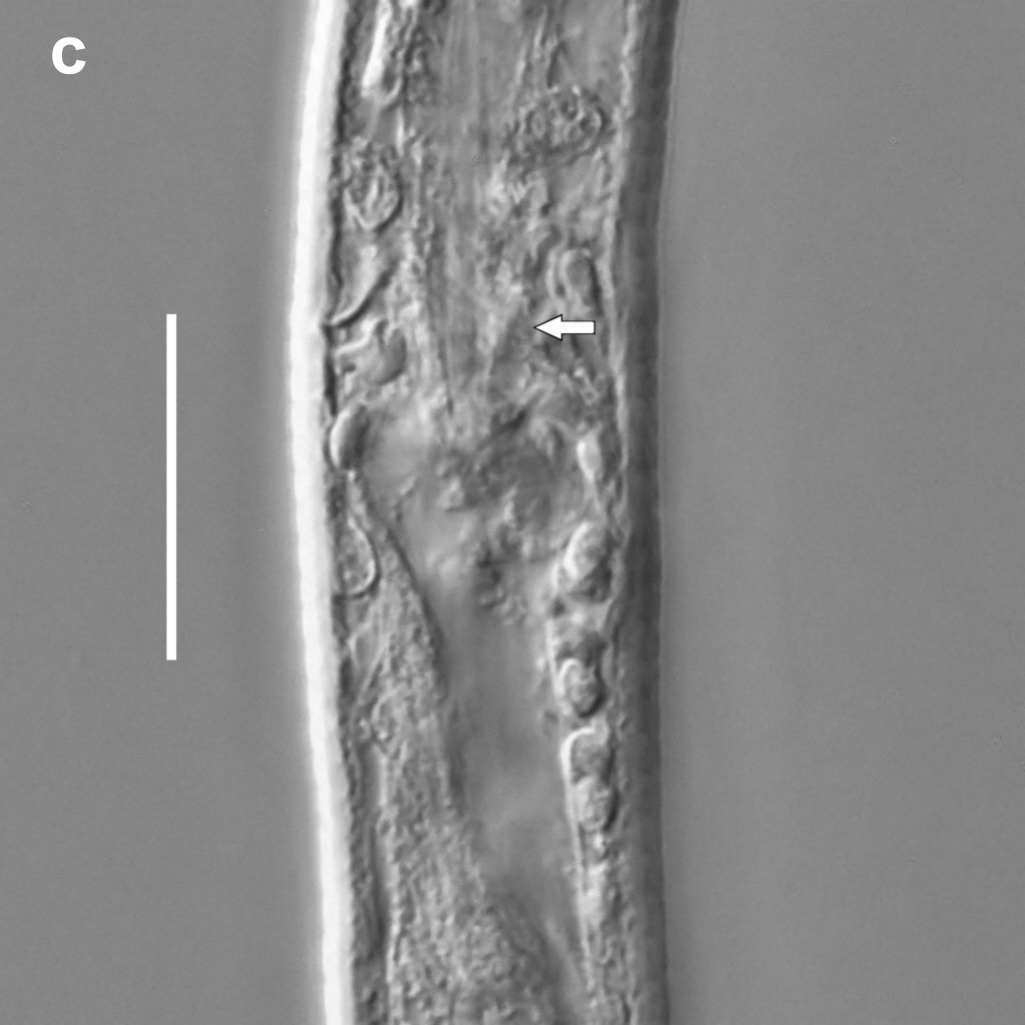
nerve ring surrounding pharyngo-intestinal junction (arrow)

**Figure 10d.**
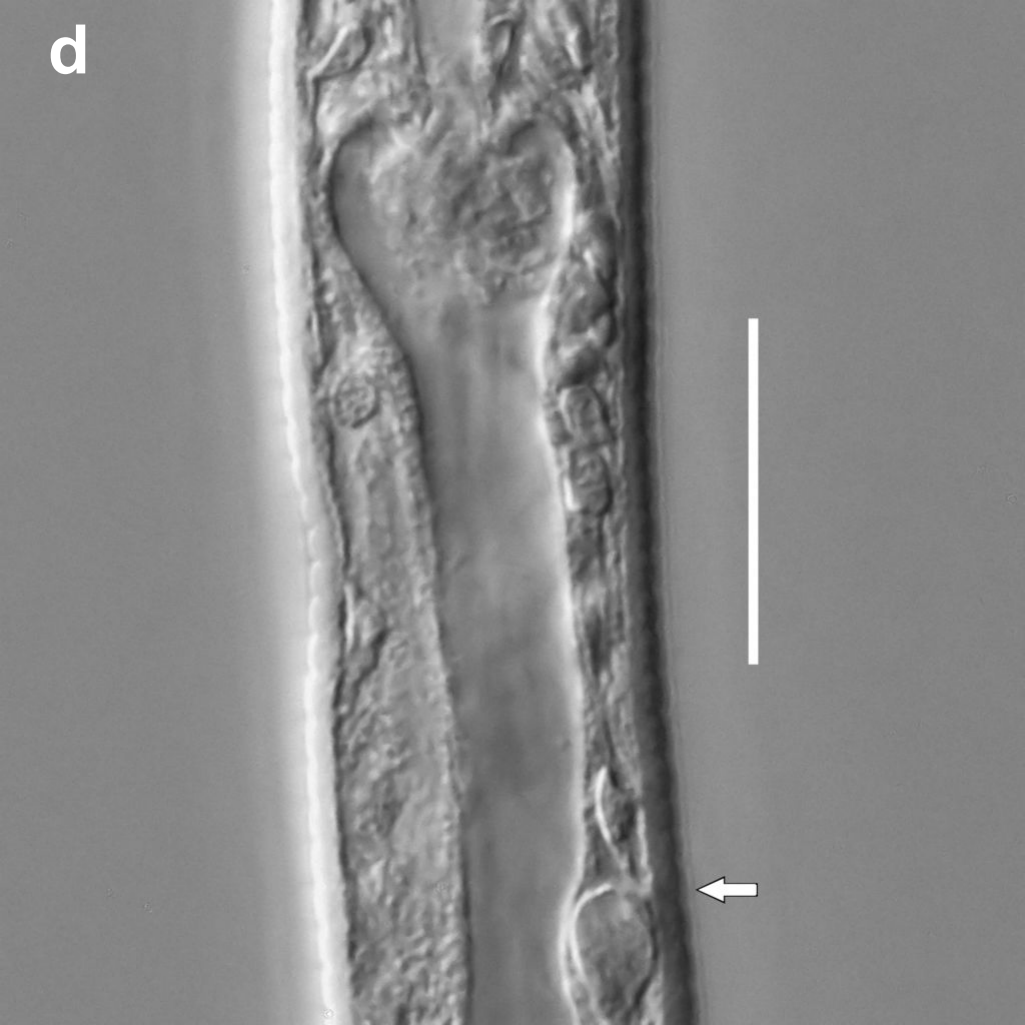
secretory-excretory pore at level with anterior part of intestine (arrow)

**Figure 10e.**
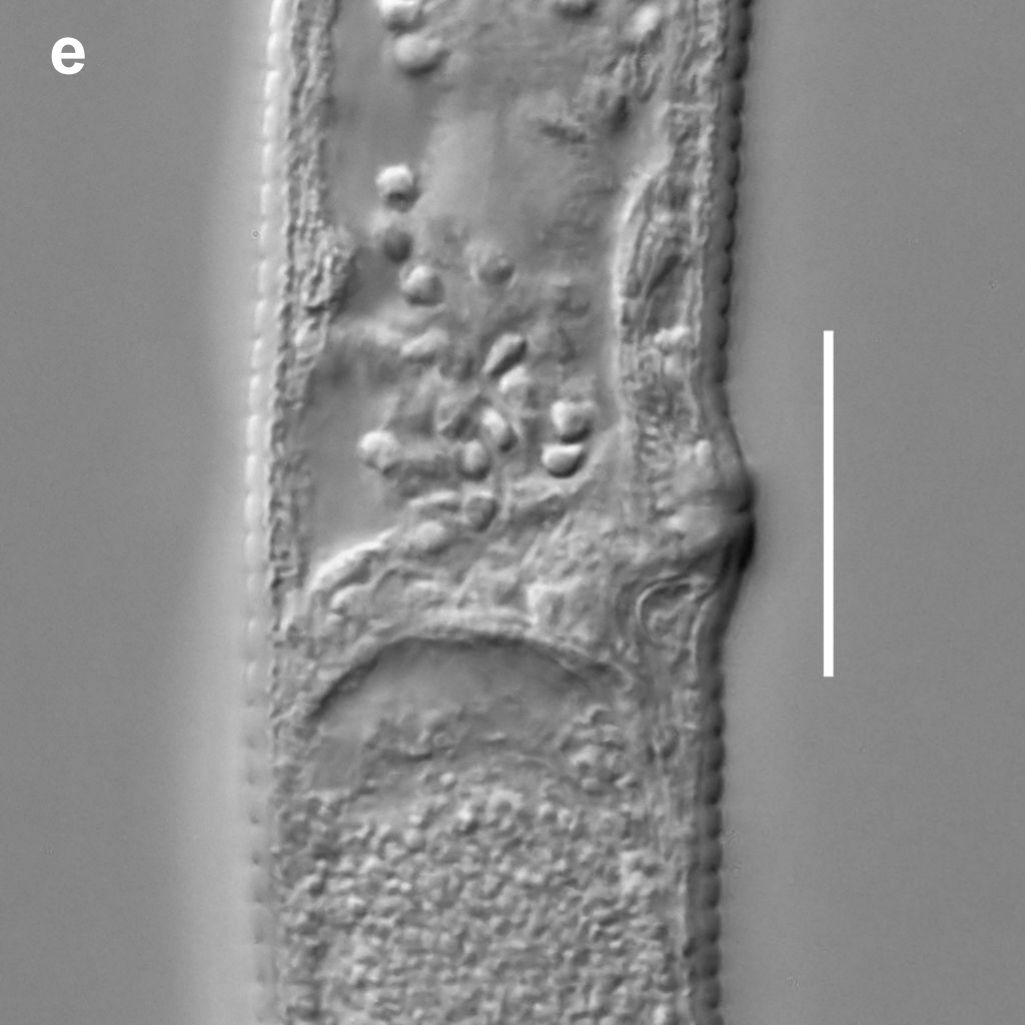
vulval region

**Figure 10f.**
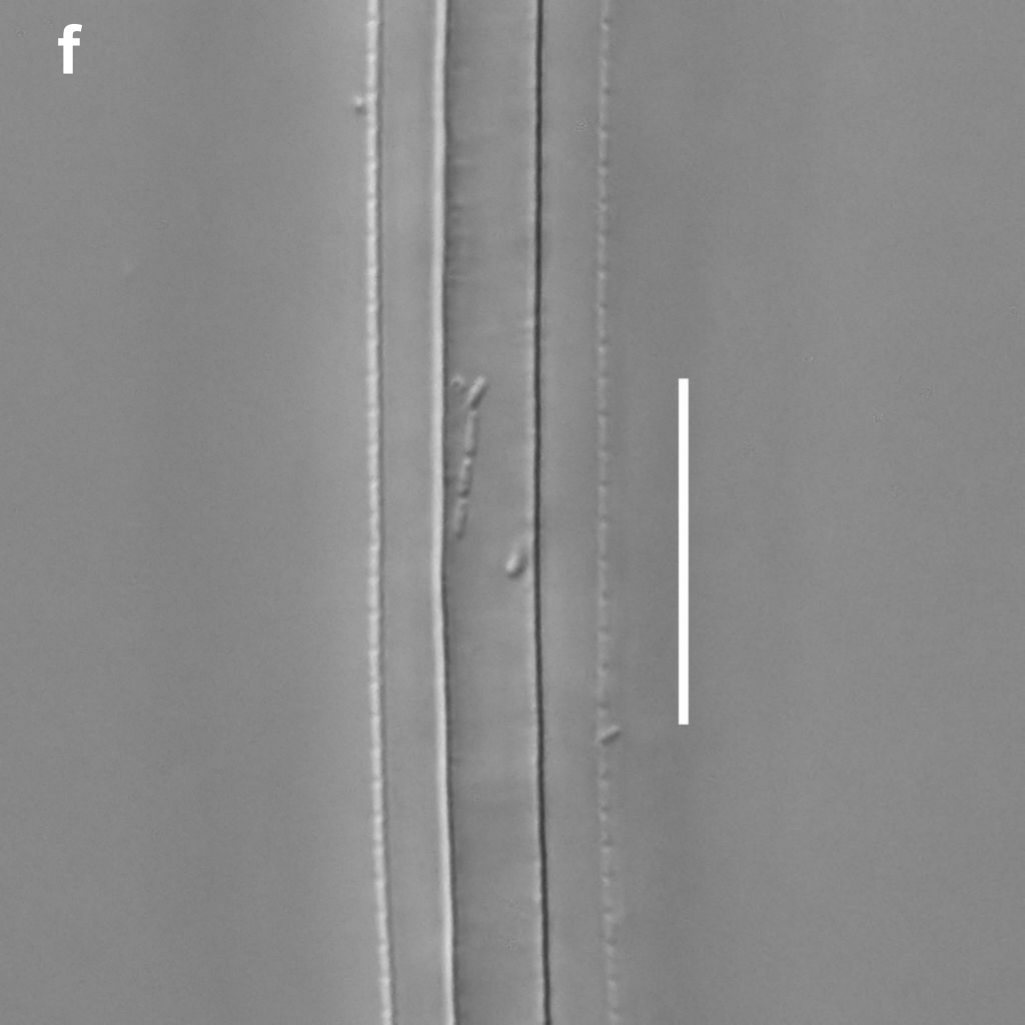
lateral alae at mid-body, surface view

**Figure 12a.**
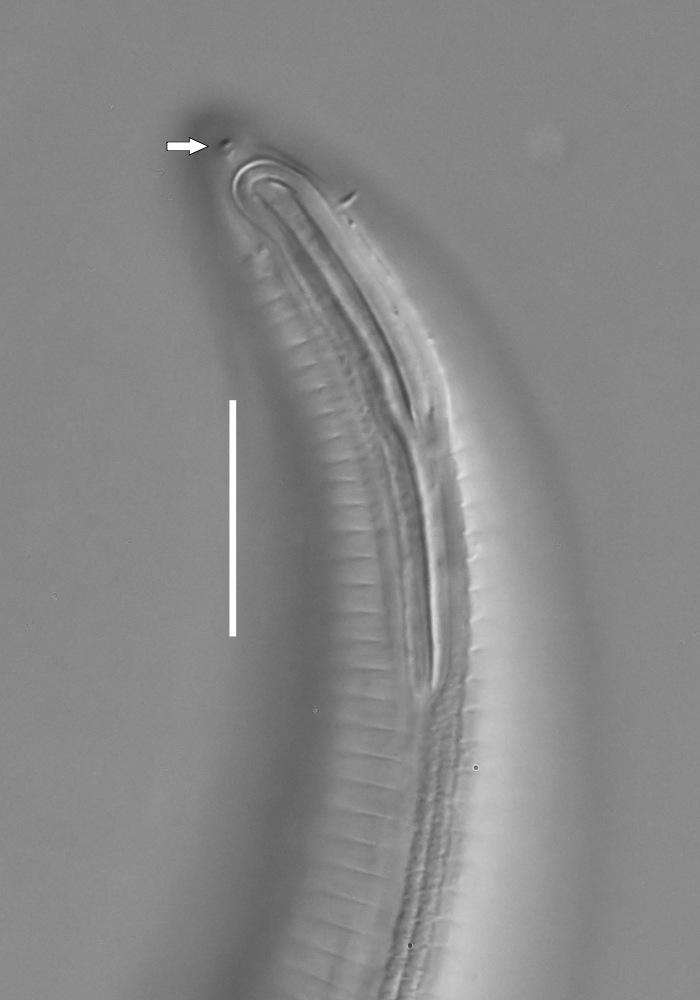
anterior end of a female, surface view showing amphid (ventral side to the left)

**Figure 12b.**
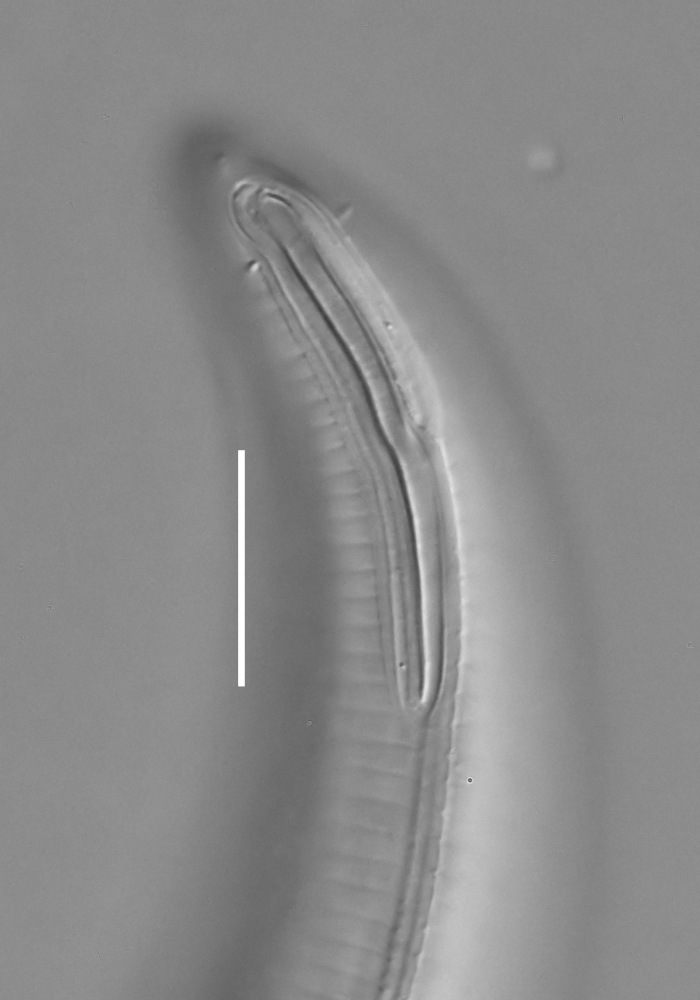
anterior end of a female, surface view showing amphid (ventral side to the left)

**Figure 12c.**
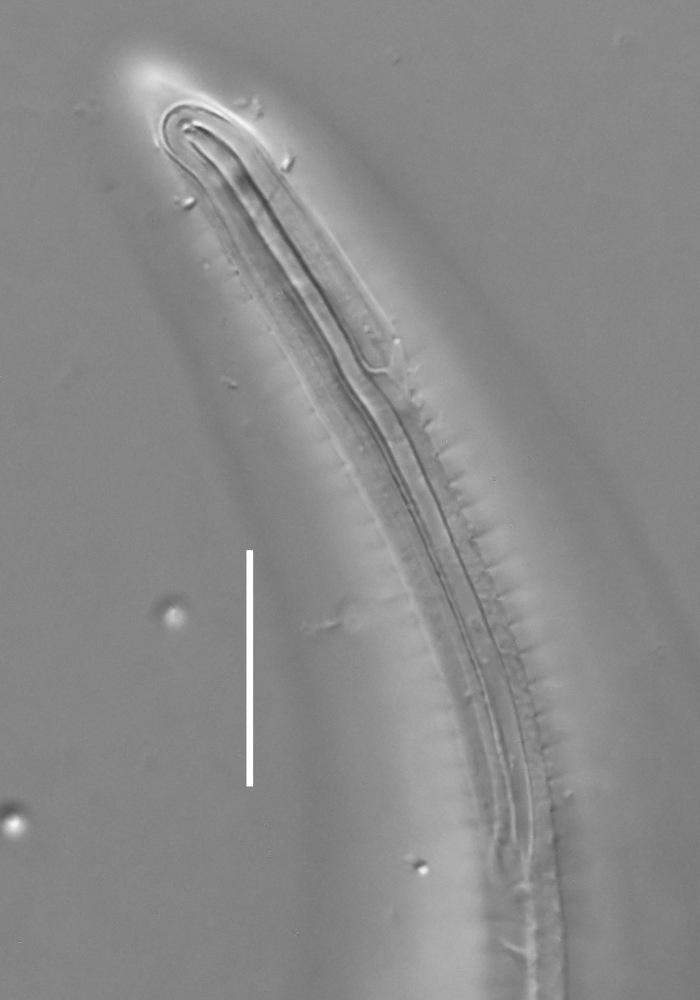
anterior end of a male, surface view showing amphid (ventral side to the left)

**Figure 12d.**
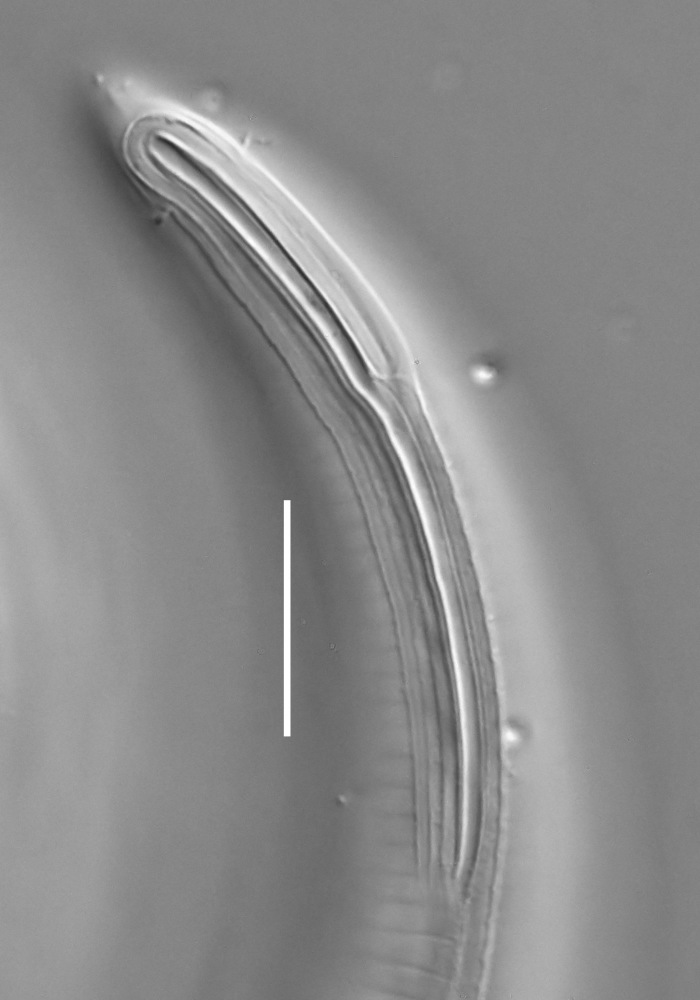
anterior end of a male, surface view showing amphid (ventral side to the left)

**Figure 13a.**
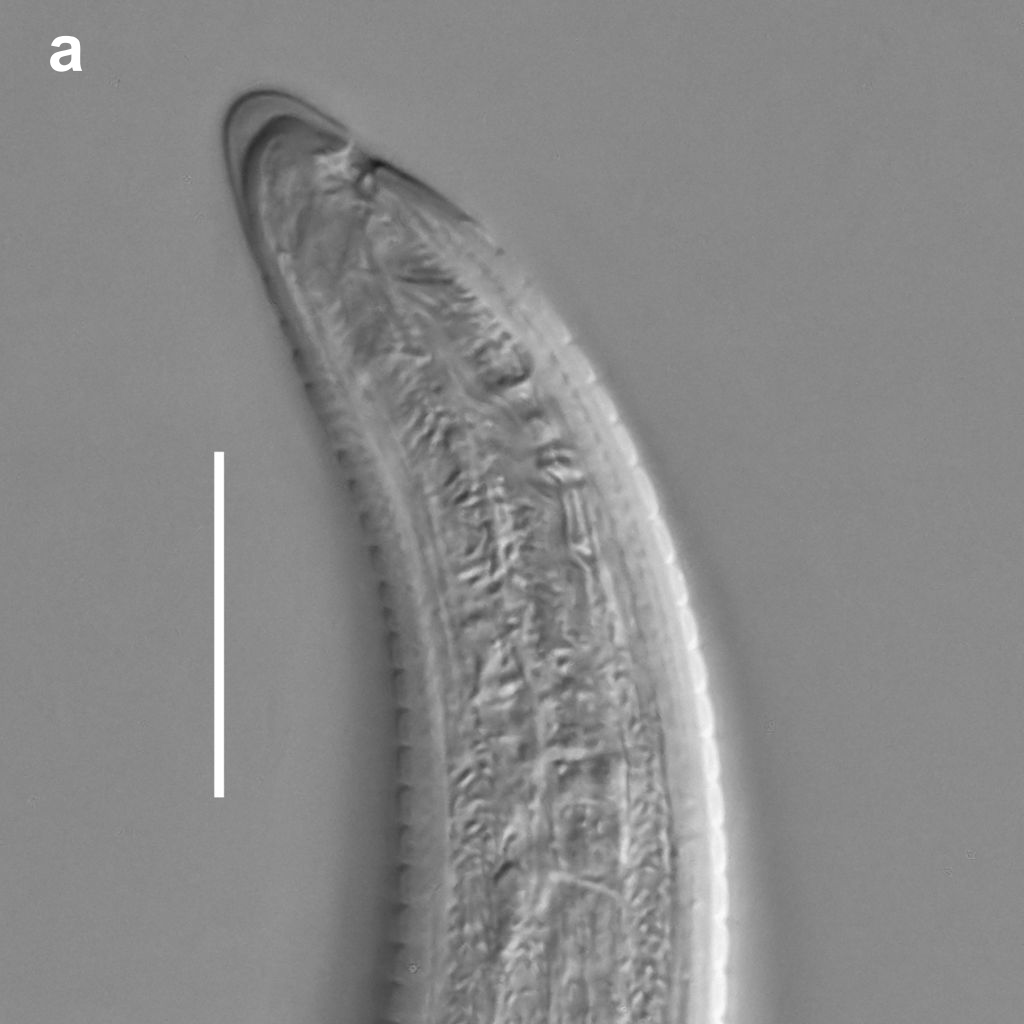
anterior end, median section (ventral side to the left)

**Figure 13b.**
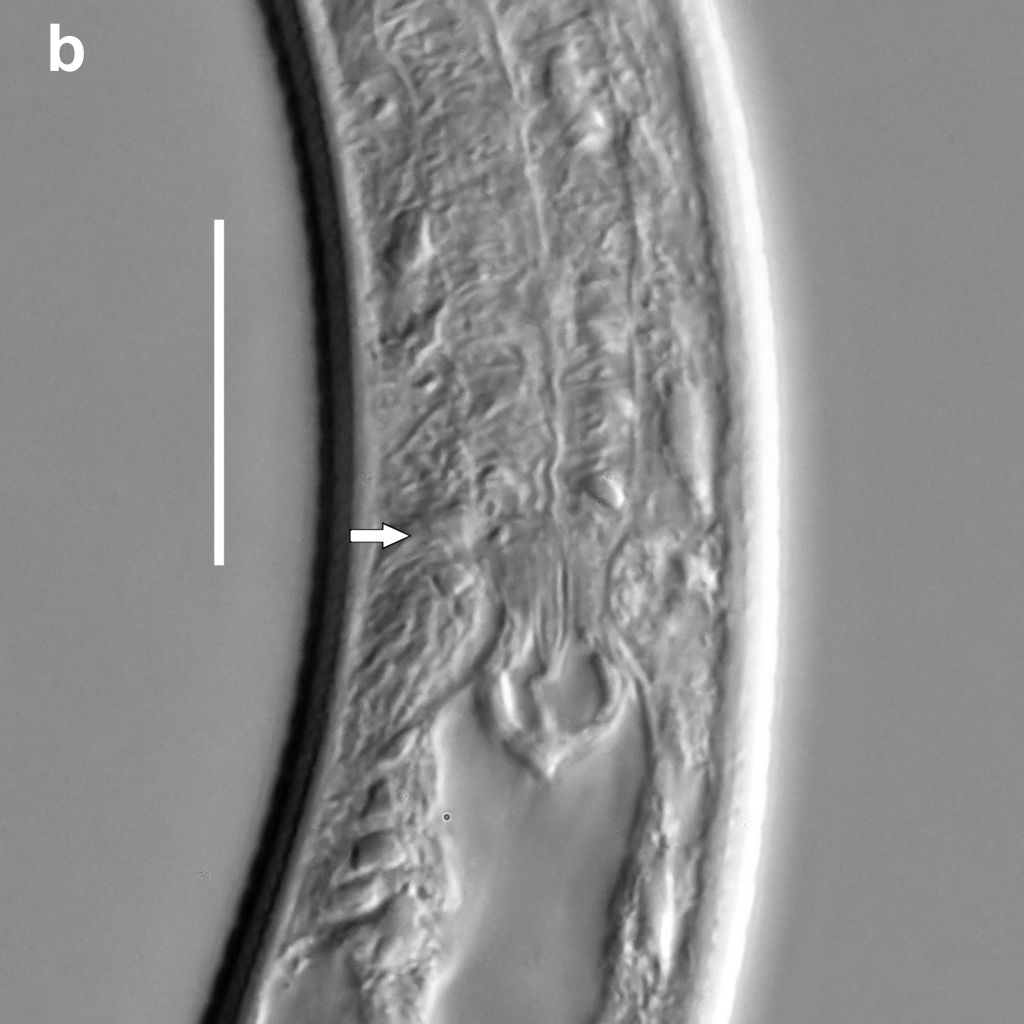
nerve ring surrounding pharyngo-intestinal junction (arrow)

**Figure 13c.**
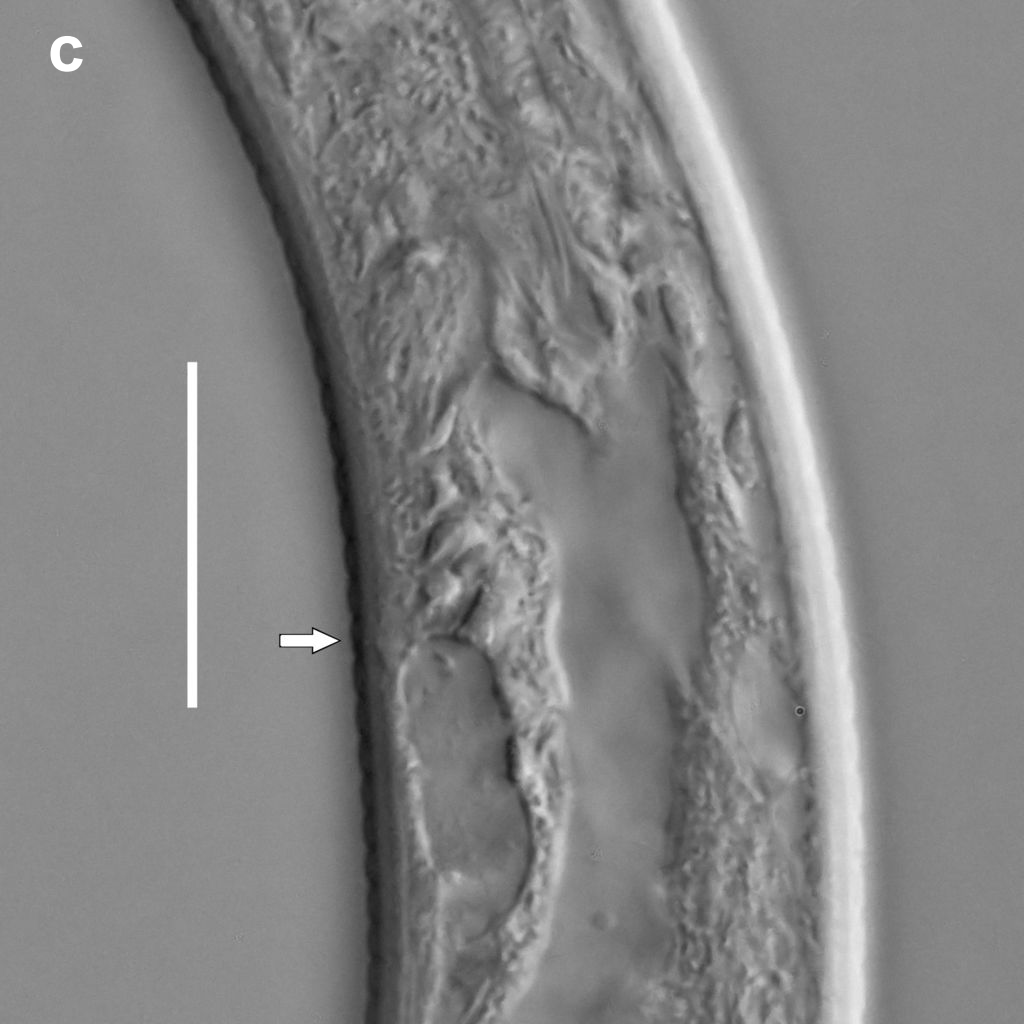
secretory-excretory pore at level with anterior part of intestine (arrow)

**Figure 13d.**
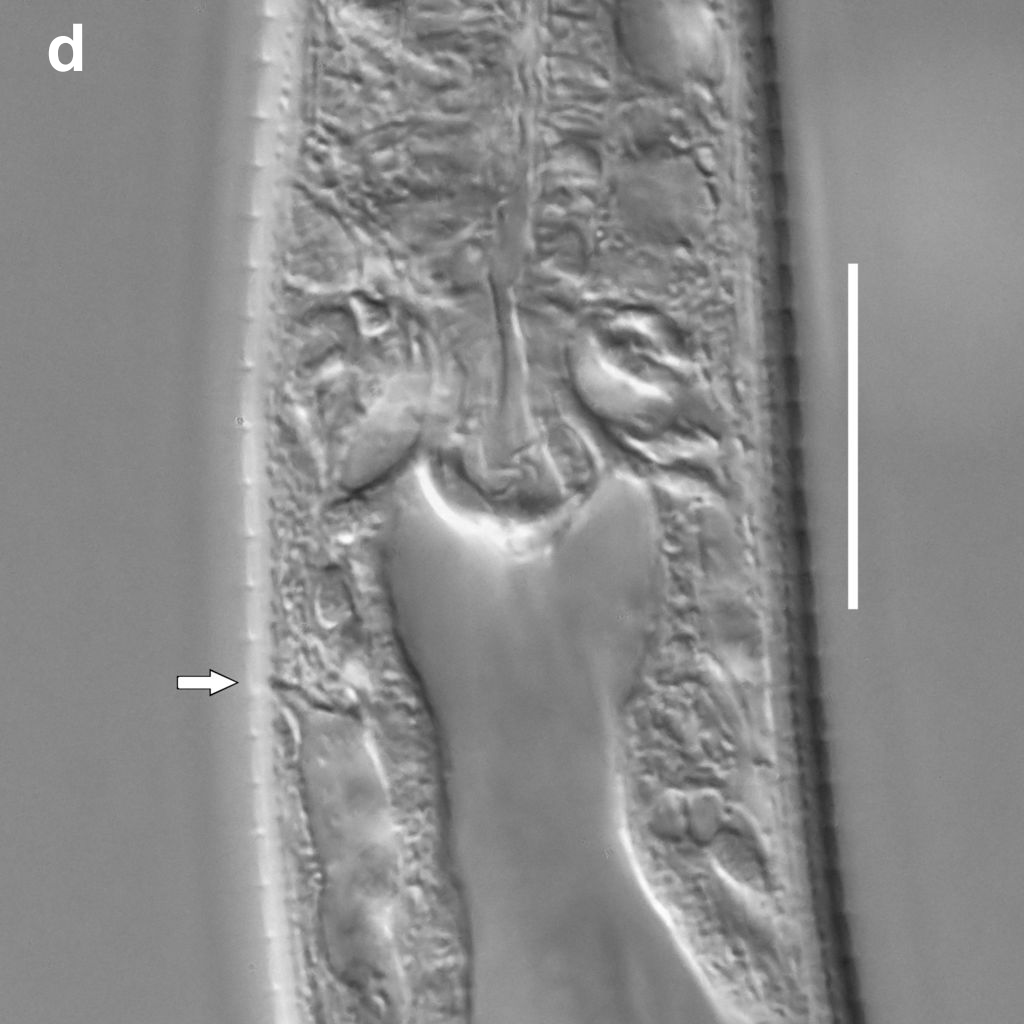
secretory-excretory pore at level with anterior part of intestine (arrow)

**Figure 13e.**
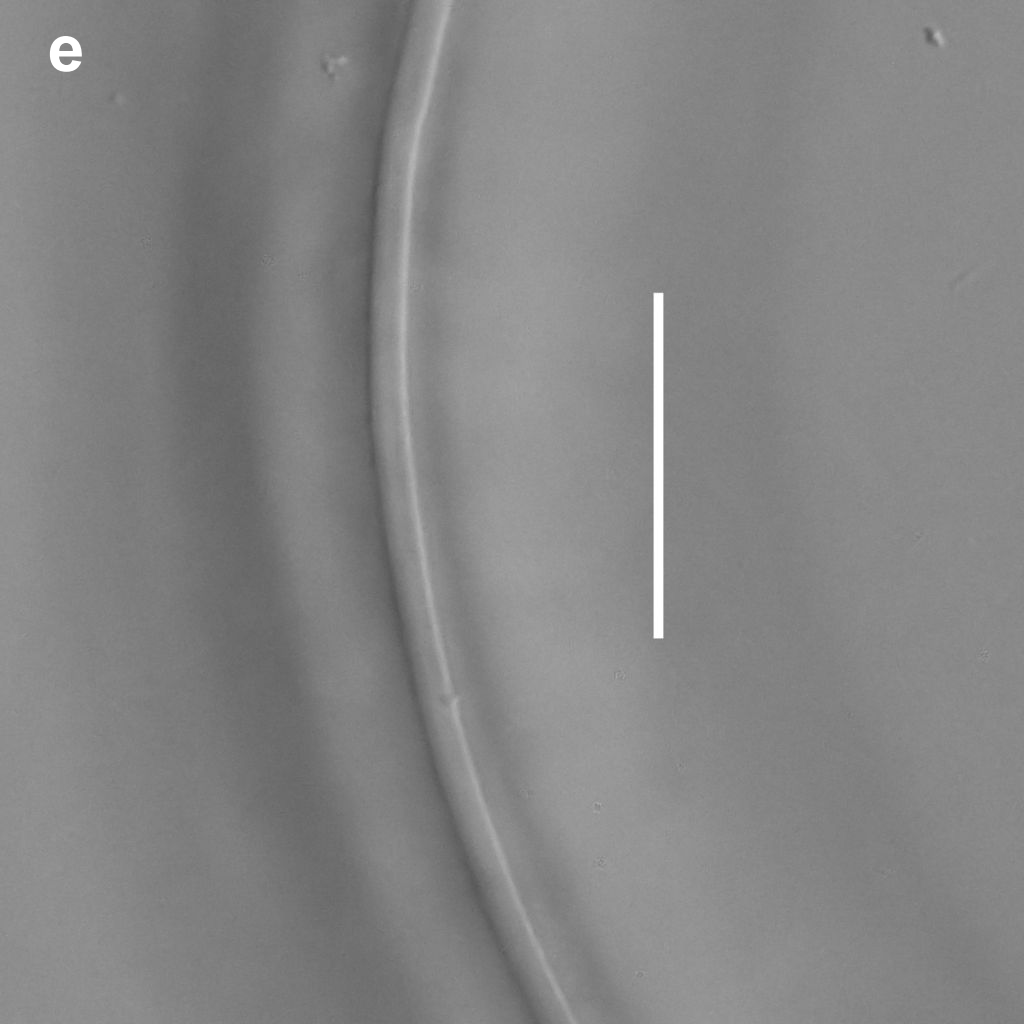
lateral alae at mid-body, surface view

**Figure 13f.**
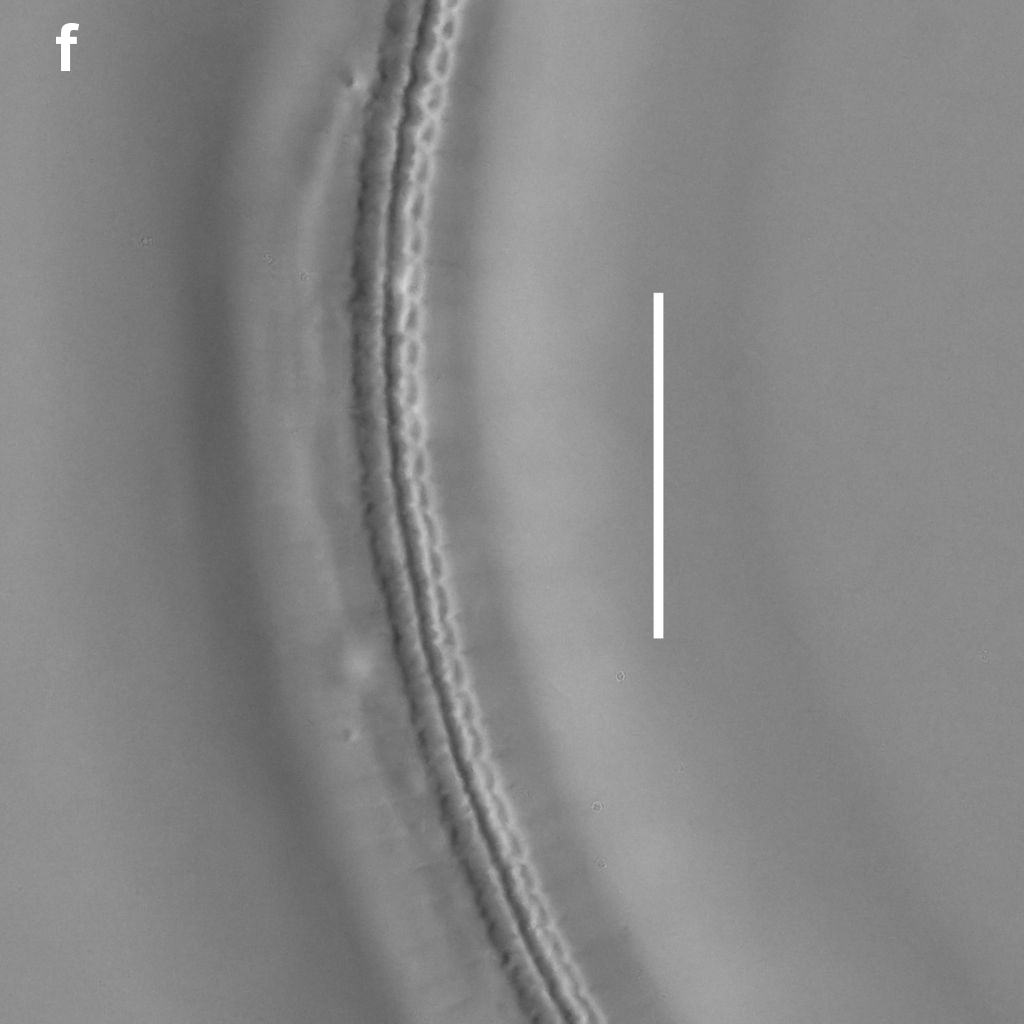
lateral alae at mid-body, subsurface view

**Figure 14a.**
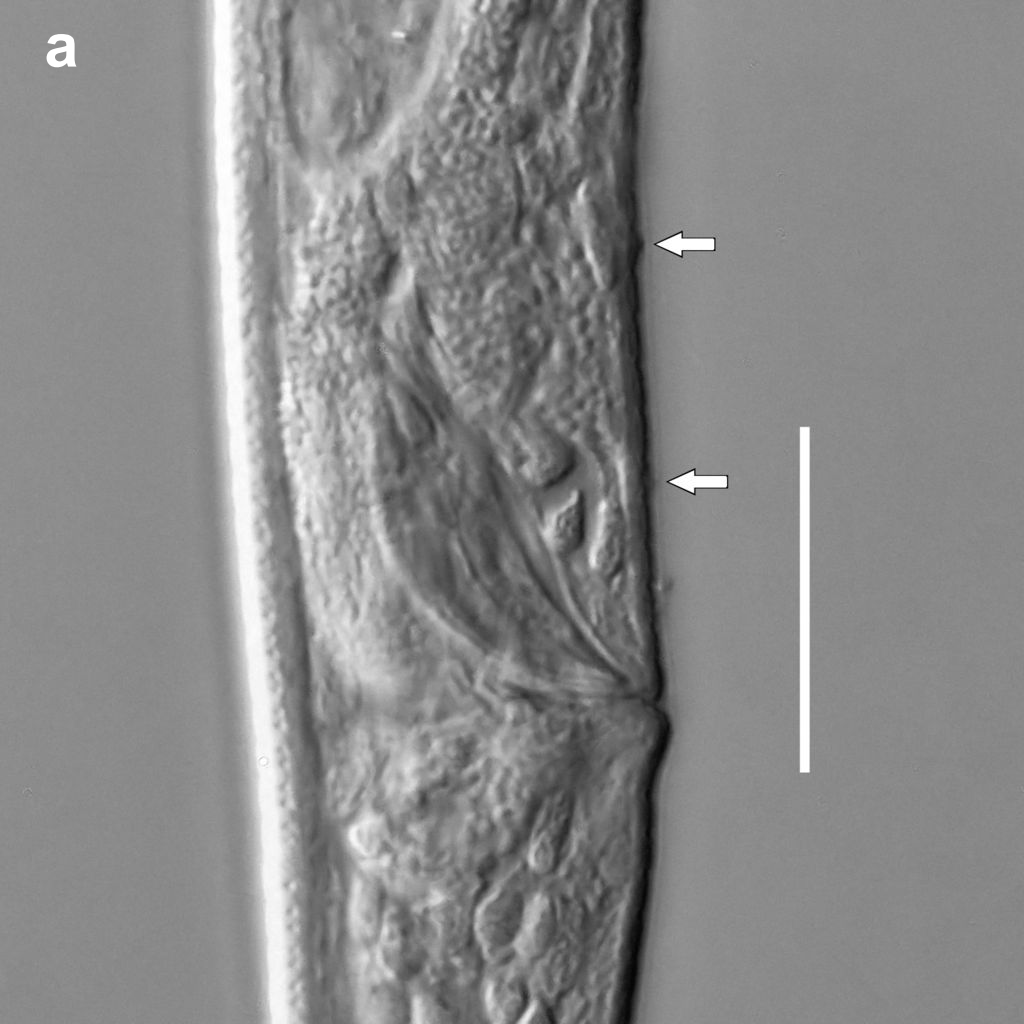
male cloacal region showing precloacal papilla (arrows)

**Figure 14b.**
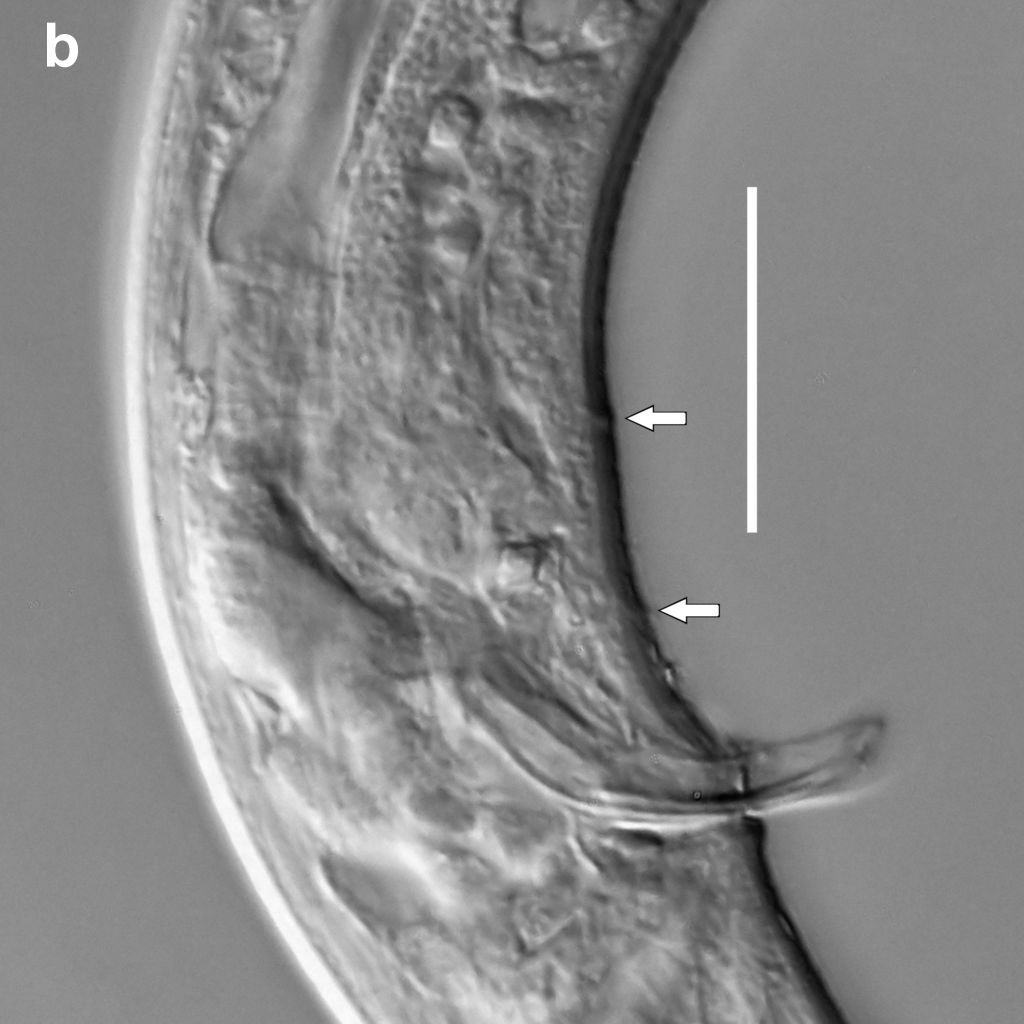
male cloacal region showing precloacal papilla (arrows) and protruding spicules

**Figure 14c.**
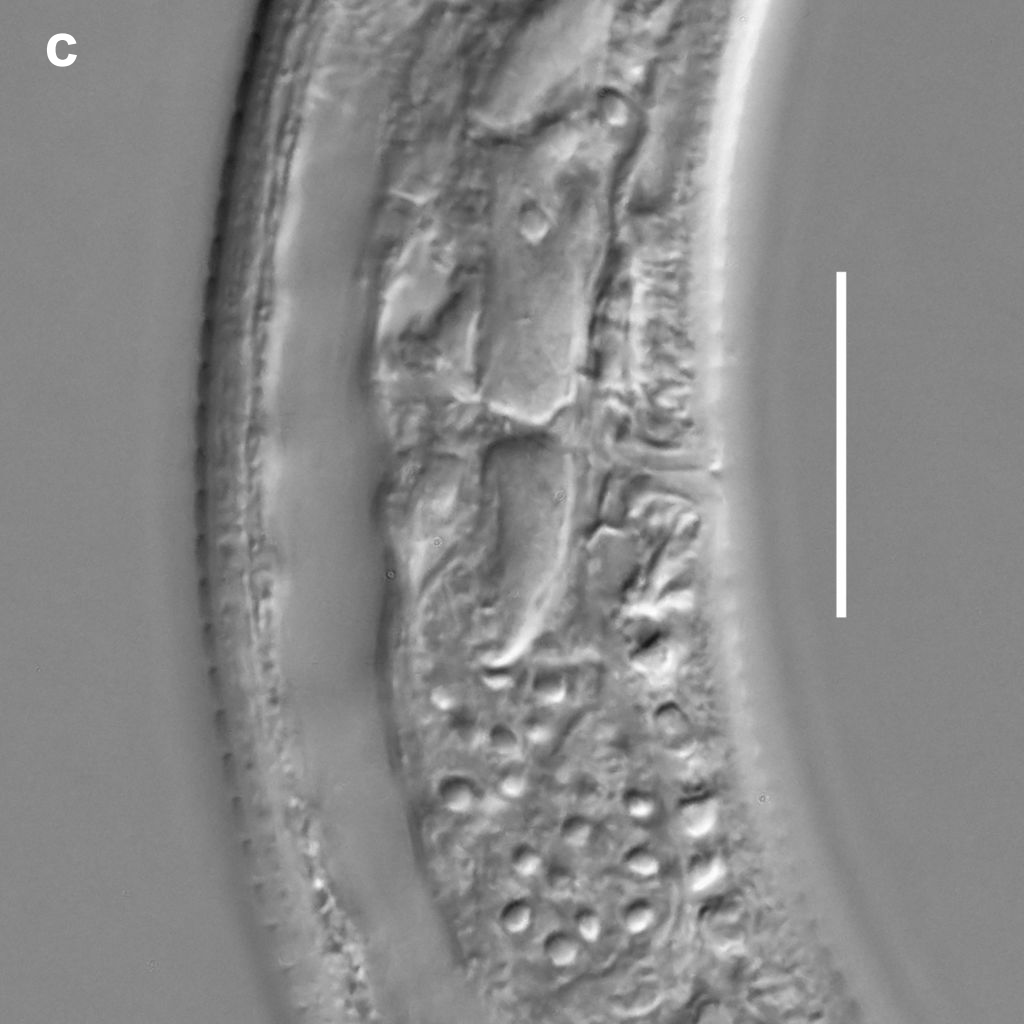
vulval region

**Figure 14d.**
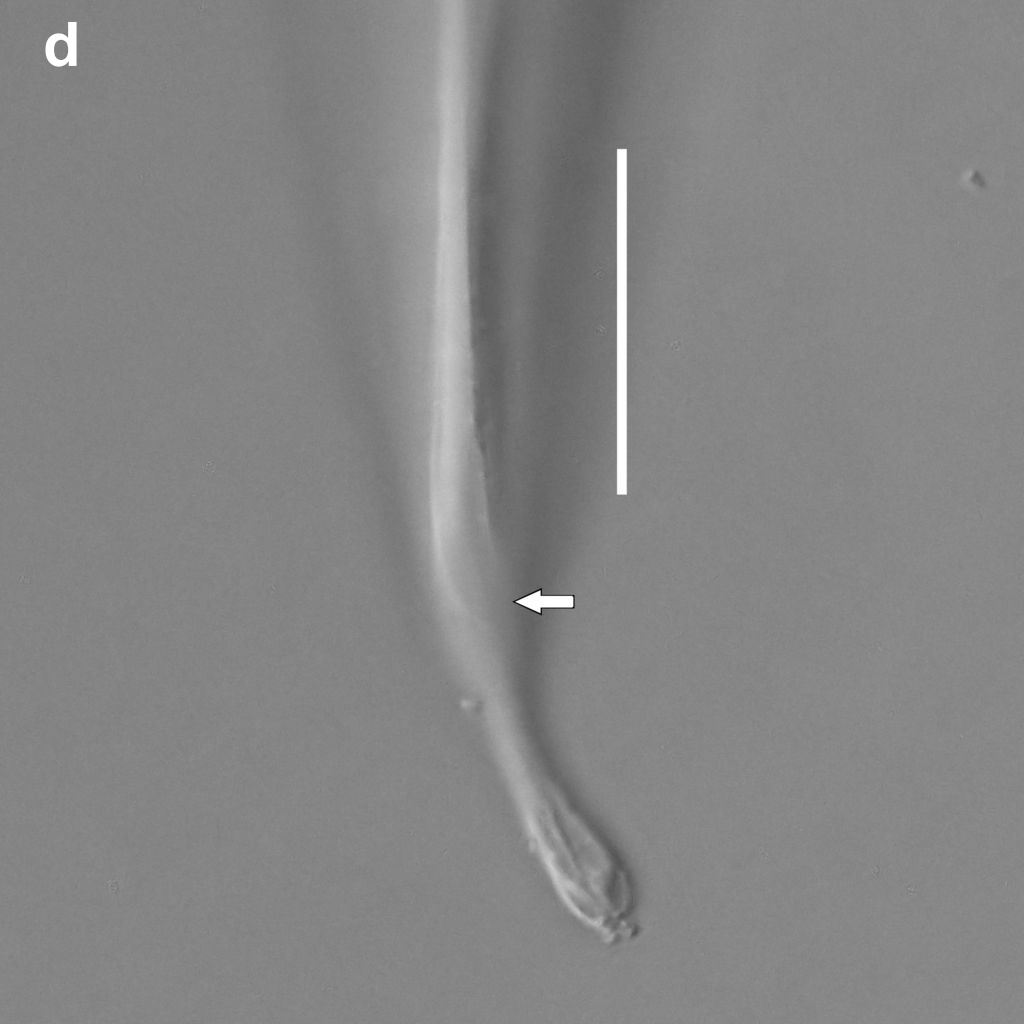
caudal region, surface view showing posterior end of lateral alae (arrow)

**Figure 16a.**
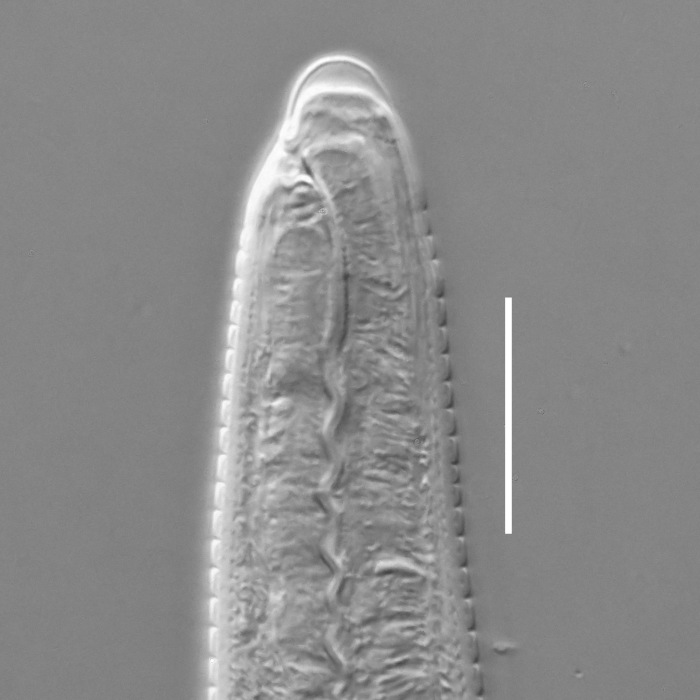
anterior end, median section (ventral side to the right)

**Figure 16b.**
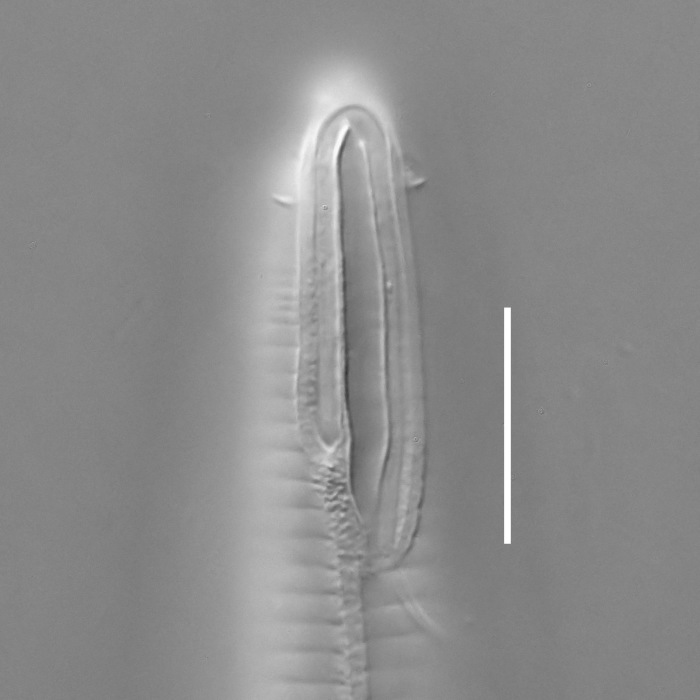
anterior end, surface view showing amphid (ventral side to the right)

**Figure 16c.**
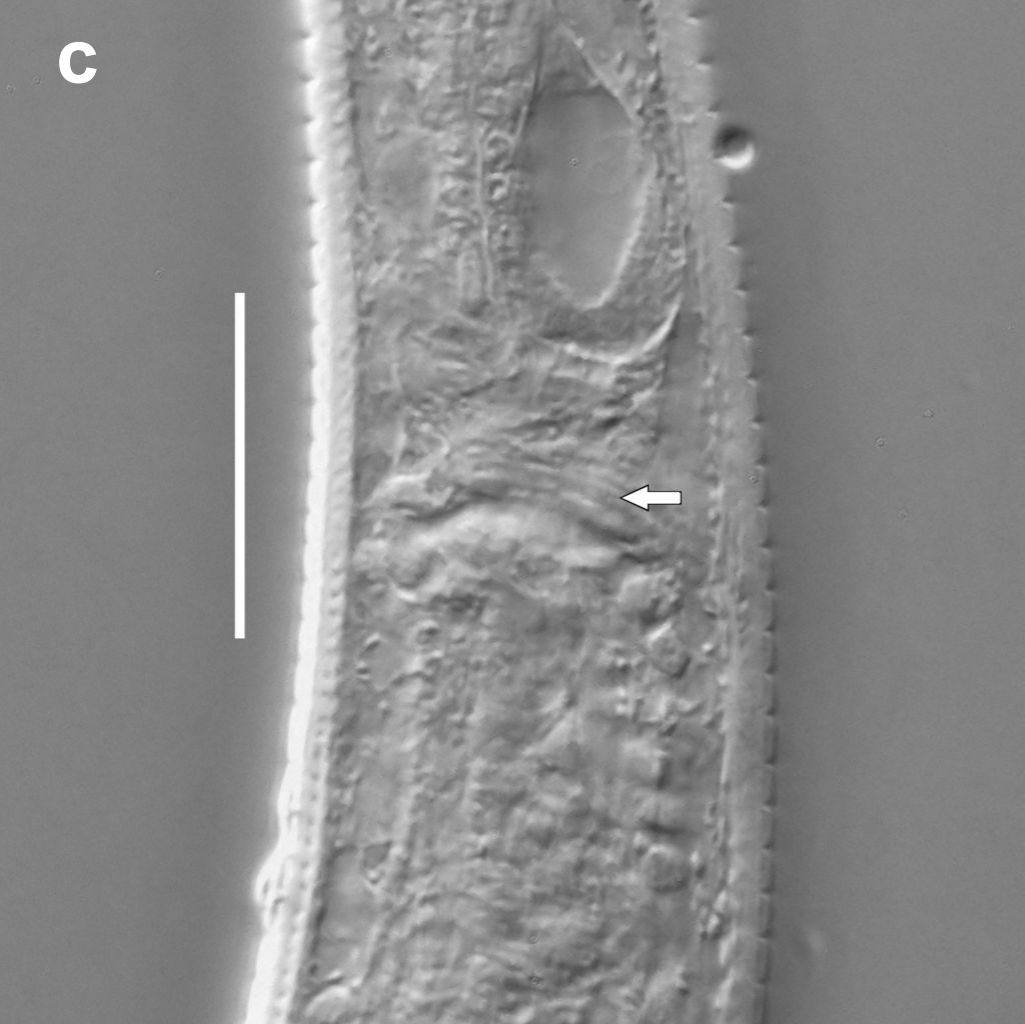
nerve ring encircling pharyngo-intestinal junction (arrow)

**Figure 16d.**
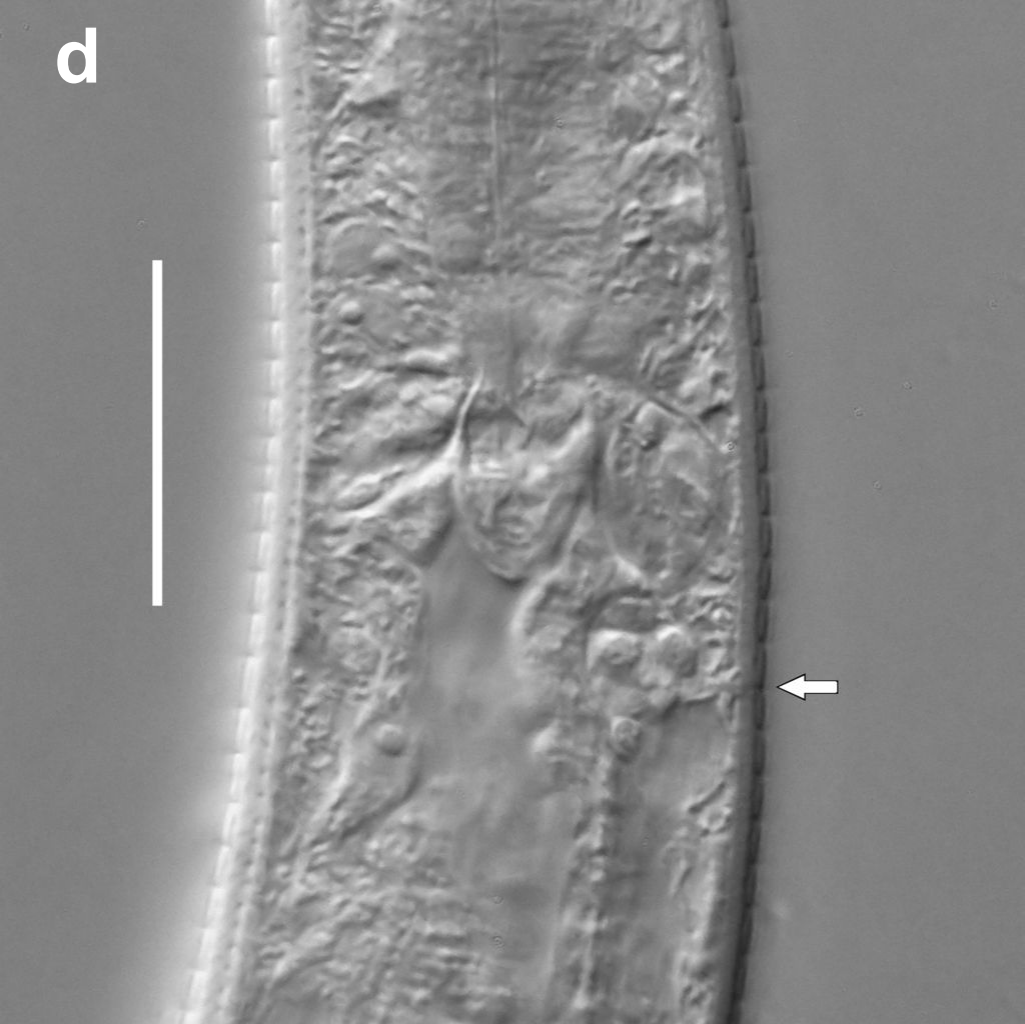
secretory-excretory pore just posterior to pharyngo-intestinal junction (arrow)

**Figure 17a.**
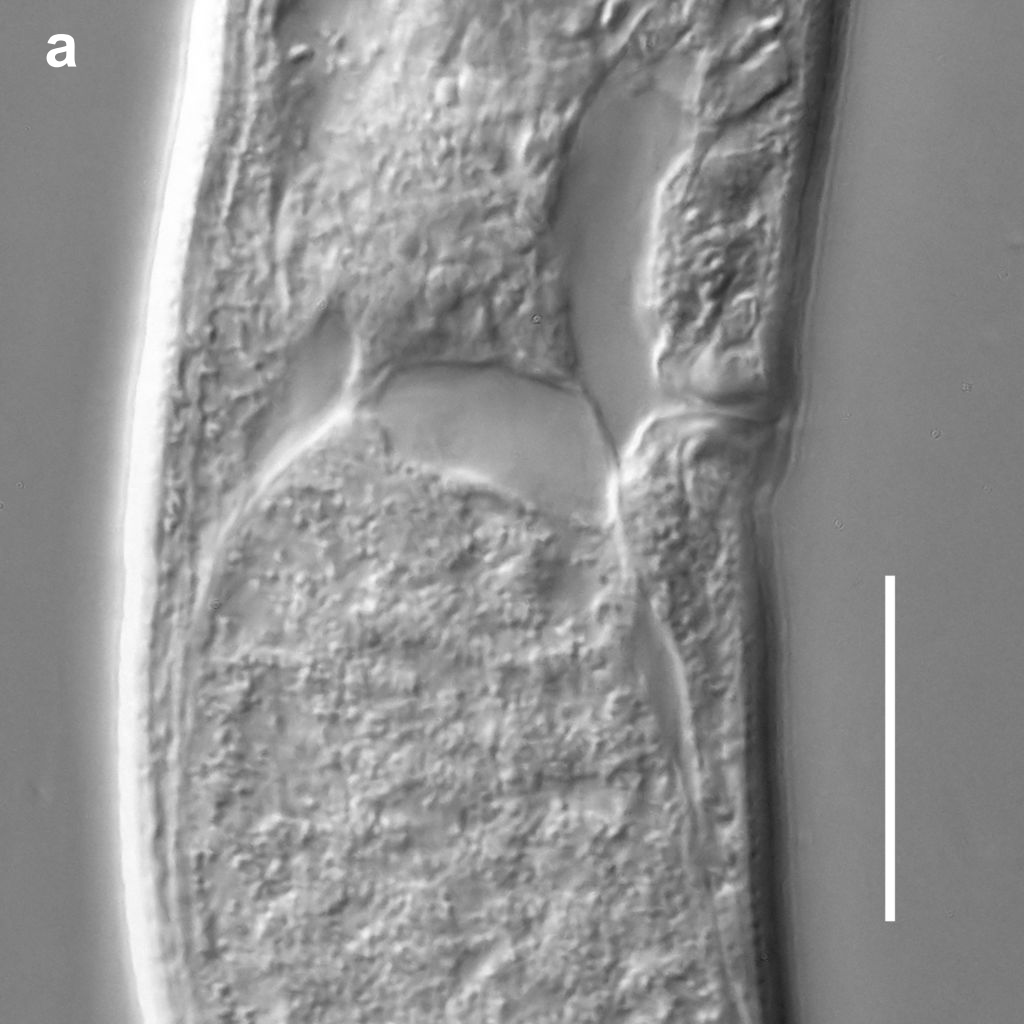
vulval region

**Figure 17b.**
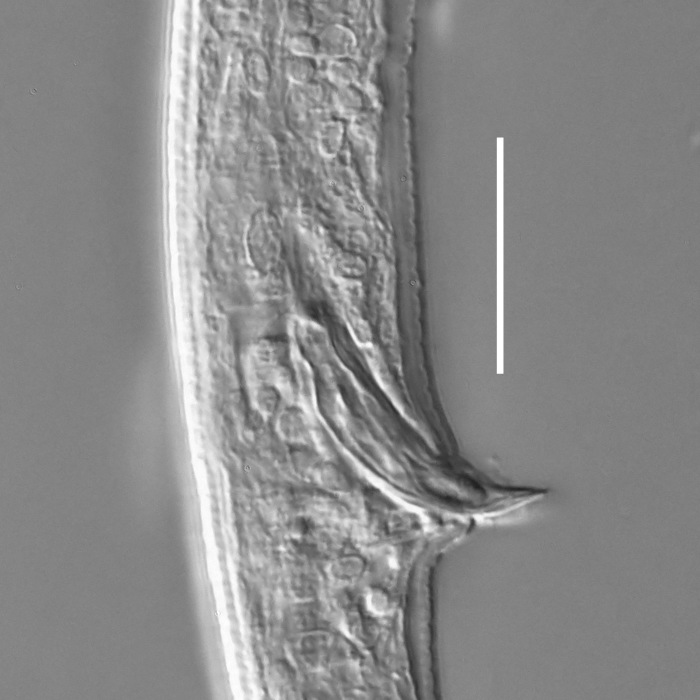
male cloacal region showing spicules

**Figure 17c.**
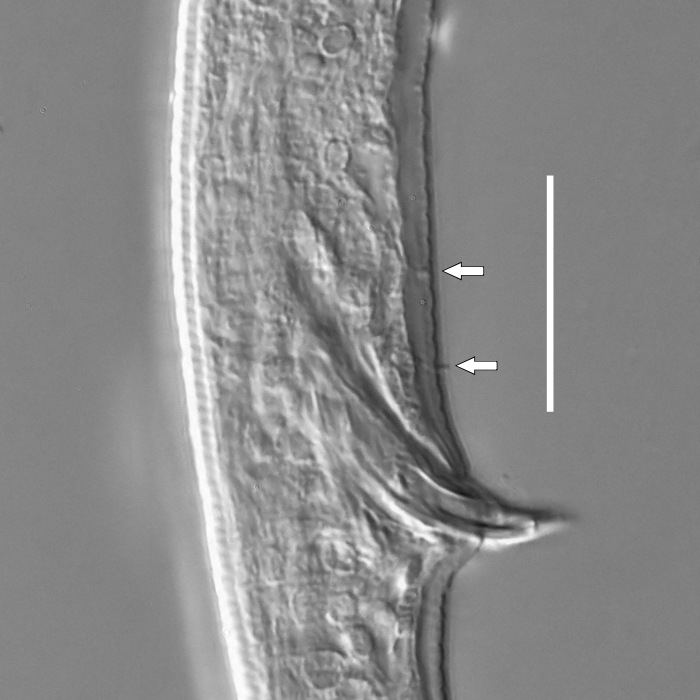
male cloacal region showing precloacal papilla in lateral view (arrows)

**Figure 17d.**
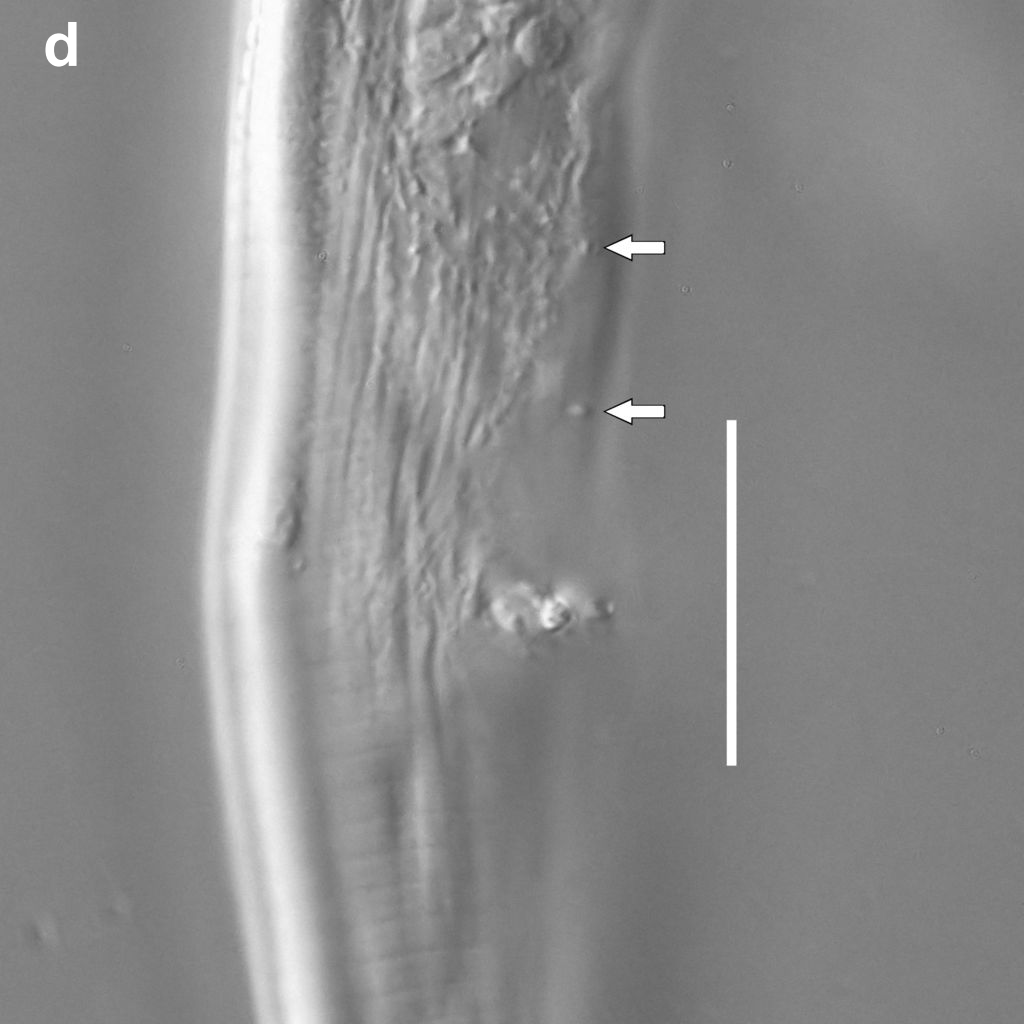
male cloacal region showing precloacal papilla in ventrosublateral view (arrows)

**Figure 17e.**
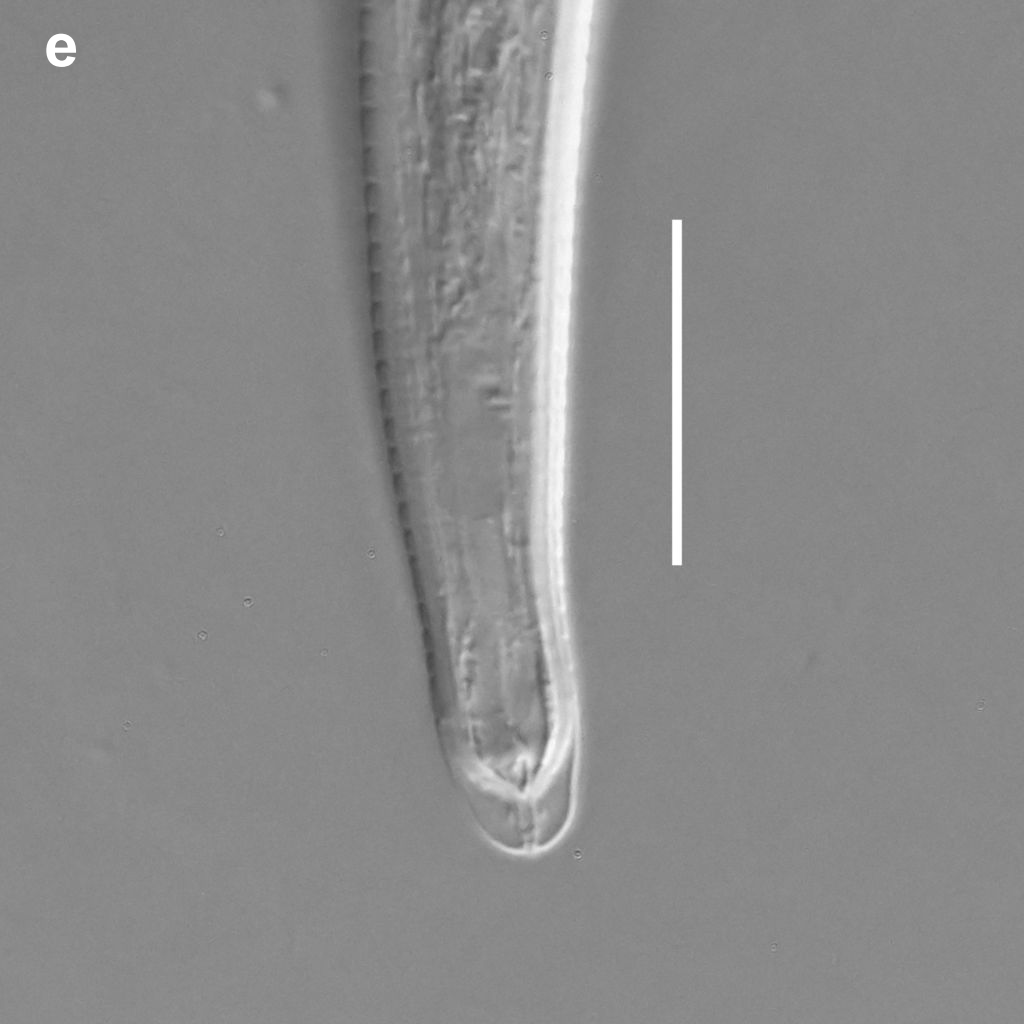
tail terminus and spinneret in lateral view

**Figure 17f.**
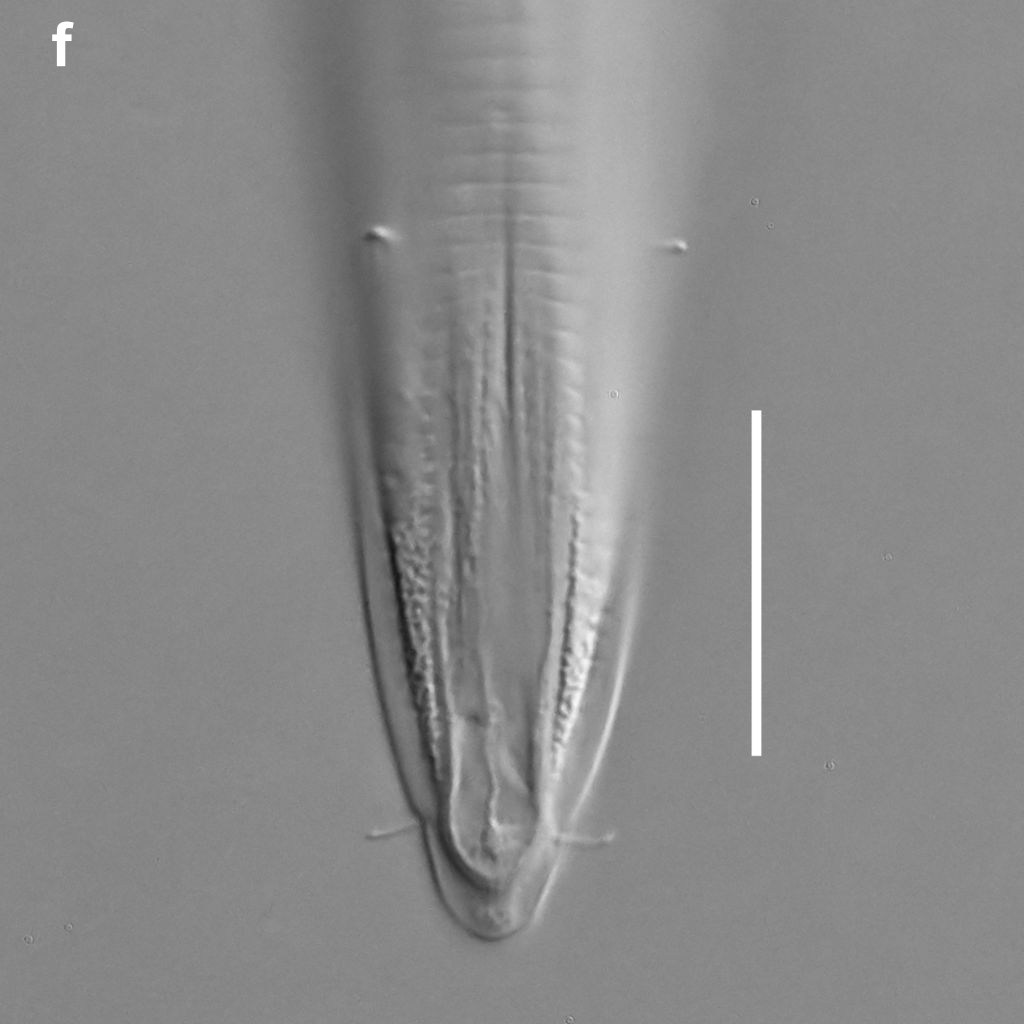
male tail terminus and spinneret in ventral view showing subterminal caudal setae

**Figure 19a.**
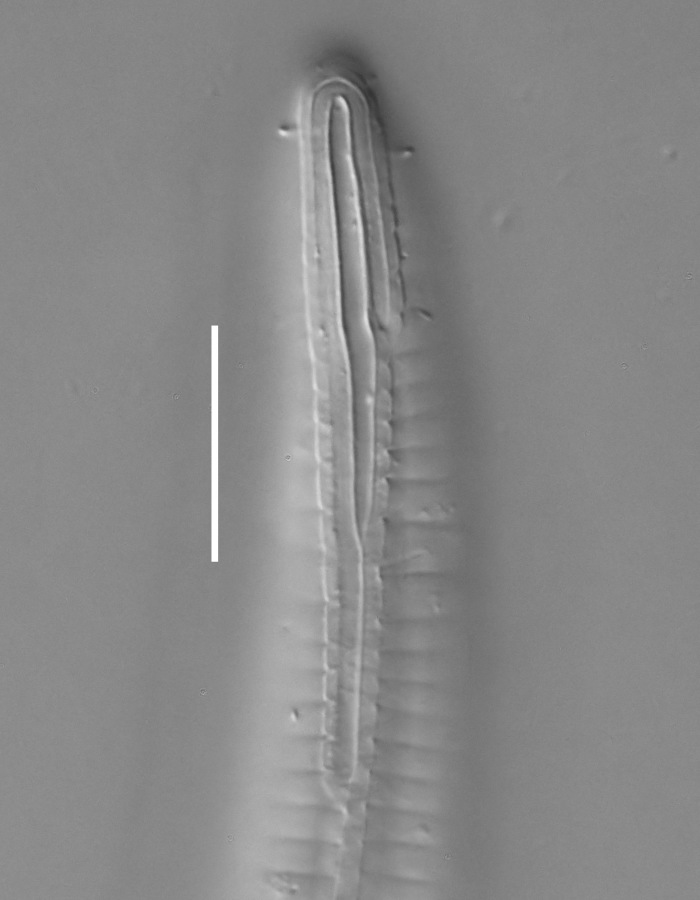
female anterior end, surface view showing amphid (ventral side to the left)

**Figure 19b.**
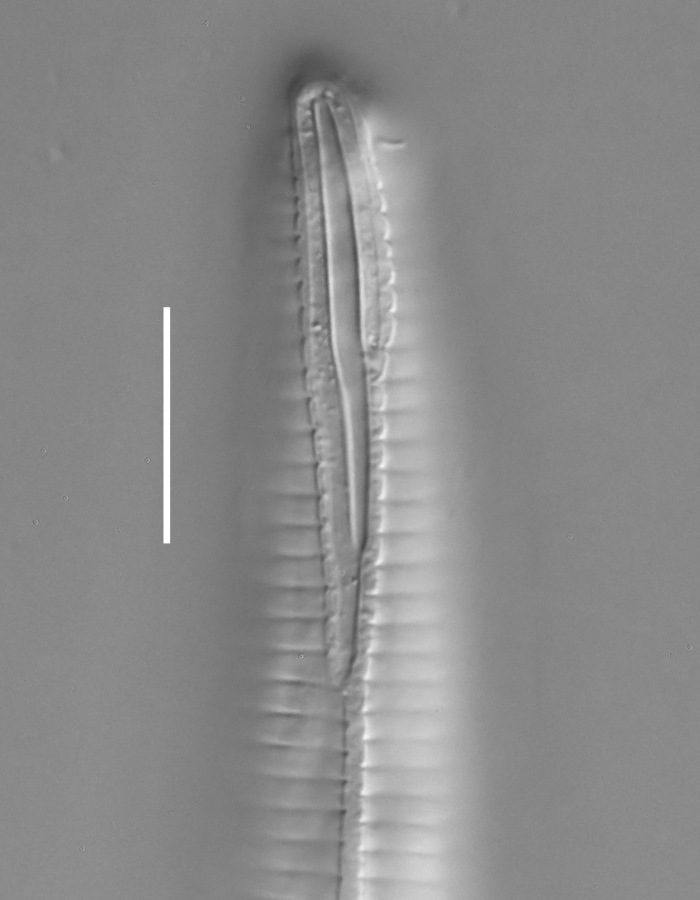
female anterior end, surface view showing amphid (ventral side to the left)

**Figure 19c.**
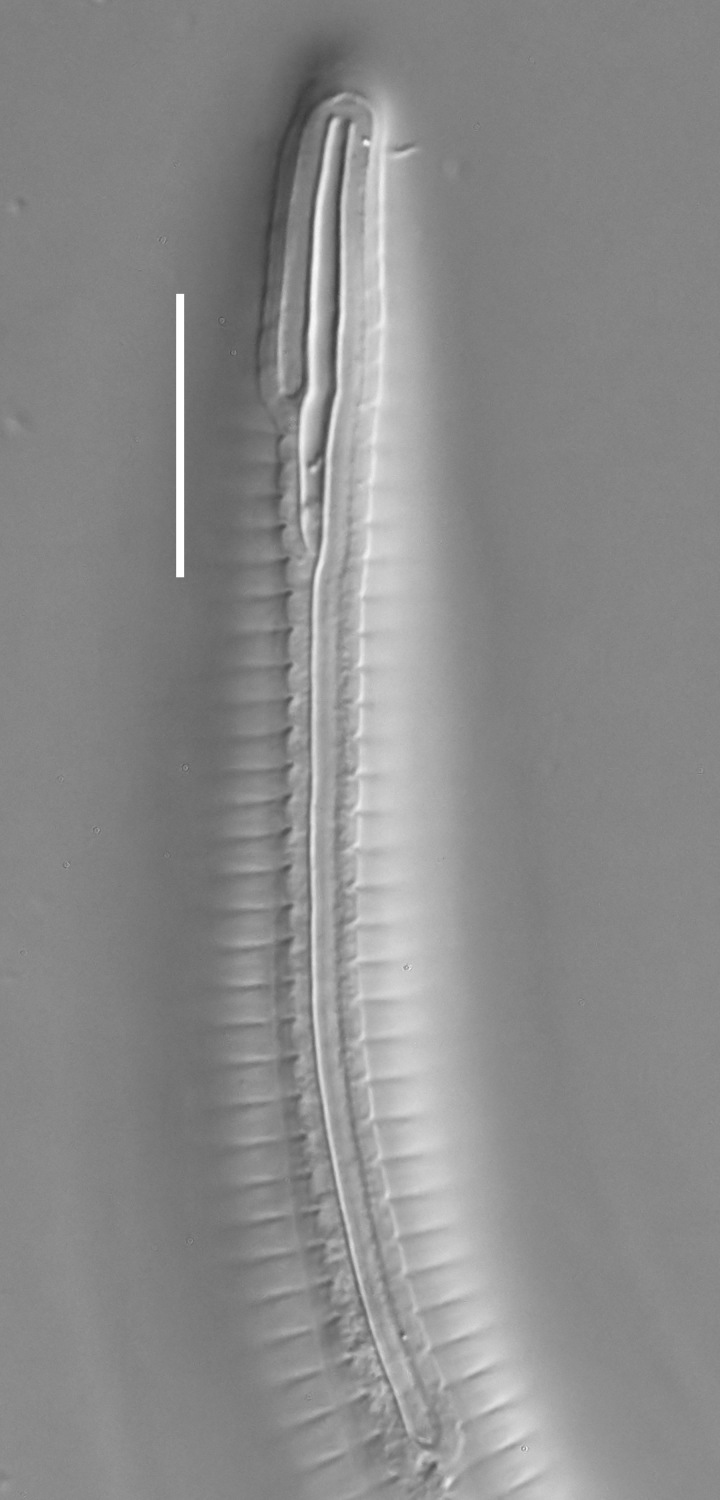
male anterior end, surface view showing amphid (ventral side to the right)

**Figure 19d.**
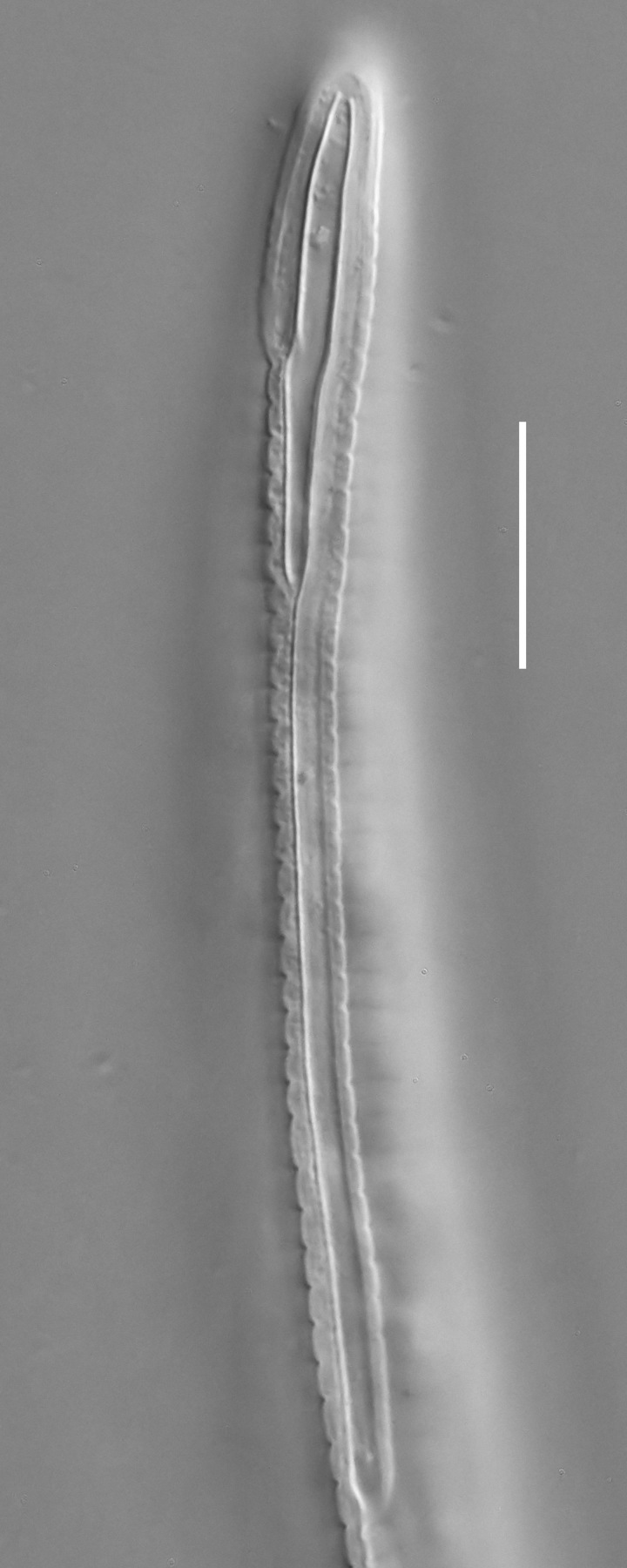
male anterior end, surface view showing amphid (ventral side to the right)

**Figure 20a.**
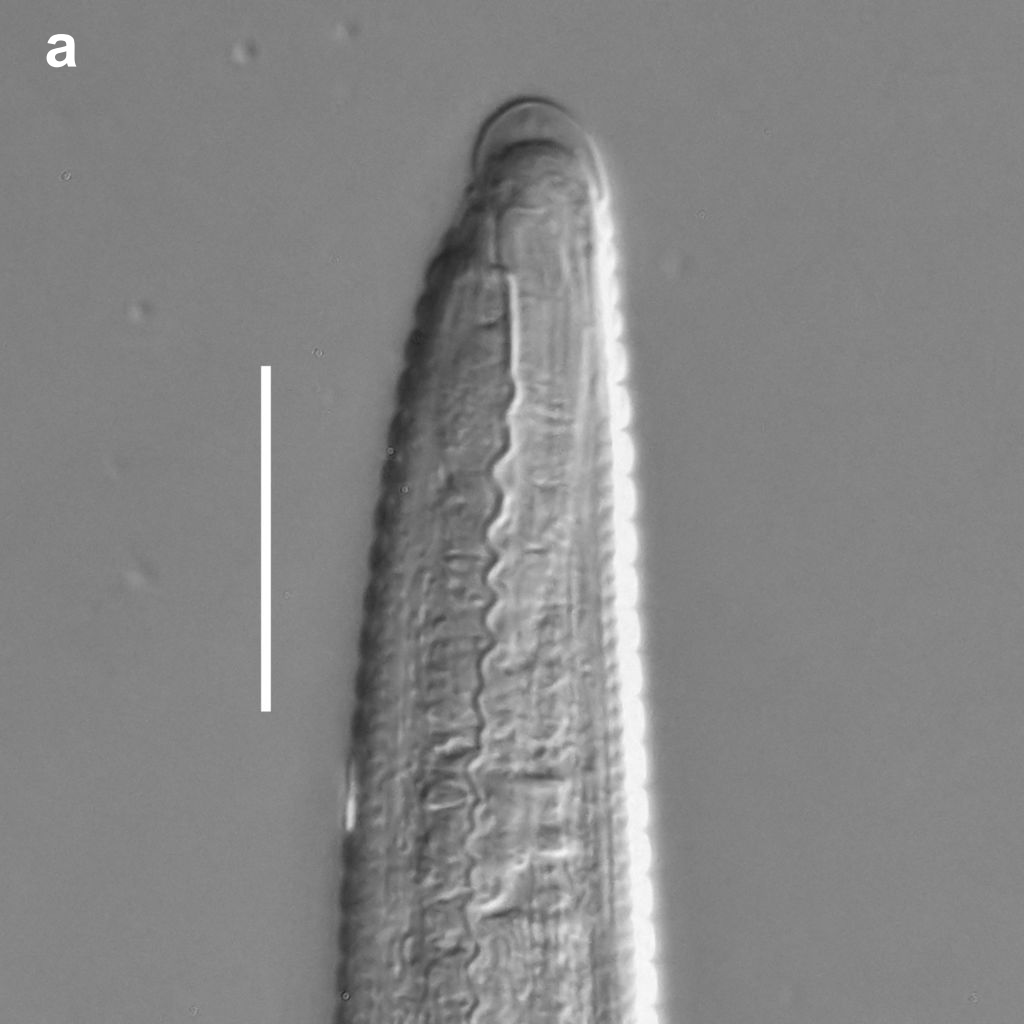
anterior end, median section (ventral side to the right)

**Figure 20b.**
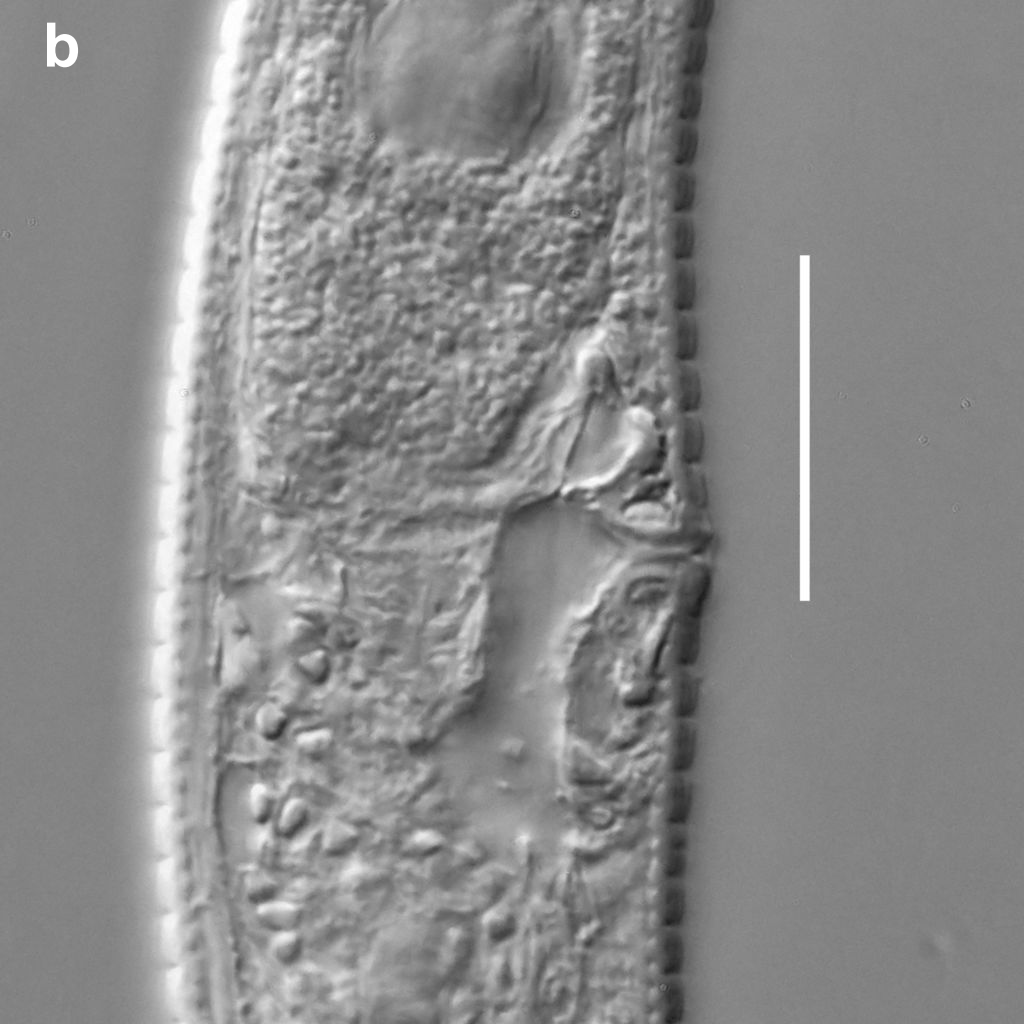
vulval region

**Figure 20c.**
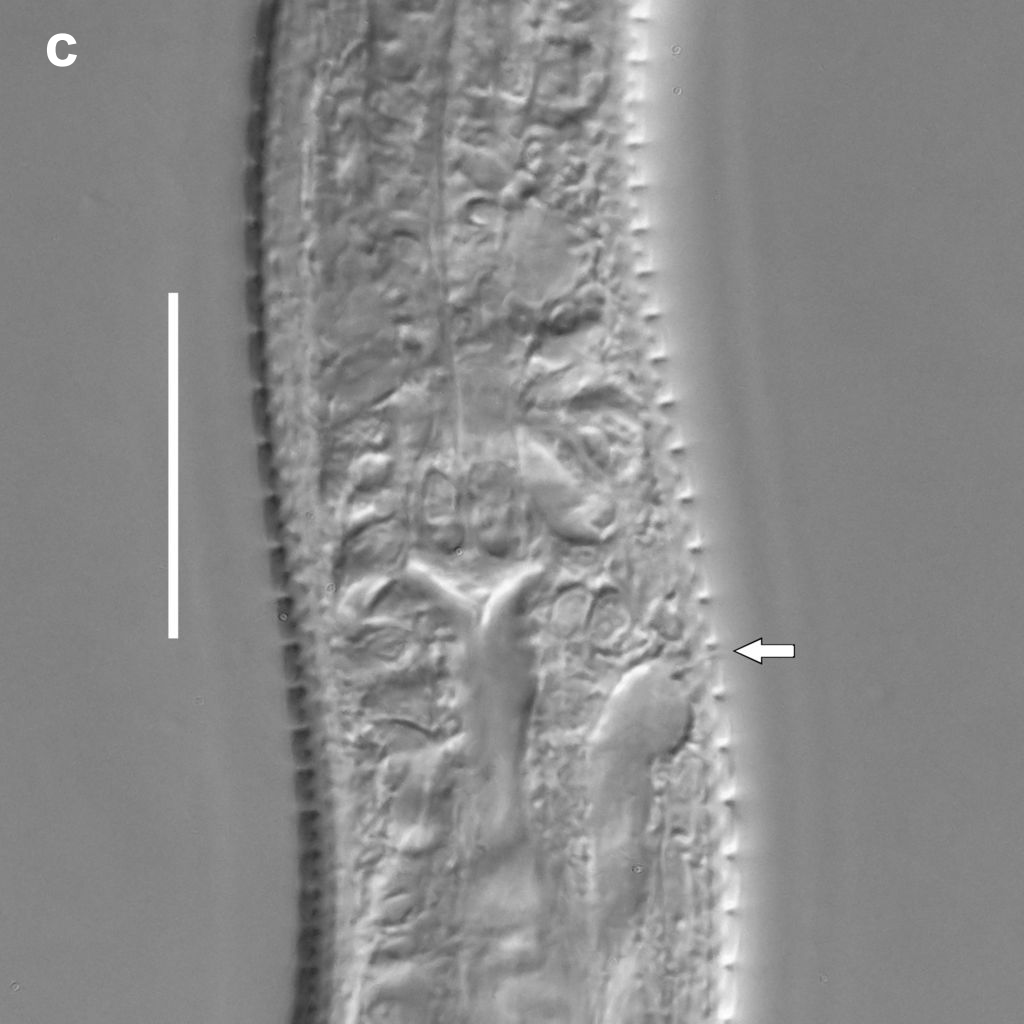
secretory-excretory pore opening just posterior to pharyngo-intestinal junction (arrow)

**Figure 20d.**
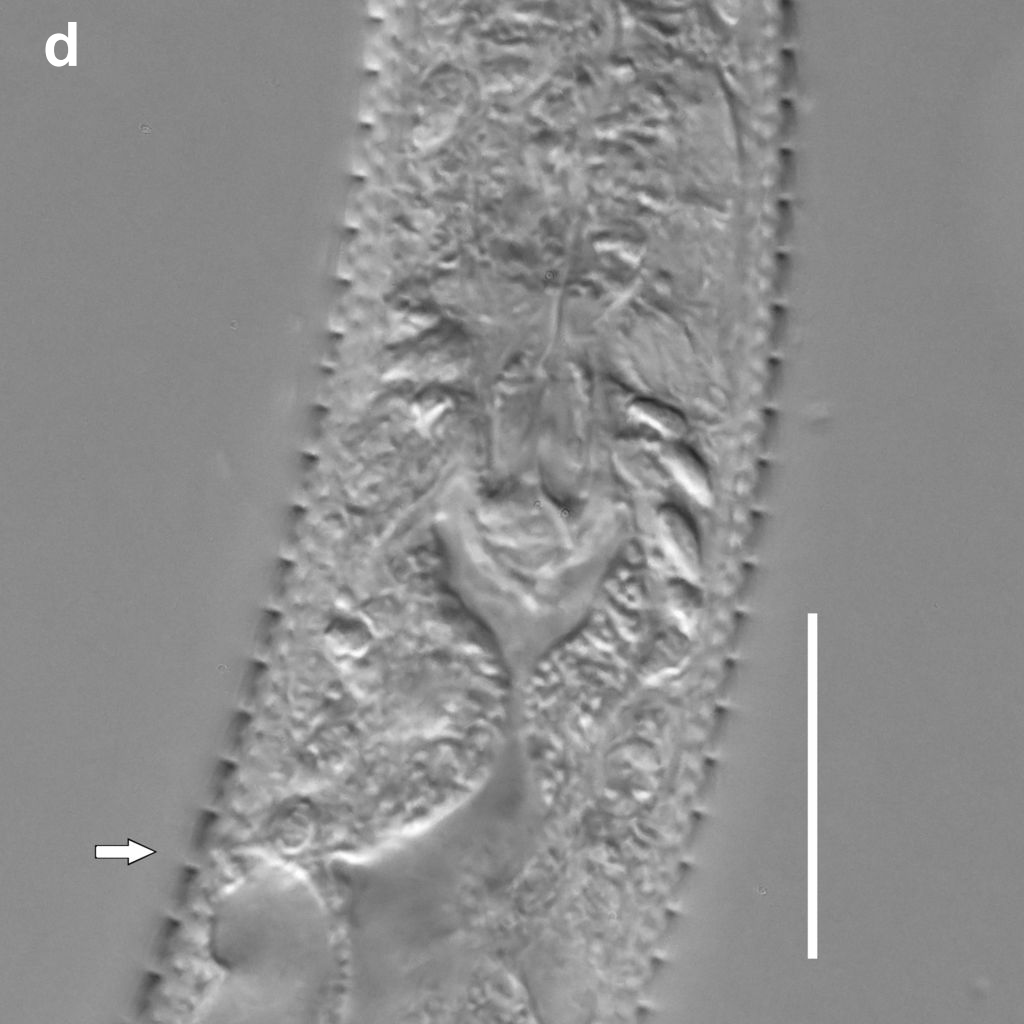
secretory-excretory pore opening just posterior to pharyngo-intestinal junction (arrow)

**Figure 21a.**
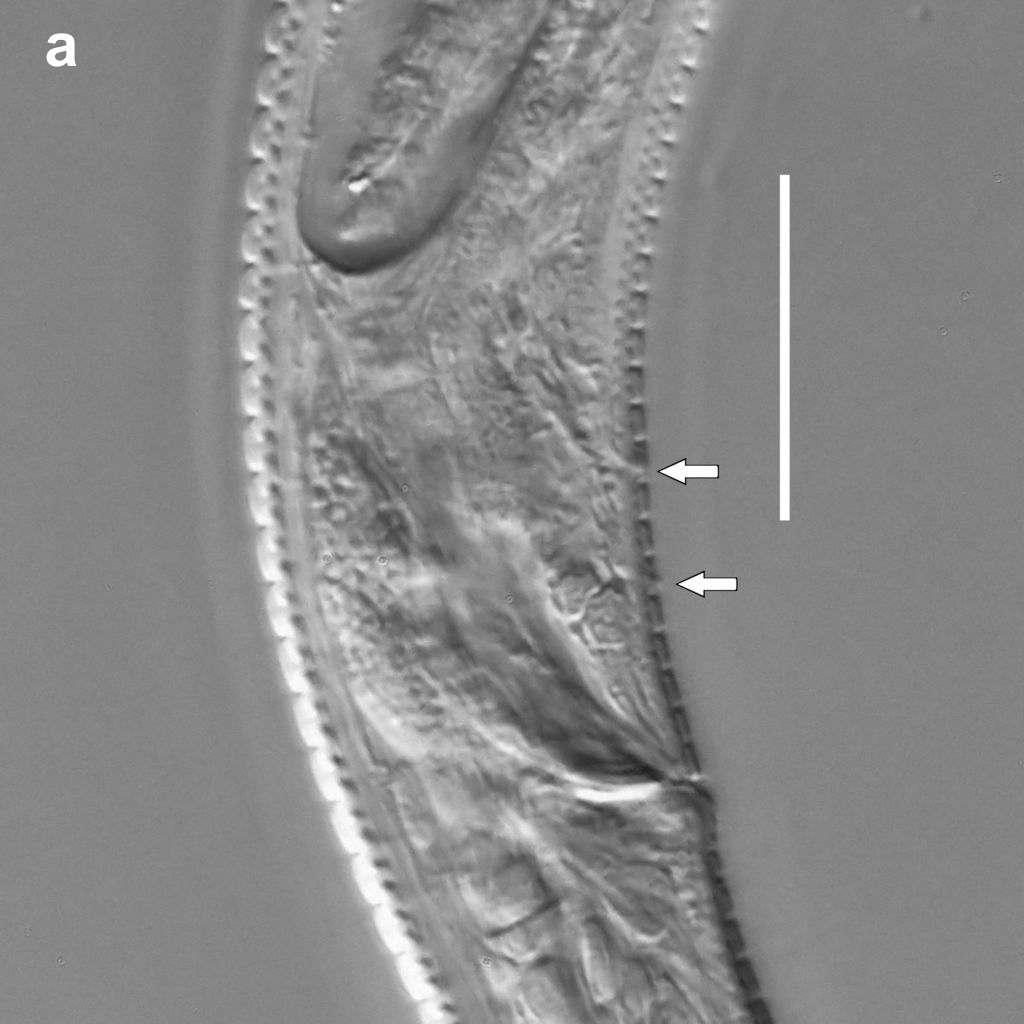
male cloacal region showing precloacal papilla (arrows)

**Figure 21b.**
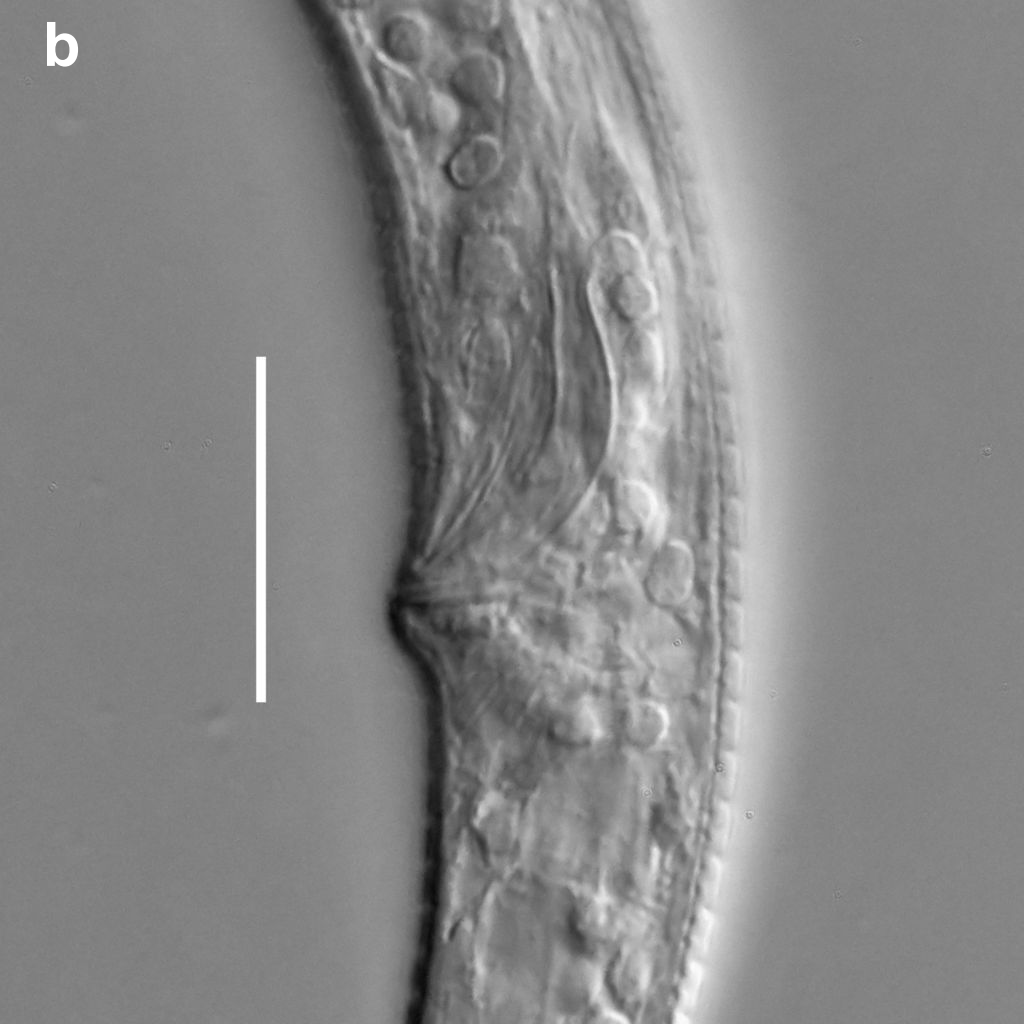
male cloacal region showing spicules

**Figure 21c.**
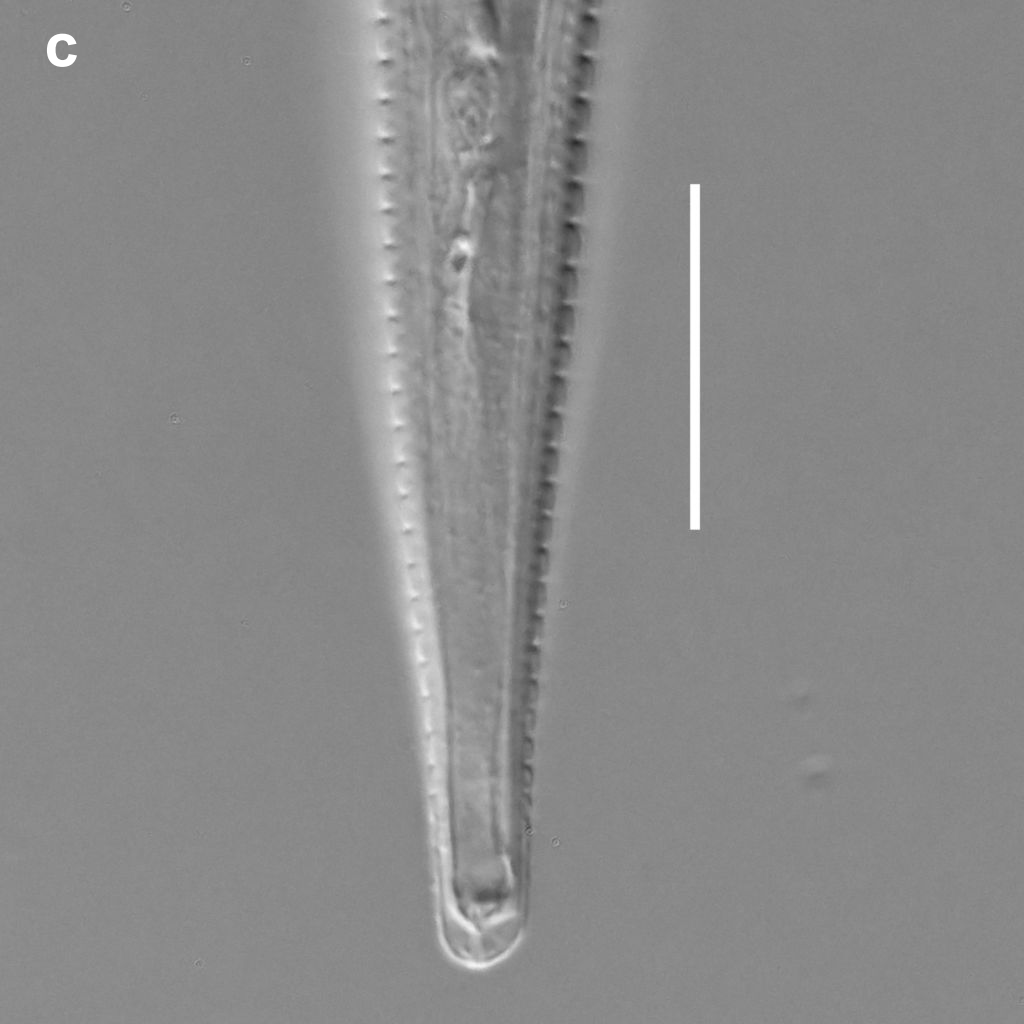
tail terminus and spinneret

**Figure 21d.**
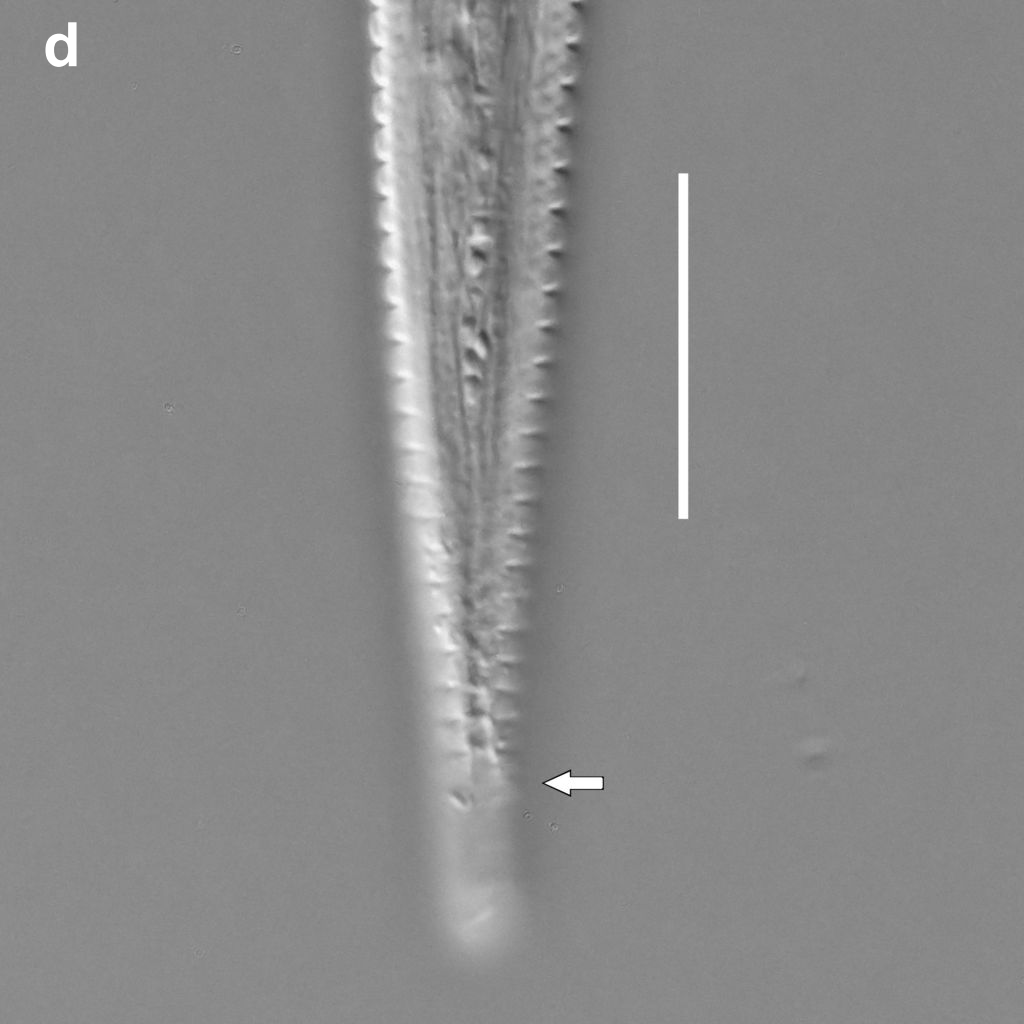
caudal region, surface view showing posterior end of lateral alae (arrow)

**Figure 23a.**
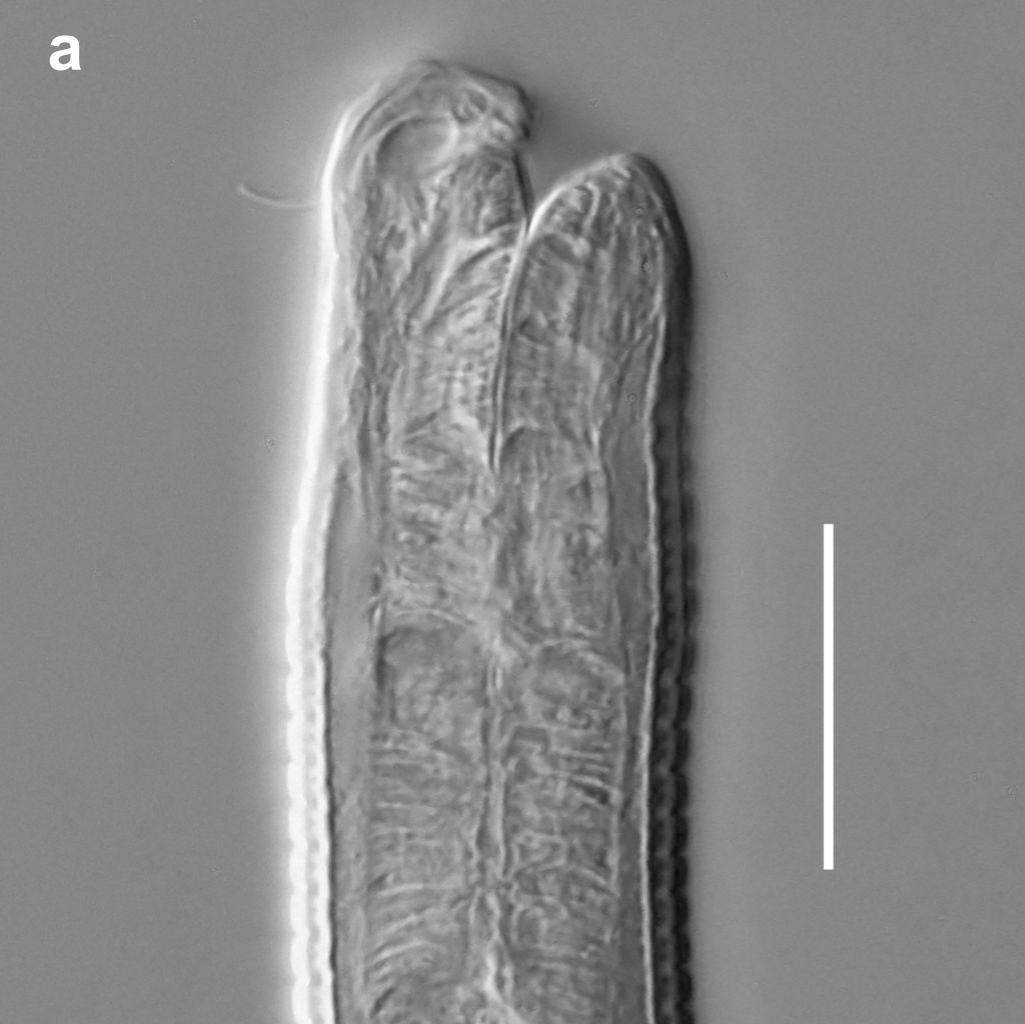
anterior end, median section (ventral side to the left)

**Figure 23b.**
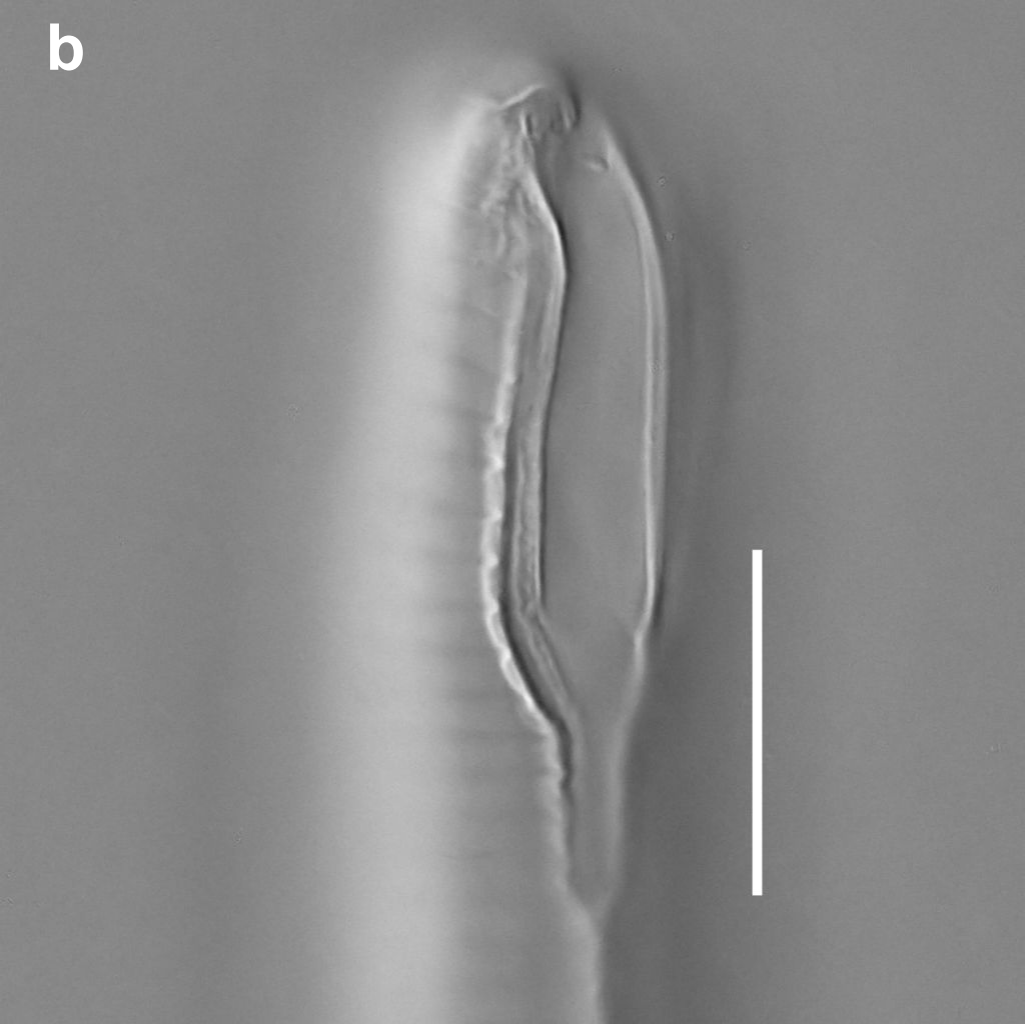
anterior end, surface view showing amphid (ventral side to the left)

**Figure 23c.**
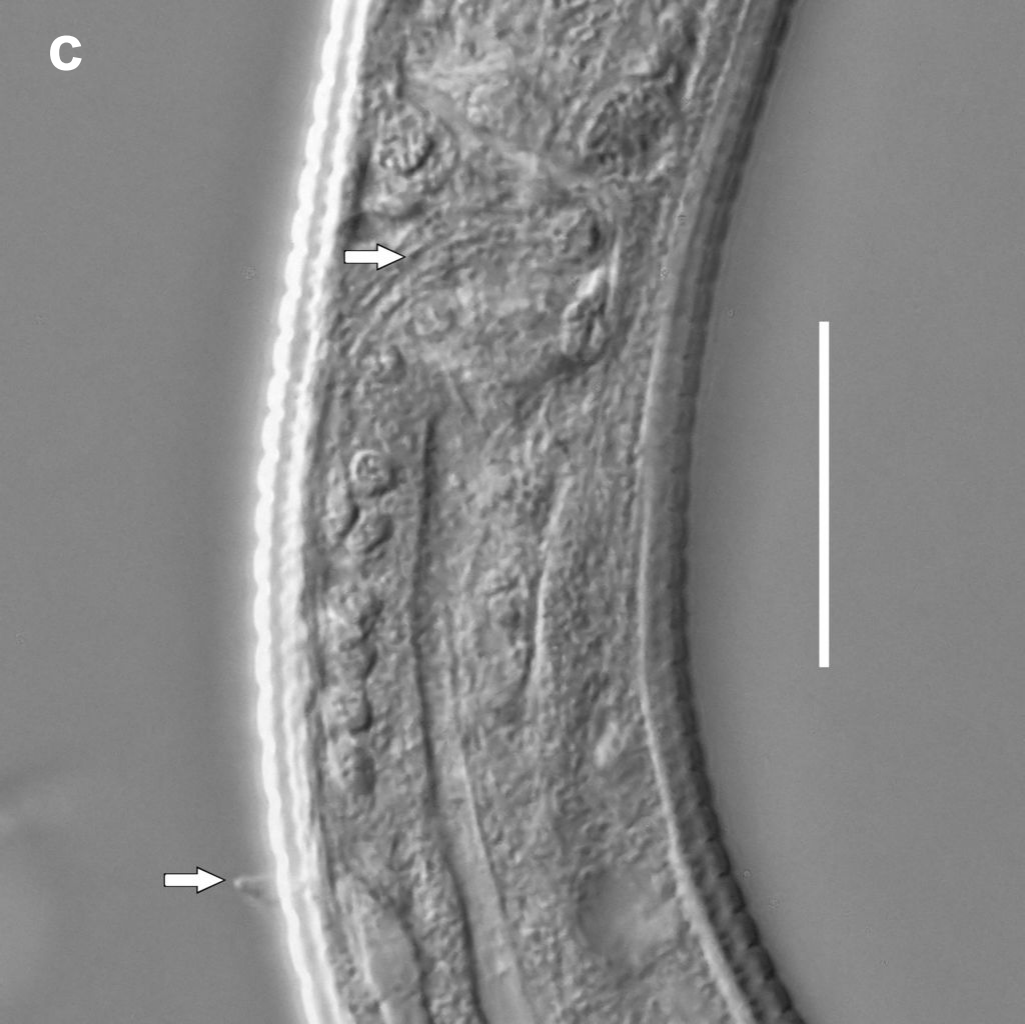
nerve ring encircling pharyngo-intestinal junction and secretory-excretory pore at level with anterior part of intestine (arrows)

**Figure 23d.**
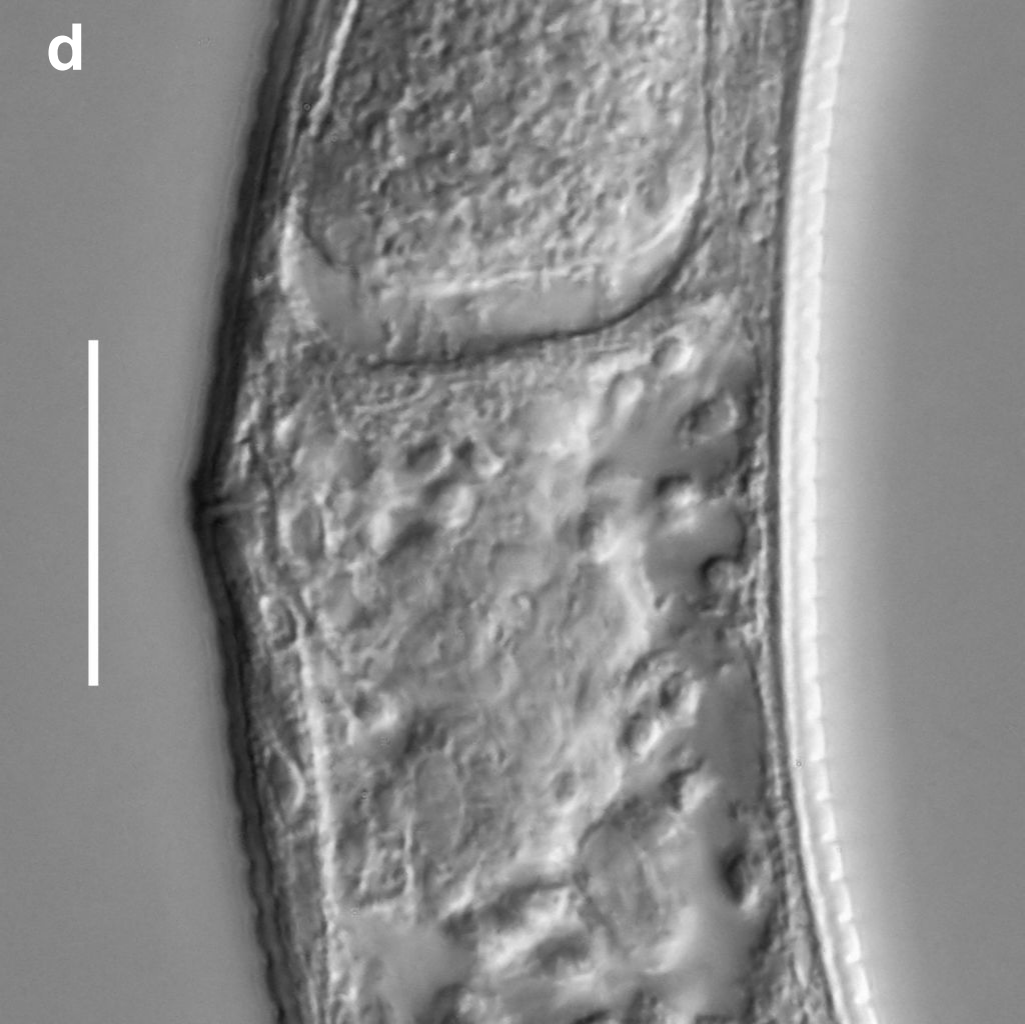
vulval region

**Figure 23e.**
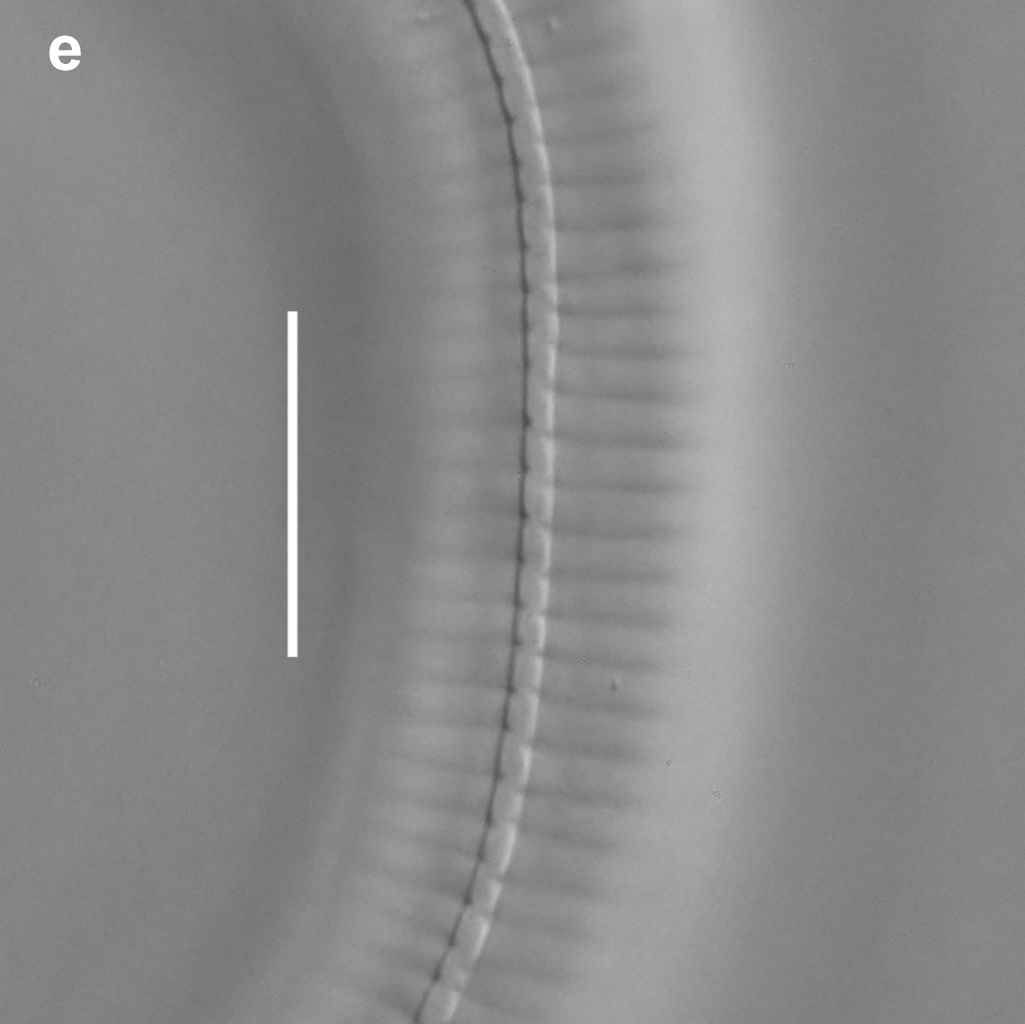
lateral alae at mid-body, surface view

**Figure 23f.**
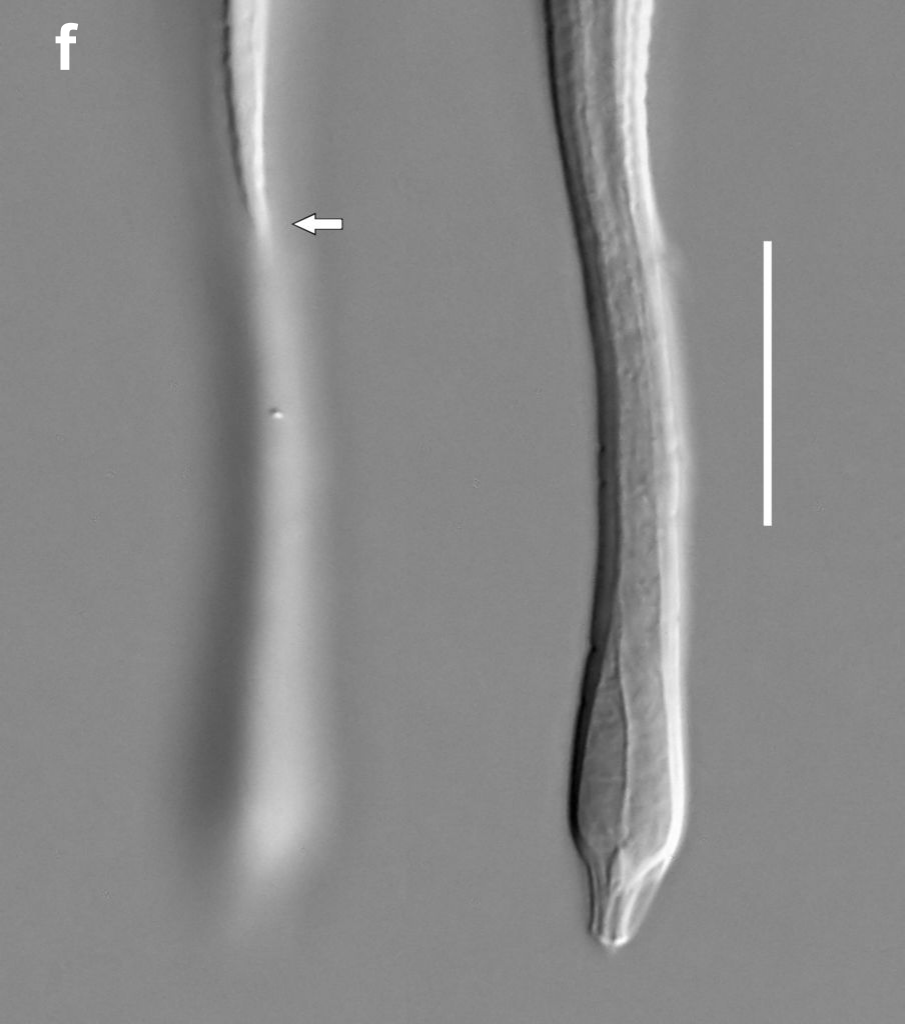
caudal region, surface view showing posterior end of lateral alae (arrow, left) and tail terminus and spinneret (right)

**Figure 25a.**
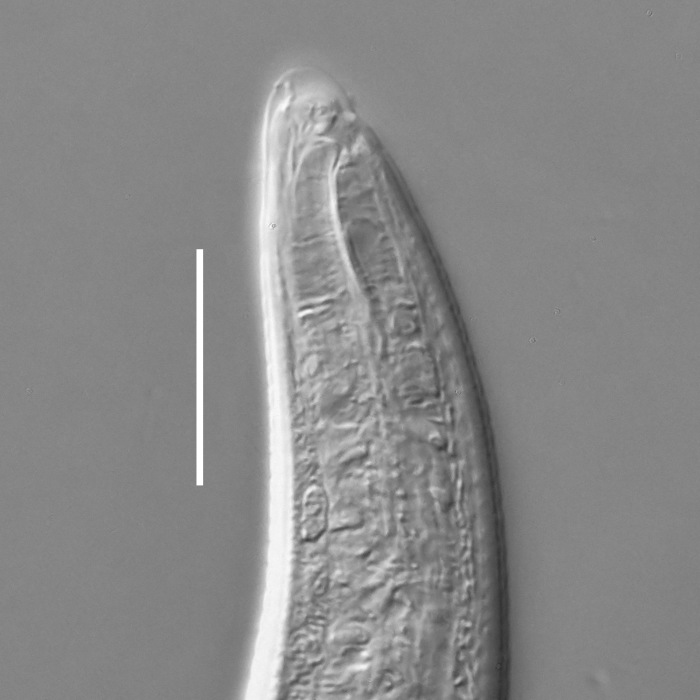
anterior end, median section (ventral side to the left)

**Figure 25b.**
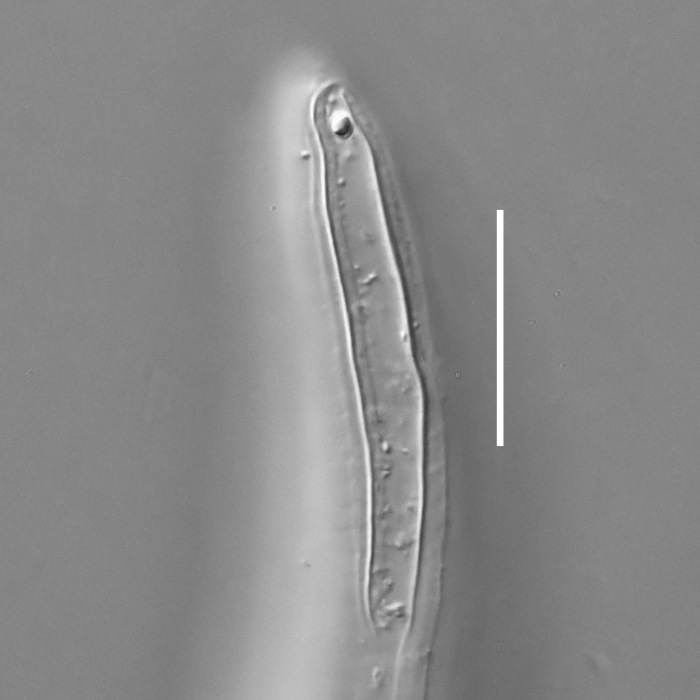
anterior end, surface view showing amphid (ventral side to the left)

**Figure 25c.**
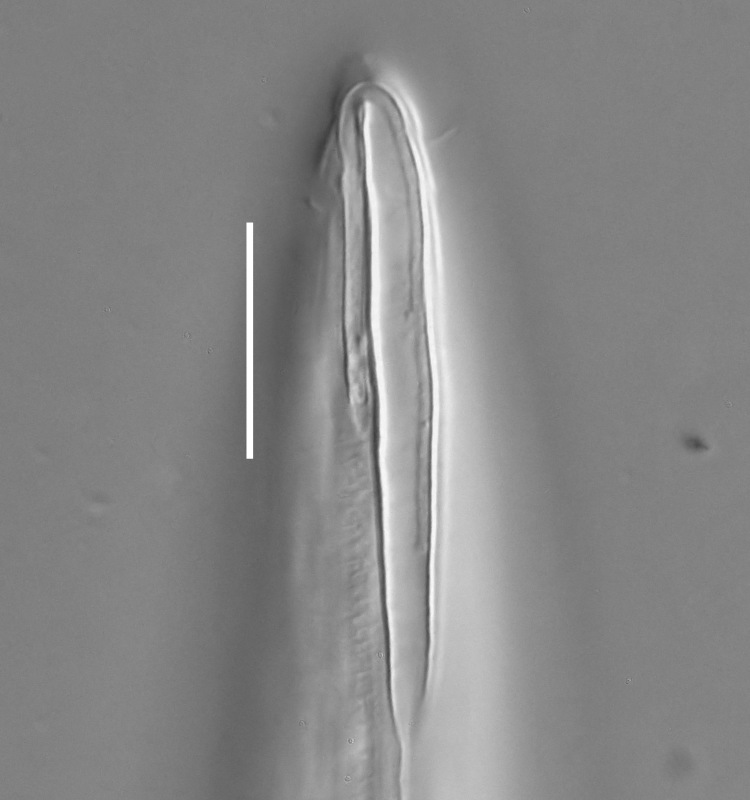
anterior end, surface view showing amphid (ventral side to the right)

**Figure 25d.**
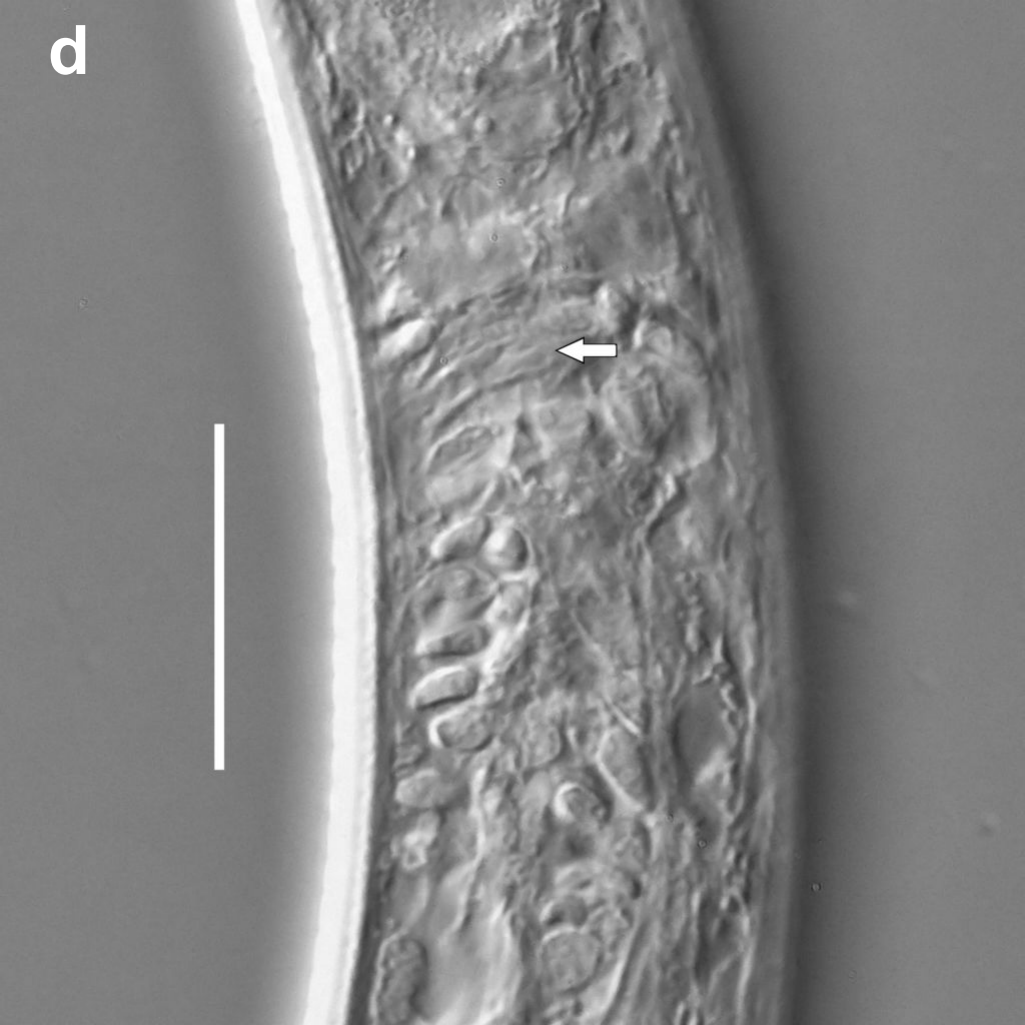
nerve ring encircling pharyngo-intestinal junction (arrow)

**Figure 25e.**
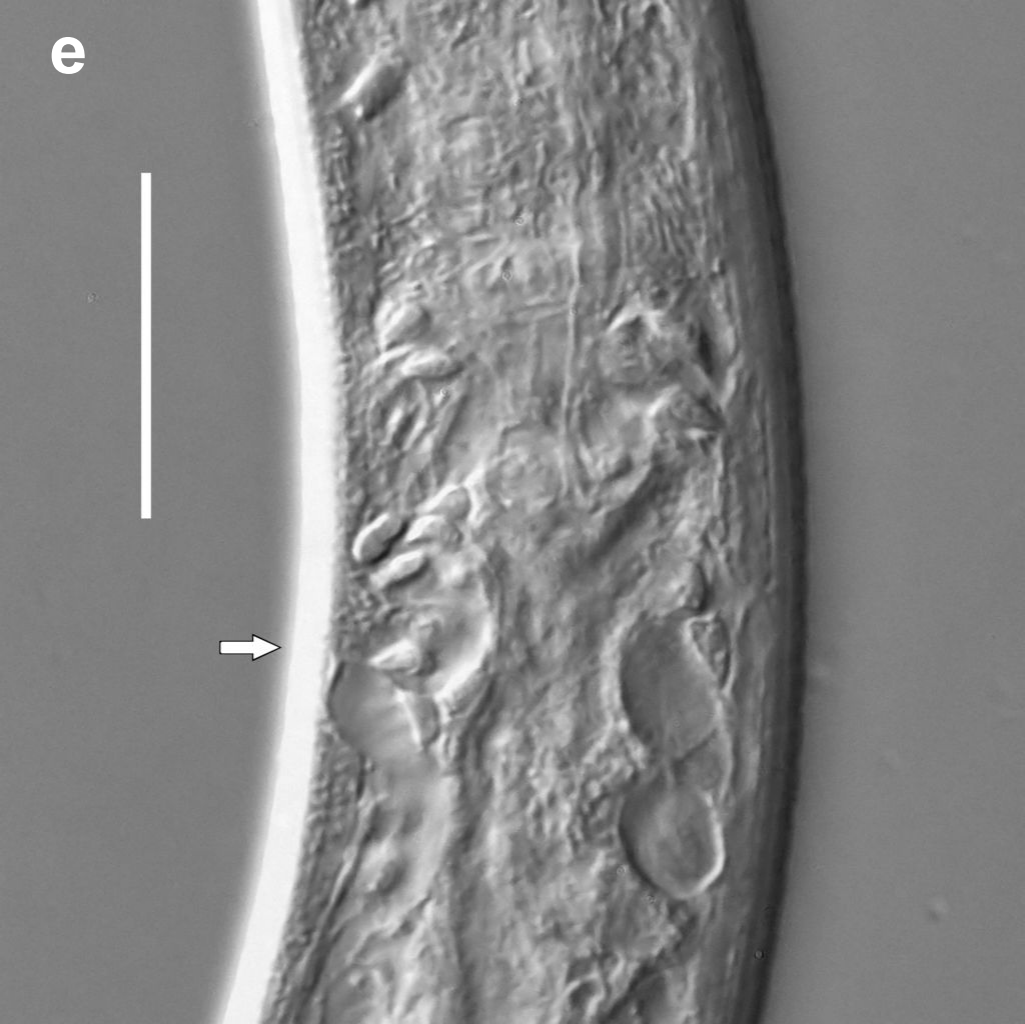
secretory-excretory pore just posterior to pharyngo-intestinal junction (arrow)

**Figure 25f.**
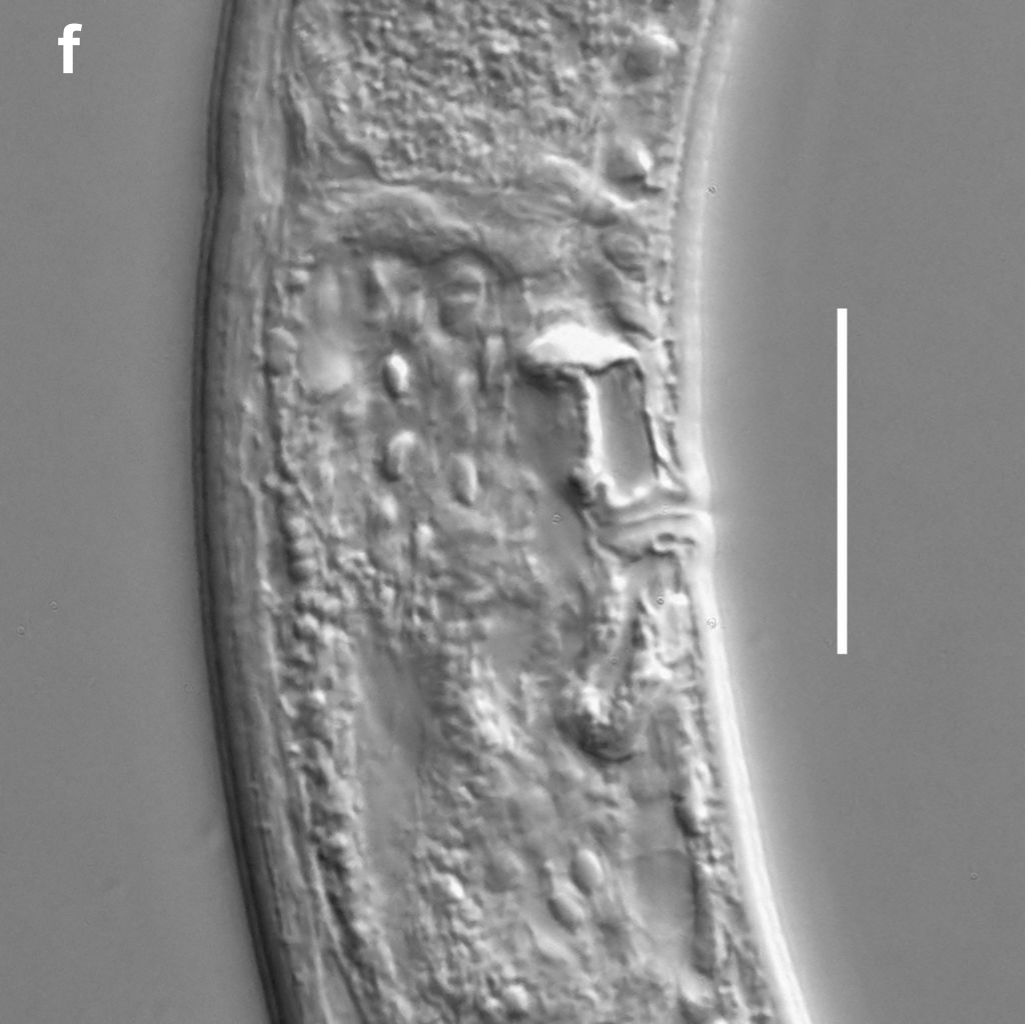
vulval region

**Figure 27a.**
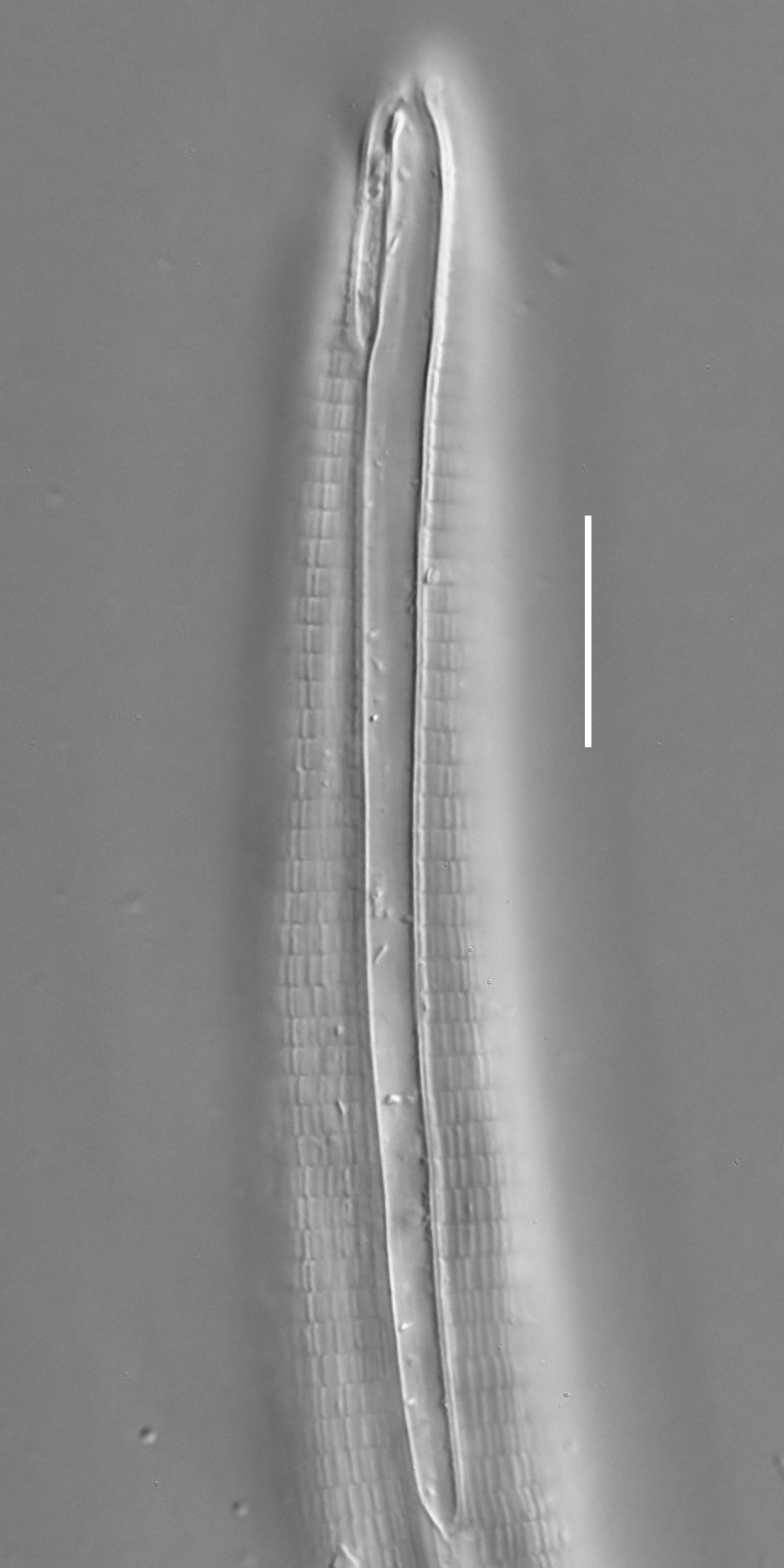
female anterior end, surface view showing amphid (ventral side to the right)

**Figure 27b.**
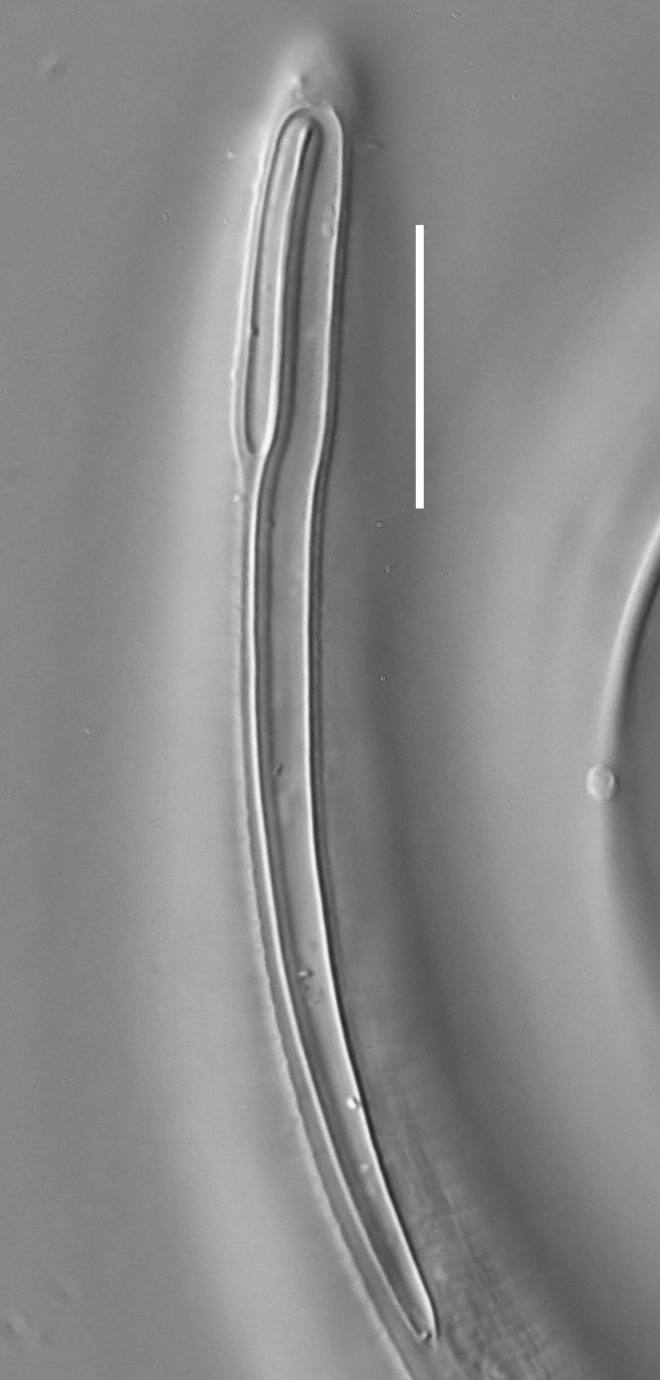
female anterior end, surface view showing amphid (ventral side to the right)

**Figure 28a.**
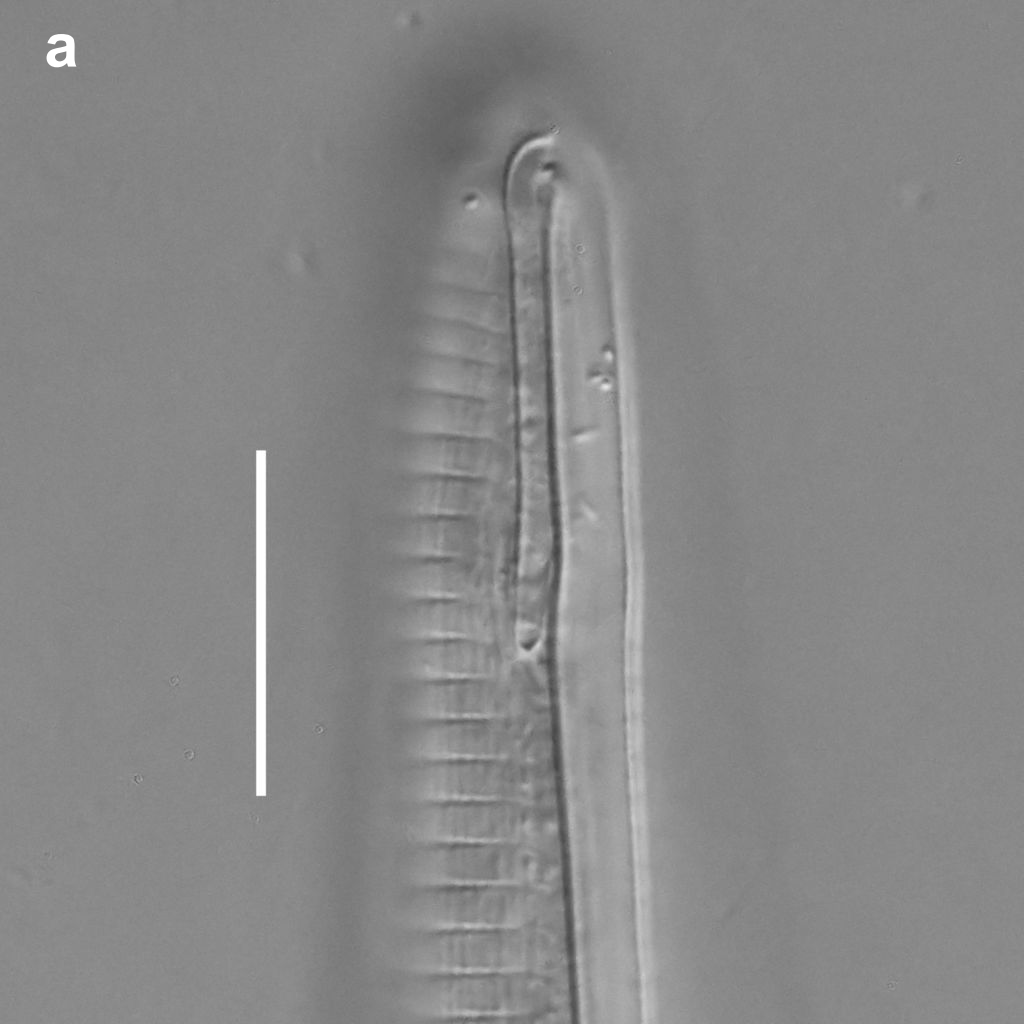
male anterior end, surface view showing anterior end of amphid and cuticle (ventral side to the right)

**Figure 28b.**
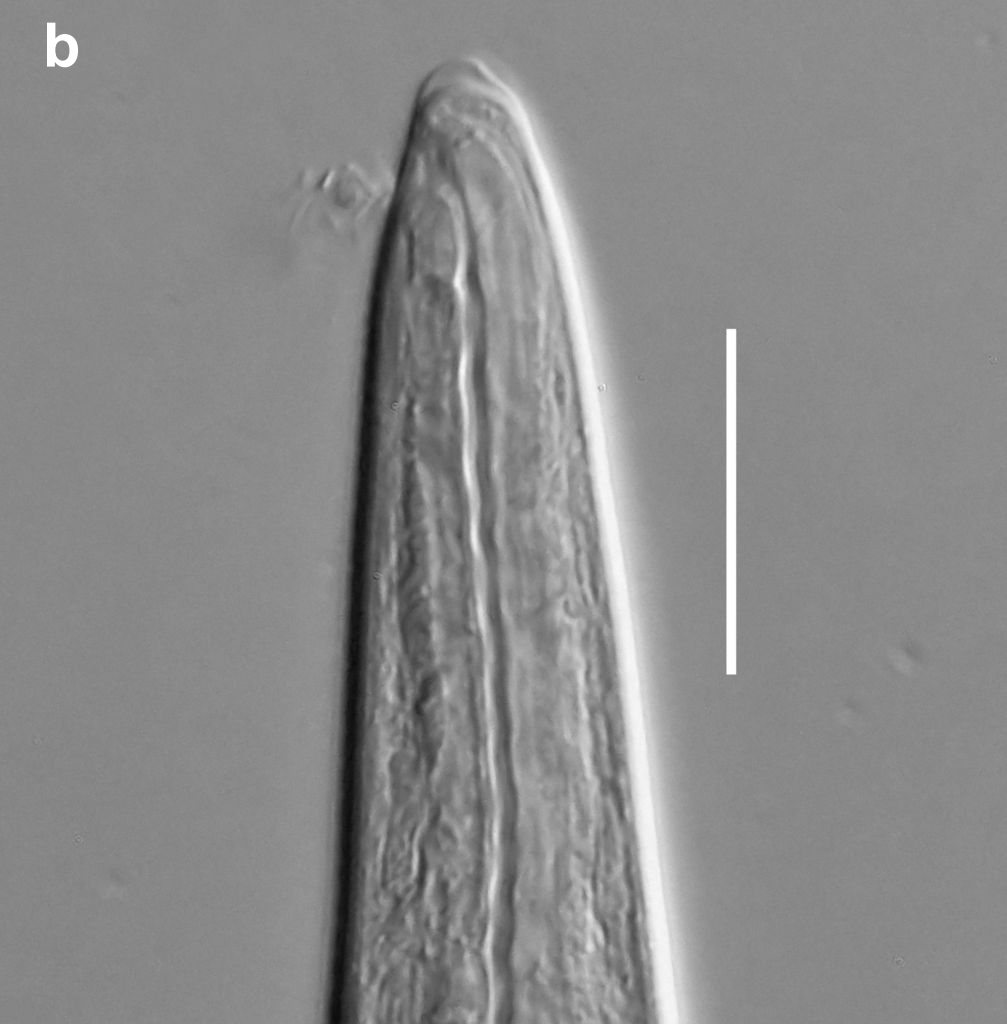
anterior end, median section (ventral side to the right)

**Figure 28c.**
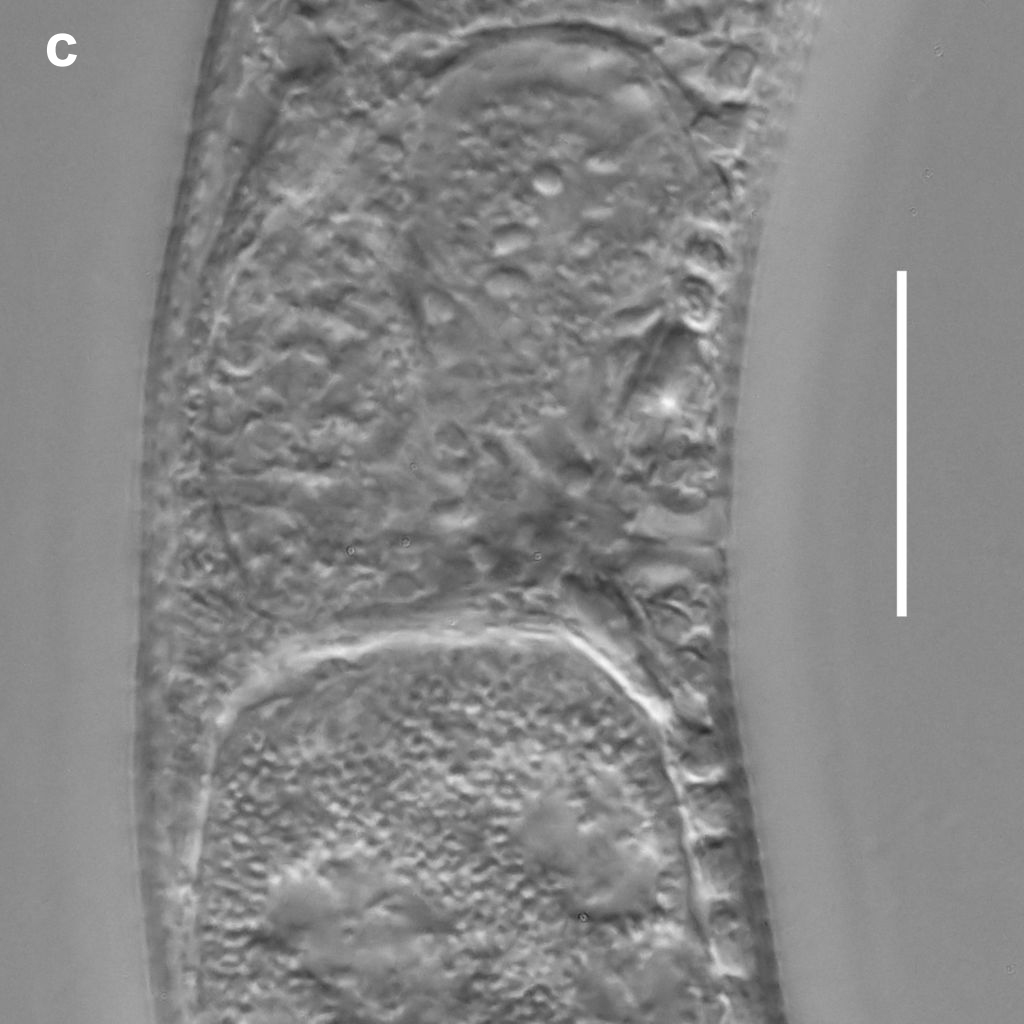
vulval region

**Figure 28d.**
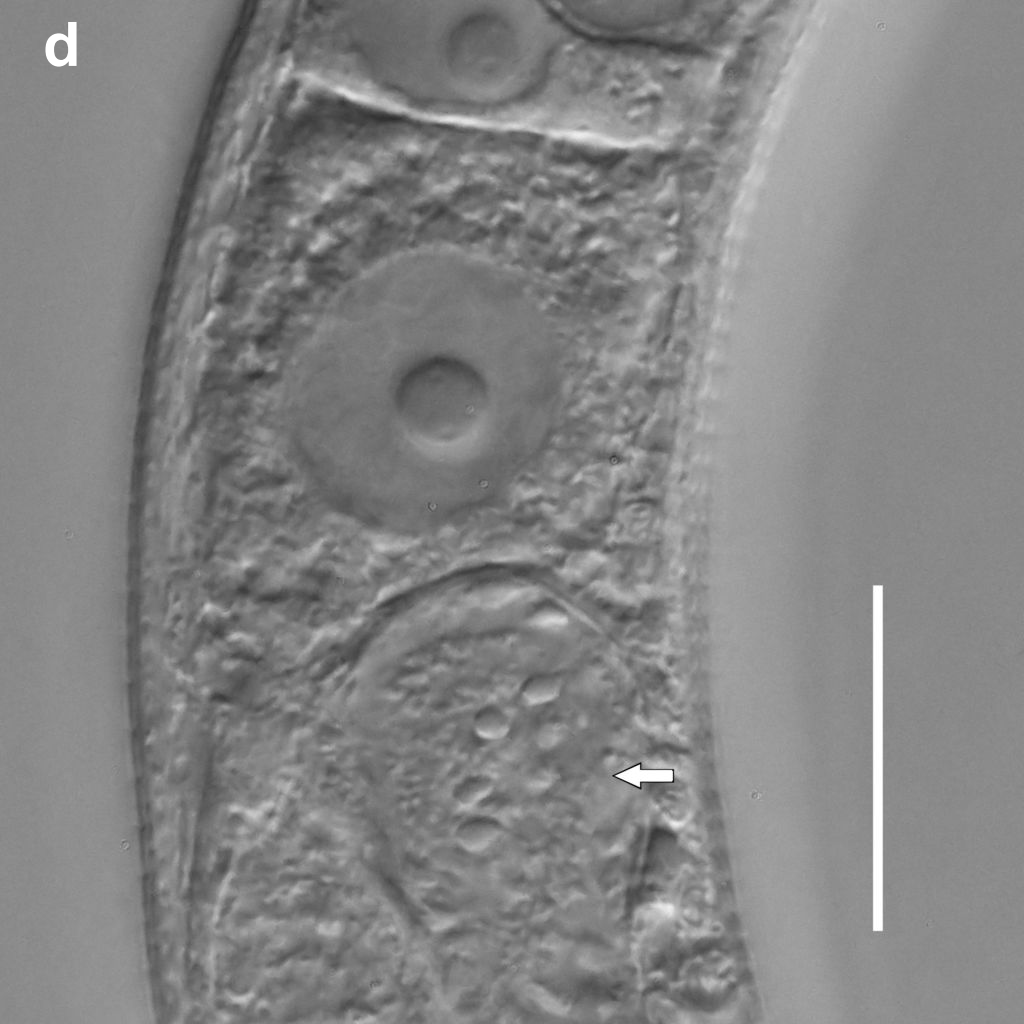
spermatheca and ovocytes

**Figure 30a.**
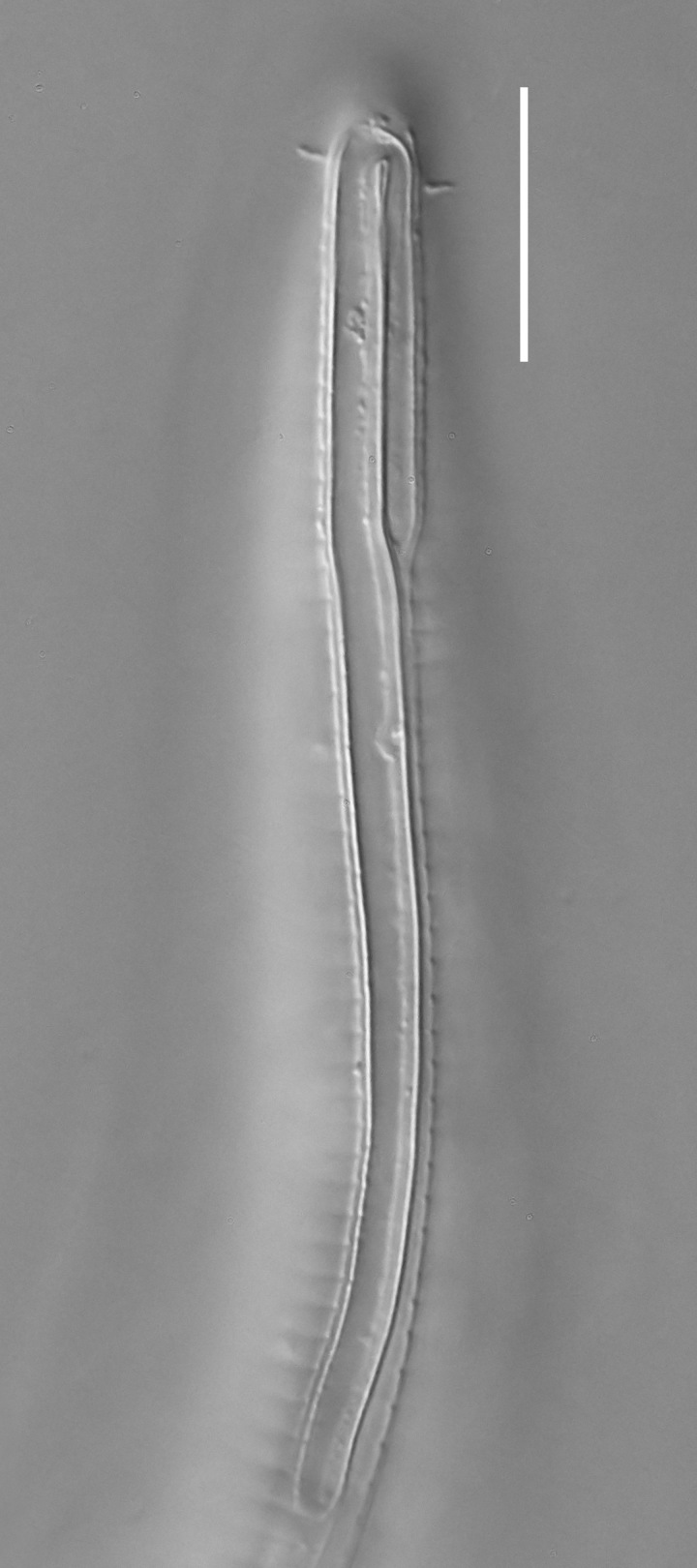
female anterior end, surface view showing amphid (ventral side to the left)

**Figure 30b.**
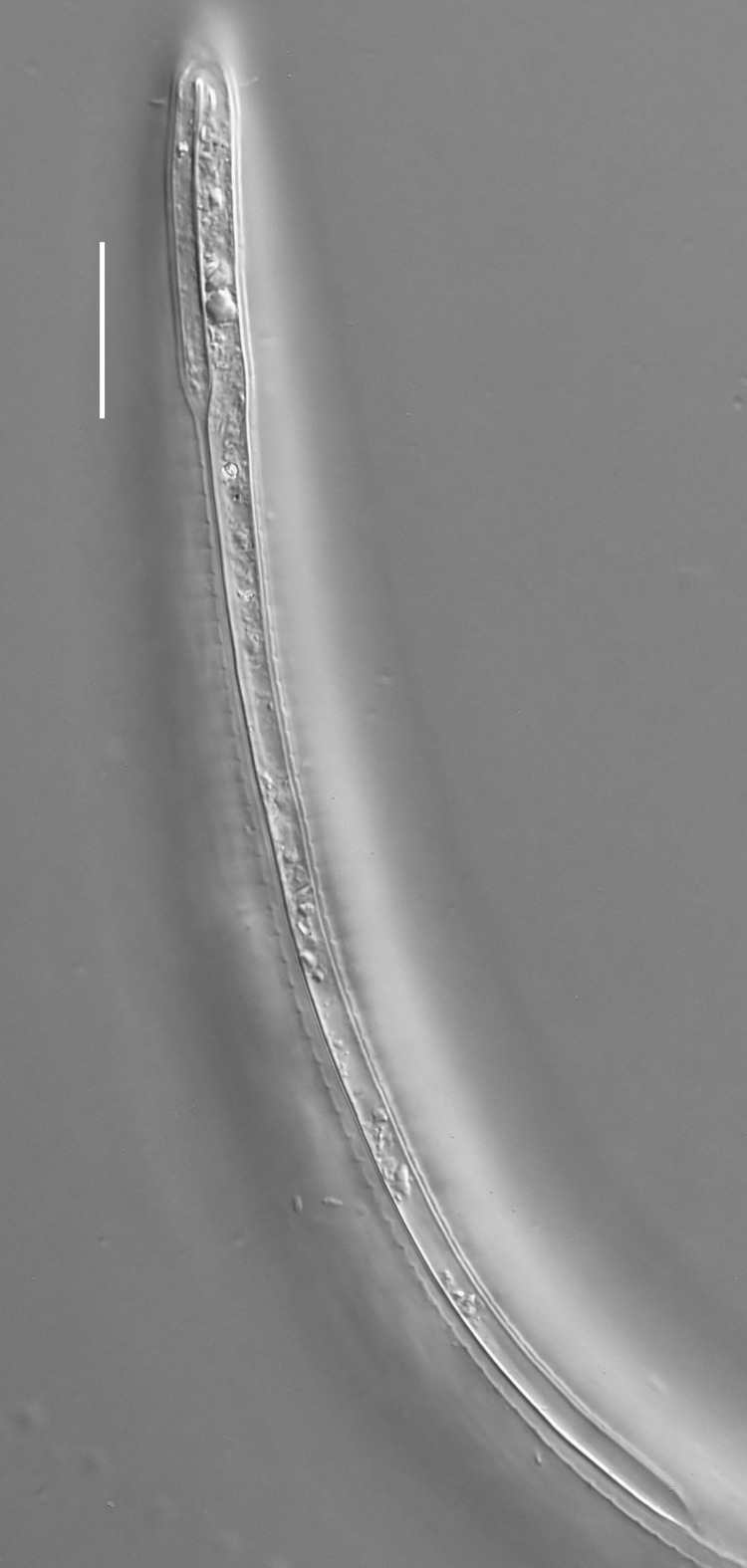
male anterior end, surface view showing amphid (ventral side to the right)

**Figure 31a.**
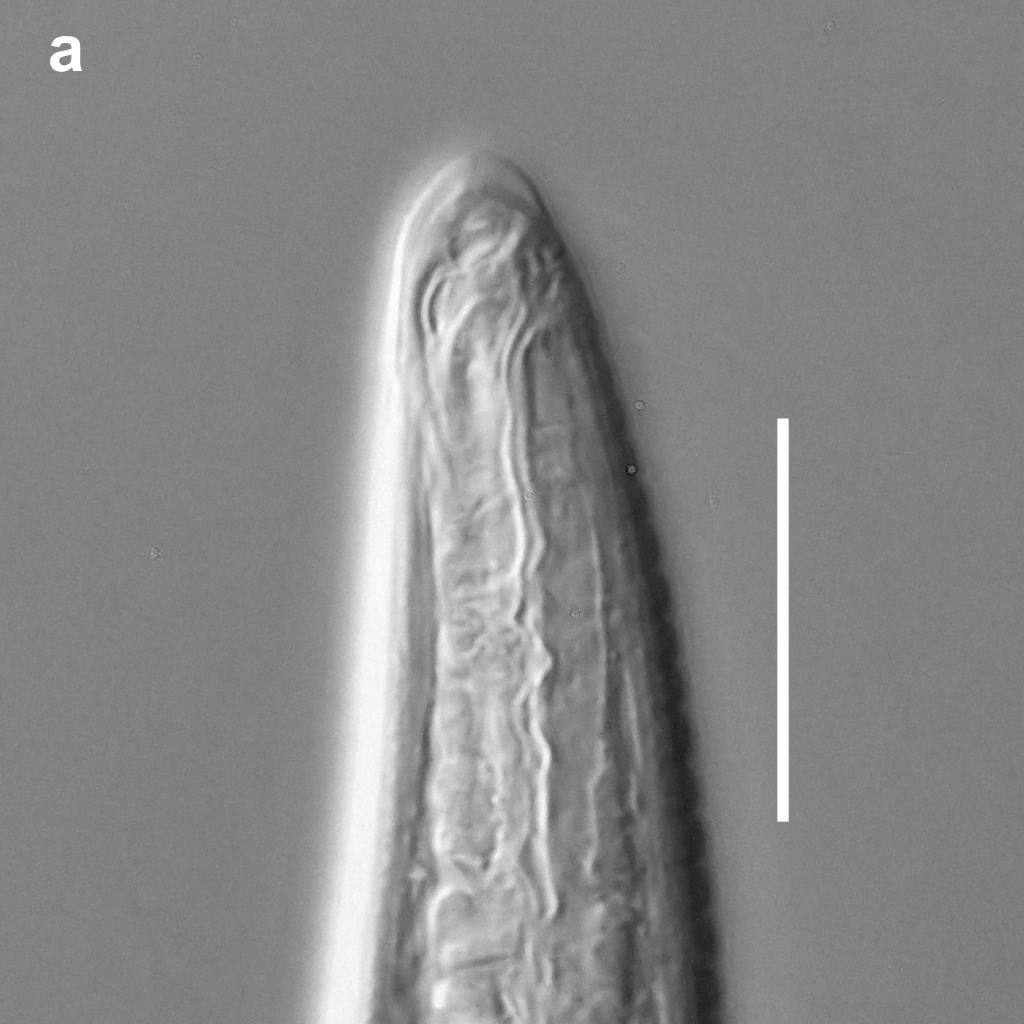
anterior end, median section (ventral side to the left)

**Figure 31b.**
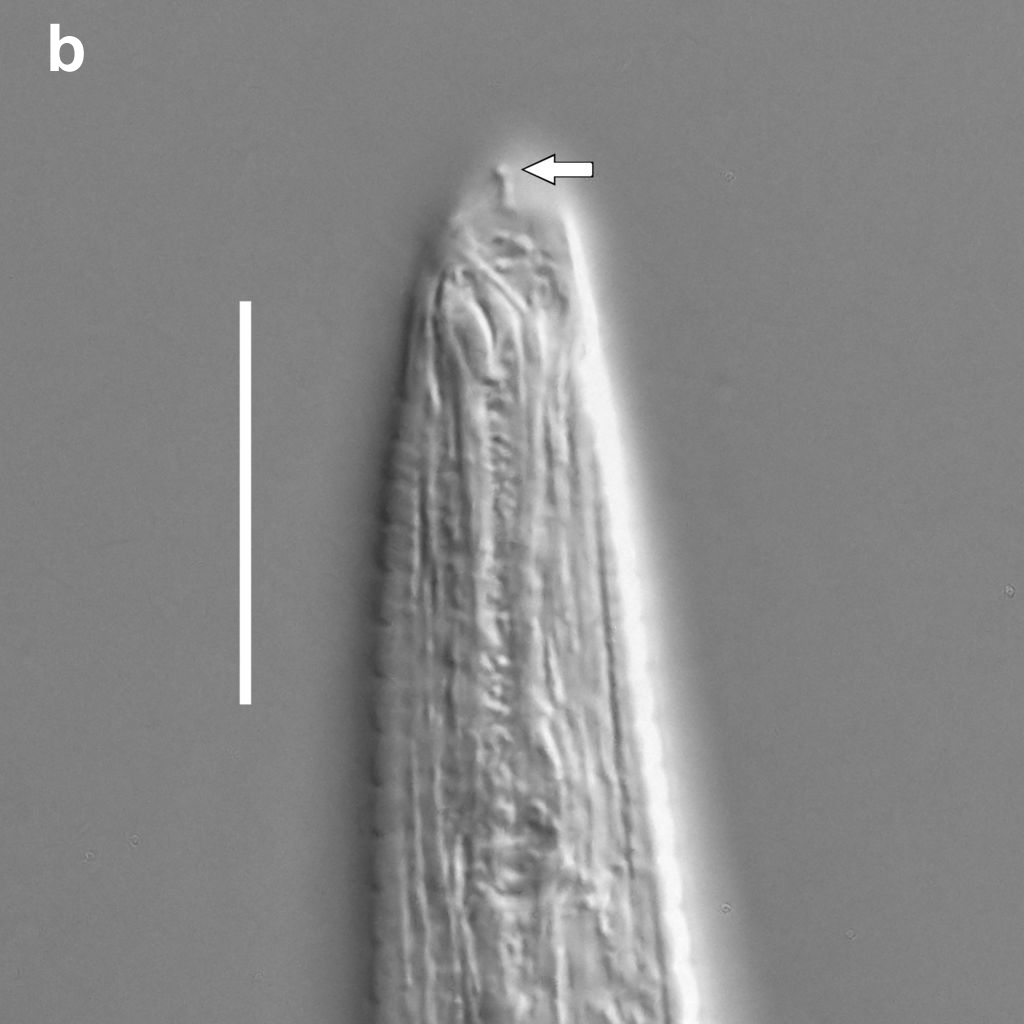
anterior end, surface view showing lateral outer labial sensillum (arrow) (ventral side to the right)

**Figure 31c.**
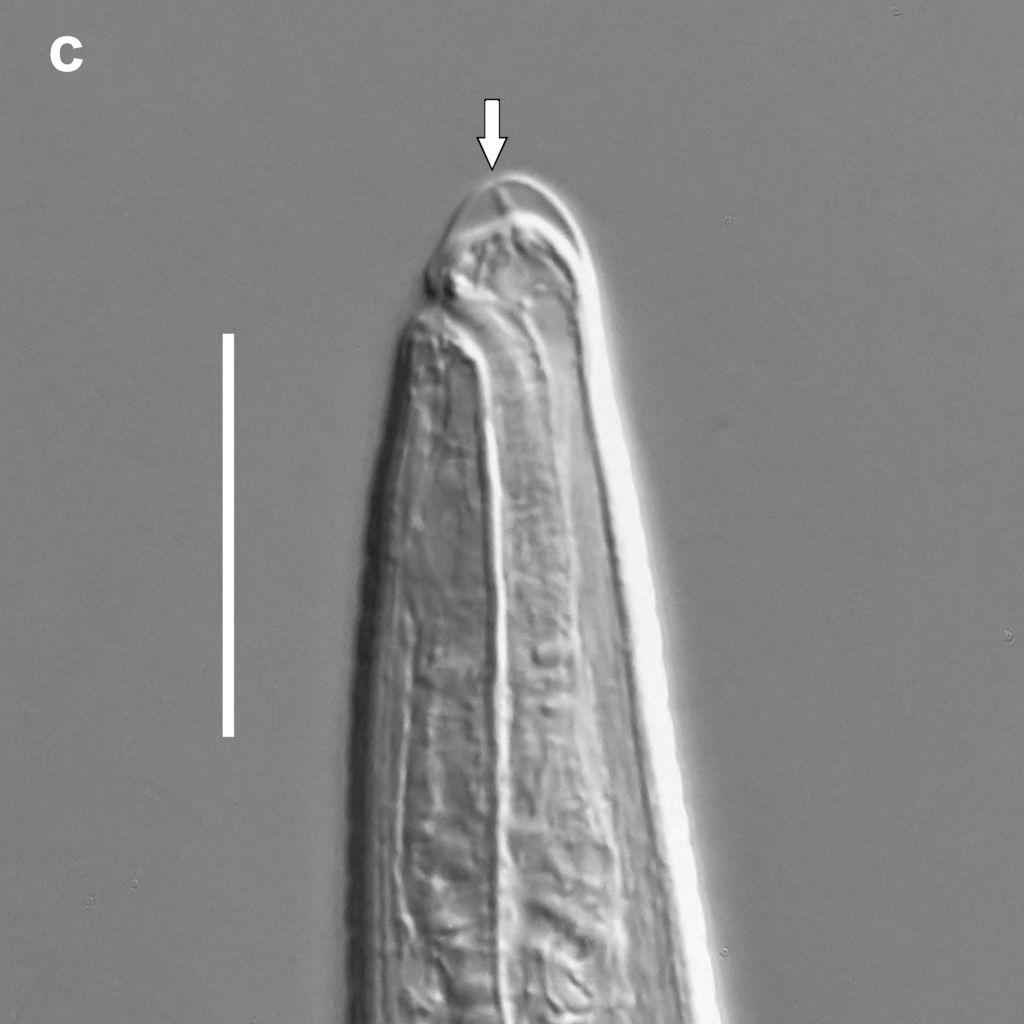
anterior end, median section showing secretory-excretory pore (arrow) (ventral side to the right)

**Figure 31d.**
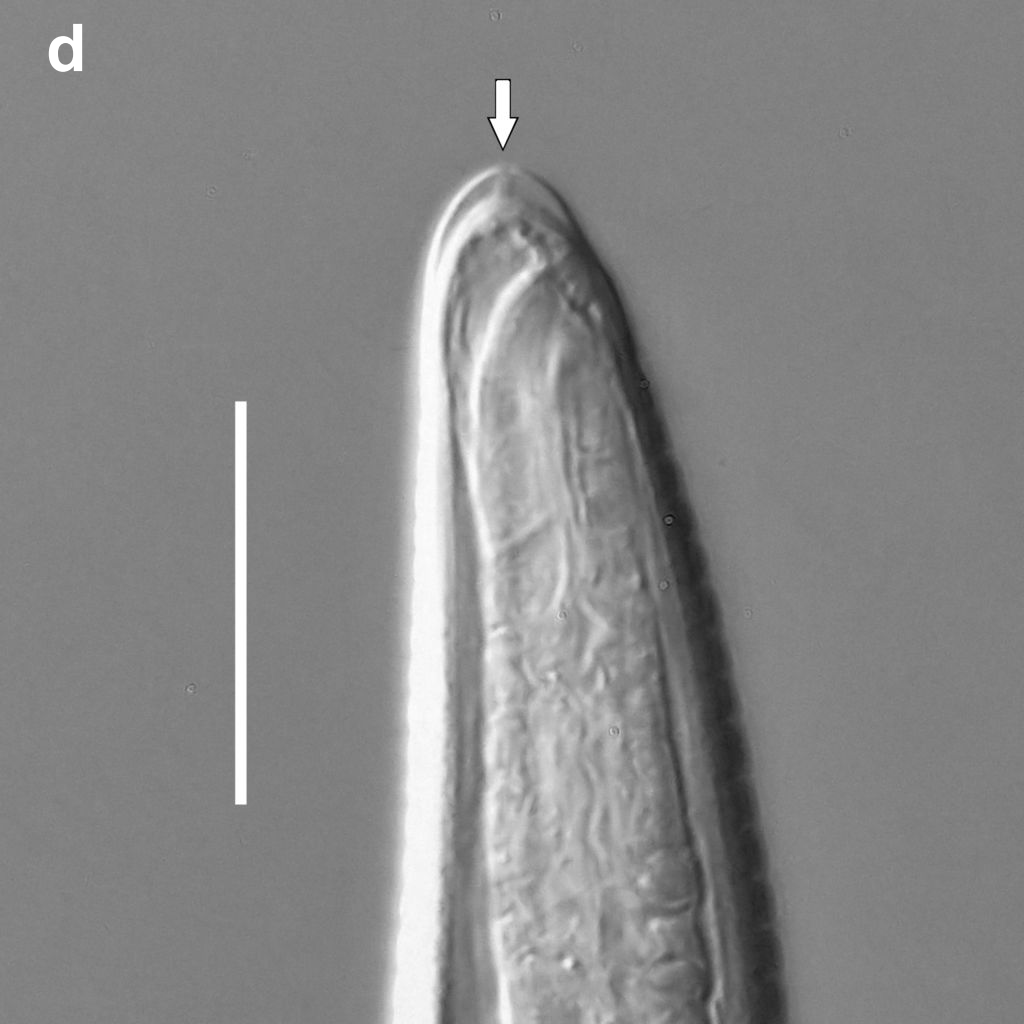
anterior end, median section showing secretory-excretory pore (arrow) (ventral side to the left)

**Figure 31e.**
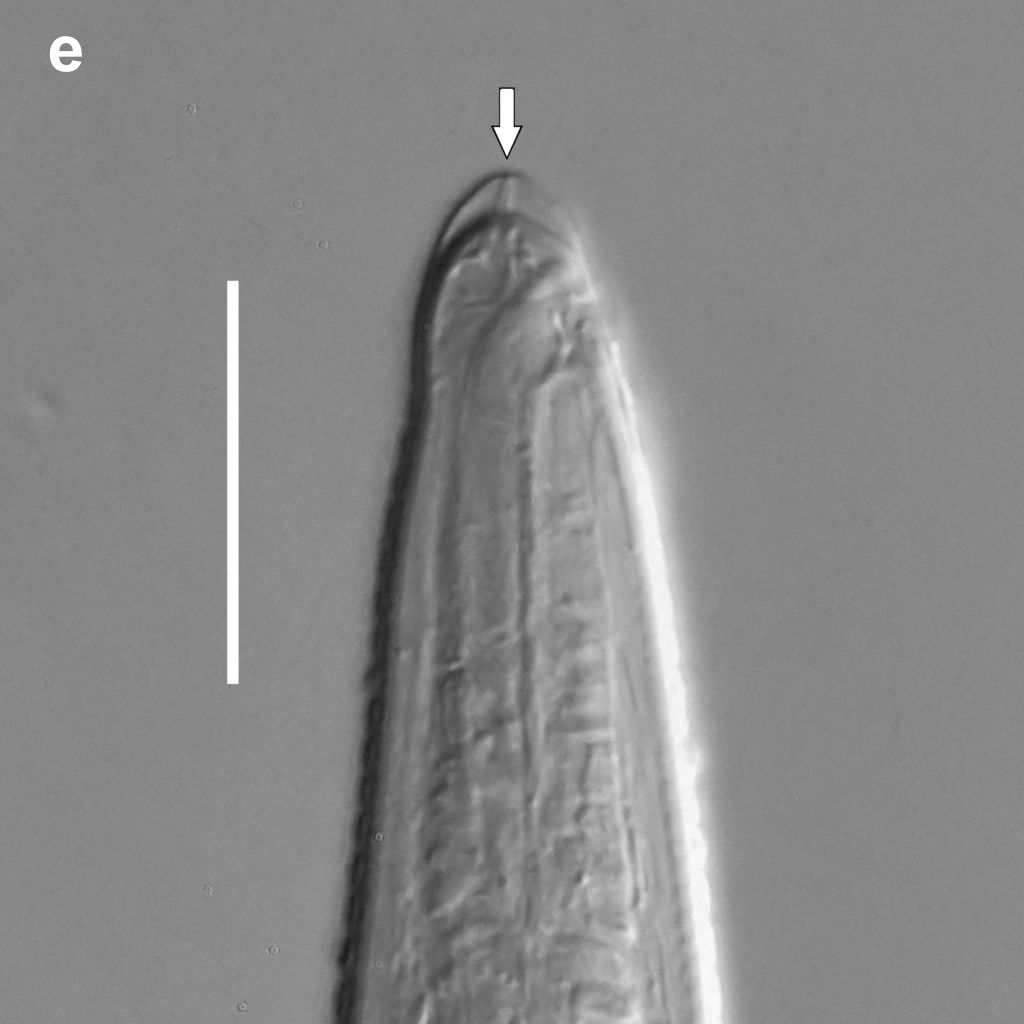
anterior end, median section showing secretory-excretory pore (arrow) (ventral side to the left)

**Figure 31f.**
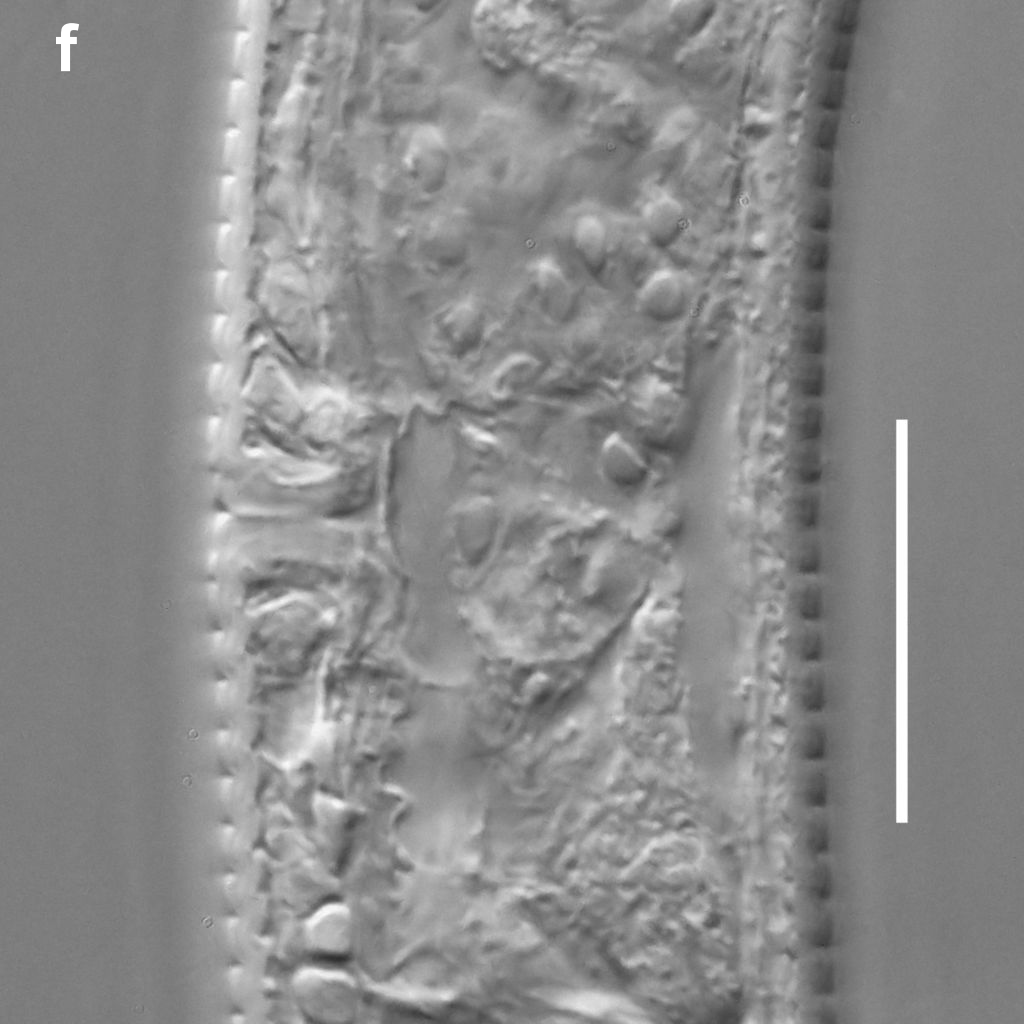
vulval region

**Figure 32a.**
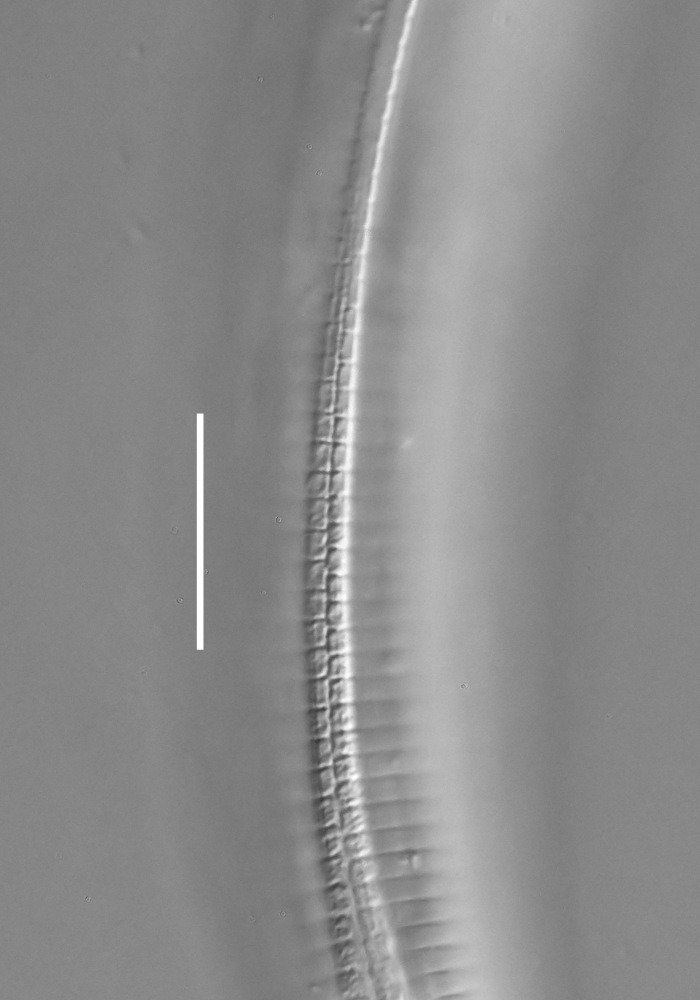
pre-anal region, surface view showing lateral alae

**Figure 32b.**
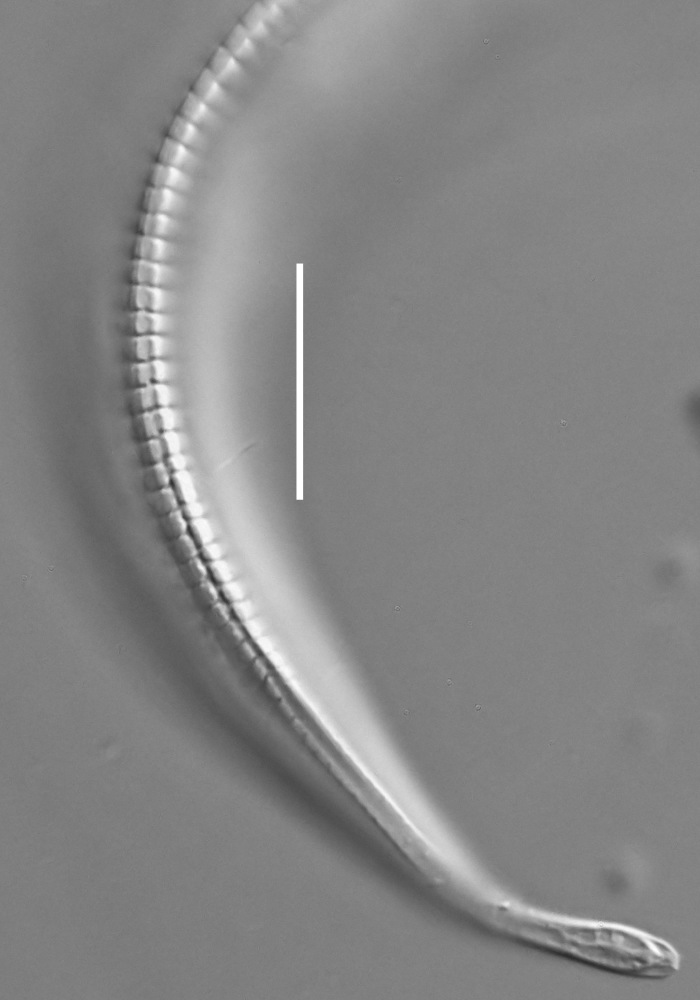
anal region and tail, surface view showing lateral alae

**Figure 32c.**
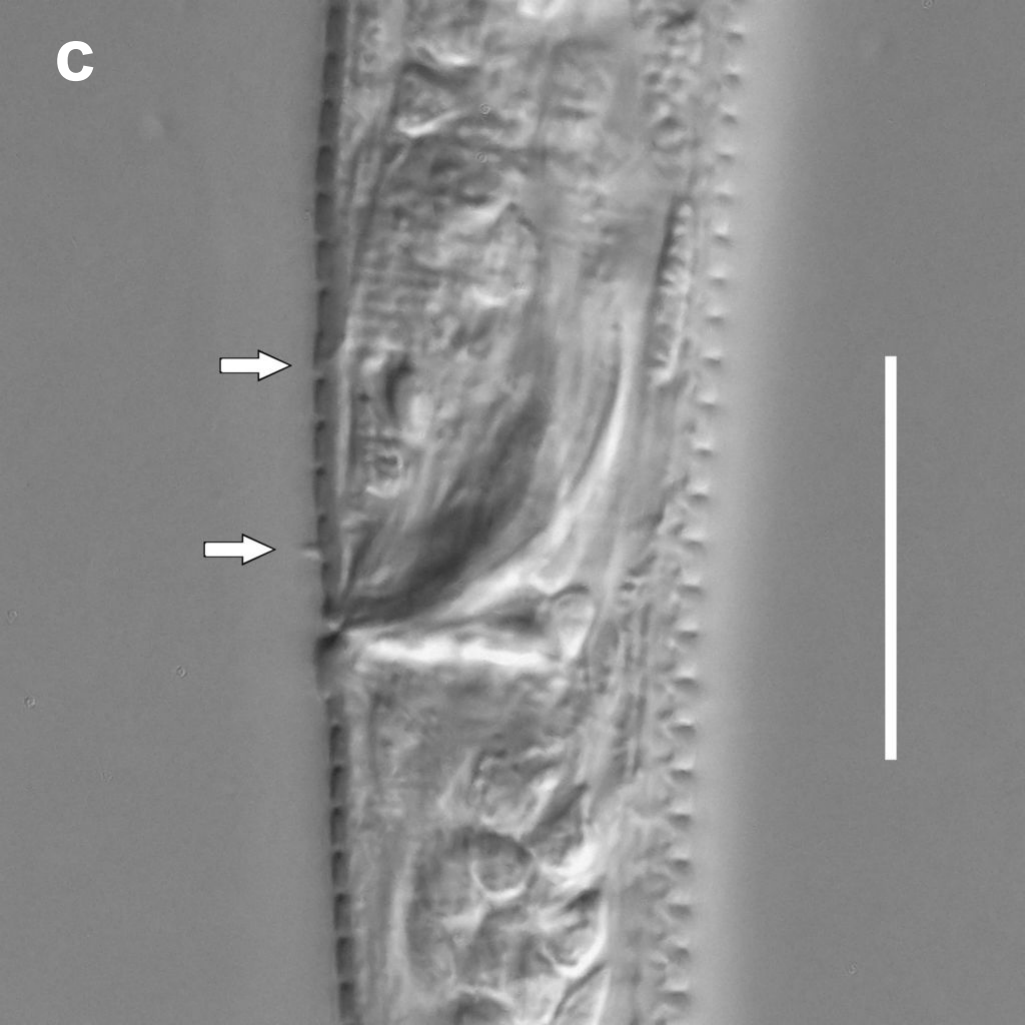
male cloacal region showing precloacal sensilla (arrows)

**Figure 32d.**
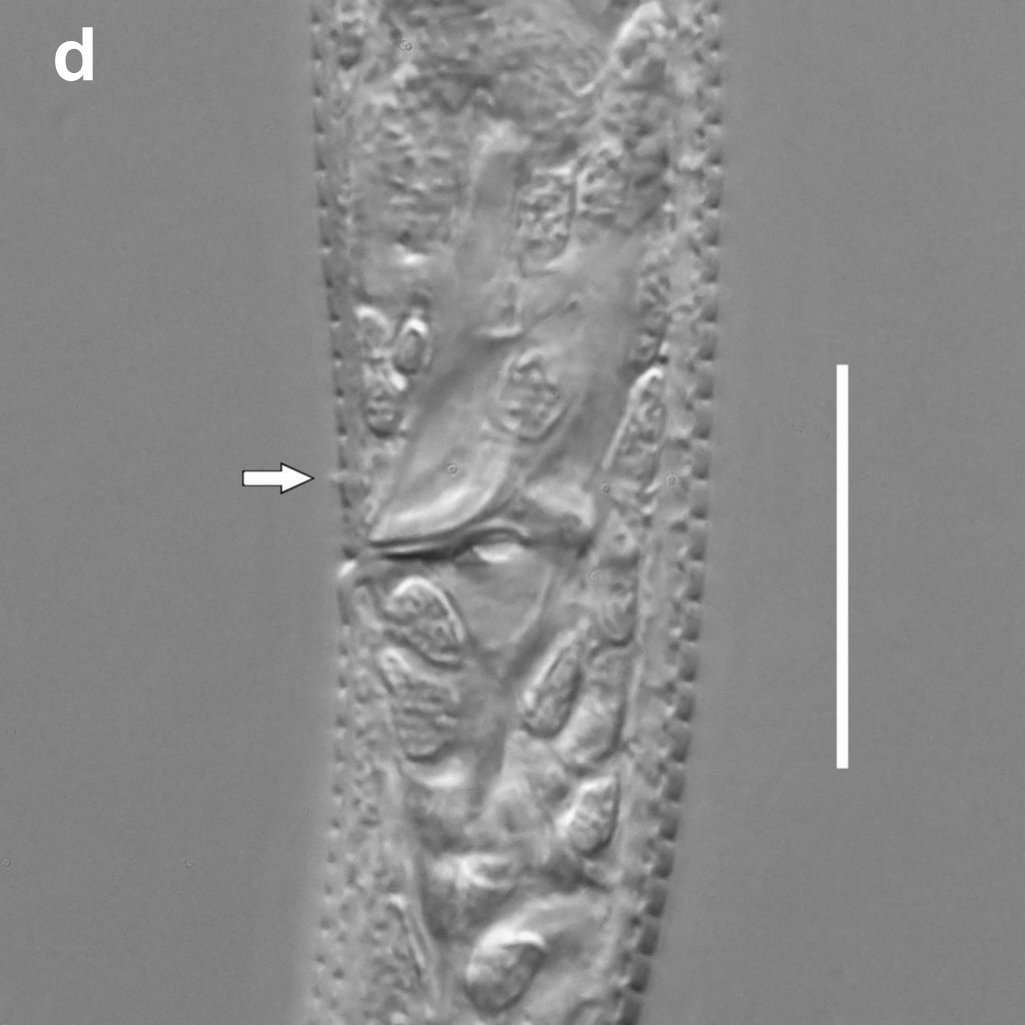
male cloacal region showing precloacal sensillum (arrow) and gubernaculum

**Figure 33a.**
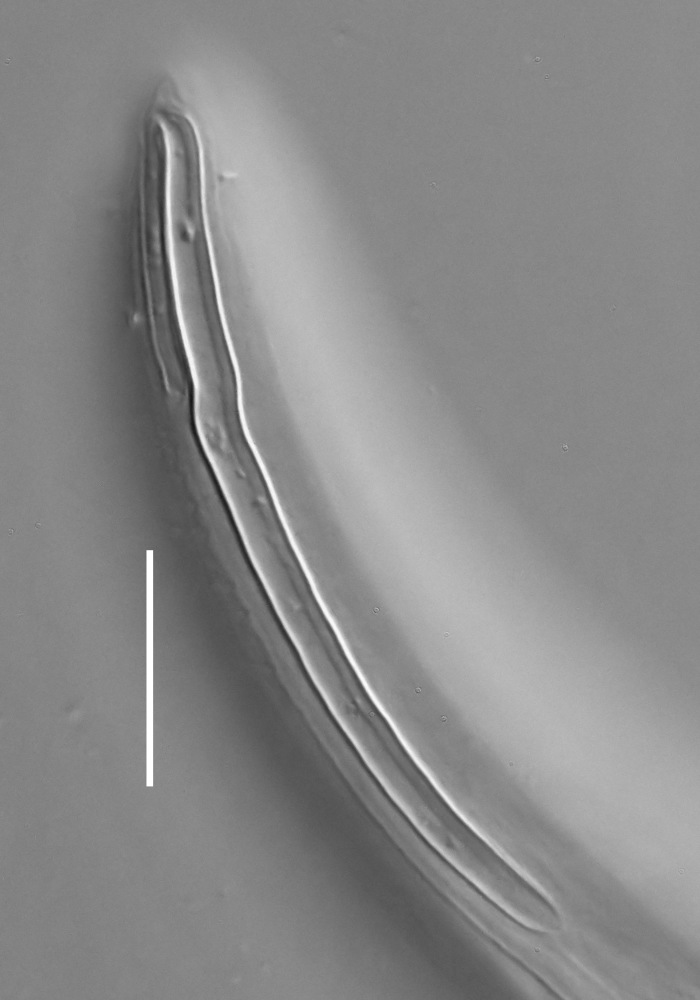
male anterior end, surface view showing amphid (ventral side to the right)

**Figure 33b.**
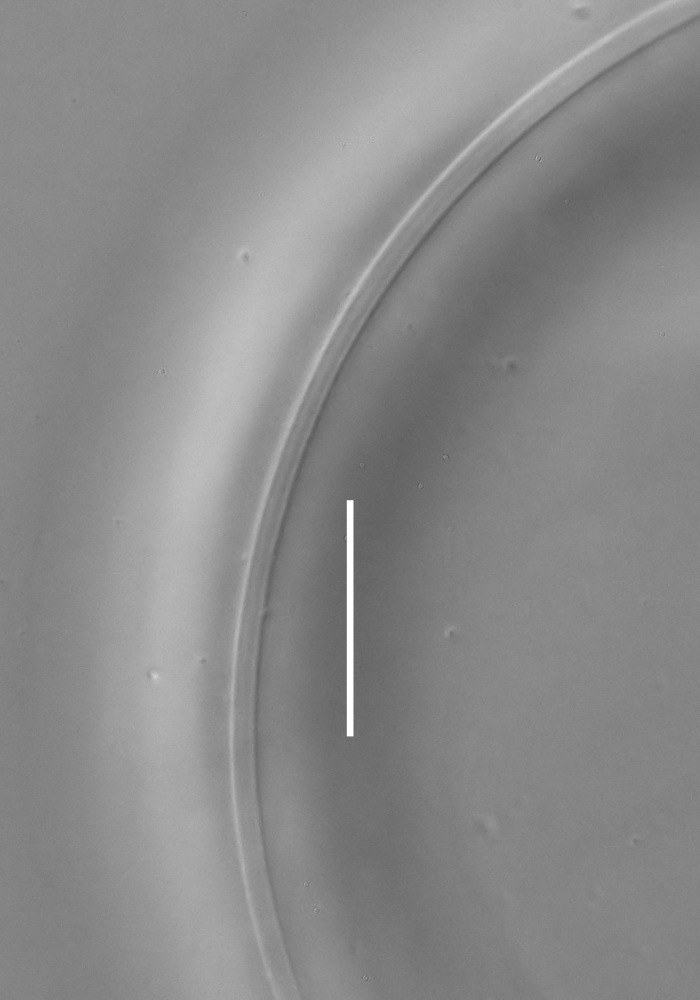
lateral alae at mid-body, surface view

**Figure 33c.**
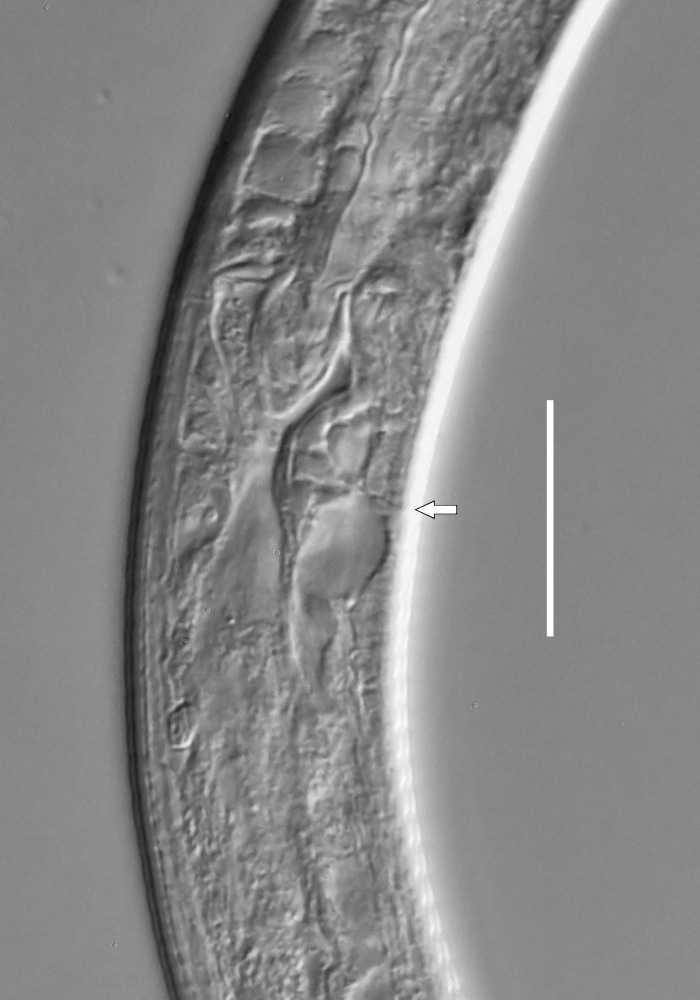
secretory-excretory pore just posterior to pharyngo-intestinal junction (arrow)

**Figure 33d.**
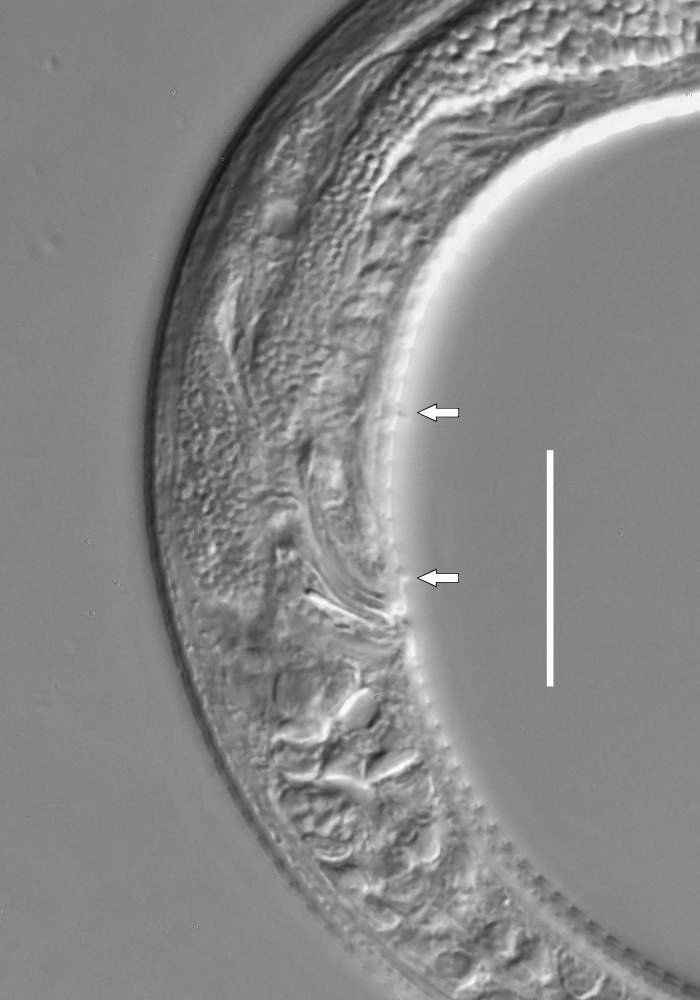
male cloacal region showing precloacal papilla (arrows)

**Table 1.** Morphometrics of *Campylaimus gerlachi* Timm, 1961 and *Campylaimus minutus* Fadeeva, Mordukhovich & Zograf, 2016.

Species	*C. gerlachi*		*C. minutus*	
Number and gender	3 males	4 females	2 males	5 females
Body length	416 (373–471)	449 (343–515)	504; 382	422±80 (355–555)
Body diameter	15 (14–16)	20 (18–23)	14; 14	19±5 (12–26)
Pharyngeal region length	84 (77–91)	94 (68–110)	84; 86	85±15 (70–107)
Tail length	68 (61–75)	84 (65–98)	58; 60	59±12 (49–79)
Cloacal/anal body diameter	13 (12–13)	12 (11–12)	12; 12	12±5 (8–19)
a	28 (26–30)	22 (19–26)	35; 28	24±5 (20–32)
b	5.0 (4.8–5.2)	4.8 (4.4–5.2)	6.0; 4.4	5.2±0.5 (4.6–5.7)
c	6.1 (5.9–6.3)	5.3 (5.3–5.5)	8.7; 6.4	7.2±0.2 (7.0–7.5)
c'	5.3 (4.7–5.7)	7.3 (5.5–8.9)	4.6; 5.1	5.3±0.9 (4.1–6.0)
V	–	52 (50–53)	–	53±1 (52–54)
Labial region diameter	8	8 (8–9)	7; 6	6±1 (5–8)
Cephalic setae length	2	2 (1–2)	2; 2	1
Subdorsal setae bases from anterior end	9 (9–10)	10 (8–13)	8; 7	8±3 (6–10)
Subventral setae bases from anterior end	9 (8–10)	8	7; 7	7±2 (5–8)
Oral opening from anterior end	5 (3–6)	5 (4–6)	5; 5	4±1 (3–6)
Amphid from anterior end	6 (6–7)	6 (6–8)	5; 5	6±1 (5–6)
Dorsal amphideal limb length	19 (18–21)	19 (17–19)	15; 14	13±2 (11–17)
Dorsal amphideal limb width	2 (1–2)	1	1; 1	2 (1–2)
Ventral amphideal limb width	2	2	1; 2	2±1 (2–3)
Annules width at mid-body	2	1 (1–2)	?; 1	1 (1–2)
Lateral field width	3 (2–3)	3 (2–3)	2; 3	3±1 (2–4)
Vagina or spicules length	18 (14–22)	4 (3–5)	16; 16	4±1 (3–6)
Rectum or Gubernaculum length	4 (3–5)	14 (11–17)	3; 3	11±3 (9–16)

**Table 2.** Morphometrics of *Campylaimus tkatchevi* Tchesunov, 1978, *Campylaimus siwaschensis* Sergeeva, 1981 and *Campylaimus lefeverei* Gerlach, 1956.

Species	*C. tkatchevi*		*C. siwaschensis*	*C. lefeverei*
Number and gender	4 males	3 females	1 female	1 female
Body length	658 (604–704)	636 (562–730)	606	959
Body diameter	26 (23–28)	29 (23–34)	28	25
Pharyngeal region length	120 (116–124)	117 (110–126)	88	131
Tail length	116 (109–128)	109 (100–119)	93	210
Cloacal/anal body diameter	21 (19–23)	20 (19–21)	16	14
a	25 (24–27)	22 (18–25)	22	39
b	5.5 (5.1–6.1)	5.4 (5.1–5.8)	6.9	7.3
c	5.7 (5.4–6.0)	6.2 (6.1–6.2)	6.5	4.6
c'	5.5 (5.2–5.6)	5.4 (4.8–5.9)	5.8	14.5
V	–	52 (52–53)	49	47
Labial region diameter	11 (10–12)	11 (11–12)	12	14
Cephalic setae length	2 (2–3)	2 (1–2)	2	4
Subdorsal setae bases from anterior end	14 (14–15)	13 (12–14)	8	8
Subventral setae bases from anterior end	11 (10–13)	11 (10–13)	6	7
Oral opening from anterior end	9 (8–10)	8 (8–9)	2	1
Amphid from anterior end	8 (8–10)	9 (8–11)	3	4
Dorsal amphideal limb length	27 (26–28)	24 (20–28)	14	22
Dorsal amphideal limb width	2	2 (1–2)	2	2
Ventral amphideal limb width	4 (3–4)	4 (3–4)	3	6
Annules width at mid-body	3 (2–3)	2	1	2
Lateral field width	8 (7–8)	7 (5–8)	3	12
Vagina or spicules length	28 (27–30)	7 (6–8)	6	5
Rectum or Gubernaculum length	7 (6–8)	23 (22–26)	18	19

**Table 3.** Morphometrics of *Campylaimus rimatus* Vitiello, 1974 and *Campylaimus orientalis* Fadeeva, Mordukhovich & Zograf, 2016.

Species	*C. rimatus*		*C. orientalis*	
Number and gender	5 males	4 females	3 males	5 females
Body length	687±36 (649–732)	663 (634–700)	605 (583–625)	616±35 (579–653)
Body diameter	28±1 (26–29)	34 (28–40)	25 (22–29)	35±8 (25–44)
Pharyngeal region length	115±4 (112–121)	109 (103–119)	93 (88–98)	101±4 (95–107)
Tail length	102±5 (98–109)	95 (88–103)	90 (89–91)	92±6 (84–98)
Cloacal/anal body diameter	23±1 (21–25)	20 (17–22)	21 (19–23)	21±2 (18–23)
a	25±1 (23–25)	20 (17–23)	24 (21–26)	19±4 (15–23)
b	6.0±0.2 (5.6–6.2)	6.1 (5.8–6.5)	6.4 (6.2–6.7)	6.1±0.5 (5.7–6.9)
c	6.7±0.4 (6.3–7.2)	7.0 (6.6–7.2)	6.7 (6.4–7.0)	6.7±0.1 (6.6–6.9)
c'	4.5±0.5 (4.1–5.1)	4.8 (4.4–5.3)	4.3 (3.9–4.7)	4.6±0.5 (4.3–5.3)
V	–	51 (50–52)	–	50±1 (49–51)
Labial region diameter	12 (11–12)	12 (12–12)	13 (13–14)	14±1 (13–16)
Cephalic setae length	2 (2–3)	2 (2–3)	2 (2–3)	2 (2–3)
Subdorsal setae bases from anterior end	11 (11–12)	13 (12–14)	12 (12–12)	11±1 (10–12)
Subventral setae bases from anterior end	11±1 (10–12)	11 (10–12)	11	9±1 (9–11)
Oral opening from anterior end	7 (6–8)	7 (7–8)	5 (3–7)	7±2 (6–10)
Amphid from anterior end	5±1 (3–6)	4 (4–6)	4 (2–6)	3±1 (2–5)
Dorsal amphideal limb length	28±2 (26–31)	25 (23–26)	27 (26–30)	27±2 (26–30)
Dorsal amphideal limb width	2	2	2 (2–3)	2±1 (1–3)
Ventral amphideal limb length	62±4 (59–66)	45 (42–48)	35 (33–37)	35±4 (32–43)
Ventral amphideal limb width	2	2	3 (2–3)	2 (1–2)
Interamphideal space length	60±3 (57–64)	42 (39–46)	32 (31–34)	31±3 (28–35)
Interamphideal space width	2±1 (2–3)	2 (2–3)	3 (2–3)	3 (2–3)
Annules width at mid-body	2	2	2	2
Lateral field width	3±1 (2–4)	3 (2–3)	1	2 (1–2)
Vagina or spicules length	25±1 (23–26)	6 (6–7)	26 (23–27)	7±1 (7–8)
Rectum or Gubernaculum length	6±2 (4–8)	19 (18–20)	6	21±2 (18–23)

**Table 4.** Morphometrics of *Campylaimus triclados* sp. n. and *Campylaimus mirus* Gerlach, 1950.

Species	*C. triclados* sp. n.			*C. mirus*
Number and gender	holotype male	5 males (incl. holotype)	2 females	1 female
Body length	588	615±19 (588–632)	551; 691	932
Body diameter	23	24±2 (21–28)	27; 29	33
Pharyngeal region length	?	109±6 (102–116)	107; 107	141
Tail length	84	92±5 (84–98)	77; 93	152
Cloacal/anal body diameter	19	19±1 (19–21)	16; 17	21
a	26	26±2 (23–29)	21; 24	28
b	?	5.7±0.3 (5.5–6.2)	5.2; 6.5	8.2
c	7.0	6.7±0.2 (6.4–7.0)	7.2; 7.5	6.1
c'	4.3	4.7±0.3 (4.3–5.1)	4.9; 5.6	7.3
V	–	–	50; 48	47
Labial region diameter	10	11±2 (8–14)	11; 10	19
Cephalic setae length	2	2 (1–2)	2; 2	6
Subdorsal setae bases from anterior end	9	9±0 (9–10)	8; 9	11
Subventral setae bases from anterior end	7	7±1 (7–8)	6; 6	8
Oral opening from anterior end	6	6±1 (4–7)	6; 4	5
Amphid from anterior end	4	3±1 (2–4)	2; 3	3
Dorsal amphideal limb length	21	22±1 (21–23)	21; 20	33
Dorsal amphideal limb width	2	2±1 (1–3)	1; 1	2
Ventral amphideal limb length	106	107±14 (89–126)	46; 55	46
Ventral amphideal limb width	2	2 (1–2)	2; 2	3
Interamphideal space length	37	35±5 (29–41)	35; 33	44
Interamphideal space width	2	2 (1–2)	3; 2	7
Annules width at mid-body	2	2	2; 3	2
Lateral field width	1	2 (1–2)	1; 1	2
Vagina or spicules length	22	22±2 (19–24)	6; 6	4
Rectum or Gubernaculum length	?	6 (6–6)	20; 18	23

**Table 5.** Morphometrics of *Campylaimus inaequalis* Cobb, 1920 and *Campylaimus striatus* Boucher & Helléouöt, 1977.

Species	*C. inaequalis*		*C. striatus*	
Number and gender	male	2 females	2 males	4 females
Body length	642	567; 525	728; 618	516–557
Body diameter	21	27; 27	24; 25	28–34
Pharyngeal region length	?	109; 96	119; 105	100–107
Tail length	100	98; 88	112; 107	82–89
Cloacal/anal body diameter	17	19; 19	21; 19	17–19
a	30.0	21.1; 19.5	30.1; 24.9	15.4–19.2
b	?	5.2; 5.5	6.1; 5.9	5.1–5.2
c	6.4	5.8; 6.0	6.5; 5.8	5.8–6.4
c'	5.8	5.3; 4.5	5.4; 5.7	4.6–5.1
V	–	53.1; 49.7	–	49.7–51.9
Labial region diameter	13	12; 12	12; 12	9–11
Cephalic setae length	?	3; 3	?; 3	1–2
Subdorsal setae bases from anterior end	?	13; 13	?; 10	9–10
Subventral setae bases from anterior end	?	9; 9	?; 8	8–9
Oral opening from anterior end	7	8; 6	3; 6	4–6
Amphid from anterior end	3	3; 4	4; 5	3–6
Dorsal amphideal limb length	30	25; 23	23; 24	20–24
Dorsal amphideal limb width	2	2; 2	2; 1	2
Ventral amphideal limb length	50	50; 44	224; 199	79–113
Ventral amphideal limb width	4	4; 5	3; 3	2–4
Annules width at mid-body	?	2; 2	3; 2	1–2
Lateral field width	1	2; 2	1; 1	1–2
Vagina or spicules length	24	9; 6	27; 26	6–9
Rectum or Gubernaculum length	6	21; 19	7; 6	12–22

**Table 6.** Morphometrics of *Campylaimus amphidialis* Fadeeva, Mordukhovich & Zograf, 2016 and *Campylaimus longispiculus* sp. n.

Species	*C. amphidialis*		*C. longispiculus* sp. n.	
Number and gender	6 males	2 females	holotype male	3 males (incl. holotype)
Body length	582±30 (534–627)	571; 588	635	615 (560–649)
Body diameter	24±3 (20–28)	23; 28	21	20 (19–21)
Pharyngeal region length	105±8 (93–114)	128; 107	114	107 (100–114)
Tail length	102±7 (91–107)	103; 95	98	94 (86–98)
Cloacal/anal body diameter	19±2 (17–21)	17; 19	20	18 (17–20)
a	25±4 (21–29)	24; 21	30	30
b	5.6±0.3 (5.3–6.2)	4.5; 5.5	5.6	5.7 (5.6–6.0)
c	5.7±0.5 (5.0–6.4)	5.5; 6.2	6.5	6.5 (6.5–6.6)
c'	5.6±0.8 (4.5–6.4)	6.2; 5.1	4.9	5.1 (4.9–5.3)
V	–	52; 52	–	–
Labial region diameter	10±1 (9–10)	9; 10	10	10 (10–11)
Cephalic setae length	2 (2–3)	1; 3	3	2 (2–3)
Subdorsal setae bases from anterior end	11±1 (10–12)	10; 12	13	12 (11–13)
Subventral setae bases from anterior end	9±1 (8–10)	8; 10	10	9 (8–10)
Oral opening from anterior end	6±1 (4–7)	8; 8	8	7 (7–8)
Amphid from anterior end	7±1 (6–8)	6; 8	3	3
Dorsal amphideal limb length	31±3 (28–37)	28; 29	22	21 (20–22)
Dorsal amphideal limb width	2 (2–3)	2; 2	3	2 (1–3)
Ventral amphideal limb length	150±14 (132–171)	94; 92	67	66 (57–73)
Ventral amphideal limb width	4 (3–4)	3; 4	3	3
Annules width at mid-body	2	2; 2	2	2
Lateral field width	2 (1–2)	1; 2	2	2 (1–2)
Vagina or spicules length	25±2 (24–29)	6; 7	35	32 (28–35)
Rectum or Gubernaculum length	7±1 (6–8)	17; 21	7	7
